# Tailoring the major groove of DNA mimic foldamers

**DOI:** 10.1039/d6sc00798h

**Published:** 2026-06-09

**Authors:** Jiaojiao Wu, Valentina Corvaglia, Tulika Chakrabortty, Pradeep K. Mandal, Ivan Huc

**Affiliations:** a Department Pharmazie, Ludwig-Maximilians-Universität München Butenandtstr. 5-13 München 81377 Germany ivan.huc@cup.lmu.de; b Institute of Science and Technology Austria Am Campus 1 Klosterneuburg 3400 Austria

## Abstract

Single-stranded, helically folded aromatic oligoamides bearing anionic phosphonate side chains have been shown to bind to some DNA-binding proteins better than DNA itself. However, these DNA mimic foldamers have until now mainly consisted of a single repeat motif, like a poly(dA:dT) DNA duplex, and contained limited sequence information. Here, we introduce new monomers designed to display different chemical functionalities in the major groove of the DNA mimics. Four new Fmoc-protected amino acid monomers have been synthesized and incorporated into oligomers. Sixteen foldamer sequences were prepared on solid phase. Their conformations in solution and in the solid state and their conformational dynamics were investigated using nuclear magnetic resonance, circular dichroism, molecular modeling, and X-ray crystallography. The results show that three of the four new monomers behaved as designed and that their introduction enhances the conformational dynamics of the DNA mimic foldamers. In a fourth case, conformational behavior proved to be more complex than expected. The modified sequences retained the ability to bind to the bacterial histone-like protein HU. These results showcase design strategies to manipulate large molecular biomimetics in which not only side chains but also main chain components are varied. The new monomers pave the way to complex DNA mimic foldamer sequences targeting proteins that recognize sequence-selective DNA-binding proteins such as transcription factors or restriction enzymes.

## Abstract 2

Methods to tailor the width, depth and functionalities of the major groove of DNA mimic foldamers allow departure from the structural homogeneity of the parent series and pave the way to the recognition of sequence-selective DNA-binding proteins.

## Graphical abstract

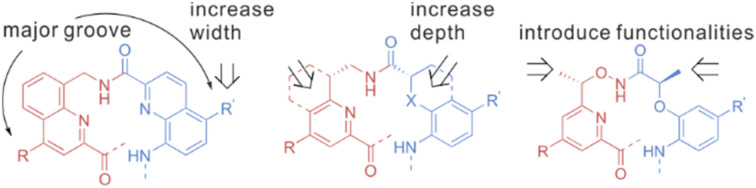

## Introduction

DNA-binding proteins control numerous essential biological processes, making protein–DNA interactions relevant targets for both fundamental research and therapeutic intervention.^1^ In nature, protein–nucleic acid interactions are regulated by a wide array of mechanisms, including small molecule-induced allosteric effects in nuclear receptors,^2,3^ chromatin remodeling,^4^ or post-translational modifications.^5^ Nature has also developed so-called DNA mimic proteins, proteins that reproduce DNA's shape and surface features and that bind to and hijack DNA-binding proteins.^6,7^ Artificial tools to interfere with DNA–protein interactions include small molecules able to block protein–DNA cross-links such as topoisomerase poisons,^8^ molecules that recognize DNA and may prevent protein binding such as pyrrole-imidazole oligoamides,^9,10^ and DNA decoys, which are modified DNA strands that divert proteins from their natural DNA targets.^11,12^

Beyond these advances, DNA mimic foldamers offer a distinct and promising approach. Like DNA mimic proteins, they reproduce some surface features of DNA and bind to proteins that normally recognize DNA. Some peptide-based DNA mimics^13^ and sulfated oligosaccharides such as heparin bind to DNA-binding proteins.^14,15^ Yet the main family of DNA mimic foldamers consist of single-stranded aromatic oligoamides bearing negatively charged side chains at positions that match the positions of phosphates in B-DNA. In contrast with other DNA mimics that possess base-pairing abilities and target nucleic acids, *e.g.* peptide nucleic acids (PNAs),^16^ locked nucleic acids (LNAs)^17^ and other xenonucleic acids (XNAs),^18,19^ DNA mimic foldamers are specifically designed to directly compete with DNA for binding to proteins.^20–24^

**Fig. 1:**
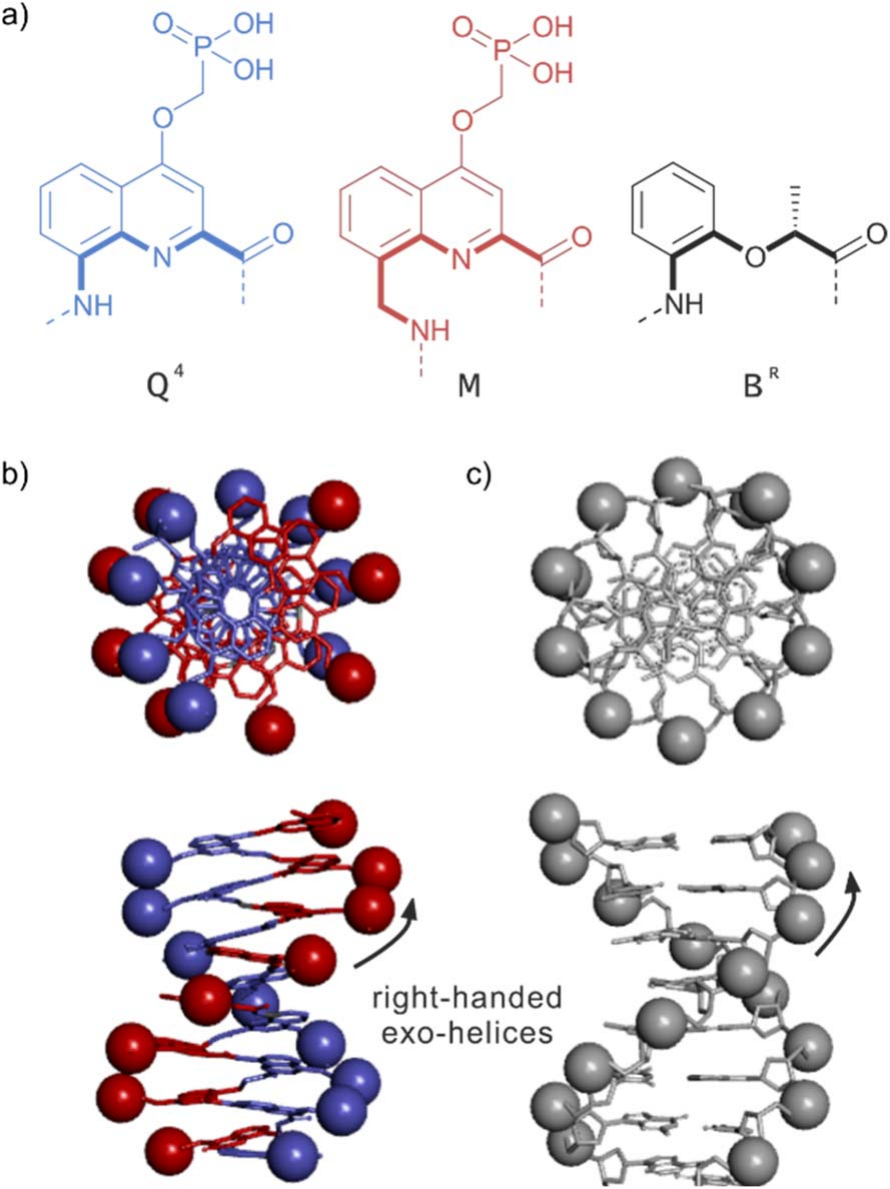
(a) Structures of Q^4^, M and B^*R*^ amino acid monomers. Top views and side views of molecular models of (MQ^4^)_8_ (b) and of an eight-base-pair B-DNA duplex (c). Models are shown at the same scale as stick representations, except for phosphorus atoms, which are shown as spheres. Monomers are color-coded in red and blue as in (a).

The DNA mimic foldamer parent series consists of alternating 8-aminomethyl-2-quinolinecarboxylic acid monomer M and 8-amino-2-quinolinecarboxylic acid monomer Q^4^, both bearing a negatively charged side chain (Fig. 1a).^20^ Like many other aromatic foldamers,^25^ these sequences adopt stable helical conformations stabilized by intramolecular hydrogen bonds, electrostatic repulsions and aromatic stacking. Since M and Q^4^ lack stereogenic centers, (MQ^4^)_*n*_ helices exist as a racemic mixture of right-handed (*P*) and left-handed (*M*) conformers. Upon introducing a chiral B^*R*^ residue, handedness can be quantitatively biased to an *M* main chain helix that displays two *P* exo-helices of negatively charged side chains matching the negative charge distribution of B-DNA (Fig. 1b and c).^26,27^ These structural attributes, along with the construction of palindromic foldamer sequences,^27^ enable DNA mimic foldamers to outcompete DNA in binding some DNA-binding proteins. For example, binding and inhibition have been demonstrated for topoisomerase 1 and HIV integrase,^20,22^ for bacterial chromosomal protein Sac7d,^21^ and for the Origin Recognition Complex (ORC).^24^ In addition, (MQ^4^)_*n*_ helices have been shown to impact chromatin composition *in vitro* and *in vivo* and disrupt cell cycle progression.^24^

DNA mimic foldamer targets have until now been limited to proteins that recognize B-DNA through its overall shape and do not include proteins specific to a particular DNA sequence. Among shape selective-proteins, not all are efficiently bound by the DNA mimic foldamers. A certain degree of selectivity is thus observed.^20,22^ Nevertheless, the large family of proteins that selectively bind to specific DNA sequences, *e.g.* transcription factors and restriction enzymes, may not be targeted with simple (MQ^4^)_*n*_ oligomers. Indeed, sequence-selective DNA-binding proteins typically interact with the grooves of B-DNA, where parts of the nucleobases are exposed.^1,28^ In contrast, an (MQ^4^)_*n*_ helix has a constant repeat motif and may be compared to a poly(dA:dT) DNA duplex deprived of sequence information.

To address this challenge and advance the next generation of DNA mimic foldamers, we have developed chimeric molecules that combine foldamer and DNA segments and that have shown promise in targeting protein–DNA interactions involving sequence-selective binding.^20,29^ Here, we introduce the first step of an alternate approach aimed at tailoring the major groove of the DNA mimics itself so that it contains sequence information through modifications of the foldamer main chain. We report the design and synthesis of novel monomers that are structural analogues of M and Q^4^ units. Through solid-state and solution investigations, we validate that these monomers are compatible with the (MQ^4^)_*n*_ helical architecture while modifying groove shape, exposing different functionalities in the major groove and allowing the modulation of the overall flexibility of the helix. The combination of the new units with M and Q^4^ monomers results in aperiodicity in the composition of the main chain, which is unusual in both biopolymers and synthetic foldamers. We show that some of these main chain modifications alter the binding of DNA mimics foldamers to bacterial histone-like protein HU. It is thus hoped that sequence control in DNA mimic foldamers will enable the selective recognition of specific protein components such as the α-helices used by many transcription factors to read base pair sequences in the major groove of DNA.

## Results and discussion

### Monomer design and synthesis

**Fig. 2:**
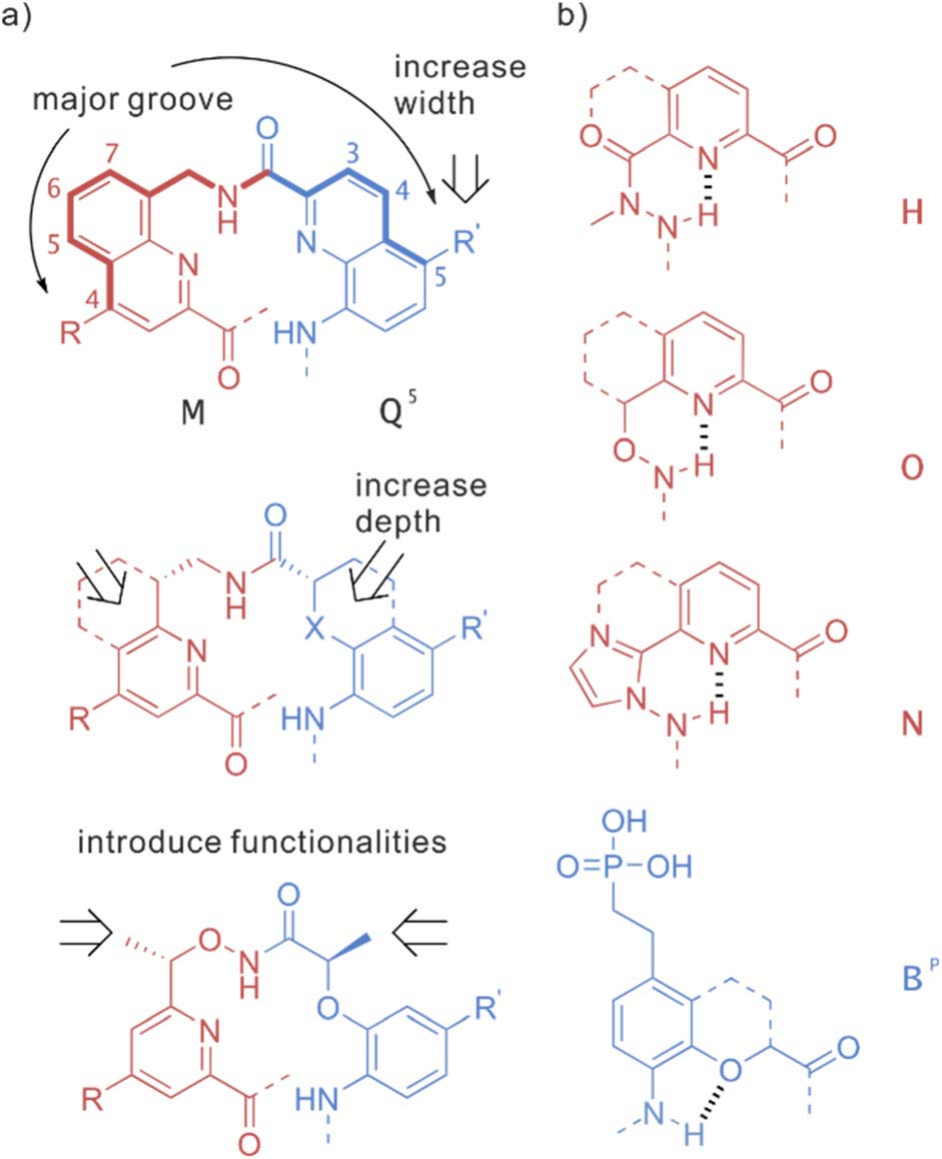
(a) Q^5^M dimer with the rim exposed in the major groove shown in bold lines. The side chain position in Q^5^ differs from that in Q^4^ (Fig. 1a). (b) M analogues H, O and N (in red), and Q analogue B^*P*^ (in blue). Hydrogen bonds are indicated as black hashed lines. Dashed lines indicate the parts of the aromatic rings of M and Q that have been removed.

The original (MQ^4^)_*n*_ helix has shallow grooves in which each MQ^4^ dimer – the equivalent of a base pair in B-DNA – displays the same functionalities. This structural homogeneity makes it challenging for these foldamers to engage in sequence-selective binding with DNA-binding proteins. To overcome this limitation, we proposed tailoring of the major groove of DNA mimic foldamers, as it is the primary site of sequence-specific interactions for the majority of DNA-binding proteins. In (MQ)_*n*_ helices, the major groove can be defined as the area starting from the side chain of Q to the side chain of M (Fig. 2a). The functions exposed in the groove include part of the pyridine ring of Q (positions 3 and 4) as well as its carbonyl group and the main chain methylene of M as well as part of its benzene ring (positions 5, 6, and 7).

Our approach focused on three key objectives (Fig. 2a): (1) widening the major groove by repositioning the side chain of Q units from position 4, as in Q^4^ in Fig. 1a, to position 5, as in Q^5^ in Fig. 2a; (2) deepening the major groove by trimming the pyridine ring of Q or the benzene ring of M; and (3) introducing new functionalities to provide additional interaction sites. To this end, we designed and synthesized H, O and N, three pyridine-based ε-amino acid analogues of monomer M, and B^*P*^, an aniline-based δ-amino acid analogue of monomer Q (Fig. 2b). The new features of these monomers include hydrogen-bond donors/acceptors and the methylene groups of O and B^*P*^ where other functionalities could be installed, including a stereogenic center. Energy-minimized molecular models of DNA mimic foldamer helices including each of these new monomers were generated, highlighting the subtle variations in the shape and polarity of the major groove that can be expected where the monomers were introduced (Fig. S1).

**Fig. 3:**
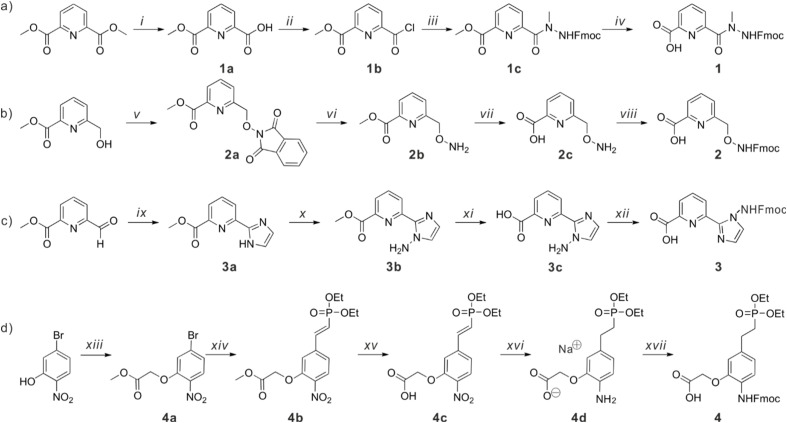
(a) Synthesis of Fmoc-H-OH (1): (i) KOH, MeOH, 0 °C, 3 h, 67%; (ii) oxalyl chloride, DMF, r.t., 3 h, quant.; (iii) Fmoc-methylhydrazine, triethylamine, dichloromethane, r.t., 2 h, 57%; (iv) LiI, EtOAc, overnight, 77%. (b) Synthesis of Fmoc-O-OH (2): (v) *N*-hydroxyphthalimide, diisopropyl-diazodicarboxylate, PPh_3_, toluene, r.t., 3 h, 95%; (vi) N_2_H_4_·H_2_O, MeOH, r.t., 30 min, quant.; (vii) KOH, MeOH/H_2_O, 0 °C, 1 h, quant.; (viii) Fmoc-*O*Su, NaHCO_3_, dioxane/H_2_O, 0 °C to r.t., overnight, 84%. (c) Synthesis of Fmoc-N-OH (3): (ix) NH_4_OAc, glyoxal, MeOH, 50 °C, 2 h, 62%; (x) lithium bis(trimethylsilyl)amide, *O*-diphenylphosphinyl hydroxylamine, THF/DMF, 0 °C, 2 h, 80%; (xi) LiOH, THF/H_2_O, 0 °C, 30 min, quant.; (xii) Fmoc-*O*Su, NaHCO_3_, dioxane/H_2_O, 0 °C to r.t., overnight, 68%. (d) Synthesis of Fmoc-B^*P*^-OH (4): (xiii) methyl bromoacetate, K_2_CO_3_, acetone, 2 h, 96%; (xiv) diethyl vinylphosphonate, Pd(PPh_3_)_2_Cl_2_, K_2_CO_3_, *o*-xylene, 125 °C, 2 h, 84%; (xv) LiOH, THF/H_2_O, 0 °C, 30 min, quant.; (xvi) H_2_, Pd/C, Na_2_CO_3_, THF, quant.; (xvii) Fmoc-Cl, NaHCO_3_, dioxane/H_2_O, 0 °C to r.t., overnight, 80%.

Amino acid H was designed with a methyl-substituted hydrazide group: pyridyl-C(=O)N(Me)NH. The methyl group was intended to promote the formation of a six-membered hydrogen-bonded ring involving the adjacent NH proton and the pyridine endocyclic nitrogen atom (Fig. 2b), as with M. In the absence of the methyl group, the *trans* conformation of the hydrazide C(=O)–NH bond would instead favor a five-membered hydrogen-bonded ring and the contribution to helix shape would differ from that of M. The H monomer lacks carbon atoms 6 and 7 of the benzene ring of M, which results in a deeper major groove. Instead of the carbon 5 of M, also in the major groove, H has a carbonyl oxygen atom, *i.e.* a hydrogen bond acceptor. Monomer H was synthesized in four steps starting from commercially available dimethyl pyridine-2,6-dicarboxylate (Fig. 3a) using described procedures for its mono-saponification into 1a and subsequent activation into mono acid chloride 1b.^30^ Coupling with Fmoc-methylhydrazine afforded compound 1c. Demethylation of 1c with lithium iodide provided 1, the Fmoc-protected form of H, in an overall 38% yield over four steps without requiring any chromatographic purification. This route can in principle be applied to variants of H bearing a substituent other than methyl on the hydrazide or a side chain, *e.g.* a phosphonate, on the pyridine ring using chelidamic acid as a starting material, but this has not been tested yet.

Monomer O was designed by trimming M to an even greater extent than H (Fig. 2b). Two sp^3^ centers, a methylene group and the oxygen atom of a hydroxylamine, are exposed in the major groove. The reduced number of sp^2^ centers in the main chain may allow for easier bond rotation, that is, enhanced flexibility. The synthesis of O was carried out in four steps from commercially available methyl 6-(hydroxymethyl)picolinate (Fig. 3b). An *N*-hydroxy-phthalimide group was introduced *via* a Mitsunobu reaction in 95% yield. Deprotection of the amine with hydrazine hydrate, followed by methyl ester saponification, yielded amino acid 2c, which was not purified and used directly in the final Fmoc installation step. Again, these relatively mild transformations are presumed to be compatible with a range of substituents on the pyridine ring.

Amino acid N can be viewed as an analogue of H in which the rotation about the C(=O)–NMe bond is locked within an imidazole ring (Fig. 2b). N has the same number of rotatable bonds as M and no sp^3^ center, which we expect to contribute to rigidity. N was prepared from methyl 6-formylpicolinate in four steps. The imidazole synthesis was carried out with NH_4_OAc and glyoxal,^31^ followed by electrophilic N-amination, methyl ester saponification and Fmoc installation (Fig. 3c).

Finally, amino acid B^*P*^ carrying a phosphonic acid side chain was conceived as an analogue of Q^5^, that is, with a wider major groove (Fig. 2b). In addition, the carbon atoms in positions 3 and 4 of the quinoline have been removed to increase groove depth as well. B^*P*^ was synthesized in its diethylphosphonate and Fmoc protected form in five steps using protocols similar to those developed for other B monomers without side chains or with other side chains (Fig. 3d).^32^ Noteworthy is the hydrogenation of the nitro group of 4c, which is performed on the sodium carboxylate, and not the carboxylic acid or the methyl ester, to avoid lactam formation. Analogues of B^*P*^ could be conceived in which the main chain methylene group would bear a substituent, thus forming a stereogenic center, as in B^*R*^ (Fig. 1a).

### Helix folding with H, O and N monomers

**Fig. 4:**
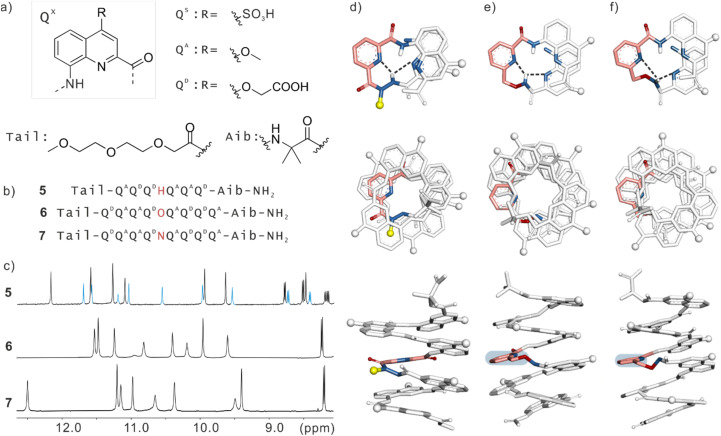
(a) Structures of Q^*X*^ and of N- and C-terminal groups Tail and Aib. (b) Aromatic foldamer sequences 5–7. (c) Excerpts of the ^1^H NMR spectra of sequences 5–7 (500 MHz, 50 mM NH_4_HCO_3_, pH 8.5, H_2_O/D_2_O 9 : 1 v/v, at 25 °C). A minor set of signals in the spectrum of 5 are highlighted in blue. (d) Crystal structure of sequence 5. Top and side views show the conformation of the H monomer. The *N*-methyl group carbon atom is shown as a yellow sphere. (e and f) Two independent molecules in the crystal structure of 6. Top and side views show different conformations of the O monomer. Atoms in the plane of the pyridine ring of O are highlighted in a blue box. The first atom of each side chain is shown as a gray sphere to indicate its position. The rest of the side chains and most hydrogen atoms are omitted for clarity. Hydrogen bonds are indicated by black dashed lines.

In order to investigate the conformations of these new building blocks both in solution and in the solid state, oligoamides 5–7 were first synthesized using previously reported solid-phase synthesis (SPS) methods (Fig. 4a and b).^33^ All consist of an H, O or N monomer flanked on both sides by three or four Q units, which span over a helix turn. The sequences include a combination of acidic Q^*D*^ and neutral Q^*A*^ monomers to ensure solubility in aqueous media, but not to an extent that may hamper crystal growth. The positions of the neutral and acidic residues were strategically chosen on different sides of the helix.^34^ In addition, an Aib group (2-aminoisobutyric acid) was included at the C-terminus to prevent head-to-head stacking of the helices,^35^ and a short diethylene glycol tail was added to the N-terminus for similar reasons.^34^ The ^1^H NMR spectra of these compounds in H_2_O/D_2_O showed a sharp set of signals distributed over a wide range of chemical shift values, which is characteristic of helical folding (Fig. 4c).^36^ Sequences 6 and 7 exhibited a single set of signals, indicative of a single, stable conformer.^37^ In contrast, sequence 5 shows two distinct sets of signals, suggesting the presence of two species.

Single crystals suitable for X-ray diffraction analysis were obtained for 5 and 6 (not for 7), and the structures in the solid state of these two oligomers could be elucidated. In both cases, the structures contained four molecules in the asymmetric unit, which represented a challenge in terms of refinement but also provided valuable information about the conformations of the new monomers. Both structures confirmed helical folding and new monomer conformations that match the original designs (Fig. 4d and e). The sequences are achiral, and equal numbers of *P* and *M* helices were found in the lattices.

In the case of 6, the four independent helices have similar overall shapes, but the conformations at the O units vary. In one helix, the main chain sp^3^ oxygen atom is found in the plane of the pyridine ring, meaning that there is almost no twist (7°) of the pyridine-methylene bond (Fig. 4e). In the other helices, the sp^3^ oxygen atom is out of the plane of the pyridine ring – it is instead close to the plane of the preceding quinoline unit – due to torsions of up to 77° of the pyridine–methylene bonds (Fig. 4f). The N–O bonds are all twisted but take quite variable angle values, from 47° to 115°. Altogether, this points to an enhanced flexibility of monomer O, as predicted at the design step.

In the case of 5, the four independent molecules all have very similar conformations. As anticipated in the initial design, the H monomer's methyl group points away from the helix core, and its hydrazide proton forms a hydrogen bond with the endocyclic nitrogen atoms of the adjacent pyridine and quinoline rings (Fig. 4d). The pyridine–hydrazide bond is twisted (from 31 to 37°), which is not possible in M monomers. The N–N bond is *gauche* (MeN–NH torsion angles from 118 to 124°). This *gauche* conformation of the acylhydrazide (O=C–NR–NR–C=O) is one of the common forms.^38^ It was also observed in the crystal structure of 1 and of a related QH dimer, where the N–N twist angle was close to 90° (Fig. S2). Completely flat *anti* conformations of acyl hydrazides have also been observed.^39^ In any case, it is reasonable to assign the solid state structure of 5 to one of the two sets of signals observed in the NMR spectra.

**Fig. 5:**
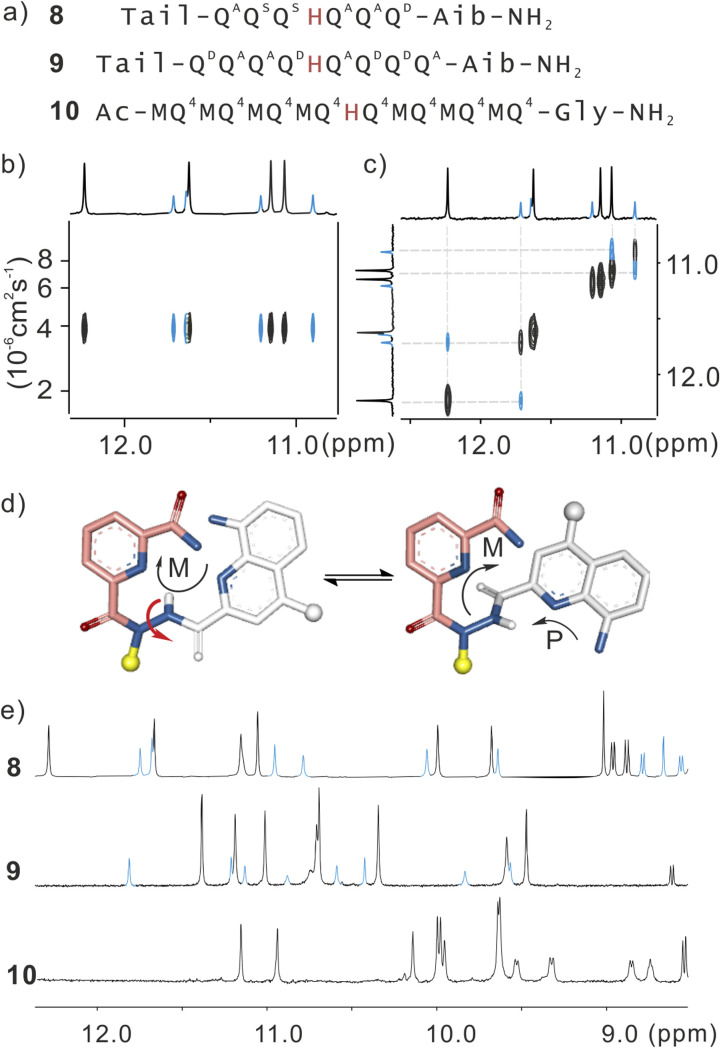
(a) A summary of aromatic amide foldamer sequences 8–10. Excerpt from the ^1^H DOSY NMR spectrum (b) and 2D ROESY NMR spectrum (c) of sequence 8 in which two different species coexist (500 MHz, H_2_O/CD_3_CN 3 : 1 v/v, at 25 °C). (d) Parts of two proposed conformations for sequence 8, with the H monomer shown in pink and the methyl group represented as a yellow sphere. (e) Excerpts of the ^1^H NMR spectra of sequences 8–10 (500 MHz, 50 mM NH_4_HCO_3_, pH 8.5, H_2_O/D_2_O 9 : 1 v/v, at 25 °C). Signals that correspond to minor species are highlighted in blue, although this does not reflect the original phase in the recorded ROESY spectrum.

Additional sequences 8–10 (Fig. 5a) were subsequently prepared to investigate what the other species present in the NMR spectra of sequence 5 may be. The moderate solubility of sequence 5 made it difficult to perform some 2D NMR experiments. These were instead performed using analogous sequence 8, which contains two Q^*S*^ units instead of Q^*D*^, resulting in better solubility. The ^1^H NMR spectrum of 8 also showed two sets of signals (Fig. 5e). The relative intensities between the two sets of signals varied upon changing temperature and solvent (% of CD_3_CN), demonstrating that the corresponding species interconvert (Fig. S3 and S4). Diffusion-ordered spectroscopy (DOSY) revealed that two species possess comparable diffusion coefficients, hinting at two conformations rather than at some aggregation (Fig. 5b and S5). In agreement with this interpretation, the proportions are not concentration dependent (not shown). Rotating-frame Overhauser effect correlation spectroscopy (ROESY) also indicated exchange between the two species (Fig. 5c and S6). In addition, nuclear Overhauser effect spectroscopy (NOESY) spectra were recorded. The ROESY spectrum allowed us to tell for each correlation whether transfer of nuclear spin polarization was due to exchange between the two species or due to a short distance between the corresponding protons (NOE), due to different signs of the cross peaks – regardless of the size of the molecule. However, ROESY suffers from sensitivity with larger molecules and also from TOCSY artifacts. Both disadvantages would hinder the required assignment for the complete subsequent identification of the protons and are absent in NOESY spectra. For the major species, the first (N-terminal) amide N*H* was identified through an NOE correlation with the neighboring diastereotopic C*H*_2_. NOEs between consecutive N*H*s in the sequence then allowed for their complete assignment (Fig. S7). Thus, the N*H* resonance at 8.5 ppm was assigned to the H monomer's amide, with the corresponding resonance of the minor species shifted downfield to 10.6 ppm, a difference of 2.1 ppm (Fig. S6 and S7). The signal of the H monomer's methyl group was also shifted upfield in the major species (Δ*δ* = 0.61 ppm). Another remarkable feature was the signal of an unassigned aromatic C*H* doublet, found to be very different in the major species (7.0 ppm) and in the minor species (8.8 ppm) (Fig. S6). Altogether, these results point to distinct conformations at the H unit. Key information is the existence of a strong NOE correlation between the hydrazide N*H* and NC*H*_3_ protons in the major species. This correlation is hardly compatible with the original design (Fig. 2b) and with the conformation of 5 in the solid state (Fig. 3d), where the H⋯H distance is larger than 3.2 Å. Instead, we propose an alternative conformation in which the N–N bond adopts the other *gauche* conformation (NH–NMe torsion of *ca.* 60° instead of *ca.* 120°) with a corresponding H⋯H distance of 2.5 Å (Fig. S8). In this conformation, intramolecular aromatic stacking was extensive when the two helix segments before and after the H unit had opposite handedness, the H unit thus promoting a local reversal of helix sense. This behavior contrasts with that of other monomers for which helix reversal also entails reduced intramolecular stacking.^37,40^

The extended sequence 9 also displays two sets of ^1^H NMR signals (Fig. 5e). The proportion of the minor species was reduced in comparison with 8, showing that the conformational behavior of H depends on sequence length. Sequence 10 is a DNA mimic (MQ^4^)_*n*_ foldamer in which one M unit has been replaced by H. Unlike other H-containing sequences, the NMR spectrum of 10 shows a single set of NMR signals suggesting that it exists predominantly as a single conformer (Fig. 5e). The conformational behavior of H therefore also depends on the composition of the sequence (only Q in 8 and 9*vs.* alternation of M and Q in 10). We made no attempt to assign the major species of 9 and 10 to one or the other conformer because it became clear at that stage that the H monomer complicates helix shape design and may not reliably be incorporated in foldamer helices without disturbing them.

### DNA mimic helices with B^*P*^ monomers

**Fig. 6:**
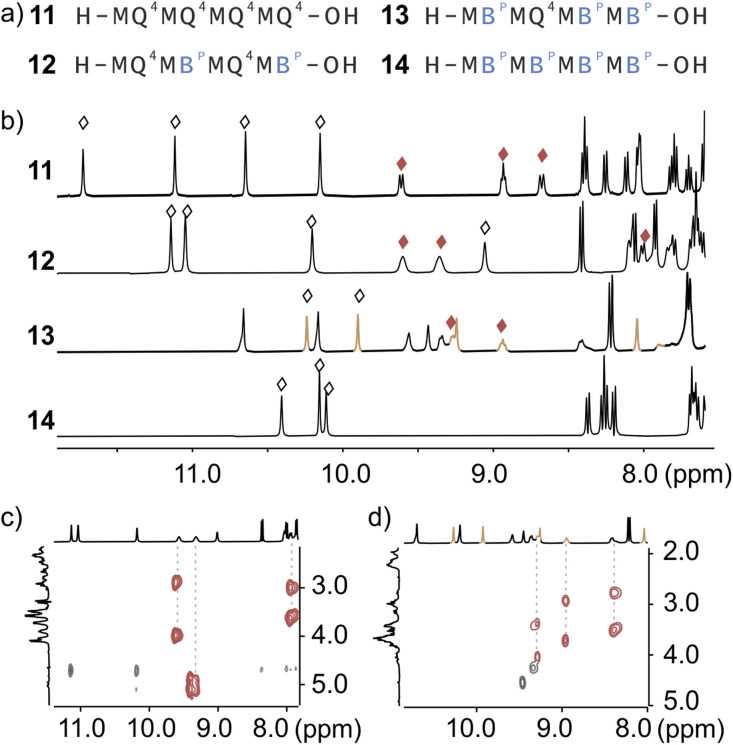
(a) Aromatic amide foldamer sequences 11–14. (b) Excerpts of the ^1^H NMR spectra of sequences 11–14 (500 MHz, 50 mM NH_4_HCO_3_, pH 8.5, H_2_O/D_2_O 9 : 1 v/v, at 25 °C). Signals assigned to aromatic N*H*s and aliphatic N*H*s are indicated by black diamonds and red diamonds, respectively. Excerpt of the ^1^H–^1^H TOCSY spectrum of sequence 12 (c) and 13 (d) showing the correlations between aliphatic N*H*s and diastereomeric pairs of C*H*_2_ protons. Signals that correspond to two different species are highlighted in black and orange, respectively.

Monomers such as B with different side chains or stereogenic centers (*e.g.* B^*R*^) have already been reported, and their conformations in Q_*n*_ helices have been validated, including in the solid state.^26,32^ Here, we assessed the behavior of B^*P*^ with a phosphonic side chain in the specific context of DNA mimic (MQ^4^)_*n*_ sequences. We prepared octaamides 12–14 as analogues of reference sequence (MQ^4^)_4_11 in which two, three, or four Q^4^ units have been replaced by B^*P*^ (Fig. 6a). The oligomers were synthesized using low-loading Wang resin according to the SPS protocols mentioned above.^33^ The final products were subsequently purified *via* RP-HPLC under acidic conditions for diethyl-phosphonate protected precursors and under basic conditions for deprotected phosphonate products, achieving high purity (>99%) and good overall isolated yields (16–20%).

The ^1^H NMR spectra of sequences 11–14 all display sharp amide and aromatic proton signals distributed over a wide range of chemical shift values (Fig. 6b). This pattern is consistent with previous studies on aromatic oligoamides and suggests helical conformations.^36^ To further confirm helical folding in water, we probed the anisochronicity of the main chain NH–C*H*_2_-aryl benzylic protons of M units. These protons are diastereotopic in helical conformations and may appear at distinct chemical shift values when *P* and *M* helices are under slow exchange on the NMR timescale. For both 12 and 13, total correlation spectroscopy (TOCSY) spectra showed distinct signals for these protons in the M monomers with Δ*δ* values between 0.7 and 0.9 ppm (Fig. 6c and d). Additionally, NOE correlations between neighboring NHs further supported canonical helical folding of these two oligomers (Fig. S9 and S10). In contrast, the aliphatic amide N*H* resonances of 14 cannot be seen in its ^1^H NMR spectrum and multiple aliphatic resonances are also missing, probably due to extreme broadening. Although this does not exclude helix folding, it suggests that some dynamics are at play that are neither fast nor slow on the NMR timescale, as was observed in other B-containing sequences.^32^ Nevertheless, cooling down to 5 °C did not significantly change the spectra (Fig. S11).

**Fig. 7:**
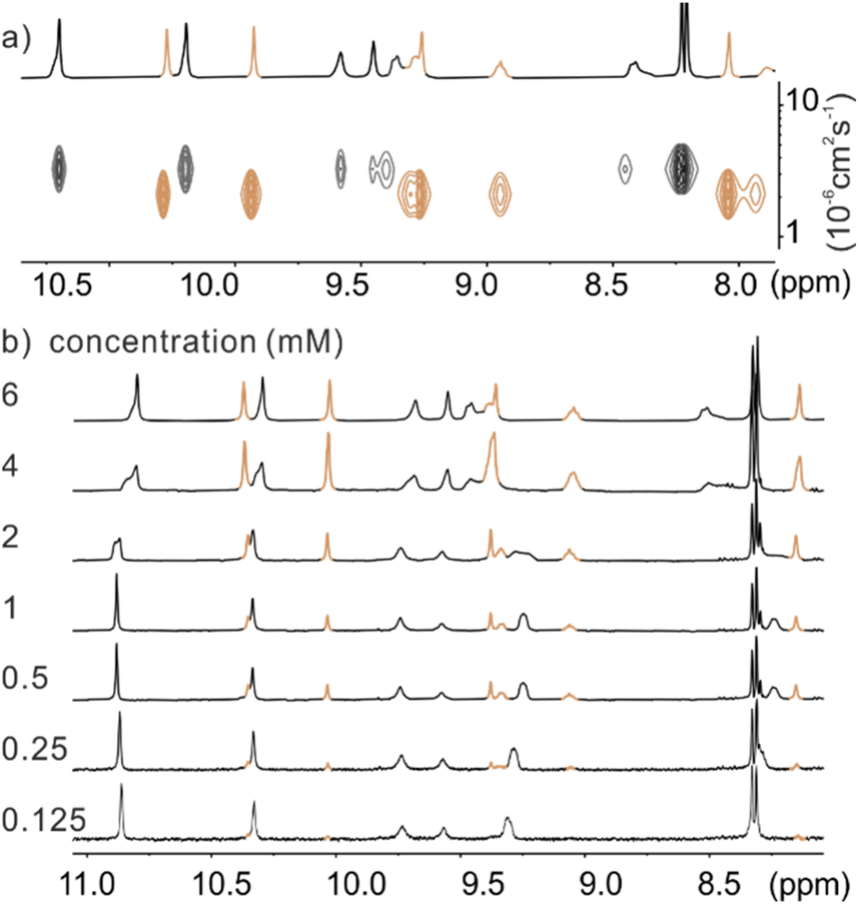
(a) ^1^H DOSY NMR spectrum of sequence 13. (b) Excerpts of the ^1^H NMR spectra of 13 at different concentrations. (500 MHz, 50 mM NH_4_HCO_3_, pH 8.5, H_2_O/D_2_O 9 : 1 v/v). Signals that correspond to two different species are highlighted in black and orange, respectively.

In this series, 13 appeared to be an outlier, as its ^1^H NMR spectrum showed two sets of signals at higher concentrations (Fig. 7 and S10). The concentration dependence of the proportion of the two species as well as DOSY indicates some sort of aggregation into a discrete and well-defined species. The fact that this behavior was not observed with 12 and 14, which have the same two C-terminal residues as 13, excludes aggregation *via* the C-terminal cross-section (the reason why a C-terminal Aib was included in 5–8).^35^ Aggregation of helical aromatic foldamers into multistranded helices, including in water, has been observed before,^41,42^ but not within segments that contain Q or M. It may well be that a new aggregation mode is at play here, which would be of particular interest for DNA mimics, but structural investigations have not yet been attempted.

Altogether, these results demonstrate that the B^*P*^ monomer does not hamper helix folding when combined with M monomers in the context of DNA mimic foldamers and that several B^*P*^ monomers may lead to an unidentified aggregation mode. B^*P*^ contains more rotatable bonds than Q, and it possesses a smaller aromatic surface, which limits hydrophobic effects associated with aromatic stacking in the helix. (MB^*P*^)_*n*_ helices are thus expected to undergo faster conformational dynamics than (MQ)_*n*_ helices, for example, faster helix handedness inversion, as do (QB)_*n*_ helices.^32^

### Quantitative assessment of conformational dynamics

The results above point to a high level of compatibility of the new monomers with the helical folding of DNA mimic (MQ^4^)_*n*_ sequences, with the exclusion of H, which may promote alternate conformations. They also point to enhanced conformational dynamics when some of these monomers are introduced due to their reduced aromatic surface and larger number of rotatable bonds. We set out to quantitatively assess DNA mimic helix stability in the presence of H, O, N and B^*P*^ monomers using a recently developed assay.^43^ In short, this assay consists of introducing a chiral B^*R*^ unit that quantitatively biases handedness to the *M* helix in aqueous media but only partially in the presence of polar organic solvents. We allowed chiral foldamer solutions to first equilibrate in a 9 : 1 v/v mixture of DMSO and 15 mM aqueous NH_4_OAc at 70 °C. The samples were freeze-dried to obtain a solid containing both *M* and *P* diastereomeric conformers. These solids were then dissolved in water and the buildup of a circular dichroism (CD) band was monitored as a function of time as the effect of the B^*R*^ unit led to complete handedness bias.

**Fig. 8:**
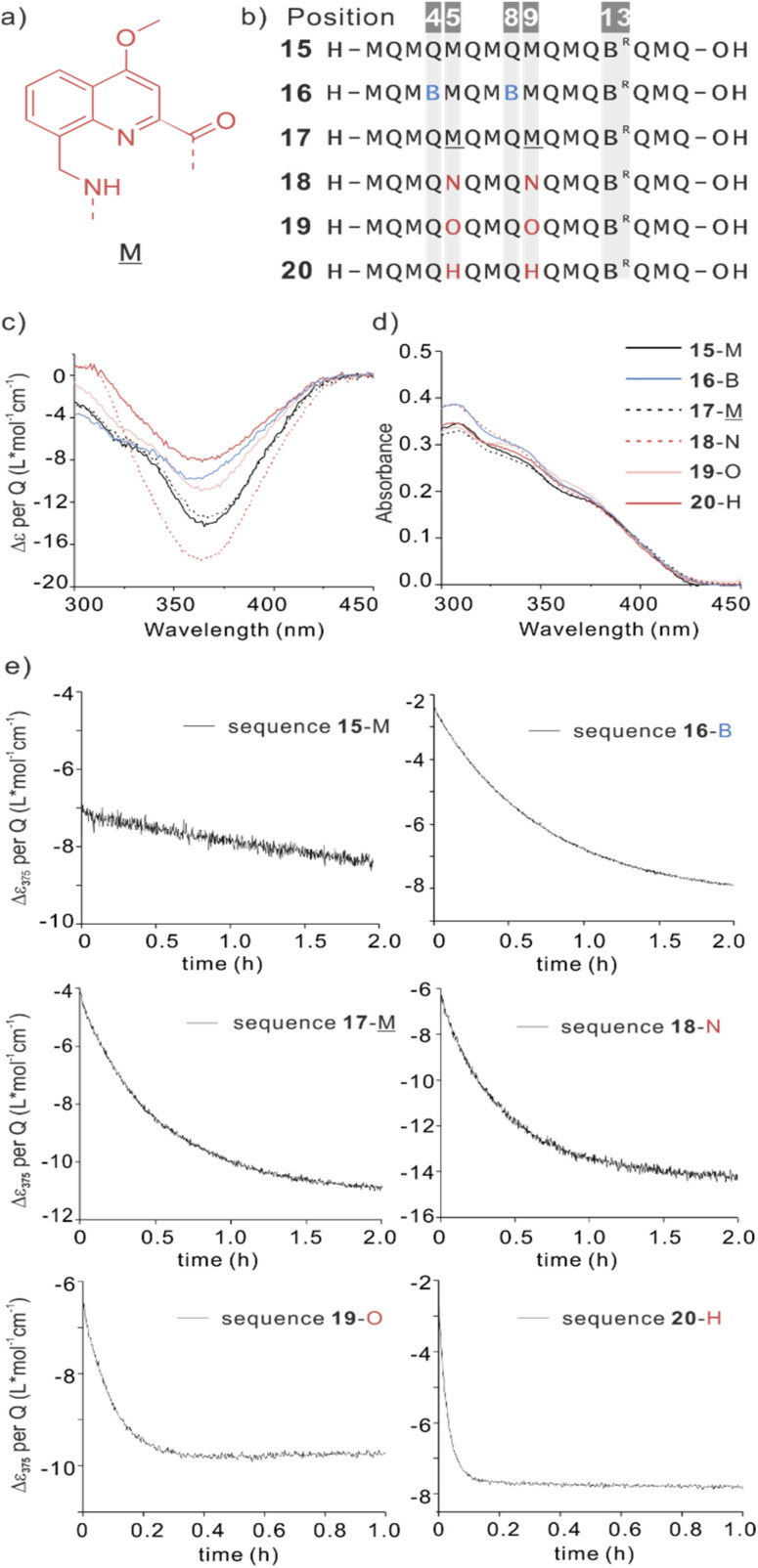
(a) Structure of monomer M̲. (b) Foldamer sequences 15–20. (c) CD spectra of 15–20 (40 µM, 50 mM NH_4_HCO_3_, pH 8.5, at 25 °C). Molar extinction (Δ*ε*) is normalized for the number of quinoline rings. (d) UV absorption spectra of sequences 15–20 (40 µM, 50 mM NH_4_HCO_3_, pH = 8.5 at 25 °C). (e) Monitoring (375 nm) of *M* helix enrichment after dissolving 15–20 (40 µM) in 50 mM aqueous NH_4_OAc (pH 5.5) at 25 °C. For clarity, the letter of a remarkable monomer that they contain is indicated after the compound number.

For these experiments, a new series of sequences were designed and synthesized (15–20 in Fig. 8a and b), all containing a chiral B^*R*^ unit at the same position. Sequences 15 and 17 served as references and the other sequences included two B^*P*^ units instead of Q^4^ units, or two H, O, or N units instead of M units. Synthesis was performed on an automated synthesizer, and the sequences were obtained in good yields. The NMR spectra of 15–19 in H_2_O/D_2_O 9 : 1 v/v showed a single set of signals (Fig. S12), indicating both helix folding and quantitative helix handedness bias. In contrast, sequence 20 exhibited at least one minor set of signals, consistent with the behavior of other H-containing sequences. The CD spectra of 15–20 in aqueous medium all showed a negative band near 360 nm characteristic of a prevalent *M* helix handedness. The CD band intensity varied despite the UV absorbance at the same wavelength being the same (Fig. 8c and d). This tells that CD band intensity is sequence dependent and not a reliable tool to quantitatively assess handedness bias, which NMR measurements already showed to be quantitative in 15–19. We nevertheless note that 20 has the weakest CD band, consistent with possible helix handedness reversals at H units, as suggested above. The CD spectra were also measured in DMSO/water to validate that handedness bias is weaker in this solvent (Fig. S13).

The kinetic curves of CD band buildup in water at pH 5.5 are shown in Fig. 8e. These curves were fitted to single exponential decays (Fig. S14), and the corresponding kinetic parameters are reported in Table 1. At pH 5.5, the phosphonic acid side chains are mostly monoanionic.^20^ The helix handedness dynamics of reference sequence 15 were so slow that no kinetic parameters were extracted. In contrast, a half-life of helix handedness inversion of 30 min was measured for sequence 16. The two B^*P*^ units of 16 thus enhance its conformational dynamics with respect to 15. The half-life of 30 min is nevertheless longer than those of sequences 17–18 and points to considerable conformational stability at this pH.

**Table 1:** Half-lives (*t*_1/2_) of helix handedness inversion of sequences 15–20 at different pH at 25 °C

Sequence	15 (w/M)	16 (w/B^*P*^)	17 (w/M̲)	18 (w/N)	19 (w/O)	20 (w/H)
pH 5.5	—a	30 min	22 min	18 min	4.2 min	1.2 min
pH 8.5	54 min	4.2 min	10 min	9.0 min	2.4 min	—b

a Kinetics were too slow to monitor at 25 °C. A value of 207 min has been reported at 45 °C for this compound.^43^

b Kinetics were too fast to monitor at 25 °C.

Sequence 17 is an analogue of 15 in which two negatively charged side chains have been replaced by neutral methoxy groups in M̲ units (Fig. 8a). Sequence 17 was designed to serve as a reference for 18–20, whose H, O and N units also do not contain a negatively charged side chain. The helix handedness dynamics of 17 were much faster than those of 15 and even faster than those of 16 (*t*_1/2_ of 30 min). This result shows, somewhat unexpectedly, that the phosphonic acid side chains have a stabilizing effect. This effect has already been discussed elsewhere.^43^ The kinetics of helix handedness inversion of 18 only marginally differed from those of 17 (*t*_1/2_ of 22 min). M̲ monomers may therefore be replaced by N monomers without significantly altering helix stability. This result is consistent with the design of N as an analogue of M that contains two aromatic rings and few rotatable bonds. It points to this design as a robust alternative even though it is the only monomer for which solid state structural evidence has not yet been obtained. In contrast, and in agreement with expectations, helix handedness inversion is faster in 19 and 20, with *t*_1/2_ values of 4.2 and 1.2 min, respectively. The fastest kinetics were thus observed when monomer H was included, consistent with its ambivalent conformational behavior.

We also monitored helix handedness inversion dynamics by CD at pH 8.5 (Fig. S15). At this pH, the phosphonic acid side chains are largely dianionic.^20^ This may lead to increased electrostatic repulsions and probably explains the observed half-lives of helix handedness inversion, all of which are shorter than those observed at pH 5.5. Nevertheless, the relative order of the contribution to helix stability remained unchanged: N > O > H. The effect of pH on helix stability appeared to be stronger for 15 and 16. *t*_1/2_ values are at least four times smaller at pH 8.5 than at pH 5.5 for these two sequences, and only two times smaller for 17, 18, and 19. This larger effect may be due to the fact that 15 and 16 contain two more charged residues than the other sequences (Q^4^ or B^*P*^*vs.* M̲, N, O or H).

### Assessment of foldamer–protein interactions

**Fig. 9:**
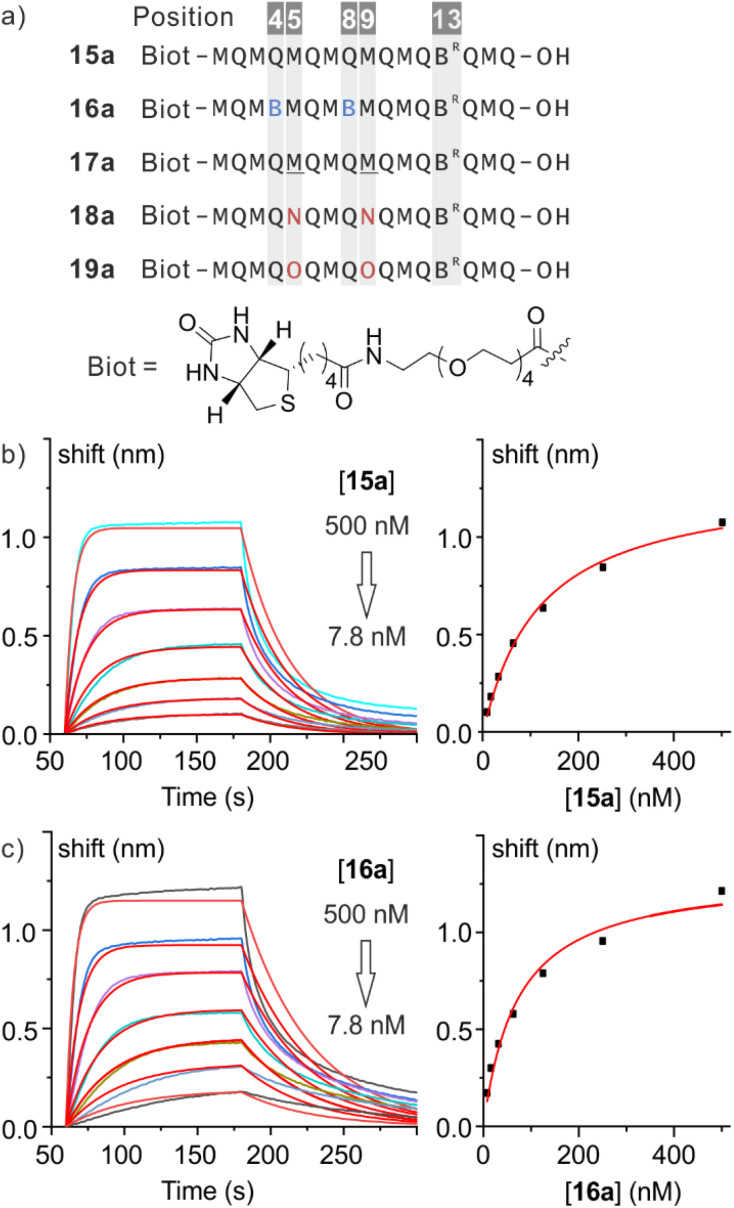
(a) Foldamer sequences 15a–19a. (b and c) BLI sensorgrams of HU protein binding to biotinylated foldamers 15a (b) and 16a (c) immobilized on streptavidin sensors. The sensorgrams were fitted to a 1 : 1 kinetic binding model (left graphs, global fitting) or to a 1 : 1 steady state binding model (right graphs). Calculated curves are shown in red.

Finally, we assessed whether the new monomers hamper the ability of DNA mimic foldamer helices to interact with a DNA-binding protein. We previously characterized the interactions of (MQ^4^)_*n*_ oligomers with histone-like bacterial protein Sac7d from hyperthermophilic archeon *Sulfolobus acidocaldarius*.^27^ This time we used another prokaryotic histone-like protein, namely HU from *Anabaena*, a 94-amino acid protein that recognizes DNA upon forming a *ca.* 20 kDa homodimer.^51^ With an HU dimer binding to 6 to 10 base pairs of double-stranded B-DNA, we expected that only one protein would bind to a 16mer DNA mimic foldamer. Compounds 15–19 were all biotinylated in DMF/water by the selective reaction of their N-terminal benzylic amine with the *N*-hydroxy-succinimide ester of a biotin derivative to yield 15a–19a, respectively (Fig. 9a). Note that 20 was not included in this series because of the complex conformational behavior of H units. Foldamer–protein interactions were assessed with biolayer interferometry (BLI). All biotinylated sequences were immobilized on streptavidin-coated BLI sensors. Sensorgrams were measured at different concentrations of HU and were fitted to the kinetic model of a 1 : 1 binding isotherm to determine the dissociation constant (*K*_D_) as well as the kinetic constant of complex formation (*k*_ass_) and dissociation (*k*_dis_). Binding kinetics were fast enough to reach a steady state in each experiment, and *K*_D_ was separately estimated using a steady-state analysis. Representative sensorgrams are shown in Fig. 9 and S16. Table 2 summarizes the data. The *K*_D_ values calculated from steady-state and kinetic fits were found to be consistent – the steady state model is fit to only seven data points and may be considered less accurate. All *K*_D_ values are in the nanomolar range, in contrast with the previously reported micromolar binding to Sac7d,^27^ but in agreement with earlier studies of the binding of HU to DNA. Binding to undistorted duplex DNA was reported to be as low as 200 nM (depending on ionic strength) but single digit nM binding was observed for distorted DNA substrates or substrates containing abasic sites.^51–53^

**Table 2:** Dissociation constants (*K*_D_) and kinetic constants of association (*k*_ass_) and dissociation (*k*_diss_) of the complexes between 15a–19a immobilized on streptavidin sensors and protein HU in aqueous Na_2_HPO_4_ (25 mM, pH 7.4), EDTA (1 mM), NaCl (250 mM), and Tween 20 (0.05%) at 25 °C

Sequence	15a (w/M)	16a (w/B^*P*^)	17a (w/M̲)	18a (w/N)	19a (w/O)
*K*_D_ (nM)^a^	100 ± 1	59 ± 1	242 ± 3	171 ± 2	194 ± 4
*k*_ass_ (10^5^ M^−1^ s^−1^)^a^	3.08 ± 0.03	3.58 ± 0.04	1.90 ± 0.02	2.32 ± 0.03	3.64 ± 0.07
*k*_dis_ (10^2^ s^−1^)^a^	3.10 ± 0.01	2.11 ± 0.01	4.60 ± 0.02	3.99 ± 0.01	7.07 ± 0.04
*K*_D_ (nM)^b^	114 ± 11	56 ± 10	195 ± 9	228 ± 20	296 ± 13

a Determined by fitting the kinetic BLI sensorgrams to a 1 : 1 kinetic model.

b Determined by fitting the BLI data to a 1 : 1 steady-state model. Standard deviations indicate the quality of the fits. Triplicate experiments showed good reproducibility (typically ± 30%).

B^*P*^-containing sequence 16a was found to be a *ca.* two-fold better binder than original sequence 15a. In contrast, introducing M monomers into 17a led to a *ca.* two-fold decrease in affinity. Replacing M̲ with N (in 18a) or O (in 19a) did not result in significant changes with respect to M̲ (in 17a). Some small variations were also observed in the kinetic constants of complex formation and dissociation. For example, the more flexible O-containing sequence 19a is the fastest at binding HU (largest *k*_ass_) and also the fastest at dissociating (largest *k*_dis_). While optimizing the conditions for the BLI experiments, we found that salt concentration and Tween 20 had notable effects. *K*_D_ values were lower at lower salt concentrations, and kinetics were slower in the absence of Tween 20, a neutral surfactant intended to reduce protein aggregation. However, the data were then less consistent with a 1 : 1 binding model and are not shown here.

Altogether these results are in agreement with the structural studies presented above showing that the new B, M̲, and O monomers do not significantly alter the overall shape of the DNA mimic foldamer helices, thus preserving their ability to recognize a DNA-binding protein. In addition, the variations in binding affinity observed upon replacing two monomers of the reference sequence for two others hint at the possibility to tailor the DNA mimic foldamers upon varying both main chain and side chain features in order to enhance their binding affinity and selectivity.

## Conclusion

In summary, we designed and synthesized four new monomers as structural analogues for the quinoline-based M and Q units in order to tailor their contribution to the features of the major groove of (MQ)_*n*_ DNA mimic foldamers. We validated the conformational behavior of these new monomers within oligoamide helices using both solid-state and solution studies. Our results show that three of the four monomers function as designed, with H exhibiting a more complicated conformation behavior. With the exception of N, the new monomers are more flexible than the original units and their incorporation leads to faster helix handedness inversion kinetics. In one case, the replacement of several Q units by B^*P*^ led to the formation of a new discrete aggregate. DNA mimic foldamers incorporating the new monomers at two positions retained their ability to bind histone-like protein HU in the nanomolar range with some variations.

The new monomers expand the toolkit required to tailor DNA mimic foldamer groove features and pave the way for foldamer sequences designed to interact selectively with defined protein targets. Our results also showcase the delicate art of controlling folded structures through changes in main chain components. Indeed, structural and functional variations in biopolymers and foldamers generally derive from defined side-chain sequences on a constant main chain repeat motif. The rational arrangement of different main chain components to produce original structures, *e.g.* in peptide homologues and analogues,^44–47^ in aromatic foldamers,^48^ and in aliphatic–aromatic hybrid sequences,^32,49,50^ represents an advanced level of design.

## Author contributions

J. W. and V. C. performed synthesis and experimental studies. J. W. performed CD kinetic experiments. TC performed protein expression and purification and BLI studies. J. W. and P. K. M. performed crystal growth. P. K. M. performed crystallographic analysis. IH supervised the research. J. W. and I. H. wrote the manuscript. All authors reviewed and edited the manuscript and approved its final version.

## Conflicts of interest

There are no conflicts to declare.

## Supplementary Material

SC-OLF-D6SC00798H-s001

SC-OLF-D6SC00798H-s002

## Data Availability

CCDC 2514117, 2514118, 2286782 and 2478322 (compound 1, compound 1d, oligomer 5, and oligomer 6, respectively) contain the supplementary crystallographic data for this paper.^54*a*–*d*^ The supporting data have been provided as part of the supplementary information (SI). Supplementary information: SI figures, detailed experimental protocols, crystallographic studies, and characterisation of new compounds. See DOI: https://doi.org/10.1039/d6sc00798h.
