# Supplementary material for: Tailoring the major groove of DNA mimic foldamers
Source: Chem Sci. 2026 Jun 9;17(32):15479–89. doi: 10.1039/d6sc00798h (PMC13322197; doi:10.1039/d6sc00798h)
Supplement: Supplementary file 1 [file SC-017-D6SC00798H-s001.pdf]

Supplementary Information (SI) for Chemical Science.
This journal is © The Royal Society of Chemistry 2026

Supporting Information for:

Tailoring the major groove of DNA mimic foldamers

Jiaojiao Wu,[a] Valentina Corvaglia,[‡a] Tulika Chakrabortty,[a] Pradeep K. Mandal,[a,b] and Ivan Huc[a*]

a. Address here. Department Pharmazie, Ludwig-Maximilians-Universität München, Butenandtstr. 5-13, München 81377, Germany. E-mail: ivan.huc@cup.lmu.de.

b. Institute of Science and Technology Austria, Am Campus 1, Klosterneuburg 3400, Austria

‡ Present affiliation: Institute for Stem-Cell Biology, Regenerative Medicine and Innovative Therapies, IRCCS Casa Sollievo della Sofferenza, San Giovanni Rotondo (Italy) & Center for Nanomedicine and Tissue Engineering (CNTE), ASST Grande Ospedale Metropolitano Niguarda, Milan, Rotondo, Italy.

Contents

1. List of Abbreviations 2
2. Supplementary Figures 3
3. Materials and Methods 16
3.1 General 16
3.2 Monomer synthesis procedures 19
3.3 Oligomer synthesis procedures 27
4. NMR spectra 38
5. HPLC chromatograms 60
6. Mass spectra 62
7. X-ray Crystallography 65
8. HU Protein expression and purification 70
9. Biolayer interferometry (BLI) 72
10. References 73

1. List of Abbreviations

Ac_2O	acetic anhydride
CD	circular dichroism
DBU	1,8-Diazabicyclo(5.4.0)undec-7-ene
DCM	dichloromethane
DIAD	diisopropyl azodicarboxylate
DIPEA	*N*,*N*-Diisopropylethylamine
DMF	*N,N*-dimethylformamide
DMSO	dimethyl sulfoxide
ESI	electrospray ionization
EtOAc	ethyl acetate
Et_2O	diethyl ether
Fmoc-Cl	fluorenylmethyloxycarbonyl chloride
HPLC	high performance liquid chromatography
HRMS	high resolution mass spectrometry
LC-MS	liquid chromatography-mass spectrometry
MeOH	methanol
NMR	nuclear magnetic resonance
RP	reversed phase
PyBOP	benzotriazol-1-yloxytripyrrolidinophosphonium hexafluorophosphate
r.t.	room temperature
min	minutes
SPS	solid phase synthesis
TEAA	triethylammonium acetate
TFA	trifluoroacetic acid
THF	tetrahydrofuran
TMSBr	trimethylbromosilane
UV	ultraviolet

2. Supplementary Figures

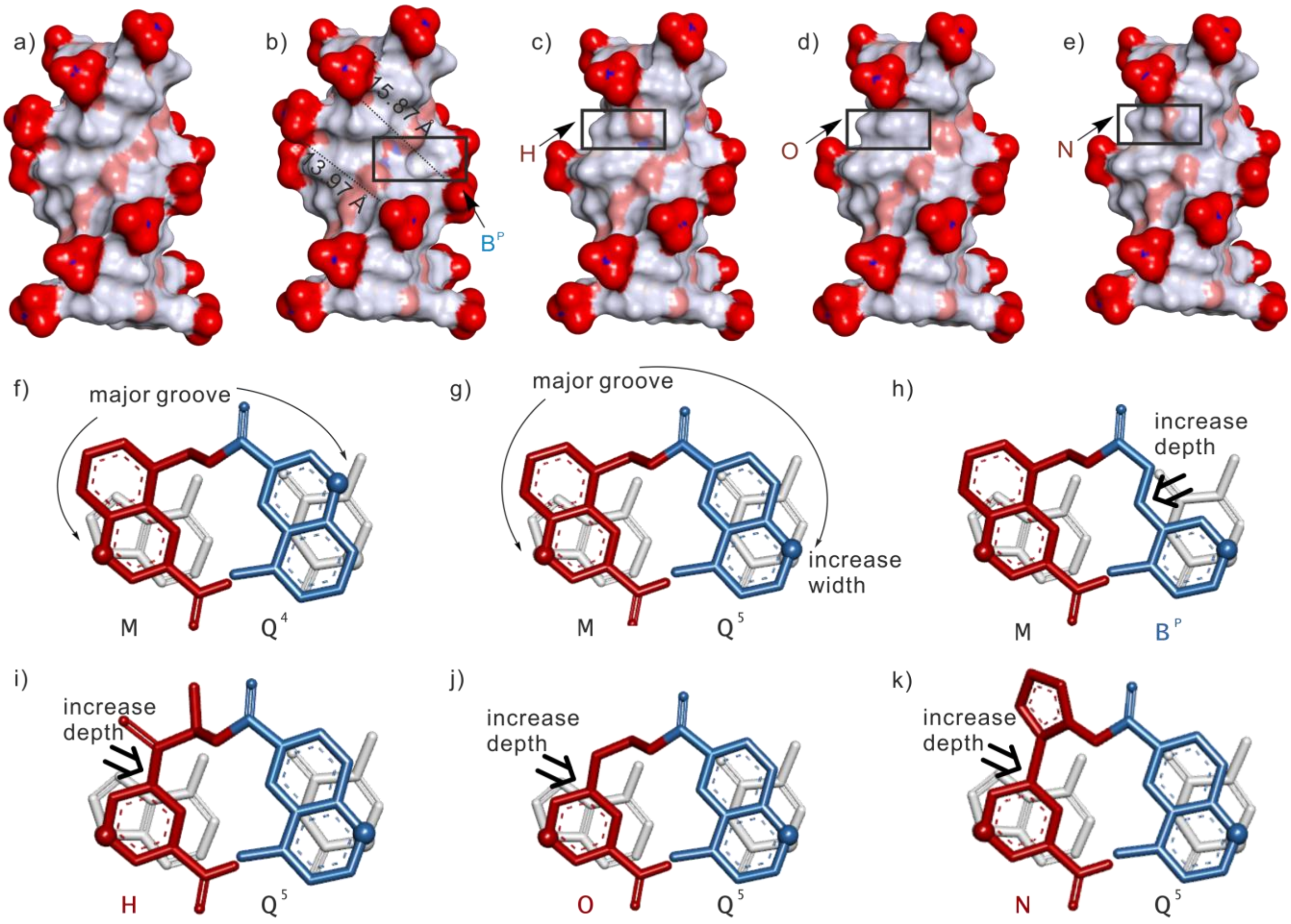

Figure S1. Energy-minimized models of DNA mimic foldamers (Maestro, MMFFs force field with implicit water solvent). Solvent accessible isosurfaces colored by atomic charge of: (a) Parent $(MQ^4)_8$ DNA mimic foldamer. (b) Derivative with Q^4 replaced by B^P, illustrating an increase in groove width (from 14.0 Å to 15.9 Å) and depth. (c–e) Structural variants where M was replaced by (c) H, (d) O, and (e) N, respectively, demonstrating variations in groove depth and polarity. Black rectangles highlight where the new monomers were introduced. Overlay of an AT base pair (gray) with various dimers across the major groove: (f) Q^4M, (g) Q^5M, (h) B^PM, (i) Q^5H, (j) Q^5O, and (k) Q^5N.The red and blue spheres indicate the positions where side chains are anchored. The N1 (pyrimidine) and N7 (purine) of the base pair were made to match the C4 (M) and C5 (Q^5) of the dimer, respectively. Note that the dimers are slightly tilted with respect to the plane of the base pair when incorporated into a helix.

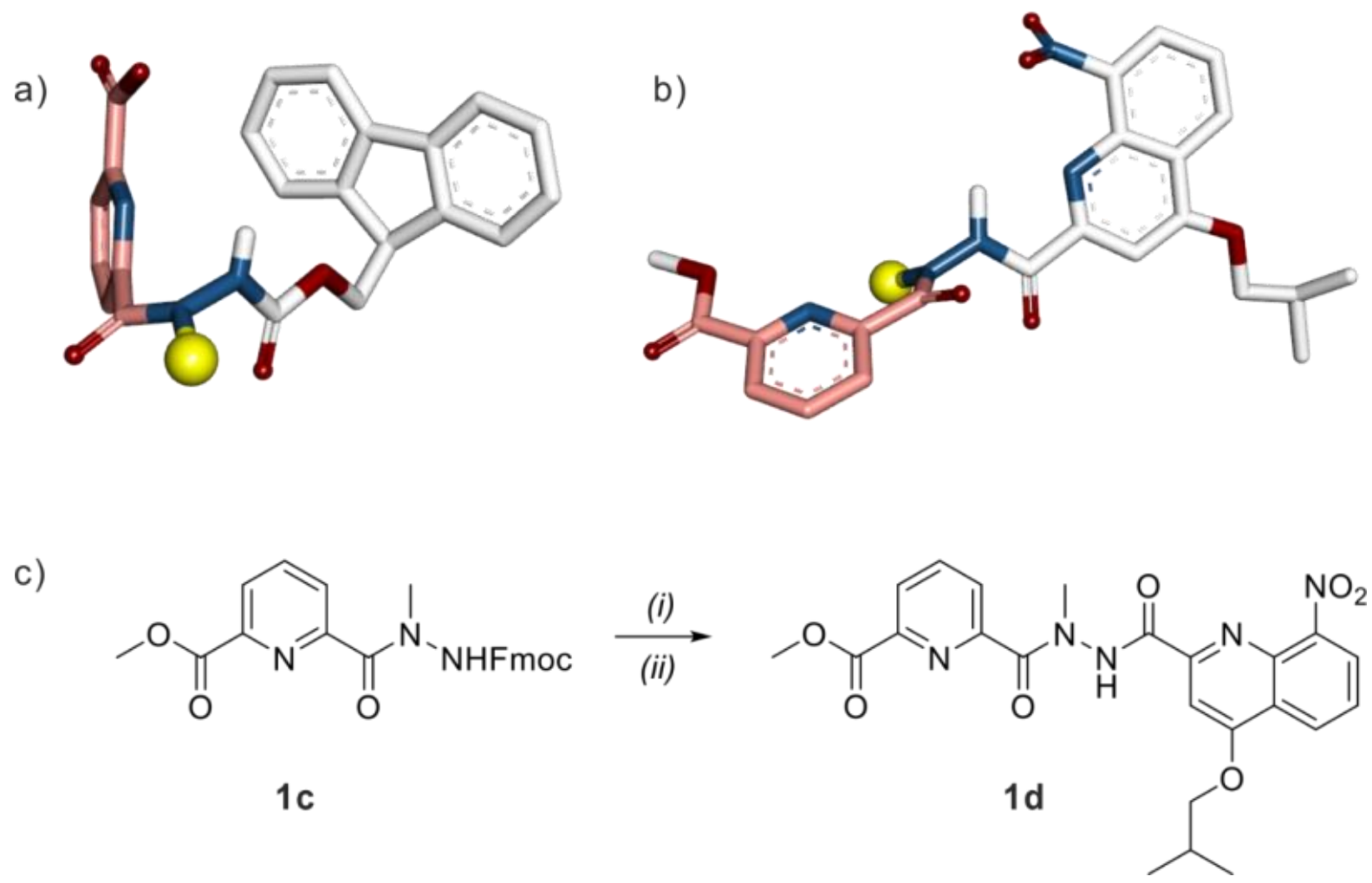

Figure S2. (a) Crystal structure of compound **1**. (b) Crystal structure of compound **1d**. H monomer is highlighted in pink. Methyl group represented as a yellow sphere. (c) Synthetic route to the compound **1d**: (i) DBU, $CHCl_3$, r.t., 2h; (ii) 4-isobutoxy-8-nitroquinoline-2-carboxylic acid, PyBOP, DIPEA, $CHCl_3$, r.t., 2h.

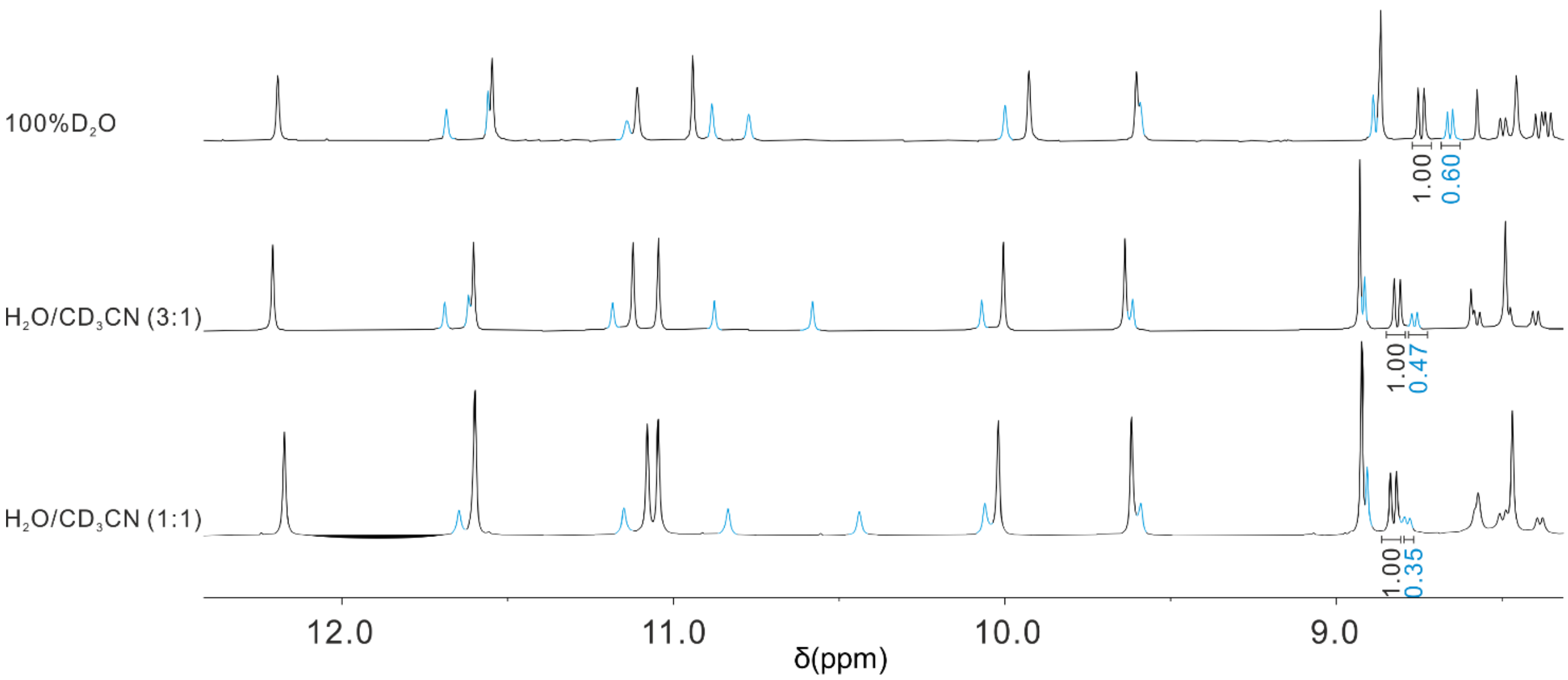

Figure S3. Excerpts of the ^{1}H NMR spectra of sequence **8** at different vol % of CD_3CN. (500 MHz, at 25°C, water suppression). Minor set of signals for sequence **8** is highlighted in blue.

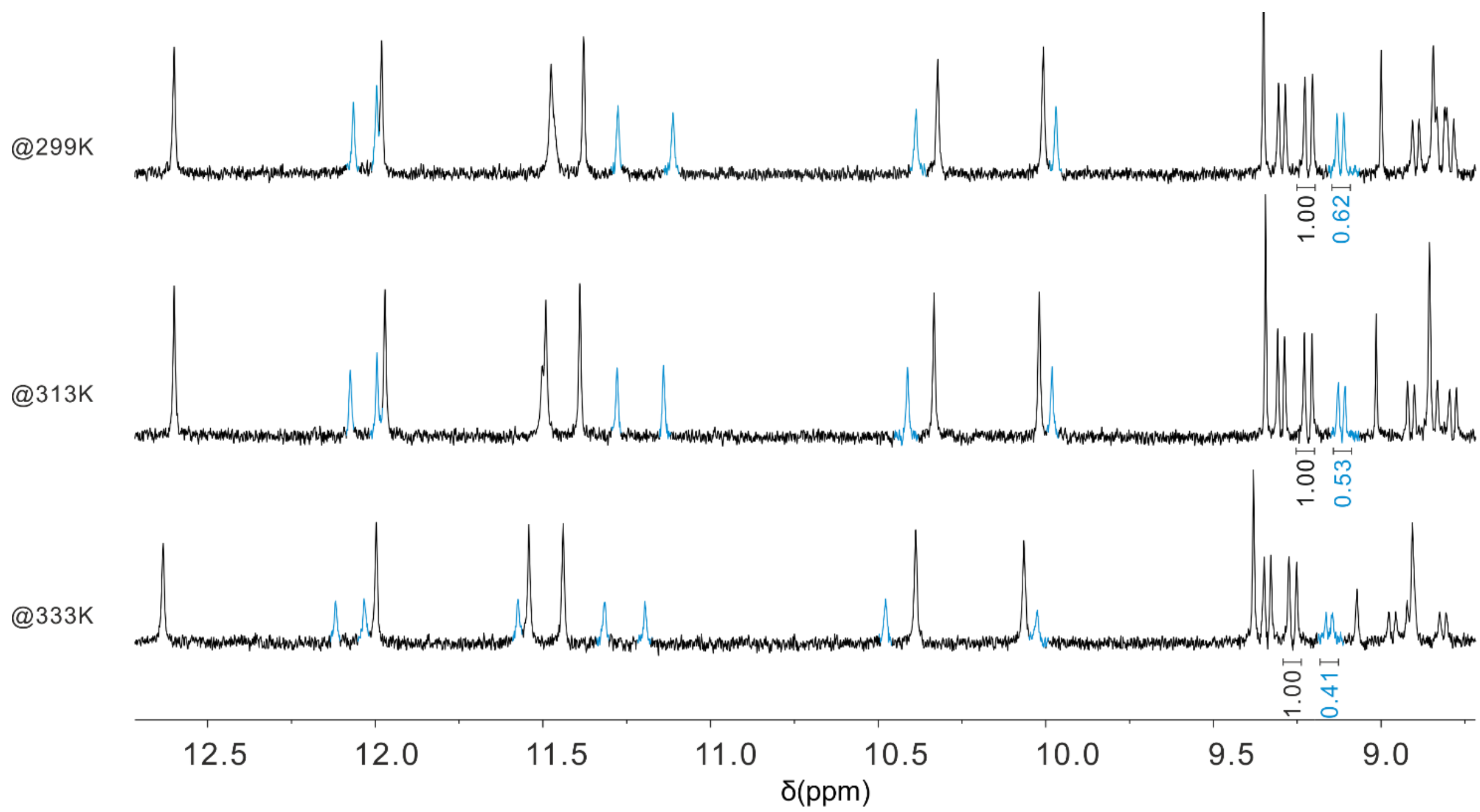

Figure S4. Excerpts of the ^{1}H NMR spectra of sequence **8** at different temperature. (500 MHz, 50 mM NH_4HCO_3, pH 8.5, H_2O/D_2O 9:1 v/v, at 25°C, water suppression). Minor set of signals for sequence **8** is highlighted in blue.

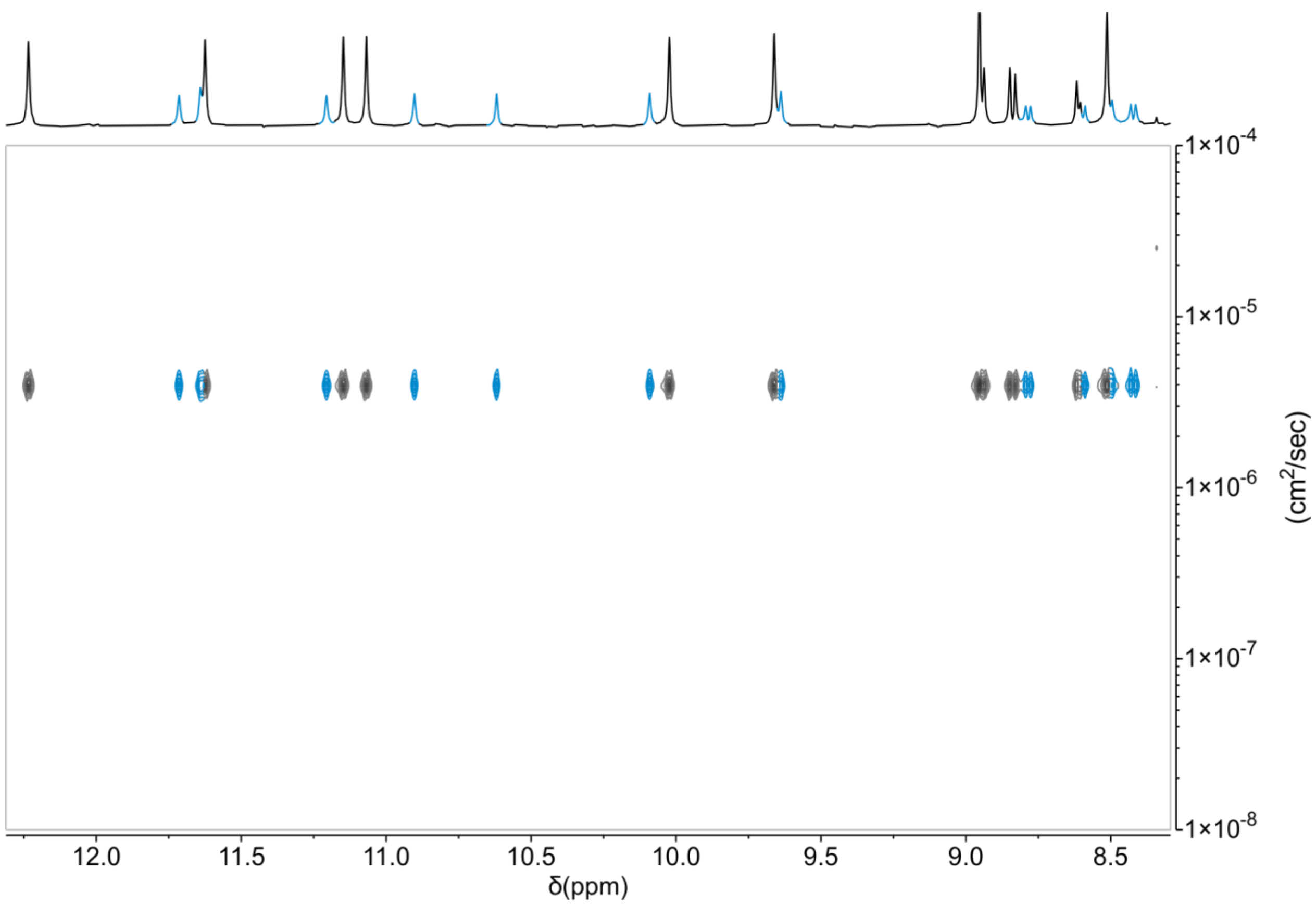

Figure S5. ^{1}H DOSY NMR spectrum of sequence **8** (500 MHz, H_2O/CD_3CN 3:1 v/v, at 25°C, water suppression). Minor set of signals for sequence **8** is highlighted in blue.

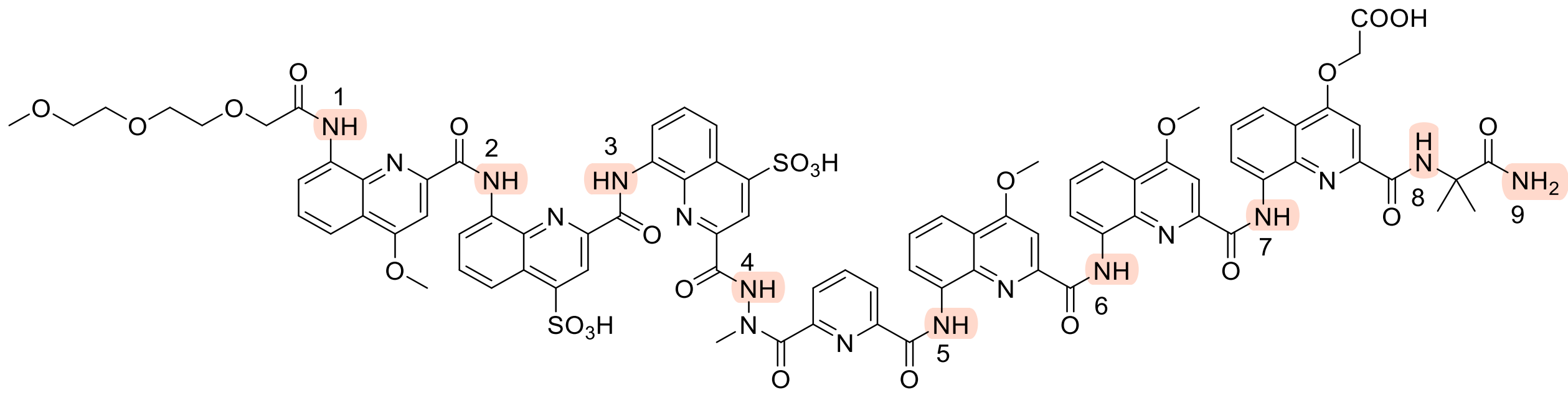

Sequence 8

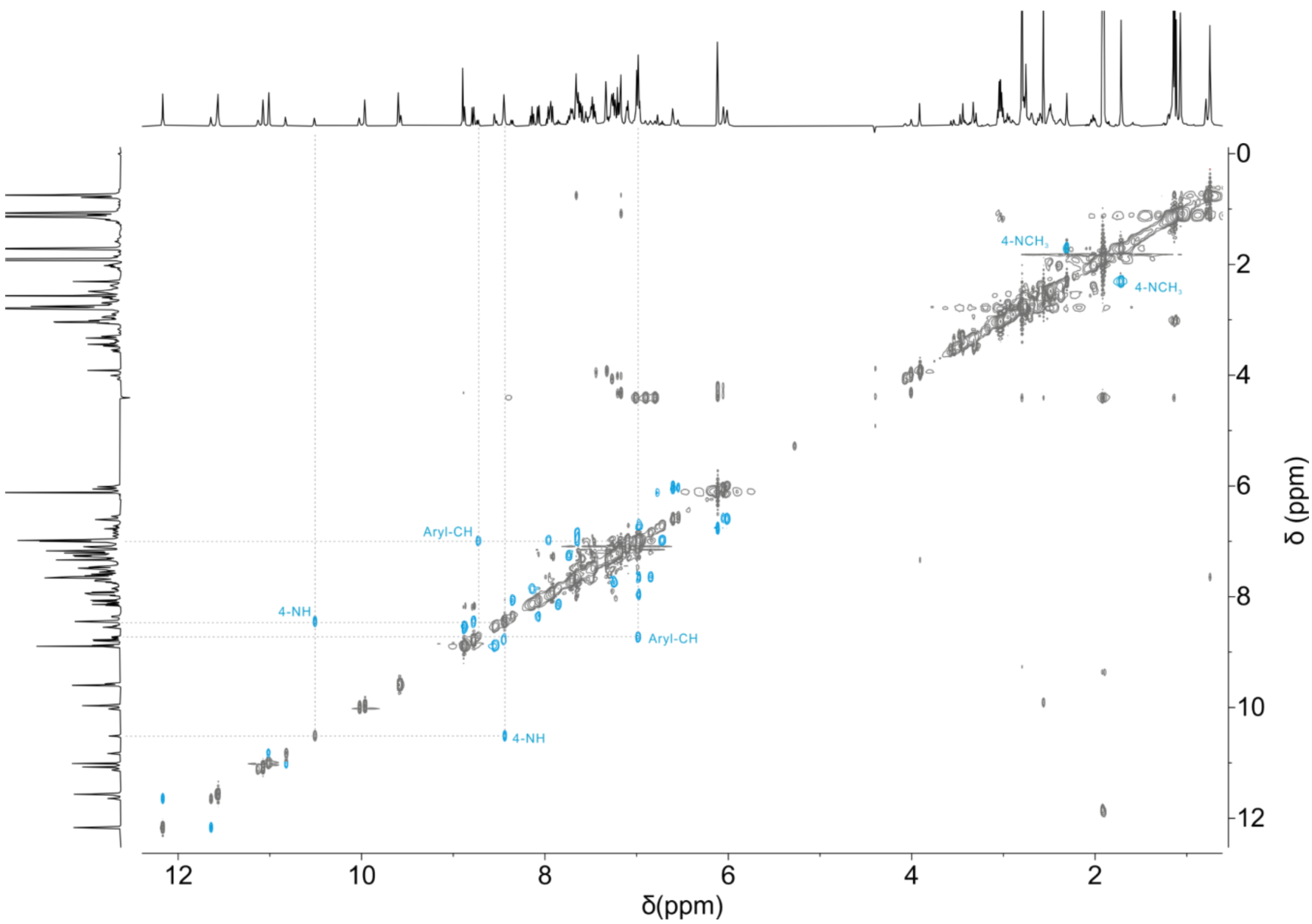

Figure S6. 2D ROESY NMR spectrum of sequence **8** (500 MHz, H_2O/CD_3CN 3:1 v/v, at 25°C, water suppression). Exchange cross-peaks between different conformers are highlighted in light blue.

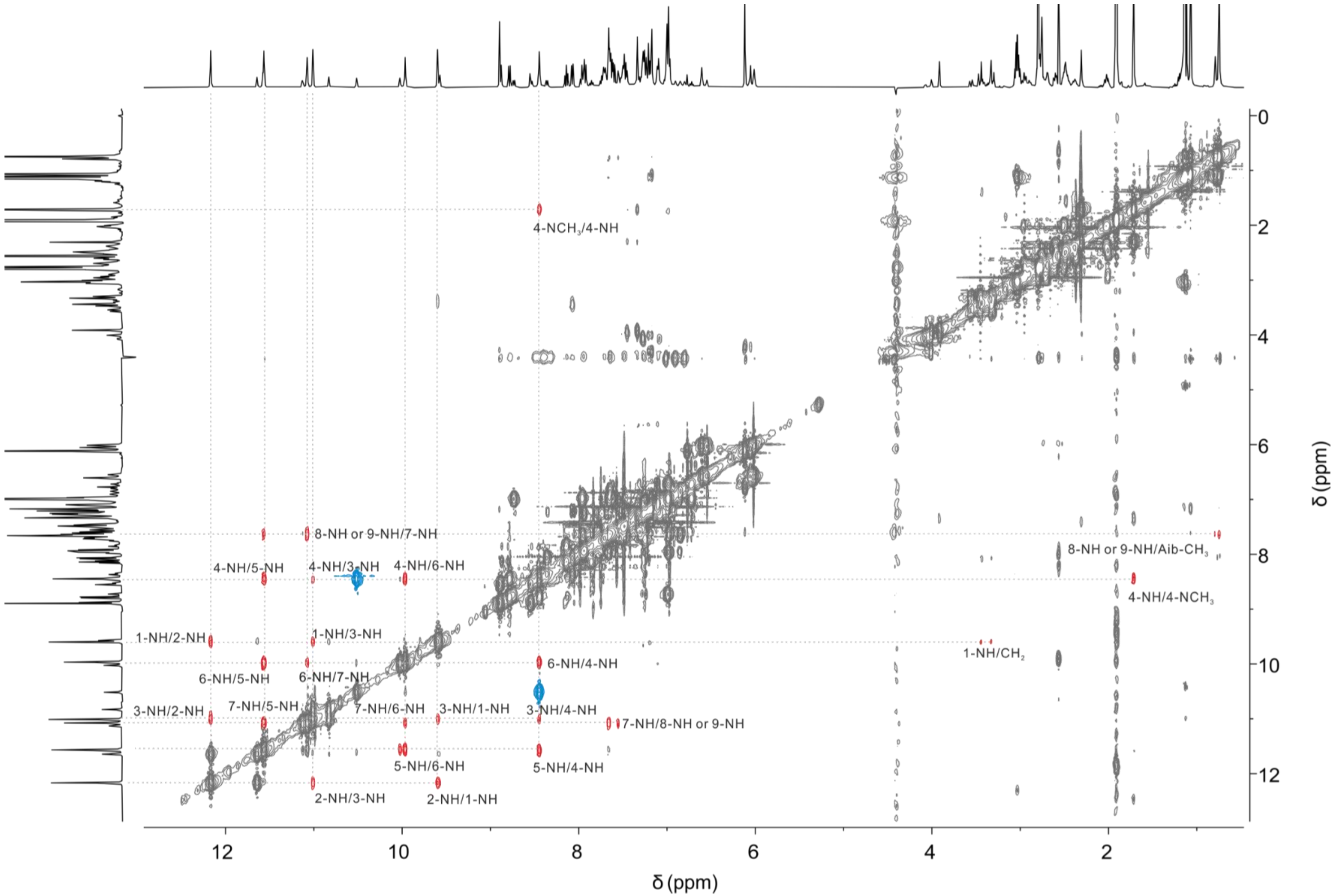

Figure S7. 2D NOESY NMR spectrum of sequence **8** (500 MHz, H_2O/CD_3CN 3:1 v/v, at 25°C, water suppression). Exchange cross-peaks between different conformers are highlighted in light blue. The NOE correlations involving the amide (NH) protons are highlighted in red.

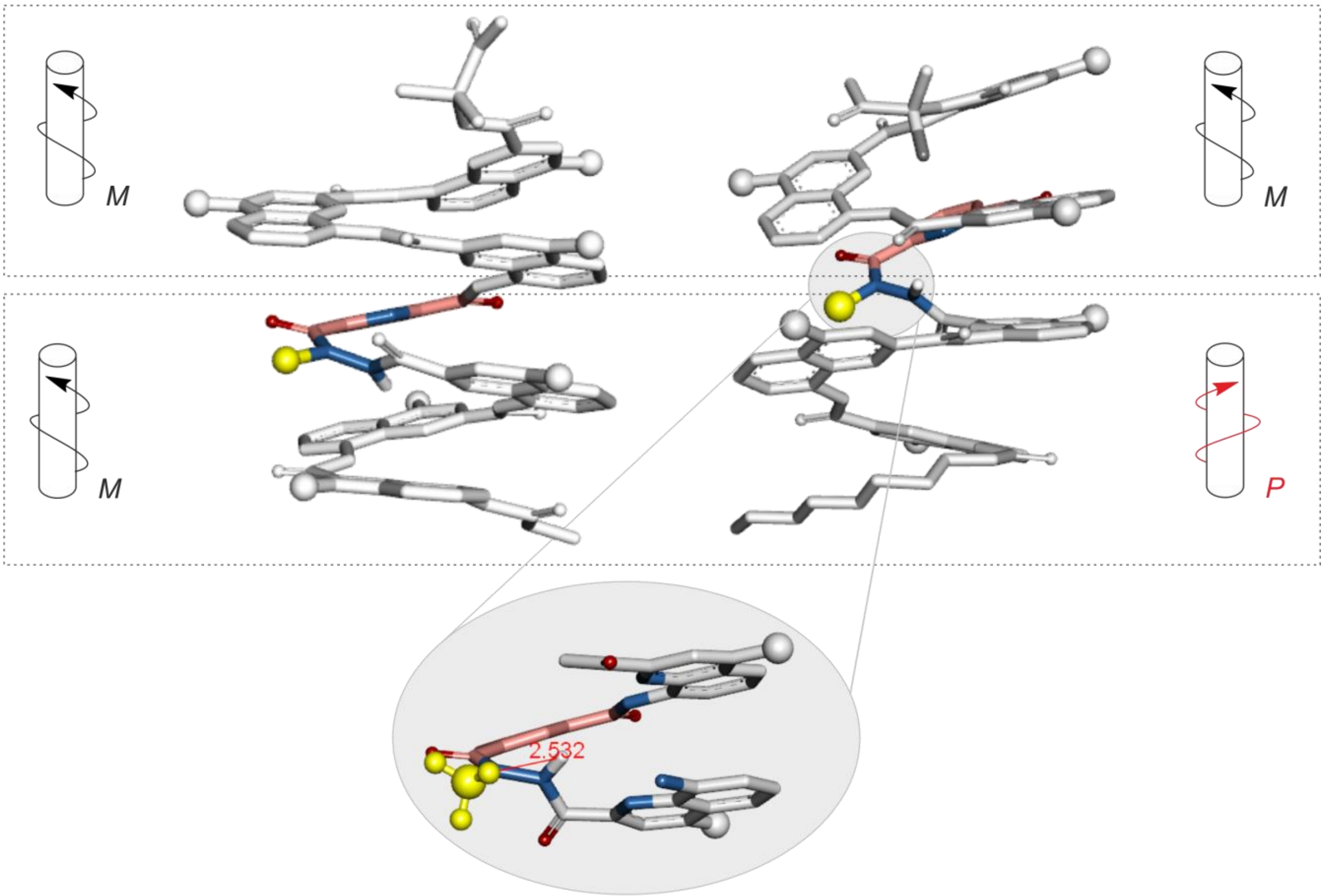

Figure S8. Left: Crystal structure of sequence **8** showing M-helicity. **Right:** Molecular model of a conformer of sequence **8** with opposite helicity on both sides of the H monomer (highlighted in pink). **Gray inset:** Close-up of the H monomer showing a flipped amide bond; the distance between the methyl group (yellow) and the NH proton is 2.5 Å. This correlation was observed by NOESY (see Fig. S6).

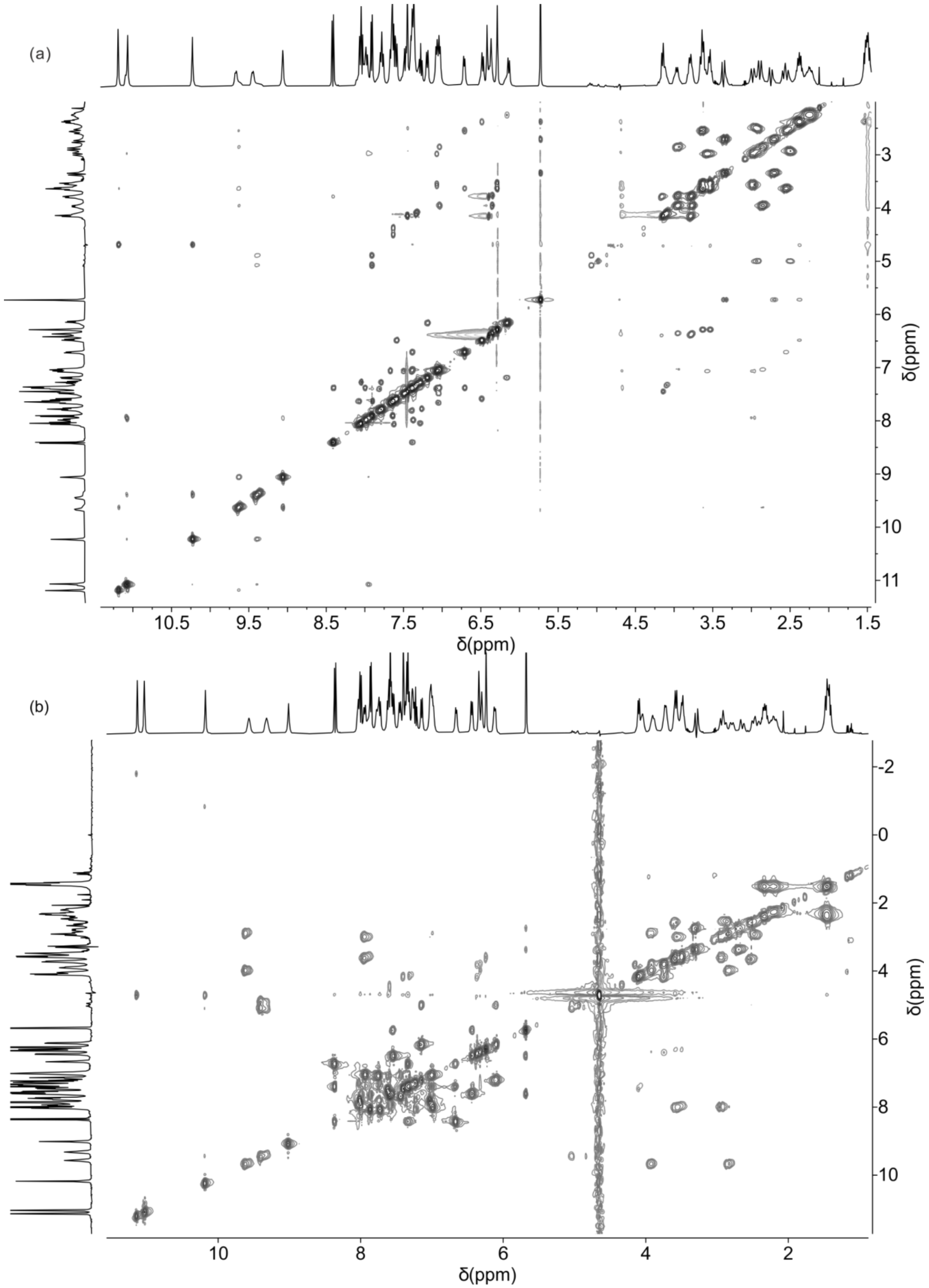

Figure S9. 2D NMR spectra of sequence **12** showing the helical folding. (a) ^1H-^1H NOESY; (b) ^1H-^1H TOCSY (500 MHz, 50 mM NH_4HCO_3, pH 8.5, H_2O/D_2O 9:1 v/v, at 25°C, water suppression).

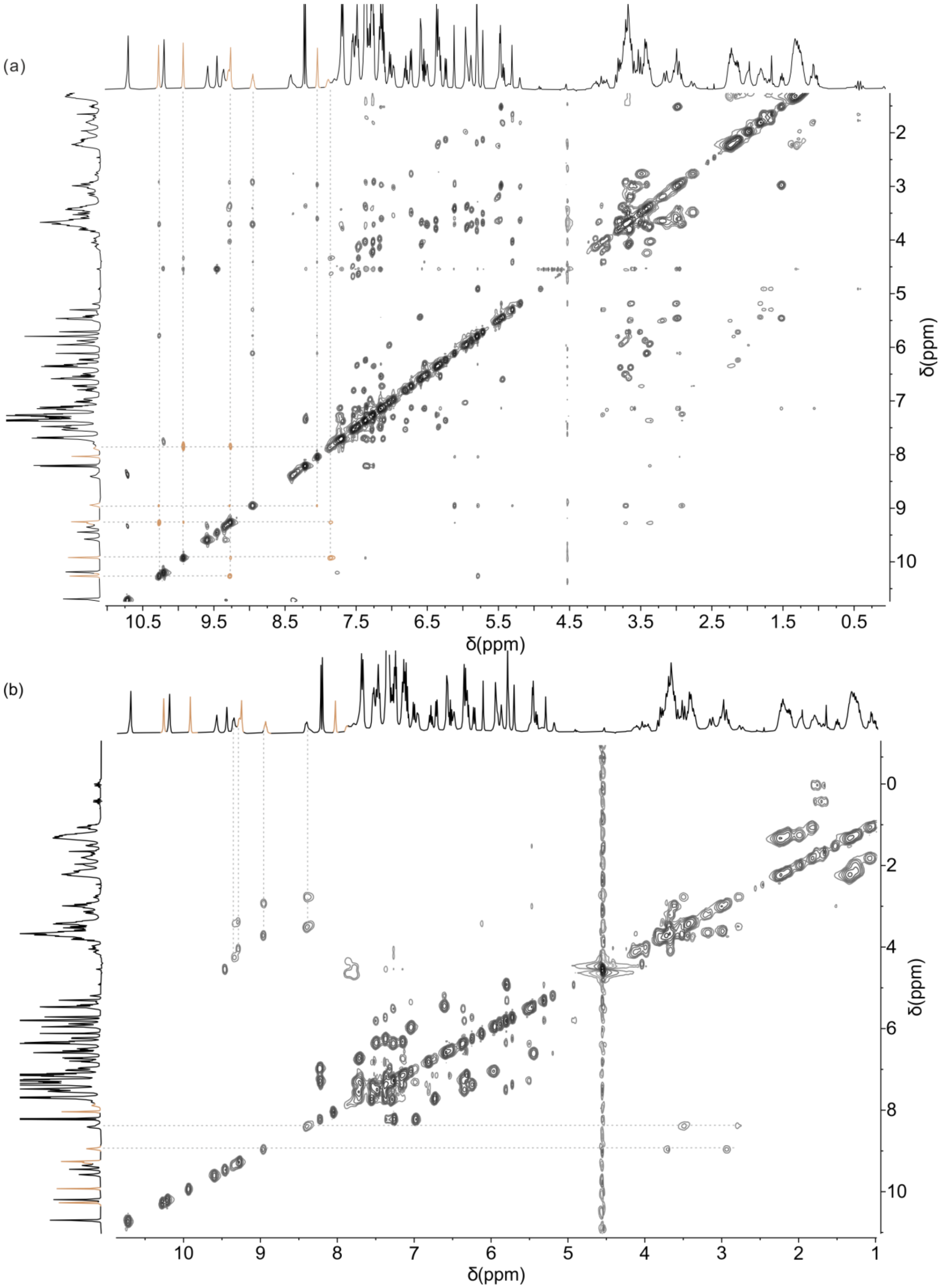

Figure S10. 2D NMR spectra of sequence **13** showing the helical folding and aggregation. (a) ^1H-^1H NOESY; (b) ^1H-^1H TOCSY (500 MHz, 50 mM NH_4HCO_3, pH 8.5, H_2O/D_2O 9:1 v/v, at 25°C, water suppression). Signals that correspond to two different species are highlighted in black and orange, respectively.

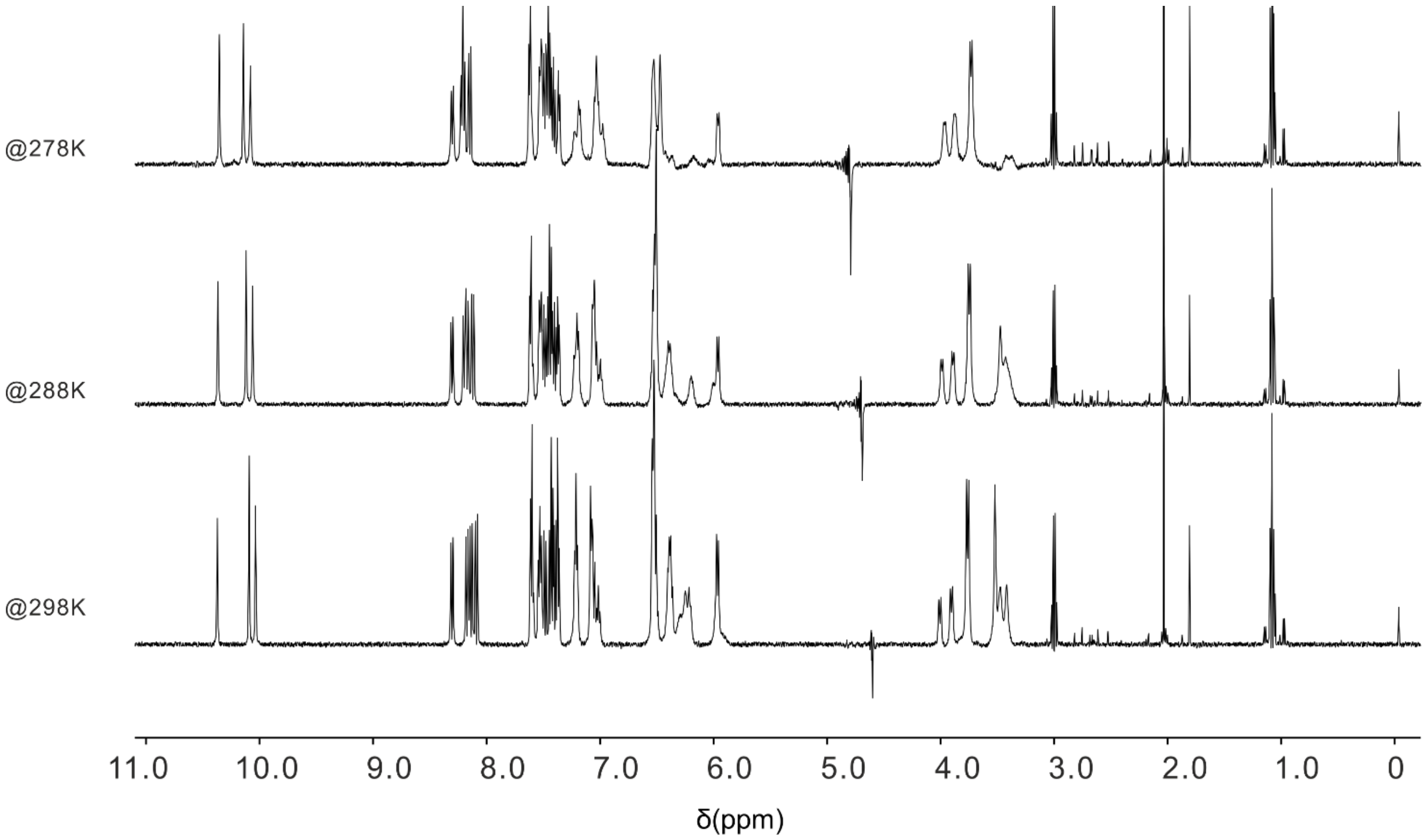

Figure S11. Excerpts of the ^{1}H NMR spectra of sequence **14** at different temperature. (500 MHz, 50 mM NH_4HCO_3, pH 8.5, H_2O/D_2O 9:1 v/v, at 25°C, water suppression).

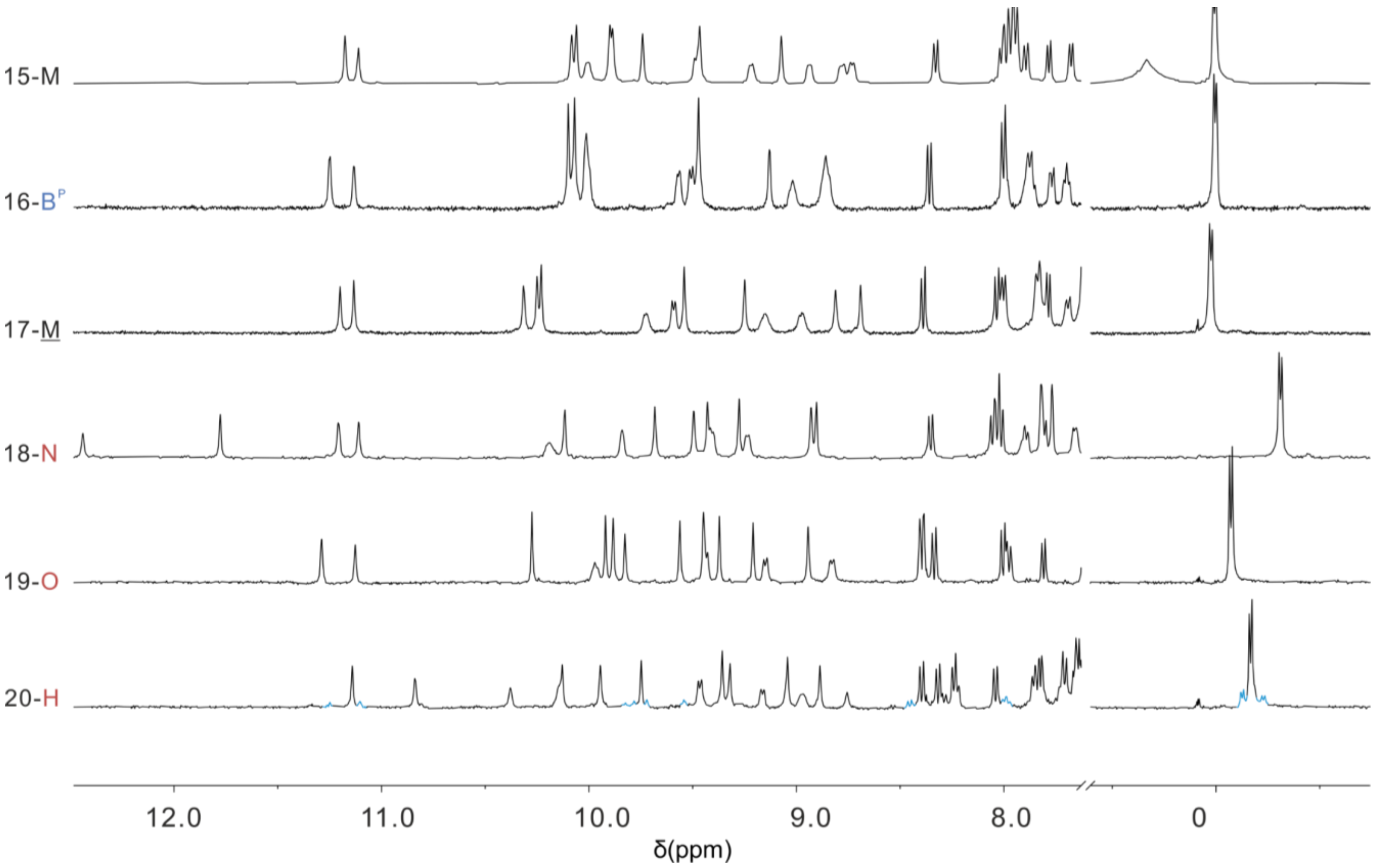

Figure S12. Excerpts of the ^{1}H NMR spectra of sequence **15-20**. (500 MHz, 50 mM NH_4HCO_3, pH 8.5, H_2O/D_2O 9:1 v/v, at 25°C, water suppression). Minor set of signals for sequence **20** is highlighted in blue.

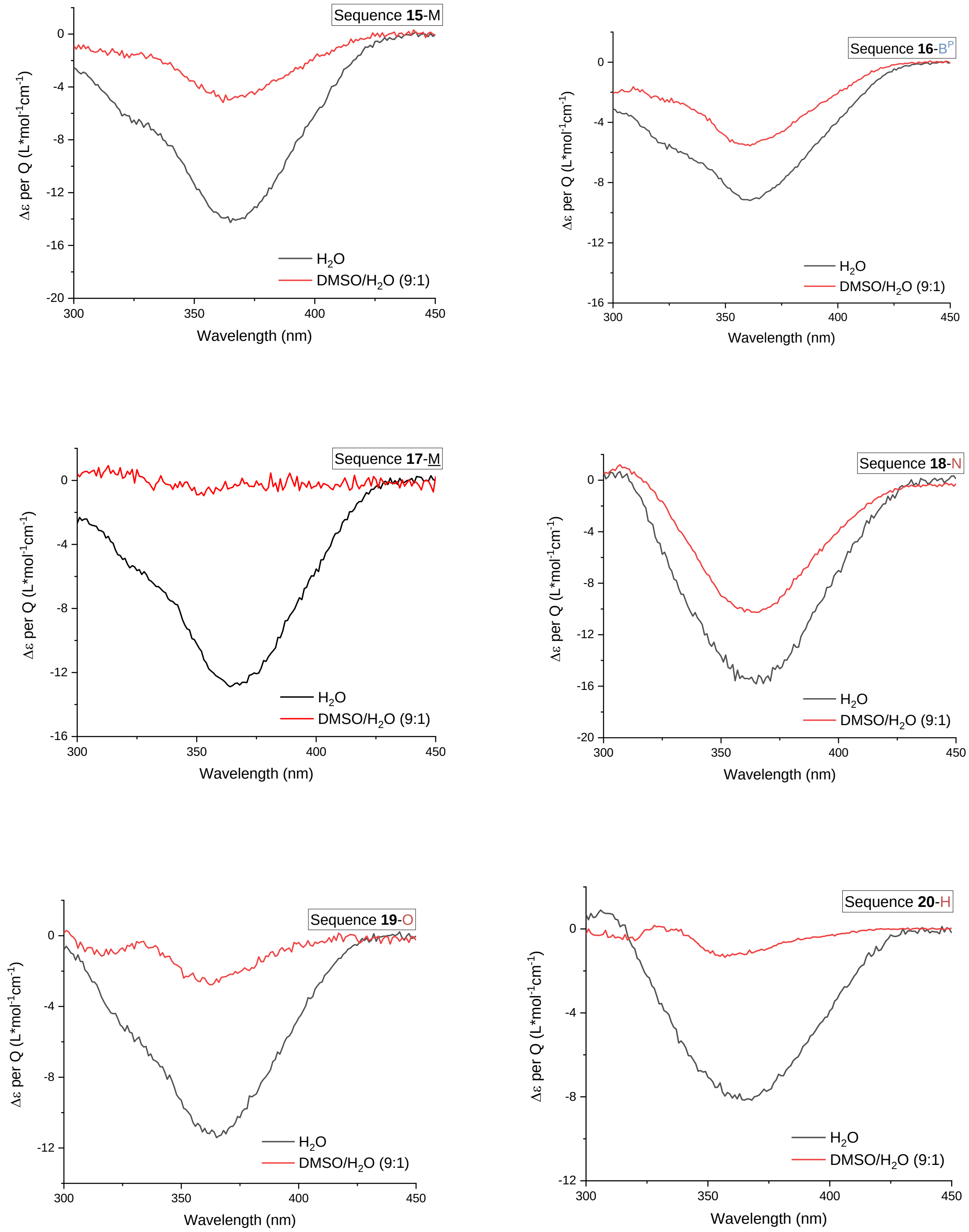

Figure S13. CD spectra of sequence **15**-**20** in H_2O and in DMSO/ H_2O (9:1 v/v) at 50 °C.

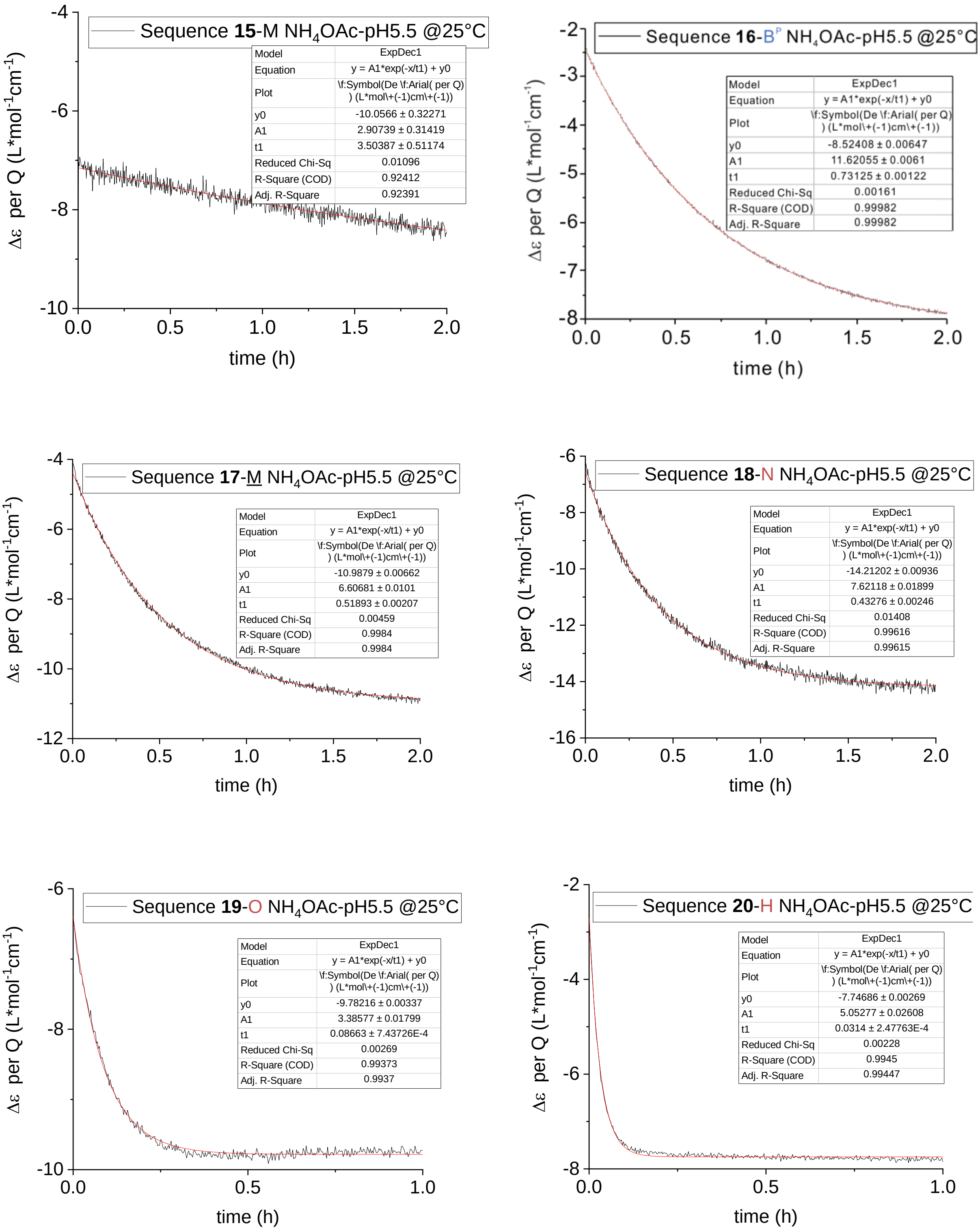

Figure S14. Helix-handedness enrichment of **15**-**20**, showing conversion of excess *P*-helix to *M*-helix (black), and a single-exponential decay fitted to the corresponding data (red) in 50 mM NH_4OAc at pH 5.5.

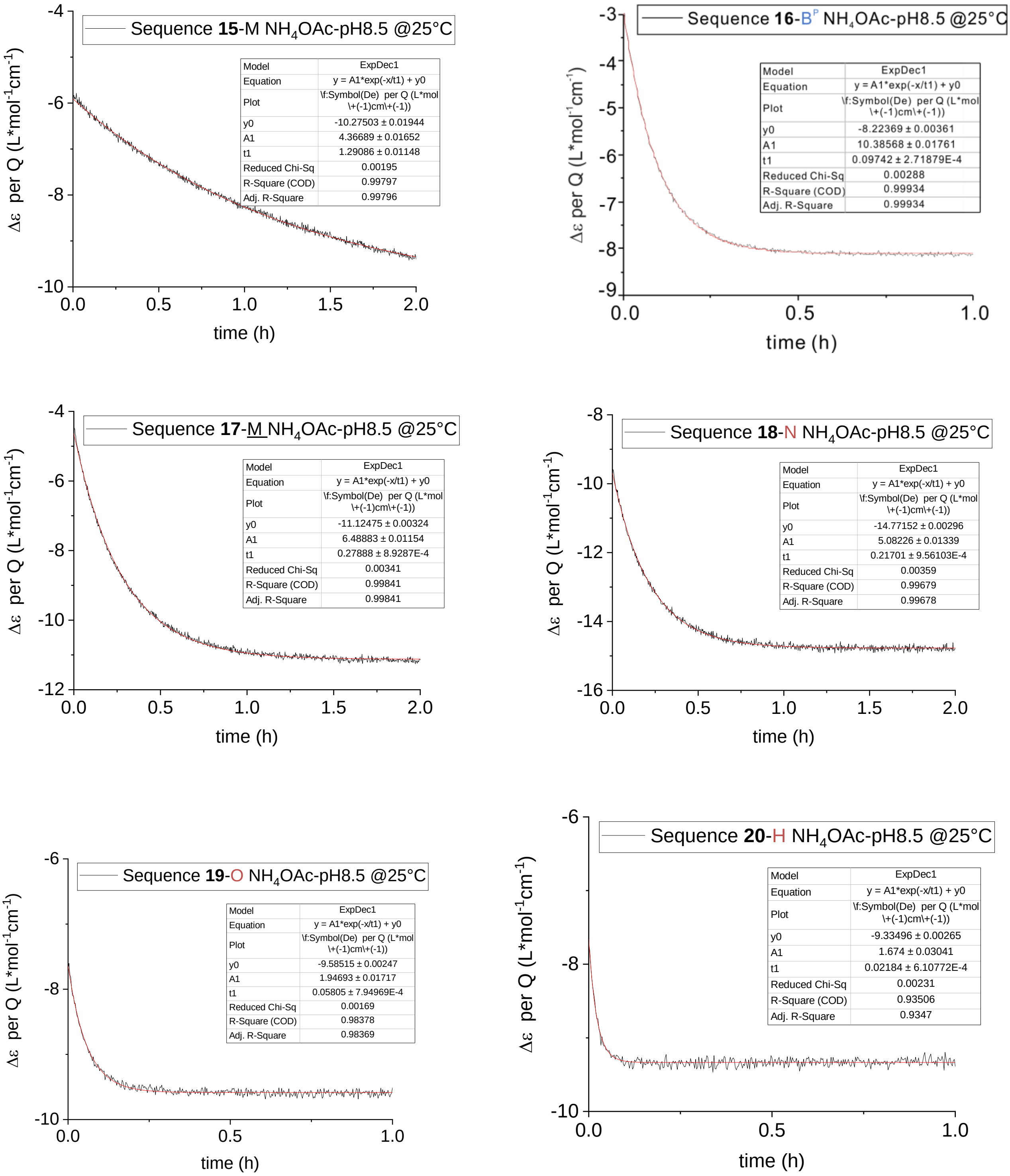

Figure S15. Helix-handedness enrichment of **15**-**20**, showing conversion of excess *P*-helix to *M*-helix (black), and a single-exponential decay fitted to the corresponding data (red) in 50 mM NH_4OAc at pH 8.5.

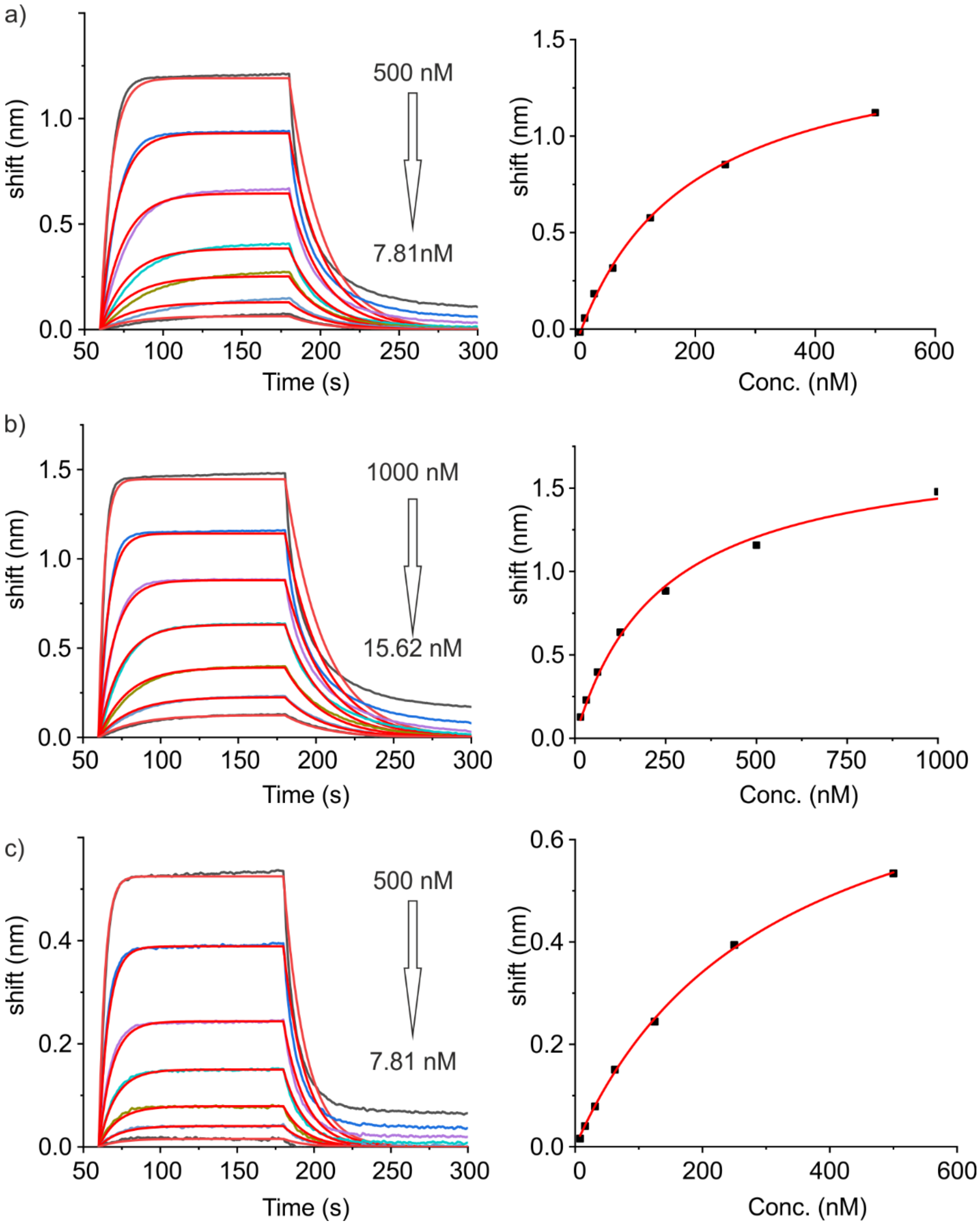

Figure S16. BLI sensorgrams of HU protein binding to biotinylated foldamers **17a** (a), **18a** (b) and **19a** (c) immobilized on streptavidin sensors. The sensorgrams were fitted to a 1:1 kinetic binding model (left graphs, global fitting) or to 1:1 steady state binding model (right graphs). Calculated curves are shown in red.

3. Materials and Methods

3.1 General

Chemicals and reagents were used as commercially supplied without any further purification unless otherwise stated. Low loading Wang resin (0.41 mmol g^{-1}) was purchased from Novabiochem. Analytical grade organic solvents were used for SPS. Anhydrous THF and DCM for SPS were dispensed from an *MBRAUN Solvent Purification System-800* solvent purification system. Reactions requiring anhydrous conditions were performed under nitrogen. Protected Fmoc-acid building blocks are shown in Figure S12.

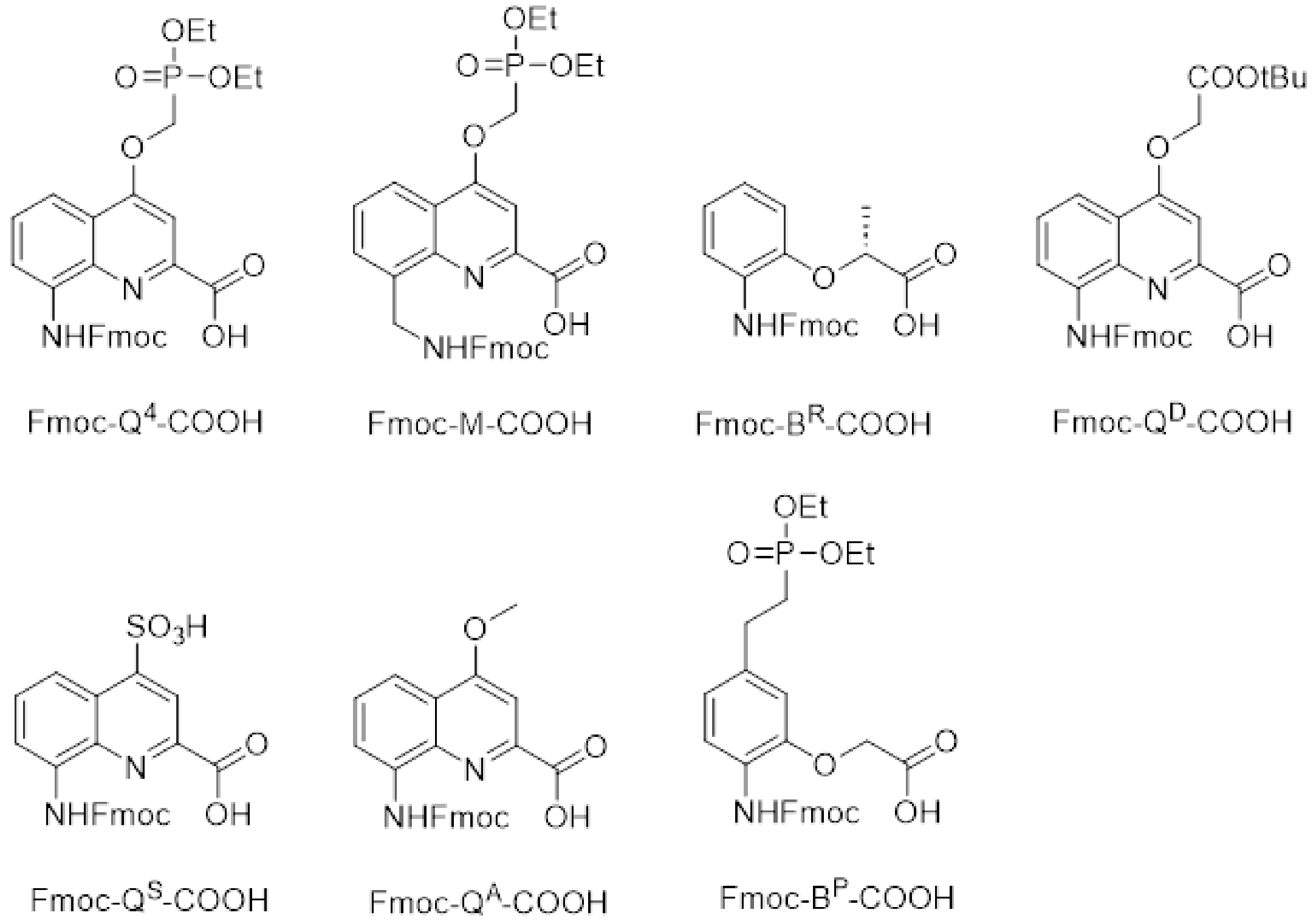

Figure S17. Side chain-protected Fmoc-acid building blocks used in this study. Fmoc-Q^4-OH, Fmoc-M-OH, Fmoc-B^R-OH,[1] Fmoc-Q^A-OH,[2] Fmoc-Q^S-OH,[3] Fmoc-Q^D-OH[4] have been described previously. For a detailed procedure to Fmoc-B-OH, see section 3.2.

Analytical RP-HPLC was performed on a Thermo Fisher Scientific Ultimate 3000 HPLC system equipped with a Macherey-Nagel Nucleodur C18 column (4.6 × 150 mm, 5 µm) at a flow rate of 1.0 mL/min. Semi-preparative RP-HPLC purification was carried out using a Kinetex C18 EVO column (10 × 100 mm, 5 µm) at a flow rate of 5.0 mL/min. For oligomer **5**-**9,** the mobile phase was composed of 12.5 mM NH_4OAc in water at pH 8.5 (solvent A) and acetonitrile (solvent B). For oligomer **12**-**20**, 0.1% TFA was added to aqueous mobile phase (referred to as mobile phase A) and to acetonitrile

(referred to as mobile phase B) under acidic conditions. When using basic conditions, the mobile phase was composed of 12.5 mM TEAA in water at pH 8.5 (solvent A) and 12.5 mM TEAA in water: acetonitrile 1:2 v/v at pH 8.5 (solvent B).

NMR spectra were recorded on different NMR spectrometers: (1) an Avance III HD NMR spectrometer 400 MHz (Bruker BioSpin) for ^{1}H NMR and ^{13}C NMR spectra of some small molecules. (2) an Avance III HD NMR spectrometer 500 MHz (Bruker BioSpin) with CryoProbe™ Prodigy for ^{1}H NMR, ^{13}C NMR, ^{1}H,^{15}N HSQC, and DOSY spectra of some small molecules and foldamers. All NMR measurements were performed at 25 °C unless specified. Chemical shifts are described in part per million (ppm, δ) relative to the ^{1}H residual signal of the deuterated solvent used – meaning DMSO-*d₆* (δ_H: 2.50 ppm), $CDCl_3$ (δ_H: 7.26 ppm) and D_2O (δ_H: 4.79) For the H_2O/CD_3CN solvent mixtures, the chemical shifts were calibrated according to CD_3CN (δ 1.94 ppm). Water suppression was performed with excitation sculpting. ^{1}H NMR splitting patterns with observed first-order coupling are entitled as singlet (s), broad singlet (bs), doublet (d), triplet (t), doublet of doublets (dd) or multiplet (m). Coupling constants (J) are reported in Hz.

^{1}H,^{15}N-HSQC spectra were recorded with a phase-sensitive pulse sequence with flip-back pulse for water suppression applying a watergate sequence (hsqcfpf3gpphwg) from the Bruker pulse program library modified to make the sequence compatible with a 2-channel spectrometer. Data acquisition was performed with 512 (F2) x 64 (F1) data points in States-TPPI acquisition mode. The recycling delay was 1.0 s and 2048 transients per increment were applied at a sweep width of 8.5 kHz in F2 and 5 kHz in F1 resulting in an acquisition time of 0.062 s. Zero filling in F1 has been used to yield a final matrix of 512 x 512 real points.

The DOSY spectrum was recorded applying a pulse sequence with stimulated echo using bipolar gradient pulses for diffusion from the Bruker pulse program library (stebpesgp1s). The diffusion delay Δ (big delta) was set to 1 ms and the diffusion gradient pulse length δ (little delta) was set to 100 ms. The number of gradient steps were set to 32 with linear spacing starting from 2% reaching 95% of the full gradient strength in the final step. For each of the 32 gradient amplitudes, 64 transients of 65k complex data points were acquired. DOSY processing was performed with the DOSY processing tool from MestReNova employing the Peak Heights Fit algorithm including autocorrect peak position.

2D TOCSY spectra were recorded with a phase-sensitive pulse sequence using composite pulse scheme MLEV with water suppression employing an excitation sculpting element (mlevesgpph)

from the Bruker pulse program library. Data acquisition was performed with 2048 (F2) x 256 (F1) data points in States-TPPI mode. The recycling delay was 2.0 s and 8 transients per increment were applied at a sweep width of 8 kHz in both dimensions resulting in an acquisition time of 0.1283 s. The TOCSY mixing time was set to 80 ms. Special acquisition parameters regarding the water suppression element of the pulse sequence were adopted from the optimized parameter set of the respective one-dimensional experiment.

The 2D ROESY spectrum was recorded with a phase-sensitive pulse sequence with water suppression employing an excitation sculpting element from the Bruker pulse program library (roesyesgpph). Data acquisition was performed with 1K (F2) x 256 (F1) data points and a mixing time of 0.2 s. The recycling delay was 1.0 s and 32 transients per increment were applied at a sweep width of 8 kHz in both dimensions resulting in an acquisition time of 1.64 s.

^{1}H,^{1}H NOESY spectra were recorded with a phase-sensitive pulse sequence from the Bruker pulse program library (noesygpph). Data acquisition was performed with 1K (F2) x 512 (F1) data points and a mixing time of 0.2 s. The recycling delay was 2 s and 16 transients per increment were applied at a sweep width of 7.5 kHz in both dimensions resulting in an acquisition time of 0.15 s.

The molecular models of DNA mimic foldamers were built in Maestro (Version 11.5)[5] and energy-minimized with the following parameters: force field: MMFFs; solvent: water; electrostatic treatment: constant dielectric; dielectric constant: 1.0; charges from: force field; cutoff: extended; mini method: SD; maximum iterations: 25000; converge on gradient; convergence threshold: 0.05.

LCMS analysis was conducted on a Thermo Fisher Scientific Ultimate 3000 HPLC system with an NH_4OAc buffer system consisting of 12.5 mM NH_4OAc dissolved in ultra-pure water and adjusted to pH 8.5 with aqueous ammonia (referred to as mobile phase A) and LCMS-grade acetonitrile (referred to as mobile phase B) on a Kinetex C18 EVO column (2.1 x 50 mm, 1.8 µm) column and a flowrate of 0.33 mL/min. In all cases, elution was monitored by UV detection at 254 and 300 nm with a diode array detector. For LCMS analysis, the LC system was coupled to a micrOTOF II mass spectrometer by Bruker Daltonics and molecules were ionized by ESI.

CD spectra were recorded on a Jasco J-1500 spectrometer with 1 mm quartz cuvette. The following parameters were used: wavelength range from 450 to 300 nm. Scan speed: 50 nm/min; accumulation: 2; response time: 1.0 s; bandwidth: 1; temperature: 20 °C; sensitivity: standard (100 mdeg); data pitch: 1 nm; nitrogen gas flow rate: 500 L/h. Molar extinction values were normalized

per quinoline units. The data shown are the mean of two measurements and were smoothed using a Savitzky-Golay filter with a polynomal order of 3. Time-course measurements were recorded at 375 nm with a D.I.T of 2 seconds and a data pitch of 10.0 seconds with a Peltier element for temperature control.

UV-Vis spectra were measured on a Jasco V-750 spectrophotometer with a peltier element for temperature control. Spectra were recorded from 450 to 300 nm, a bandwidth of 2.00 nm, a continuous scanning mode with a scanning speed of 400 nm/min and a UV-Vis response of 0.06 s. All spectra were recorded in 1 mm quartz glass cuvettes at a concentration range of 30-40 µM for CD spectra and 50 µM for UV spectra. Baseline correction with the respective solvent or buffer used was implemented. DNA mimic foldamers are readily soluble in water. Concentrations were determined by UV-absorbance using an average ε value at 375 nm per monomer of 2506 $Lmol^{-1}cm^{-1}$ for $(MQ^4)_n$ sequences.[6]

3.2 Monomer synthesis procedures

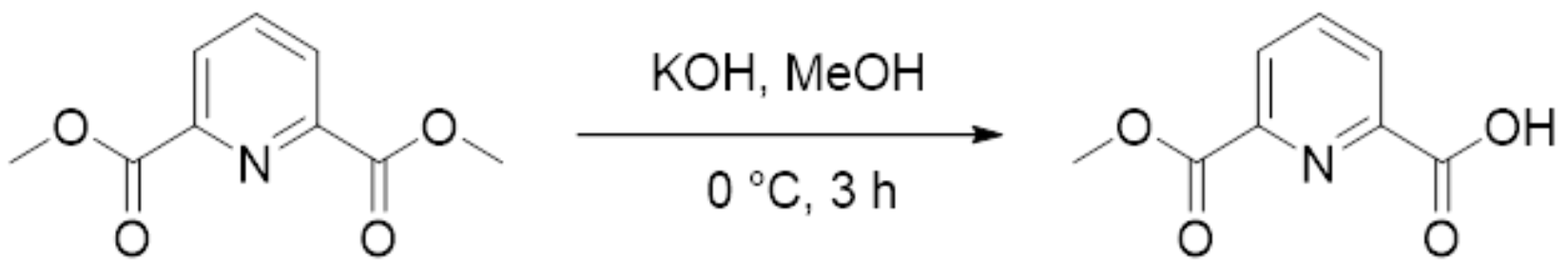

1a

6-(Methoxycarbonyl)picolinic acid (1a): Dimethyl pyridine-2,6-dicarboxylate (3.8 g, 19.5 mmol, 1.0 eq) was dissolved in MeOH (150 mL) and the solution was cooled to 0 °C in an ice bath. KOH pellets (1.09 g, 19.5 mmol, 1.0 eq) were added portion wise, and the reaction mixture was stirred at 0 °C for 4 h. The solvent was then removed under reduced pressure. The solid residue was washed thoroughly with ethyl acetate, then dissolved in water and acidified to pH 2 using 4 M HCl. The aqueous layer was extracted with chloroform (3 × 50 mL), and the combined organic layers were dried over anhydrous $MgSO_4$. After filtration, the solvent was evaporated under reduced pressure to afford the product as a white solid (2.35 g, 67%). **^{1}H NMR (500 MHz, $CDCl_3$):** δ (ppm) = 13.11 (s, 1H), 7.67 – 7.41 (m, 3H), 3.21 (s, 3H).[7]

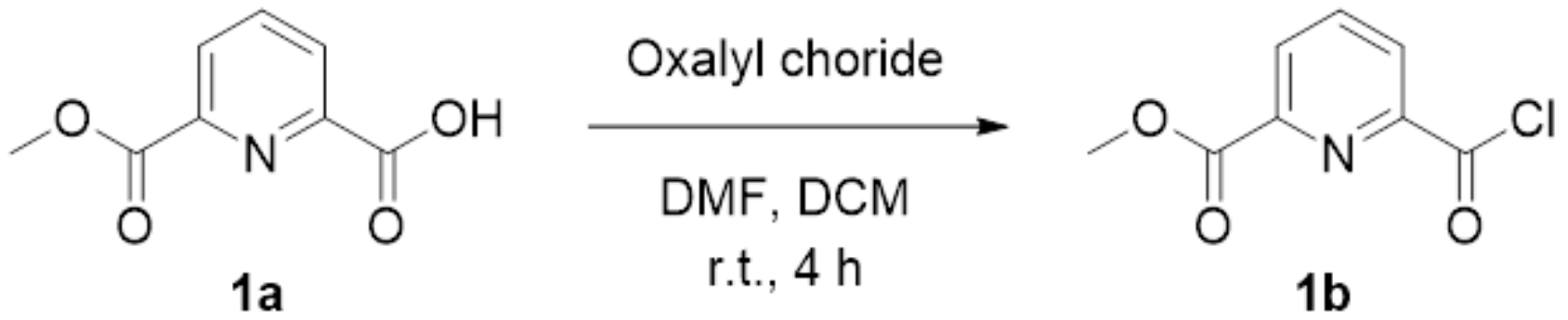

1a **1b**

Methyl 6-(chlorocarbonyl)picolinate (1b): 6-(Methoxycarbonyl)picolinic acid **1a** (2.35 g, 13 mmol, 1.0 eq) was dissolved in anhydrous DCM (26 mL) and cooled to 0 °C under a N_2 atmosphere. To the stirred solution, two drops of DMF were added, followed by the dropwise addition of oxalyl chloride (1.34 mL, 15.5 mmol, 1.2 eq) over 10 min. The reaction mixture was stirred at r.t. for 3 h. The solvent and excess reagents were then removed under reduced pressure overnight. The crude acid chloride was used in the next step without further purification. **^{1}H NMR (500 MHz, $CDCl_3$):** δ (ppm) = 8.40 (dd, *J* = 7.8, 1.1 Hz, 1H), 8.29 (dd, *J* = 7.8, 1.1 Hz, 1H), 8.09 (t, *J* = 7.8 Hz, 1H), 4.06 (s, 3H).[8]

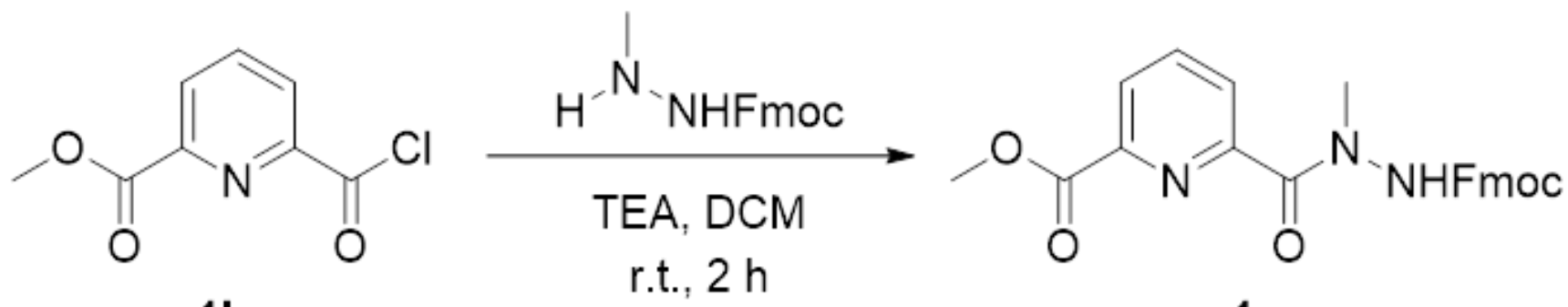

Methyl 6-(2-(((9*H*-fluoren-9-yl)methoxy)carbonyl)-1-methylhydrazine-1-carbonyl)picolinate (1c): Fmoc-methylhydrazine (3.83 g, 14.3 mmol, 1.1 eq) was dissolved in anhydrous DCM (40 mL), followed by the addition of triethylamine (2.00 mL, 14.3 mmol, 1.1 eq). A solution of methyl 6-(chlorocarbonyl)picolinate **1b** in DCM (20 mL) was added dropwise to the reaction mixture at 0 °C. The reaction was then stirred at r.t. for 2 h. The reaction mixture was poured into water and extracted with DCM (3 × 50 mL). The combined organic layers were washed sequentially with saturated $NaHCO_3$ solution and brine, then dried over Na_2SO_4. The solvent was removed under reduced pressure to afford the product as a white solid (3.2 g, 57%). **^{1}H NMR (500 MHz, $CDCl_3$):** δ (ppm) = 8.13 (d br, 1H), 7.89 (d br, 2H), 7.74 (d br, 2H), 7.40-7.24 (m, 6H), 4.30 (m, 3H), 3.98 (s br, 3H), 3.37 (s br, 3H) . **^{13}C NMR (126 MHz, $CDCl_3$):** δ (ppm) = 168.7, 164.9, 155.1, 152.8, 146.8, 143.4, 141.4, 138.4, 128.0, 127.1, 126.4, 124.8, 120.2, 67.7, 53.1, 47.0, 36.9. **HRMS** (ESI+) *m/z* calcd. for $C_{24}H_{21}N_3O_5$: 432.1913 $[M+H]^+$; found: 432.1910.

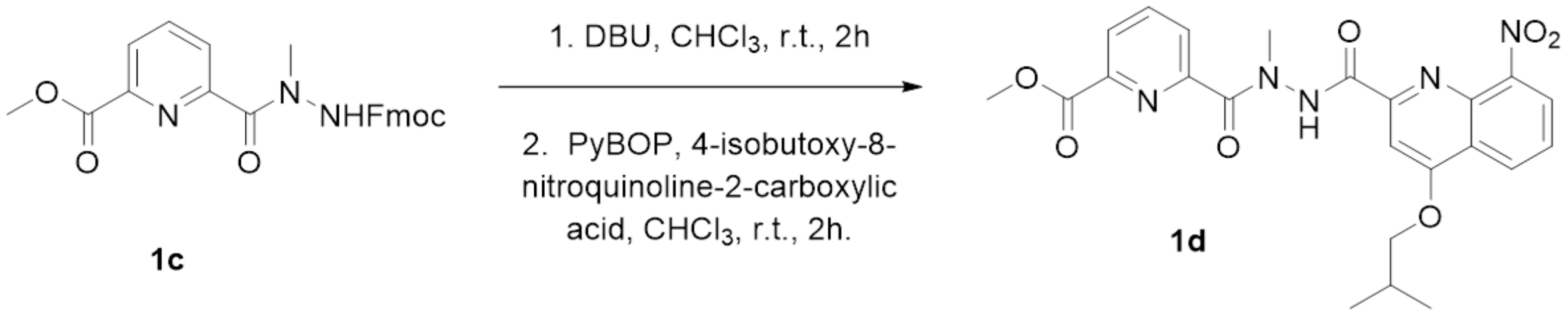

6-(2-(((9*H*-fluoren-9-yl)methoxy)carbonyl)-1-methylhydrazine-1-carbonyl)picolinic acid (1d): methyl 6-(2-(((9*H*-fluoren-9-yl)methoxy)carbonyl)-1-methylhydrazine-1-carbonyl)picolinate **1c** (130 mg, 0.3 mmol, 1.0 eq) and DBU (134 µL, 0.9 mmol, 3.0 eq) were dissolved in $CHCl_3$ (3 mL) at r.t.. After completion monitored by TLC, 4-isobutoxy-8-nitroquinoline-2-carboxylic acid (87 mg,

0.3 mmol, 1.0 eq), PyBOP (87 mg, 0.9 mmol, 3.0 eq) were added to the above mixture, and the reaction mixture was stirred at r.t. for 2h. The product was purified by column chromatography (100% EtOAc) to give the white solid (125 mg, 87%). **^{1}H NMR (500 MHz, $CDCl_3$):** δ (ppm) = 10.21 (s, 1H), 8.42 (dd, *J* = 8.4, 1.4 Hz, 1H), 8.05 (m, 2H), 7.90 (dd, *J* = 7.8, 1.4 Hz, 1H), 7.85 (t, *J* = 7.8 Hz, 1H), 7.62 (dd, *J* = 8.4, 7.5 Hz, 1H), 7.52 (s, 1H), 4.02 – 3.98 (m, 5H), 3.48 (s, 3H), 2.25 (m) 6.6 Hz, 1H), 1.09 (d, *J* = 6.6 Hz, 6H). **^{13}C NMR (126 MHz, $CDCl_3$):** δ (ppm) = 169.9, 165.4, 163.3, 162.1, 153.1, 151.2, 148.02, 146.7, 139.1, 138.2, 126.7, 126.5, 126.2, 126.0, 125.1, 123.6, 100.1, 76.0, 53.4, 36.5, 28.2, 19.3. **HRMS** (ESI+) *m/z* calcd. for $C_{23}H_{23}N_5O_7$: 482.1670 $[M+H]^+$; found: 482.1675.

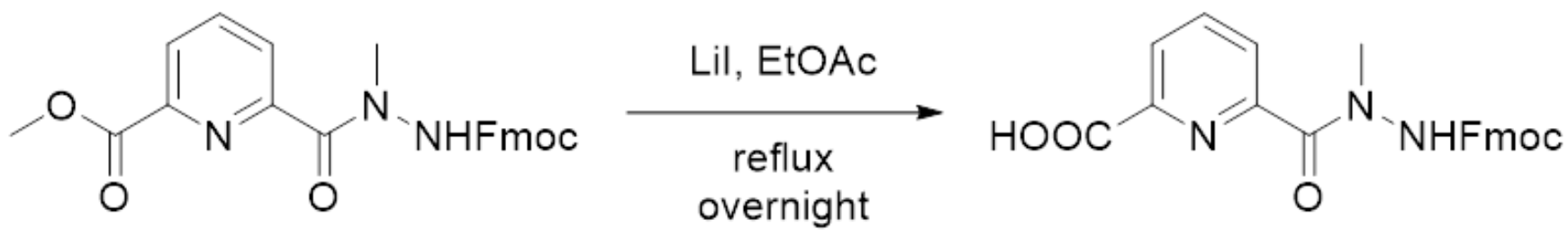

1c **1**

6-(2-(((9*H*-fluoren-9-yl)methoxy)carbonyl)-1-methylhydrazine-1-carbonyl)picolinic acid (1): methyl 6-(2-(((9H-fluoren-9-yl)methoxy)carbonyl)-1-methylhydrazine-1-carbonyl)picolinate **1c** (3.2 g, 7.4 mmol, 1.0 eq) and LiI (7.95 g, 59 mmol, 8.0 eq) were dissolved in degassed EtOAc (85 mL) and heated to reflux under nitrogen. After completion monitored by TLC, the mixture was cooled to r.t., quenched with water, and acidified with 5% aqueous citric acid. The product was extracted with DCM (3 × 50 mL), and the combined organic layers were washed with brine, dried over Na_2SO_4, filtered, and concentrated under reduced pressure to give the white solid (2.38 g, 77%). **^{1}H NMR (500 MHz, CD_3OD):** δ (ppm) = 8.13 (d br, 1H), 7.89 (d br, 1H), 7.74 (d br, 2H), 7.68 (s br, 1H),7.40-7.24 (m, 6H), 4.30 (d br, 2H), 3.98 (dd br, 1H), 3.37 (s br, 3H). **^{13}C NMR (126 MHz, CD_3OD):** δ (ppm) = 172.3, 170.2, 157.3, 152.8, 152.3, 144.7, 142.5, 139.5, 128.8, 128.2, 126.9, 126.2, 125.9, 120.9, 68.4, 57.5, 48.1, 36.9. **HRMS** (ESI+) *m/z* calcd. for $C_{23}H_{19}N_3O_5$: 418.1397 $[M+H]^+$; found: 418.1391.

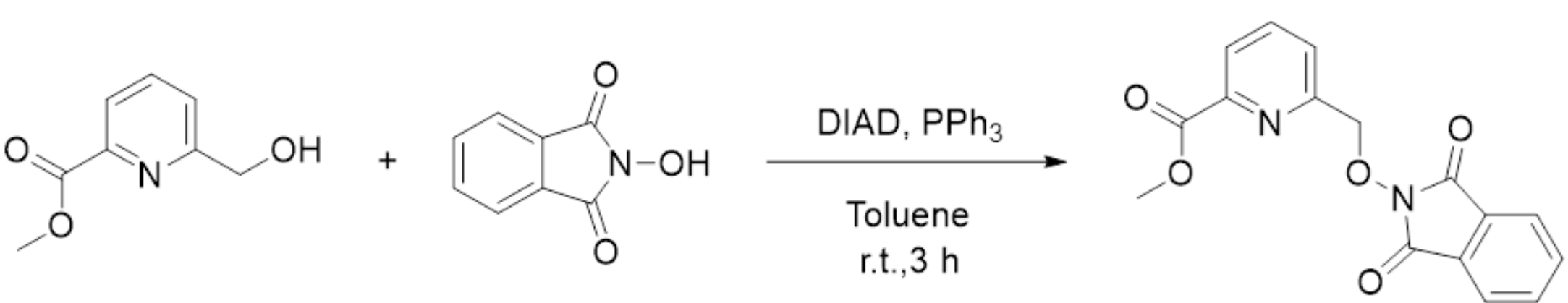

2a

Methyl 6-(((1,3-dioxoisoindolin-2-yl)oxy)methyl)picolinate (2a): To a solution of methyl 6-(hydroxymethyl)picolinate (1.5 g, 9 mmol, 1.0 eq), PPh_3 (3.0 g, 11.7 mmol, 1.3 eq), and *N*-hydroxyphthalimide (1.61 g, 9.9 mmol, 1.1 eq) in anhydrous toluene (90 mL), DIAD (2.3 mL, 11.7 mmol, 1.3 eq) was added dropwise at r.t.. The reaction mixture was stirred for 3 h, then

concentrated under reduced pressure. The resulting oil was covered with Et_2O and left overnight to give a white precipitate, which was collected by filtration (2.7 g, 95%). **^{1}H NMR (500 MHz, $CDCl_3$):** δ (ppm) = 8.12 (d, *J* = 7.7 Hz, 1H), 8.07 (d, *J* = 7.7 Hz, 1H), 7.96 (dd, *J* = *J* =7.7 Hz, 1H), 7.82 (m, 2H), 7.75 (m, 2H), 5.45 (s, 2H), 3.95 (s, 3H). **^{13}C NMR (126 MHz, $CDCl_3$):** δ (ppm) = 165.6, 163.5, 155.3, 147.5, 138.0, 134.7, 129.0, 126.8, 125.2, 123.8, 80.1, 53.1. **HRMS** (ESI+) *m/z* calcd. for $C_{16}H_{12}N_2O_5$: 313.0819 $[M+H]^+$; found: 313.0813.

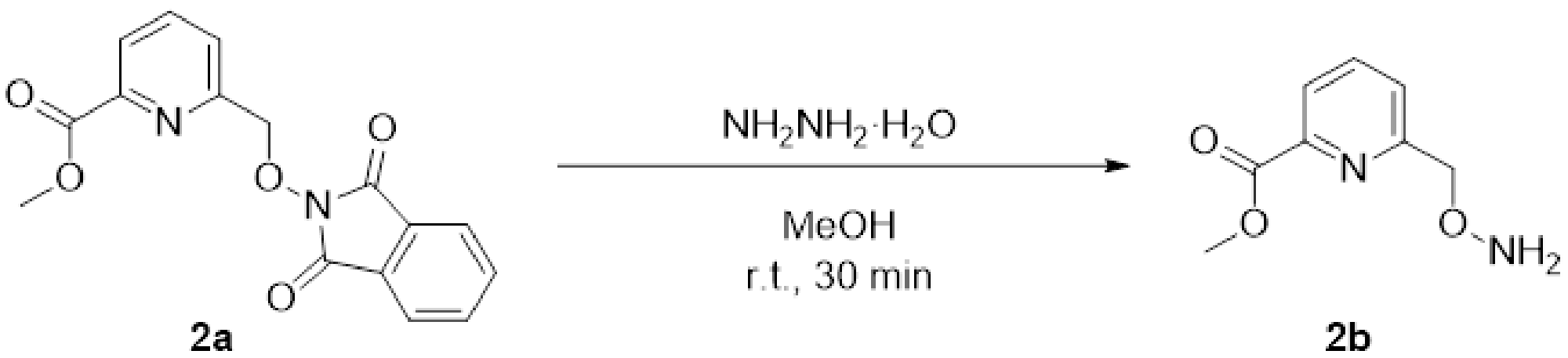

2a **2b**

Methyl 6-((aminooxy)methyl)picolinate (2b): methyl 6-(((1,3-dioxoisoindolin-2-yl)oxy)methyl)picolinate **2a** (936 mg, 3 mmol, 1.0 eq) was suspended in MeOH (9 mL), and monohydrate (150 mL, 3 mmol, 1.0 eq) was added to the above solution in one portion. The reaction was monitored by TLC. After the reaction was complete, the precipitate was filtered off and the remaining solution was purified by column chromatography with EtOAc, yielding a transparent oil. **^{1}H NMR (500 MHz, $CDCl_3$):** δ (ppm) = 8.06 (dd, *J* = 7.8, 1.1 Hz, 1H), 7.86 (dd, *J* = *J* = 7.8 Hz, 1H), 7.61 (dd, *J* = 7.8, 1.1 Hz, 1H), 5.63 (s br, 2H), 4.92 (s, 2H), 4.00 (s, 3H). **^{13}C NMR (126 MHz, $CDCl_3$):** δ (ppm) = 165.9, 158.9, 147.8, 137.7, 125.1, 124.2, 78.2, 53.1. **HRMS** (EI+) *m/z* calcd. for $C_8H_{10}N_2O_3$: 182.0685 $[M]^{\cdot+}$; found: 182.0680.[9]

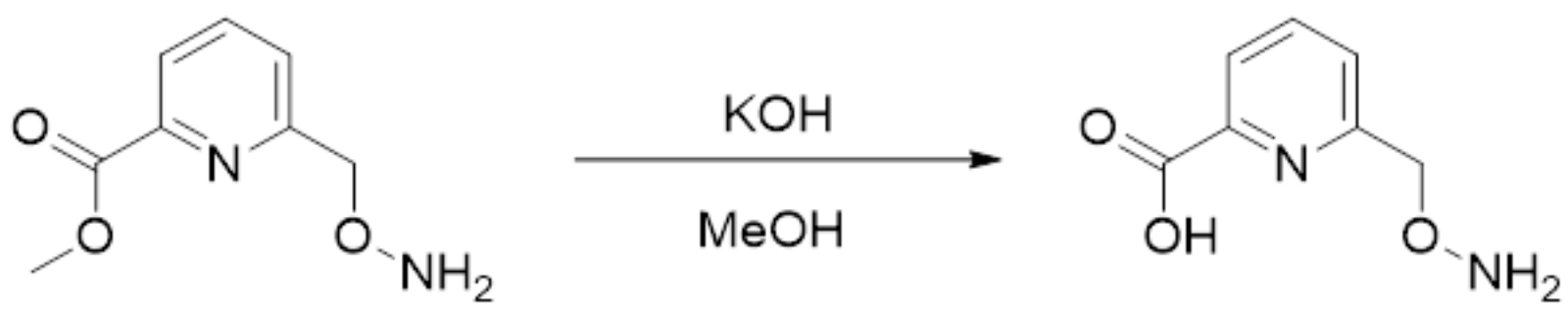

2b **2c**

6-((aminooxy)methyl)picolinic acid (2c): methyl 6-((aminooxy)methyl)picolinate **2b** (546 mg, 3 mmol, 1.0 eq) was dissolved in 11 mL of MeOH, and a solution of KOH (168 mg, 3 mmol, 1.0 eq) in water (1 mL) was added. The reaction mixture was stirred at 0 °C and the reaction was monitored by TLC. Upon completion, the solvent was removed under reduced pressure. The residue was acidified with 1M citric acid to pH 3 and used directly in the next step without further purification. **^{1}H NMR (500 MHz, DMSO-*d_6*):** δ (ppm) = 7.98 (dd, *J* = *J* =7.7 Hz, 1H), 7.93 (dd, *J* = 7.7, 1.3 Hz, 1H), 7.65 (dd, *J* = 7.7, 1.3 Hz, 1H), 4.71 (s, 2H). **^{13}C NMR (126 MHz, DMSO- *d_6*):** δ (ppm) = 166.2, 159.0,

147.7, 137.8, 124.6, 123.2, 77.2. **HRMS** (EI+) *m/z* calcd. for $C_7H_8N_2O_3$: 168.0529 $[M]^{\cdot+}$; found: 168.0523

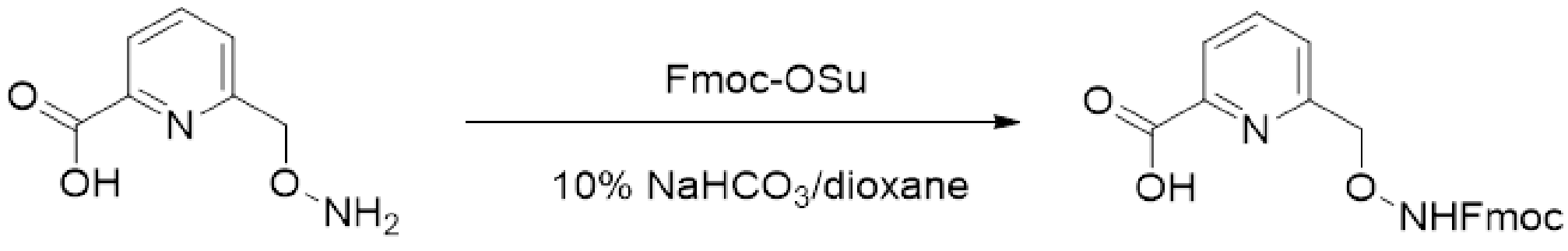

2c **2**

6-((((((9*H*-fluoren-9-yl)methoxy)carbonyl)amino)oxy)methyl)picolinic acid (2): 6-((aminooxy) methyl)picolinic acid **2c** (504 mg, 3 mmol, 1.0 eq) was suspended in dioxane (6 mL), and 10% $NaHCO_3$ solution (6 mL) was added. After cooling to 0 °C, Fmoc-*O*Su (1.11 g, 3.3 mmol, 1.1 eq) was added and the mixture stirred overnight. The reaction mixture was acidified to pH 3 with saturated $KHSO_4$, then extracted with DCM (3 × 100 mL) and dried over Na_2SO_4. The crude product was precipitated from acetonitrile (10 mL), sonicated, filtered, and washed with cold acetonitrile (−14 °C). Additional quantities of product were obtained from the filtrate after precipitation and freeze-drying, yielding the title compound as a white solid (1.07 g, 84%). **^{1}H NMR (500 MHz, DMSO-*d$_6$*):** δ (ppm) = 13.22 (s, 1H), 10.62 (s, 1H), 8.02 – 7.95 (m, 2H), 7.88 (d, *J* = 7.5 Hz, 2H), 7.66 (d, *J* = 7.5 Hz, 2H), 7.62 (s br, 1H), 7.40 (t, *J* = 7.4 Hz, 2H), 7.32 (t, *J* = 7.4, Hz, 2H), 4.85 (s, 2H), 4.45 (d, *J* = 6.6 Hz, 2H), 4.25 (t, *J* = 6.6 Hz, 1H). **^{13}C NMR (126 MHz, DMSO-*d$_6$*):** δ (ppm) = 166.0, 156.8, 156.3, 147.9, 143.6, 140.8, 138.1, 127.7, 127.1, 125.7, 125.1, 124.0, 120.2, 77.7, 65.8, 46.6. **HRMS** (ESI+) *m/z* calcd. for $C_{22}H_{18}N_2O_5$: 391.1289 $[M+H]^+$; found: 391.1286.

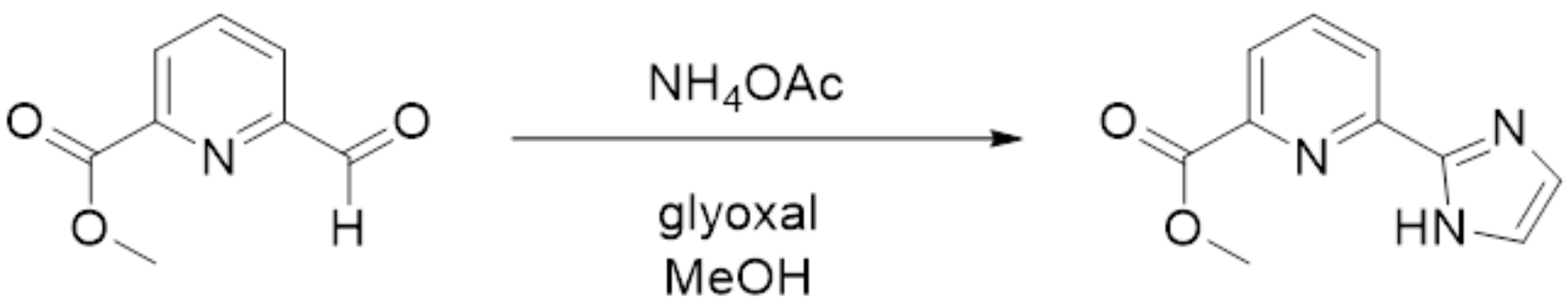

3a

Methyl 6-(1*H*-imidazol-2-yl)picolinate (3a): Methyl 6-formyl-2-pyridinecarboxylate (2.0 g, 12 mmol, 1.0 eq) and NH_4OAC (4.67 g, 60 mmol, 5.0 eq) were dissolved in MeOH (120 mL). The reaction mixture was stirred at 50°C for 30 min, then 40% glyoxal (1.41 mL, 12 mmol, 1.0 eq) was added, and stirring continued for 2 h. MeOH was removed under reduced pressure, and the crude product was purified by silica column chromatography (DCM/MeOH = 4:1), yielding a grey solid (1.5 g, 62%). **^{1}H NMR (500 MHz, $CDCl_3$):** δ (ppm) = 10.91 (s, 1H), 8.33 (dd, *J* = 7.9, 1.1 Hz, 1H), 8.02 (dd, *J* = 7.9, 1.1 Hz, 1H), 7.90 (dd, *J* = *J* =7.9 Hz, 1H), 7.25 (s, 1H), 7.15 (s, 1H), 3.98 (s, 3H). **^{13}C NMR (126 MHz, $CDCl_3$)** δ (ppm) = 165.6, 149.0, 147.2, 145.7, 138.2, 130.8, 124.4, 123.2, 117.9, 52.9. **HRMS** (EI+) *m/z* calcd.

for $C_{10}H_9N_3O_2$: 203.0689 [M]$^{\cdot+}$; found: 203.0686.

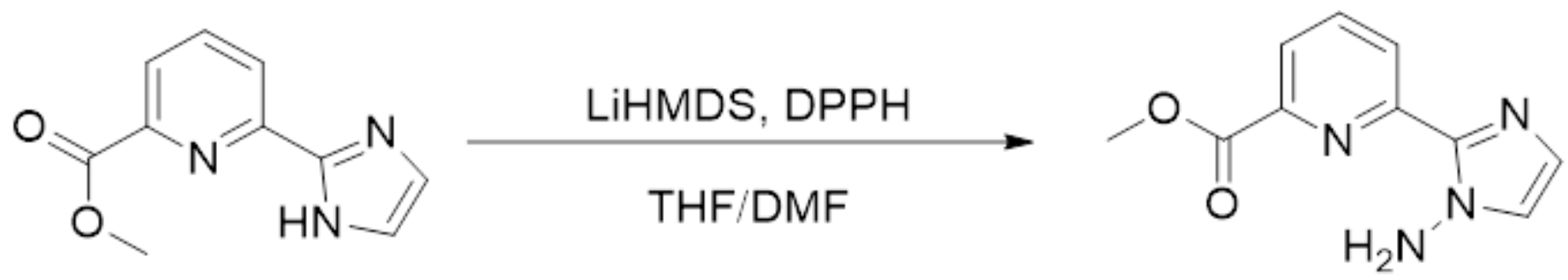

3a 3b

Methyl 6-(1-amino-1*H*-imidazol-2-yl)picolinate (3b): Lithium hexamethyldisilazide (LiHMDS, 6 mL, 6 mmol, 1.2 eq, 1 M in THF) was added dropwise to methyl 6-(1H-imidazol-2-yl)picolinate **3a** (1.0 g, 5 mmol, 1.0 eq) dissolved in dry DMF (25 mL) at -10 °C, forming a yellow solution. After stirring for 10 min, *O*-(diphenylphosphinyl) (DPPH, 2.15 g, 10 mmol, 2.0 eq) was added at 0 °C, and the mixture was stirred at r.t. for 2 h. The reaction was quenched with water at 0 °C, and the solvent was removed under reduced pressure. The residue was washed with EtOAc (3 × 50 mL), and then combined organic phases were dried, concentrated, and purified by silica gel chromatography (DCM/MeOH = 80:1 to 40:1) to afford the product as a white solid (860 mg, 80%). **^{1}H NMR (500 MHz, $CDCl_3$):** δ (ppm) = 8.36 (dd, *J* = 8.0, 1.1 Hz, 1H), 8.04 (dd, *J* = 8.0, 1.1 Hz, 1H), 7.93 (dd, *J* = *J* =8.0 Hz, 1H), 7.17 (d, *J* = 1.1 Hz, 1H), 7.07 (d, *J* = 1.1 Hz, 1H), 6.67 (s, 2H), 4.00 (s, 3H). **^{13}C NMR (126 MHz, $CDCl_3$):** δ (ppm) = 165.2, 150.6, 146.0, 139.6, 138.3, 126.7, 124.7, 123.7, 123.5, 53.0. **HRMS** (EI+) *m/z* calcd. for $C_{10}H_{10}N_4O_2$: 218.0798 [M]$^{\cdot+}$; found: 218.0804

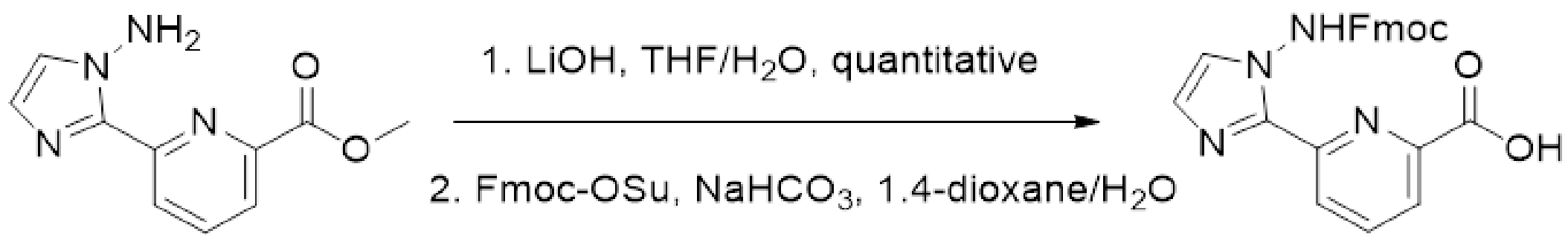

3b 3

6-(1-((((9*H*-fluoren-9-yl)methoxy)carbonyl)amino)-1*H*-imidazol-2-yl)picolinic acid (3): methyl 6-(1-amino-1*H*-imidazol-2-yl)picolinate **3b** (1.05 g, 5 mmol, 1.0 equiv.) was dissolved in THF (80 mL). After the addition of LiOH (252 mg, 6 mmol, 1.2 equiv.) in H_2O (20 mL), the solution was stirred for 30 min at r.t.. Then, the mixture was acidified to pH 2 using 1 M citric acid. The solvents were removed under reduced pressure, and the resulting solid (1.02 g, 5.5 mmol, 1.0 equiv.) was dissolved in dioxane (97 mL). $NaHCO_3$ (9.7 g) was dissolved in H_2O (97 mL). The reaction solution was cooled to 0°C. Fmoc-Osu (1.85 g, 5.5 mmol, 1.0 equiv.) was dissolved in dioxane (47 mL) and added to the above solution. The organic solvent was removed under reduced pressure, and the remaining aqueous phase was acidified with 5% citric acid to pH 3-4, then extracted with DCM, which was dried over Na_2SO_4. After the removal of the solvent under reduced pressure, the crude product was

precipitated from acetonitrile (10 mL), yielding the title compound as a white solid (1.61 g, 68%). **^{1}H NMR (500 MHz, DMSO-*d$_6$*):** δ (ppm) = 12.99 (s, 1H), 11.08 (s, 1H), 8.23 (d, *J* = 7.7 Hz, 1H), 8.07 (d, *J* = 7.7 Hz, 2H), 7.88 (s, 2H), 7.68 (s, 1H), 7.53 – 7.20 (m, 5H), 7.11 (s, 1H), 4.34 (s, 3H). **^{13}C NMR (126 MHz, DMSO-*d$_6$*):** δ (ppm) = 165.7, 148.5, 147.5, 143.5, 142.0, 140.7, 138.5, 127.7, 127.1, 126.5, 125.7, 125.2, 124.0, 120.1, 66.9, 46.4. **HRMS** (ESI+) *m/z* calcd. for $C_{24}H_{18}N_4O_4$: 427.1401 $[M+H]^+$; found:427.1393

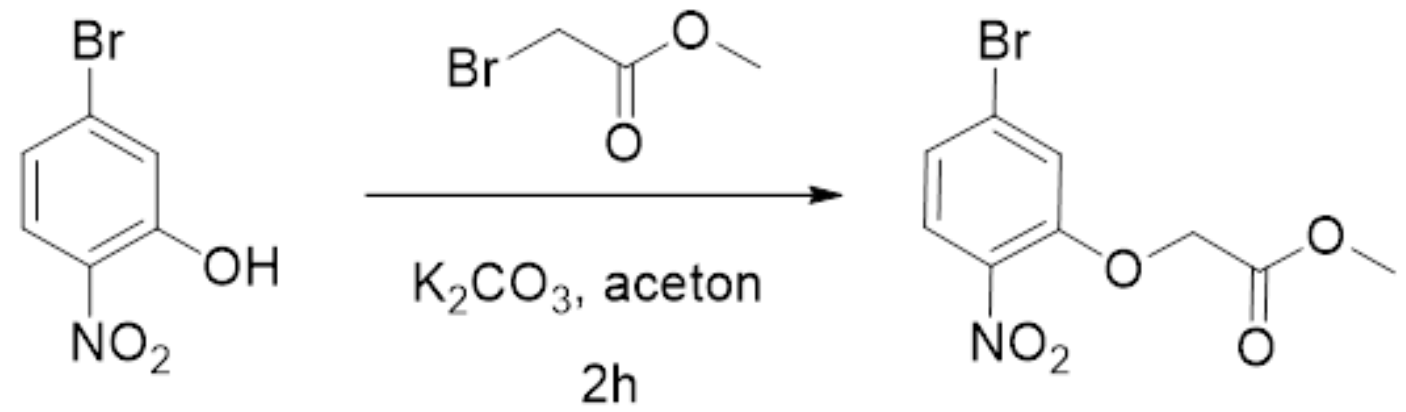

4a

Methyl 2-(5-bromo-2-nitrophenoxy)acetate (4a): 5-Bromo-2-nitrophenol (4.98 g, 23 mmol, 1.0 eq), methyl bromoacetate (2.39 mL, 25.3 mmol, 1.1 eq) and K_2CO_3 (3.50 g, 25.3 mmol, 1.1 eq) were suspended in dry acetone (90 mL). The reaction mixture was then refluxed for 2 h and filtered. The solvent was evaporated under reduced pressure and the resulting residue was extracted with water and DCM (2 x 50 mL). The compound was obtained as a light-yellow solid (12.8 g, 96%). **^{1}H NMR (500 MHz, $CDCl_3$):** δ (ppm) = 7.78 (d, *J* = 8.7 Hz, 1H), 7.26 (dd, *J* = 8.7, 1.9 Hz, 1H), 7.14 (d, *J* = 1.9 Hz, 1H), 4.79 (s, 2H), 3.83 (s, 3H). **^{13}C NMR (126 MHz, $CDCl_3$):** δ (ppm) = 167.8, 151.9, 139.4, 128.4, 127.2, 125.3, 118.9, 66.7, 52.8. **HRMS** (ESI+) *m/z* calcd. for $C_9H_8BrNO_5$: 289.9659 $[M+H]^+$; found: 289.9654.

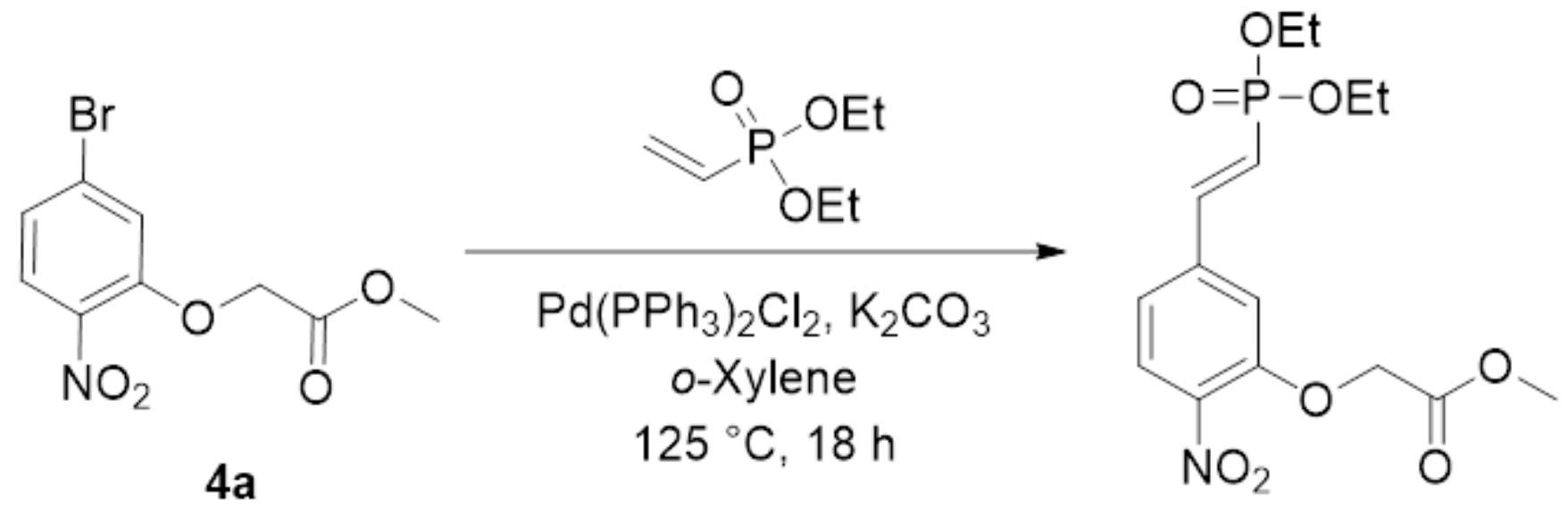

4b

Methyl (*E*)-2-(5-(2-(diethoxyphosphoryl)vinyl)-2-nitrophenoxy)acetate (4b): In a dry flask, methyl 2-(5-bromo-2-nitrophenoxy)acetate **4a** (5.8 g, 20 mmol, 1.0 eq) and K_2CO_3 (2.76 g, 20 mmol, 1.0 eq) were suspended in dry *o*-xylene (72 mL) under N_2. Diethyl vinylphosphonate (3.68 mL, 1.2eq) and $Pd(PPh_3)_2Cl_2$ (421 mg,0.6 mmol, 0.03 eq) were added, and the reaction mixture was stirred at 125°C for 20 h. The solution was diluted with EtOAc (70 mL) and washed by 5% citric acid (3 x 50 mL). The

organic phase was then washed by brine (80 mL) and dried over Na_2SO_4. The solvent was removed by vacuum and the crude was purified by RP-chromatography (from 15% acetonitrile to 100% acetonitrile) to give the target compound (6 g, 84%) as white solid. **^{1}H NMR (500 MHz, DMSO-*d₆*):** δ (ppm) = 7.91 (d, *J* = 8.4 Hz, 1H), 7.70 (s, 1H), 7.48 (d, *J* = 8.4 Hz, 1H), 7.40 (dd, *J* = 22.6, 17.3 Hz, 1H), 6.88 (dd, *J* = *J* =17.3 Hz, 1H), 5.09 (s, 2H), 4.11 – 3.97 (m, 4H), 3.71 (s, 3H), 1.27 (t, *J* = 7.1 Hz, 6H). **^{13}C NMR (126 MHz, DMSO-*d₆*):** δ (ppm)=168.3, 150.5, 145.1 (*J* = 6.8 Hz), 140.3 (*J* = 24.0 Hz), 140.0, 125.4, 120.8, 120.5 (*J* = 184Hz), 114.3, 65.48, 61.5 (*J* = 5.5 Hz), 52.0, 16.2 (*J* = 5.5 Hz). **HRMS** (ESI+) *m/z* calcd. for $C_{15}H_{20}NO_8P$: 374.1000 $[M+H]^+$; found: 374.1064.

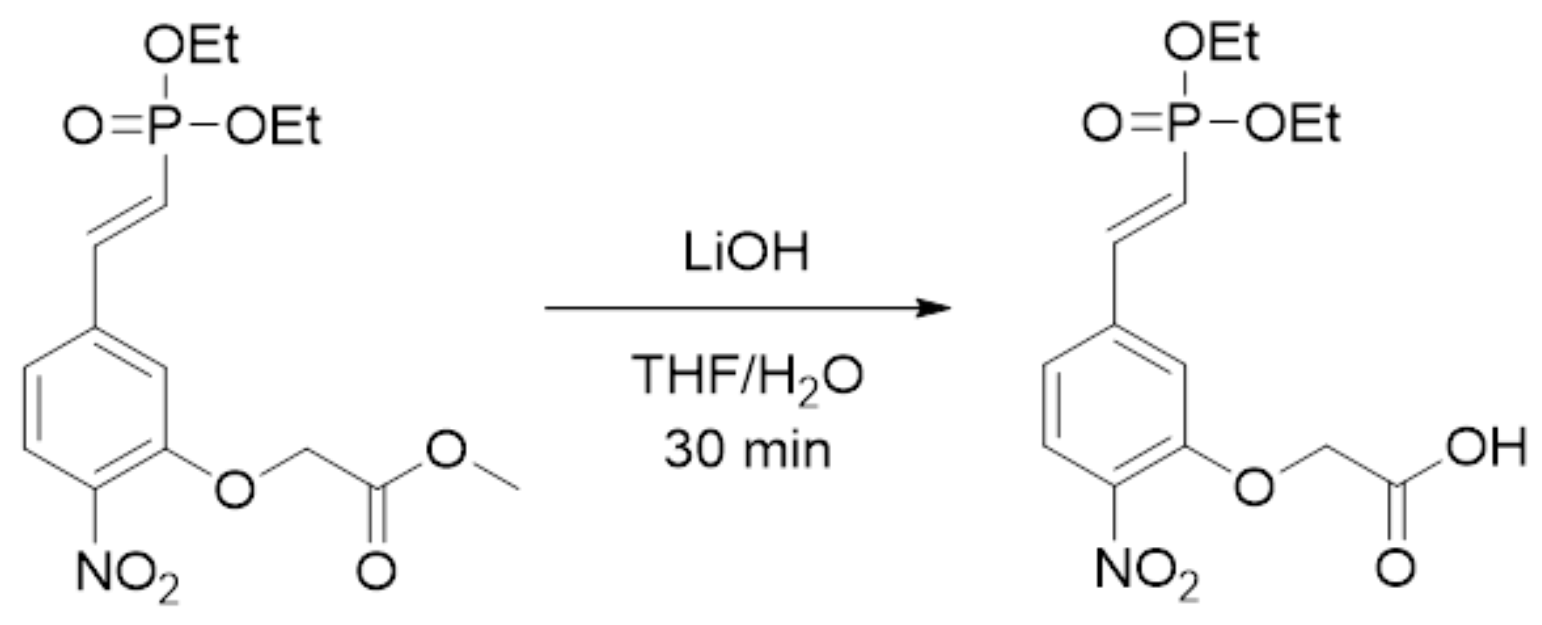

4b **4c**

(*E*)-2-(5-(2-(diethoxyphosphoryl)vinyl)-2-nitrophenoxy)acetic acid (4c): methyl (*E*)-2-(5-(2-(diethoxyphosphoryl)vinyl)-2-nitrophenoxy)acetate **4b** (8.2 g, 21.9 mmol, 1.0 equiv.) was dissolved in THF (320 mL). After the addition of 526 mg LiOH (21.9 mmol, 1.0 equiv.) in H_2O (80 mL), the solution was stirred for 30 min at r.t.. Then, the mixture was acidified to pH 2 using 1 M citric acid in H_2O. The resulting solution was extracted with DCM (3 × 50 mL)) and dried over $MgSO_4$. The solvents were removed under reduced pressure, yielding 6.0 g (quant.) of the title compound as a yellow solid that was used without further purification. **^{1}H NMR (500 MHz, $CDCl_3$):** δ (ppm)= 7.89 (d, *J* = 8.4 Hz, 1H), 7.53 (dd, *J* = 22.6, 17.3 Hz, 1H), 7.22 (dd, *J* = 8.4, 1.6 Hz, 1H), 7.10 (d, *J* = 1.6 Hz, 1H), 6.35 (dd, *J* = *J* =17.3 Hz, 1H), 4.84 (s, 2H), 4.21 – 4.11 (m, 4H), 1.36 (t, *J* = 7.1 Hz, 6H). **^{13}C NMR (126 MHz, $CDCl_3$):** δ (ppm) = 151.99, 146.93, 140.60 (*J* = 24.0 Hz), 140.34, 126.49, 119.72, 118.77 (*J* = 195 Hz), 117.24, 115.11, 66.08, 62.95 (*J* = 5.5 Hz), 16.47 (*J* = 5.5 Hz). **HRMS** (ESI+) *m/z* calcd. for $C_{14}H_{18}NO_8P$: 360.0843 $[M+H]^+$; found: 360.0846.

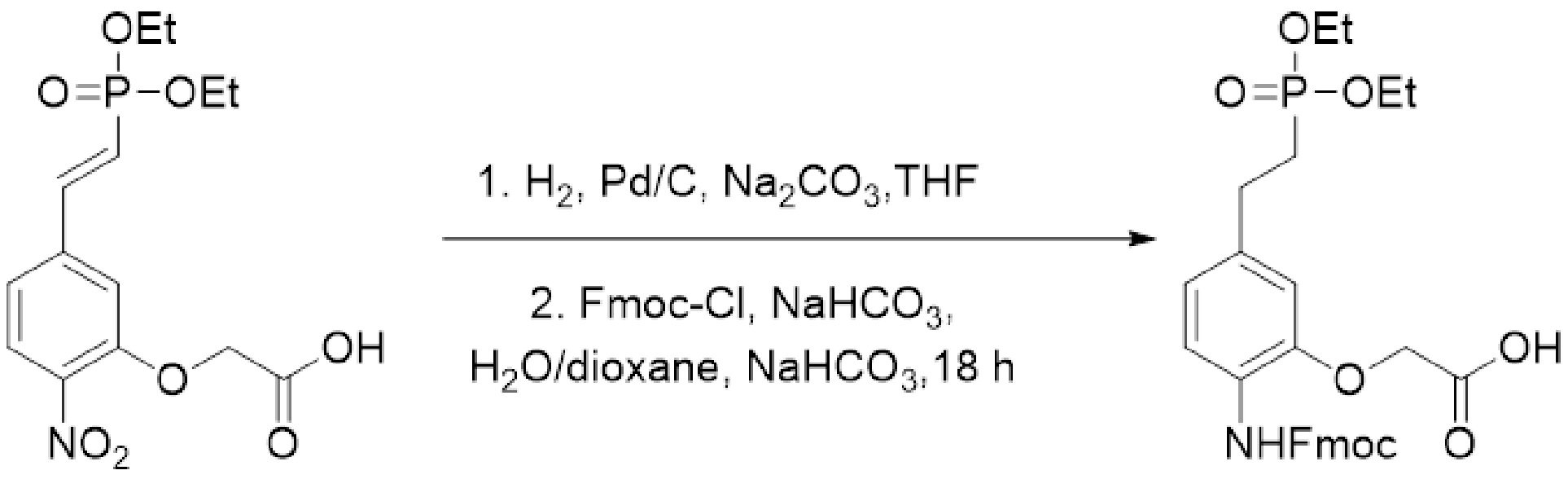

2-(2-((((9*H*-fluoren-9-yl)methoxy)carbonyl)amino)-5-(2(diethoxyphosphoryl)ethyl)phenoxy) acetic acid (4): (*E*)-2-(5-(2-(diethoxyphosphoryl)vinyl)-2-nitrophenoxy)acetic acid **4c** (2.5 g, 6.96 mmol, 1.0 equiv.) and Na_2CO_3 (738 mg, 6.96 mmol, 1.0 equiv.) were suspended in MeOH (250 mL). After the solution was degassed by vacuum N_2 cycles (3x), 250 mg Pd/C (10%w) were added and the N_2 was replaced by H_2. The reaction mixture was stirred at r.t. for 6 h, filtered over celite and washed with MeOH. Solvents were evaporated under reduced pressure to obtain the crude amine as a white solid that was used for next step without further purification. The crude amine (4.6 g, 13.92 mmol, 1.0 equiv.) and $NaHCO_3$ (24.5 g, 292.3 mmol, 21.0 equiv.) were dissolved in H_2O (255 mL). Then, Fmoc-Cl (3.6 g, 13.92 mmol, 1.0 equiv.) in dioxane (100 mL) was added dropwise at 0 °C over a period of 1 h. The reaction mixture was stirred at 0 °C for 1 h and then at r.t. for 18 h. After the mixture was acidified to approximately pH 2 using 1 M citric acid in H_2O, it was extracted with DCM (3 x 50 mL), dried over $MgSO_4$ and solvents were removed under reduced pressure. The solid was precipitated from acetonitrile as a white solid (6.3 g, 80%). **^{1}H NMR (500 MHz, DMSO-*d₆*):** δ (ppm)= 13.07 (s, 1H), 8.61 (s, 1H), 7.91 (d, *J* = 7.4 Hz, 2H), 7.74 (d, *J* = 7.4 Hz, 2H), 7.53 (s, 1H), 7.43 (t, *J* = 7.5 Hz, 2H), 7.34 (t, *J* = 7.5 Hz, 2H), 6.94 (d, *J* = 1.8 Hz, 1H), 6.82 (d, *J* = 8.2 Hz, 1H), 4.74 (s, 2H), 4.40 (d, *J* = 7.0 Hz, 2H), 4.30 (t, *J* = 7.0 Hz, 1H), 3.99 (s, 4H), 2.74 – 2.68 (m, 2H), 2.08 – 1.99 (m, 2H), 1.23 (t, *J* = 7.0 Hz, 6H). **^{13}C NMR (126 MHz, DMSO-d_6):** δ (ppm) = 170.5, 153.6, 143.8, 140.7, 127.7, 127.1, 125.8, 125.3, 121.0, 120.1, 113.5, 66.1, 61.0 (*J* = 5.5 Hz), 46.6, 27.8 (*J* = 4.3 Hz), 26.7 (*J* = 137 Hz), 16.3 (*J* = 5.5 Hz). **HRMS** (ESI+) *m/z* calcd. for $C_{29}H_{32}NO_8P$: 554.1938 $[M+H]^+$; found: 554.1949.

3.3 Oligomer synthesis procedures

Oligomers **5**-**20** were synthesized by recently reported automated solid phase foldamer synthesis (SPFS) procedures.[10] Fmoc acid building blocks were activated *in situ* by generating the respective acid chlorides prior to coupling. The biotinylation of foldamers **15**-**19** was performed according to the previously reported protocol.[11]

Acetylation: The resin (1.0 eq.) was washed with DCM (3 x 3 mL) and incubated in Ac_2O/DCM (1:1 v:/v) for 10 min. Then, the resin was washed with DCM (2 x 3 mL) and DMF (3 x 3 mL).

Resin cleavage and Preparative HPLC purification: The resin-bound oligomer was placed in a syringe equipped with a filter, washed with DMF (3 x 3 mL), DCM (3 x 3 mL), and dried by passing N_2 flow through it. It was then suspended in a solution of TFA. The resin was next shaken for at least 2 h at r.t. and then filtered off and washed one time with TFA. The combined solvent was removed in vacuo. After precipitation in cold Et_2O, the crude oligomer with protecting groups was purified by semi prep RP-HPLC under acidic condition to give the oligomer as a yellow solid.

Synthesis of water-soluble oligomers: The previously purified oligomer was treated by TMSBr to remove the ethyl groups. Subsequently, the crude was purified by semi prep RP-HPLC under basic conditions (as described in section 3.1) to give the oligomer as a yellow solid. Subsequently, an ion exchange process was performed to obtain the side chains as water-soluble ammonium phosphonate salts. The removal of ethyl phosphonate protecting groups and ion exchange were performed as previously described.[12]

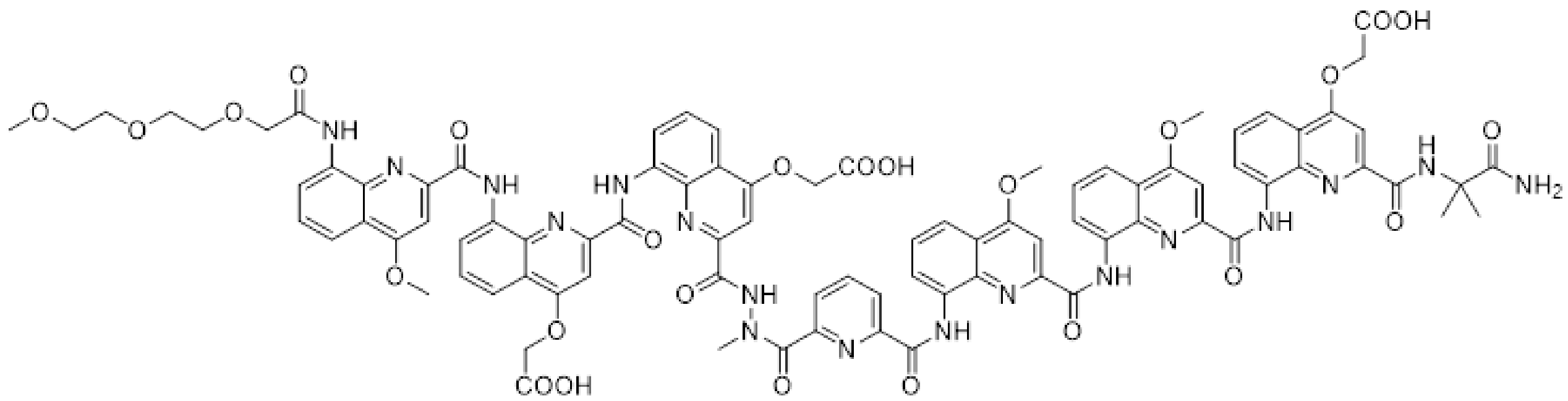

Tail-Q^AQ^DQ^DHQ^AQ^AQ^D-Aib-NH$_2$ (5): Oligomer **5** was synthesized by the automated SPFS.[10] The title compound was obtained as a white solid (15.2 mg, 44%).**^{1}H NMR (500 MHz, H_2O/D_2O 9:1):** δ (ppm) = 12.17 (s, 1H), 11.70 (s, 1H), 11.60 (s, 1H), 11.58 (s, 1H), 11.27 (s, 1H), 11.20 (s, 1H), 11.09 (s, 1H), 11.04 (s, 1H), 10.56 (s, 1H), 9.98 (s, 1H), 9.95 (s, 1H), 9.64 (s, 1H), 9.54 (s, 1H) 8.78 (d, *J* = 8.1 Hz, 1H), 8.73 (d, *J* = 8.1 Hz, 1H), 8.52 (d, *J* = 9.0 Hz, 1H), 8.48 (s, 1H), 8.42 (d, *J* = 8.0 Hz, 1H), 8.19 (d, *J* = 8.1 Hz, 1H), 8.16 (d, *J* = 8.9 Hz, 1H), 8.04-7.95 (m, 5H), 7.89 (d, *J* = 8.2 Hz, 1H), 7.84 – 7.61 (m, 8H), 7.59 (d, *J* = 8.9 Hz, 1H), 7.54 – 7.46 (m, 2H), 7.42 (d, *J* = 8.8 Hz, 1H), 7.39 – 7.03 (m, 17H); 7.00 – 6.74 (m, 6H), 6.10 (s, 1H), 6.03 (s, 1H), 5.70 (s, 1H), 4.07 – 3.81 (m, 11H), 3.57 – 3.34 (m, 4H), 3.13 – 2.92 (m, 4H), 2.84 – 2.62 (m, 12H), 2.55 – 2.44 (m, 2H), 2.34 (s, 2H), 1.97 (s, 2H), 1.90 (s, 2H), 1.71 (s, 3H), 1.21 – 1.15 (m, 4H), 1.13 (s, 3H), 1.00 (s, 2H), 0.91 (s, 3H). **HRMS** (ESI$^-$) *m/z* calcd. for $C_{88}H_{78}N_{17}O_{25}$: 1771.5282 [M-H]$^-$; found: 1771.5308.

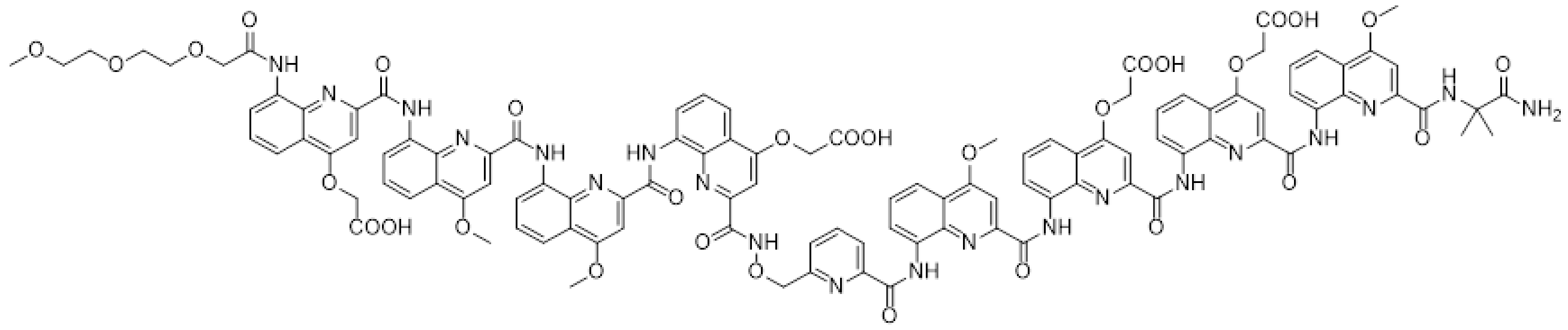

Tail-$Q^{D}Q^{A}Q^{A}Q^{D}OQ^{A}Q^{D}Q^{D}Q^{A}$-Aib-$NH_2$ (6): Oligomer **6** was synthesized by the automated SPFS.[10] The title compound was obtained as a white solid (30 mg, 45%). **^{1}H NMR (500 MHz, H_2O/D_2O 9:1):** δ (ppm) = 11.53 (s 1H), 11.48 (s 1H) 11.24 (s, 1H), 10.95 (s, 1H), 10.82 (s, 1H), 10.40 (s, 1H), 10.19 (s, 1H), 9.96 (s, 1H), 9.60 (s, 1H), 8.24 (d, *J* = 8.0 Hz, 1H), 8.06 – 7.99 (m, 1H), 7.89 (m, 4H), 7.74 (m, 3H), 7.55 – 7.15 (m, 16H), 7.14 – 7.05 (m, 3H), 6.94 (s, 1H) 6.79 (s, 1H), 6.64 (d, *J* = 8.2 Hz, 1H), 6.42 (s, 1H), 6.38 (s, 1H), 6.23 (s, 1H), 6.06 (s, 1H), 4.11-3.99 (m, 6H), 3.89 (s, 3H), 3.76 -3.73 (m, 2H), 3.57 - 3.54 (m, 2H), 3.41 -3.35 (m, 2H), 2.97 -2.93 (m, 1H), 2.80 -2.55 (m, 6H), 2.41 -2.21 (m, 4H), 2.66 (s, 3H), 2.56 (t, *J* = 4.8 Hz, 2H), 2.46 – 2.24 (m, 5H), 1.20 (s, 3H), 0.98 (s, 3H). **HRMS** (ESI^+) *m/z* calcd. for $C_{110}H_{92}N_{20}O_{31}$: 1095.3191 $[M+2H]^{2+}$; found: 1095.3201.

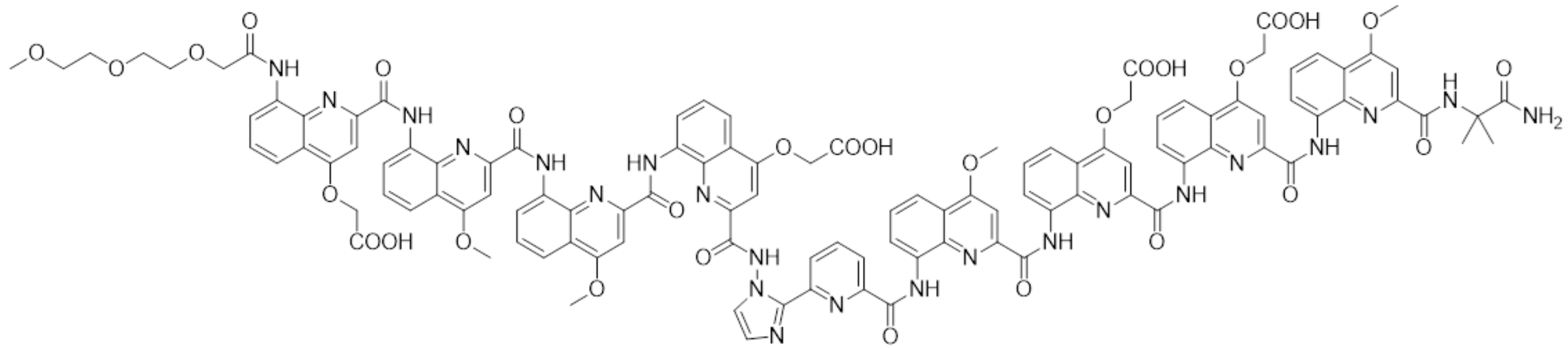

Tail-$Q^{D}Q^{A}Q^{A}Q^{D}NQ^{A}Q^{D}Q^{D}Q^{A}$-Aib-$NH_2$ (7): Oligomer **7** was synthesized by the automated SPFS.[10] The title compound was obtained as a white solid (26.8 mg, 40%). **^{1}H NMR (500 MHz, H_2O/D_2O 9:1):** δ (ppm) = 12.50 (s, 1H), 11.21 (s, 1H), 11.15 (s, 1H), 10.98 (s, 1H), 10.65 (s, 1H), 10.37 (s, 1H), 9.49 (s, 1H), 9.40 (s, 1H), 8.20 (d, *J* = 7.9 Hz, 1H), 7.83 (d, *J* = 8.8 Hz, 1H), 7.79 - 7.71 (m, 4H), 7.63 - 7.60 (m, 2H), 7.47 - 7.44 (m, 2H), 7.32 - 7.23 (m, 4H), 7.19 - 7.13 (m, 4H), 7.06 – 6.73 (m, 7H) 6.55 - 6.20 (m, 6H), 5.75 (s, 1H), 4.83 – 4.65 (m, 1H), 3.99 (s, 1H), 3.91 (s, 2H), 3.75 (s, 2H), 3.33-3.30 (m, 1H), 3.22 – 3.16 (m, 1H), 3.04-2.73 (m, 2H), 2.52 (s, 1H), 2.48 (s, 3H), 2.39 (t, *J* = 4.8 Hz, 2H), 2.23 – 2.12 (m, 2H), 2.11 – 2.05 (m, 1H), 1.67 – 1.60 (m, 1H), 1.19 – 1.07 (m, 2H), 0.98 (s, 3H), 0.78 (s, 3H), 0.70 (s, 1H). **HRMS** (ESI^-) *m/z* calcd. for $C_{112}H_{92}N_{22}O_{30}$: 2223.6277 $[M-H]^-$; found: 2223.6283.

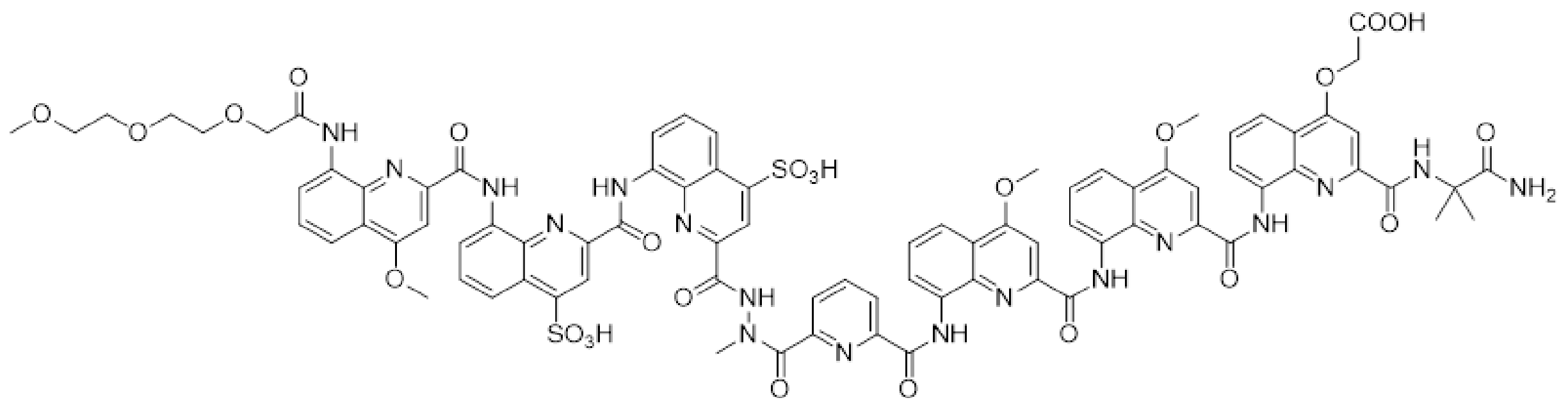

Tail-Q^A^Q^S^Q^S^HQ^A^Q^D^Q^D^-Aib-NH₂ (8): Oligomer **8** was synthesized by the automated SPFS.[10] The title compound was obtained as a white solid (27.2 mg, 50 %).**^{1}H NMR (500 MHz, H_2O/D_2O 9:1):**δ (ppm) = 12.27 (s, 1H), 11.73 (s, 1H), 11.66 (s, 1H), 11.65 (s, 1H), 11.14 (s, 1H), 11.04 (s, 1H), 10.94 (s, 1H), 10.78 (s, 1H), 10.05 (s, 1H), 9.99 (s, 1H), 9.67 (s, 1H), 9.64 (s, 1H), 9.02 (s, 1H), 8.88 (d, *J* = 9.1 Hz, 1H), 8.79 (d, *J* = 8.1 Hz, 1H), 8.67 (s, 1H), 8.56 (d, *J* = 8.0 Hz, 1H), 8.53 – 8.44 (m, 2H), 8.28 (t, *J* = 8.6 Hz, 1H), 8.14 (d, *J* = 8.3 Hz, 1H), 8.11 – 7.84 (m, 4H), 7.83 – 7.52 (m, 6H), 7.50 – 7.31 (m, 5H), 7.24 (s, 1H), 7.22 (s, 1H), 7.19 –6.86 (m, 7H), 6.43 (s, 1H), 6.35 (s, 1H), 6.19 (s, 2H), 6.13 (s, 1H), 4.14 (s, 1H), 4.05 (s, 1H), 4.01 (s, 2H), 3.94 (s, 2H), 3.89 (s, 1H), 3.70 – 3.44 (m, 4H), 3.11 – 2.92 (m, 2H), 2.88 (s, 3H), 2.84 (s, 2H), 2.39 (s, 2H), 2.06 (s, 1H), 1.28 (s, 2H), 1.22 (s, 3H), 1.10 (s, 2H), 1.02 (s, 3H). **HRMS** (ESI^-) *m/z* calcd. for $C_{84}H_{73}N_{17}O_{25}S_2$: 1782.4332$[M-H]^-$; found: 1782.4340.

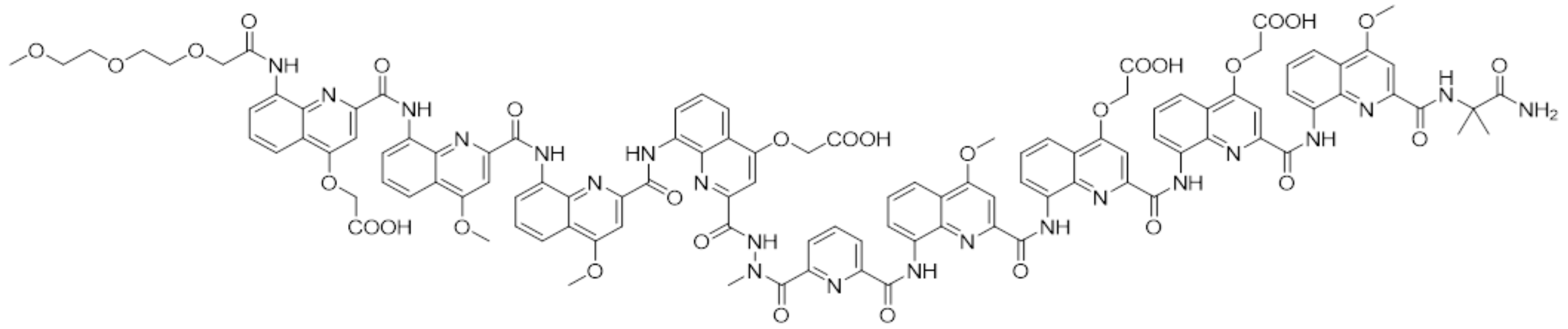

Tail-$Q^DQ^AQ^AQ^DHQ^AQ^DQ^DQ^A$-Aib-$NH_2$ (9): Oligomer **9** was synthesized by the automated SPFS.[10] The title compound was obtained as a white solid (16.2 mg, 45%). **^{1}H NMR (500 MHz, H_2O/D_2O 9:1):**δ (ppm) = 11.38 (s, 1H), 11.19 (s, 1H), 11.01 (s, 1H), 10.70 (s, 1H), 10.69 (s, 1H), 10.34 (s, 1H), 9.59 (s, 1H), 9.47 (s, 1H), 8.41 (d, *J* = 7.8 Hz, 1H), 8.03 – 7.85 (m, 4H), 7.80 – 7.53 (m, 9H), 7.46 – 7.13 (m, 10H), 7.12 – 7.05 (m, 1H), 6.99 – 6.88 (m, 3H), 6.85 – 6.61 (m, 7H) 6.48 (s, 1H), 6.37-6.31 (m, 2H), 5.43 (s, 1H), 4.04 (s, 2H), 3.97 – 3.72 (m, 11H), 3.61 – 3.30 (m, 3H), 3.10 – 2.81 (m, 2H), 2.78 – 2.67 (m, 2H), 2.62 (s, 1H), 2.57 (s, 3H), 2.49 (t, *J* = 4.7 Hz, 2H), 2.37 – 2.17 (m, 3H), 2.09 (s, 3H), 1.83 – 1.79 (m, 1H), 1.43 (s, 1H), 1.26 – 1.16 (m, 2H), 1.08 (s, 3H), 0.94 (s, 1H), 0.88 (s, 3H). **HRMS** (ESI^-) *m/z* calcd. for $C_{111}H_{93}N_{21}O_{31}$: 2214.6274 $[M-H]^-$; found: 2214.6203

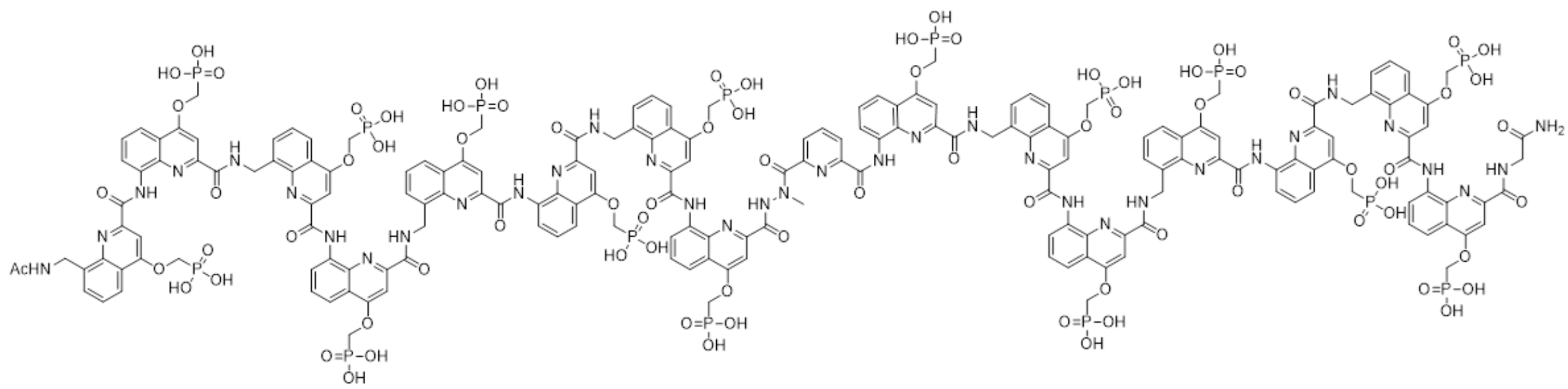

Ac-MQ⁴MQ⁴MQ⁴MQ⁴MQ⁴HQ⁴MQ⁴MQ⁴-Gly-NH₂ (10): Oligomer **10** was synthesized by the automated SPFS.[10] The title compound was obtained as a white solid (14.2 mg, 45%).**^{1}H NMR (500 MHz, H_2O/D_2O 9:1):**δ (ppm) = 11.15 (s, 1H), 10.94 (s, 1H), 10.14 (s, 1H), 10.04 – 9.93 (m, 3H), 9.64-9.53 (m, 3H), 9.33 (d, *J* = 9.7 Hz, 1H), 8.86 (d, *J* = 8.7 Hz, 1H), 8.74 (d, *J* = 6.7 Hz, 1H), 8.55 (d, *J* = 8.6 Hz, 1H), 8.45 (t, *J* = 8.7 Hz, 3H), 8.32 – 8.10 (m, 8H), 8.06 – 7.90 (m, 7H), 7.83 - 7.67 (m, 10H), 7.55 – 7.26 (m, 12H), 7.23 – 7.02 (m, 13H), 7.01 – 6.78 (m, 13H), 6.76 – 6.63 (m, 8H), 6.59 – 6.44 (m, 12H), 6.35 (s, 1H), 6.30 (s, 2H), 3.25 – 3.08 (m, 3H), 3.03 – 2.89 (m, 2H), 2.77 – 2.31 (m, 5H), 2.04 (s, 4H), 1.37 – 1.22 (m, 9H). **HRMS** (ESI^-) *m/z* calcd. for $C_{184}H_{164}N_{35}O_{79}P_{15}$: 1146.8916 $[M-4H]^{4-}$; found: 1146.9021.

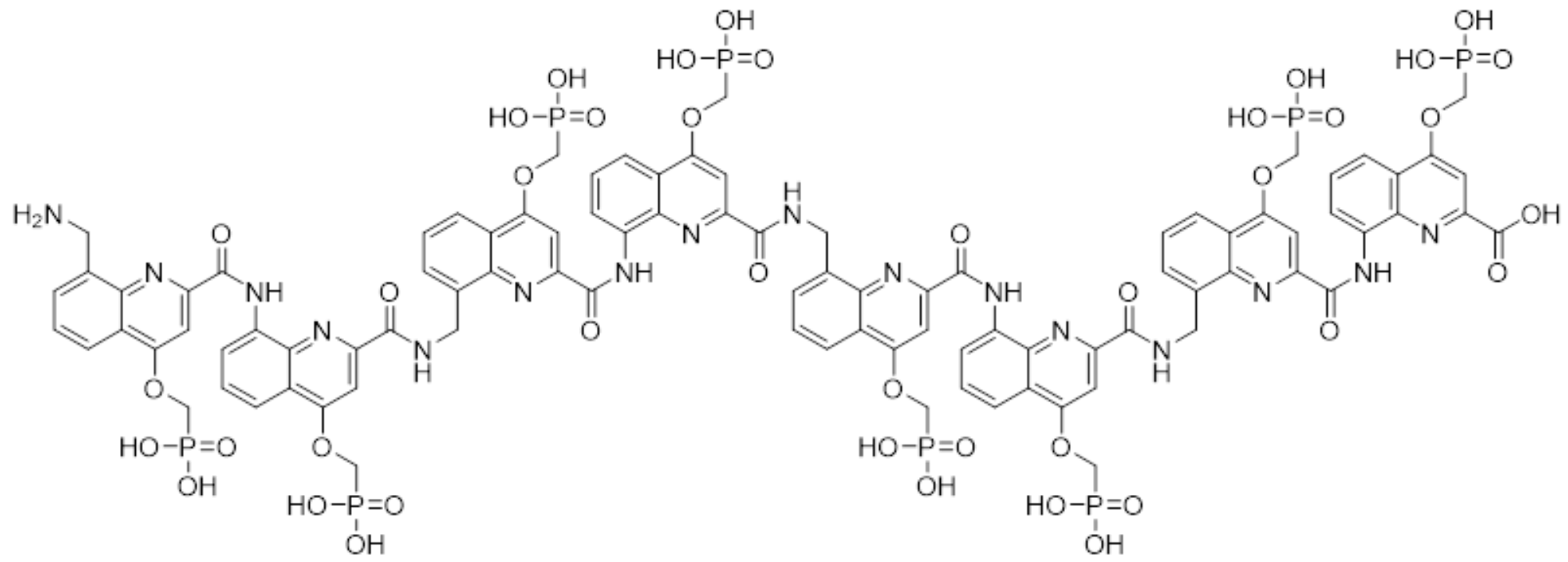

H-MQ⁴MQ⁴MQ⁴MQ⁴-OH (11): Oligomer **11** was synthesized by the automated SPFS.[10] The title compound was obtained as a white solid (12.4 mg, 35%).**^{1}H NMR (500 MHz, H_2O/D_2O 9:1):** δ (ppm) = 11.72 (s, 1H), 11.10 (s, 1H), 10.63 (s, 1H), 10.12 (s, 1H), 9.57 (d, *J* = 10.3 Hz, 1H), 8.88 (t, *J* = 6.9 Hz, 1H), 8.62 (d, *J* = 12.1 Hz, 1H), 8.33 (t, *J* = 8.5 Hz, 2H), 8.19 (d, *J* = 9.4 Hz, 1H), 8.05 (d, *J* = 9.4 Hz, 1H), 7.99 -7.94 (m, 2H), 7.77 - 7.70 (m, 2H), 7.67 – 7.60 (m, 1H), 7.56 – 7.49 (m, 3H), 7.48 – 7.41 (m, 2H), 7.35 – 7.24 (m, 2H), 7.18– 7.11 (m, 4H), 7.06 (t, *J* = 8.6 Hz, 1H), 6.96 – 6.85 (m, 4H); 6.70 (s, 1H), 6.55 (d, *J* = 7.9 Hz, 1H), 6.48 (s, 1H), 6.42 (s, 1H), 6.35 (d, *J* = 5.3 Hz, 2H), 6.10 (s, 1H), 4.14 – 3.97 (m, 1H), 3.86 – 3.48 (m, 9H), 3.35 – 3.27 (m, 1H), 2.98 – 2.93 (m, 1H), 2.45 – 2.37 (m, 2H), 1.84 (s, 3H), 1.76 (s, 2H). **HRMS** (ESI^-) *m/z* calcd. for $C_{92}H_{82}N_{16}O_{41}P_8$: 1156.1289 $[M-2H]^{2-}$; found: 1156.1331.

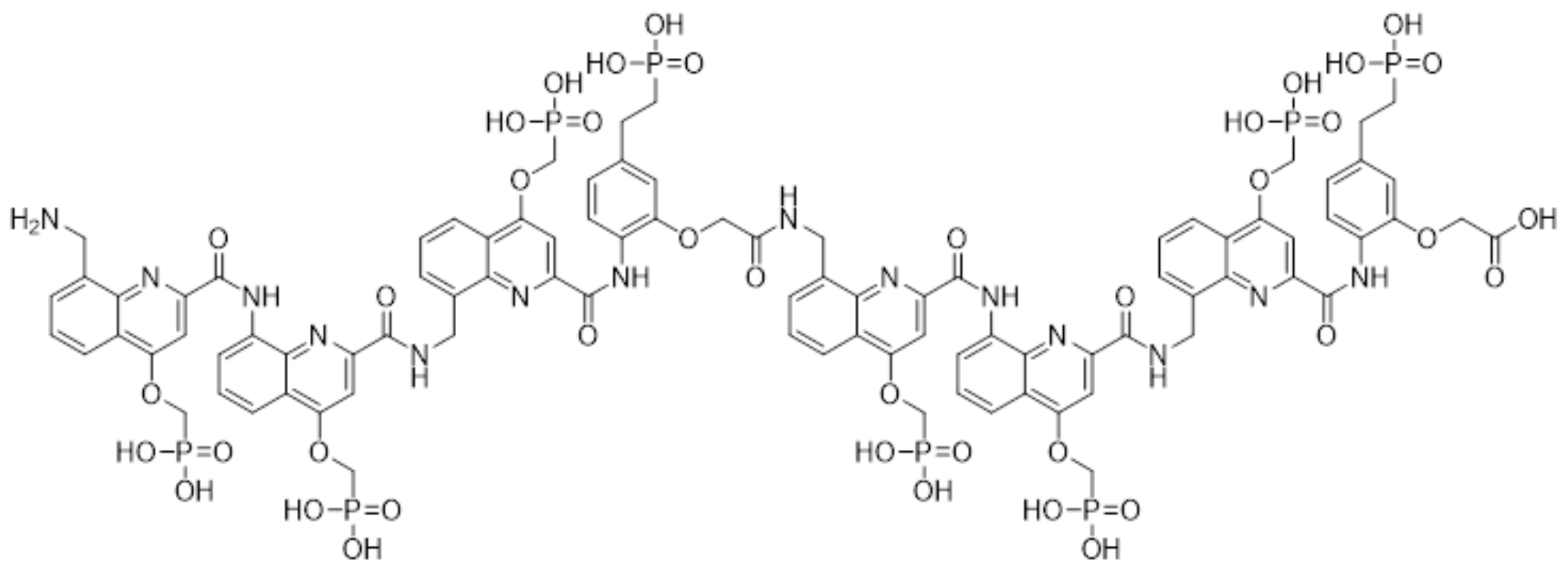

H-MQ⁴MBᴾMQ⁴MBᴾ-OH (12): Oligomer **12** was synthesized by the automated SPFS.[10] The title compound was obtained as a white solid (11.3 mg, 34%).**¹H NMR (500 MHz, H_2O/D_2O 9:1):**δ (ppm) = 11.19 (s, 1H), 11.09 (s, 1H), 10.24 (s, 1H), 9.62 (s, 1H), 9.37 (s, 1H), 9.07 (s, 1H), 8.41 (d, *J* = 9.3 Hz, 1H), 8.10 – 7.03 (m, 16H), 6.72 (d, *J* = 8.5 Hz, 1H), 6.50 (d, *J* = 9.7 Hz, 1H), 6.40 (s, 1H), 6.36 (s, 1H), 6.30 (s, 1H), 6.18 – 6.11 (m, 1H), 5.73 (s, 1H), 4.16 -3.35 (m, 7H), 3.01 - 2.23 (m, 5H), 1.55 – 1.46 (m, 3H). **HRMS** (ESI^-) *m/z* calcd. for $C_{90}H_{88}N_{14}O_{41}P_8$: 1133.1493 $[M-2H]^{2-}$; found: 1133.1504.

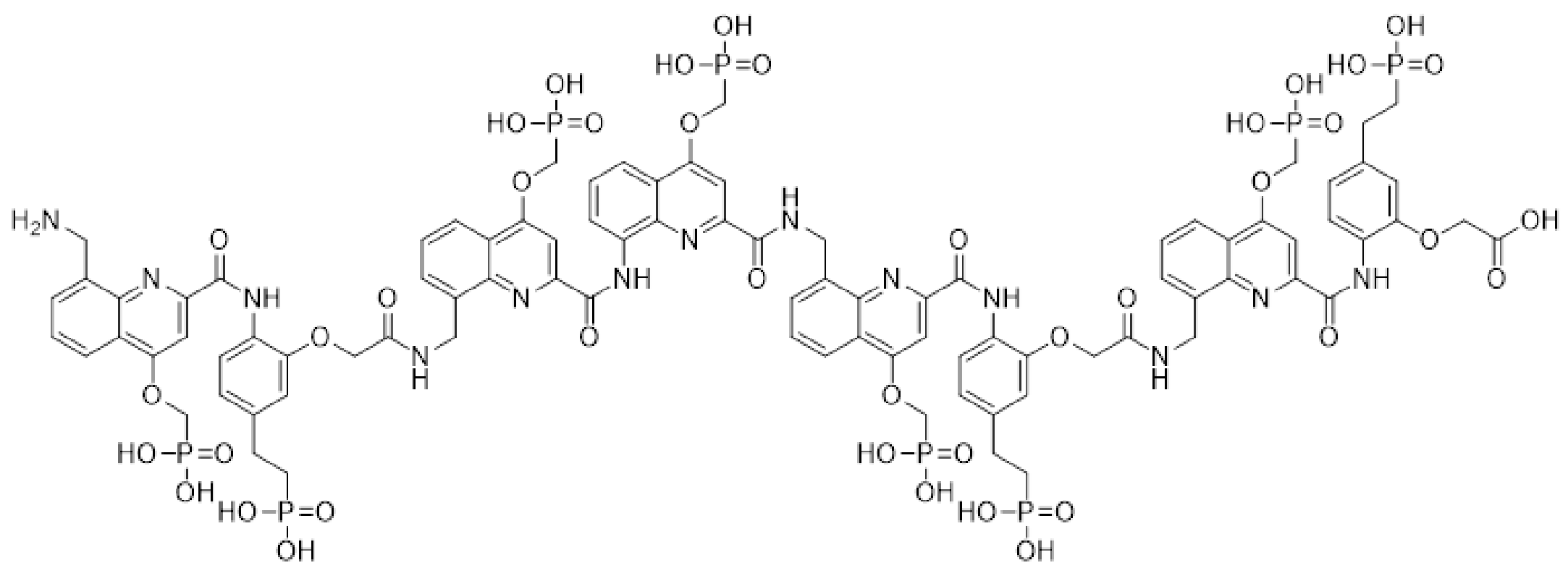

H-MBᴾMQ⁴MBᴾMBᴾ-OH (13): Oligomer **13** was synthesized by the automated SPFS.[10] The title compound was obtained as a white solid (14.6 mg, 38%).**¹H NMR (500 MHz, H_2O/D_2O 9:1):** δ (ppm) = 10.71 (s, 1H), 10.27 (s, 1H), 10.20 (s, 1H), 9.93 (s, 1H), 9.58 (s, 1H), 9.45 (s, 1H), 9.37 - 9.28 (m, 3H), 8.95 (t, *J* = 7.4 Hz, 1H), 8.42 (d, *J* = 9.5 Hz, 1H), 8.22 (d, *J* = 9.5 Hz, 2H), 8.04 (s, 1H), 7.84 - 7.69 (m, 5H), 7.51 – 7.23 (m, 17H), 7.17 - 7.13 (m, 5H), 6.99 - 6.80 (m, 2H), 6.72 – 6.48 (m, 5H), 6.34 - 6.23 (m, 4H), 6.12 (s, 1H), 5.95 (d, *J* = 6.6 Hz, 2H), 5.88 (s, 1H), 5.79 (d, *J* = 7.2 Hz, 2H), 5.71 (s, 1H), 5.47 – 5.41 (m, 2H), 3.86 – 3.75 (m, 1H), 3.73 (d, *J* = 8.3 Hz, 2H), 3.72 – 3.65 (m, 3H), 3.65 - 3.41 (m, 6H), 3.03 - 2.91 (m, 3H), 2.30 – 2.08 (m, 6H), 1.99 -1.81 (m, 3H), 1.41 – 1.17 (m, 7H), 1.12 – 0.98 (m, 1H). **HRMS** (ESI^-) *m/z* calcd. for $C_{89}H_{91}N_{13}O_{41}P_8$: 1121.6595 $[M-2H]^{2-}$; found: 1121.6540.

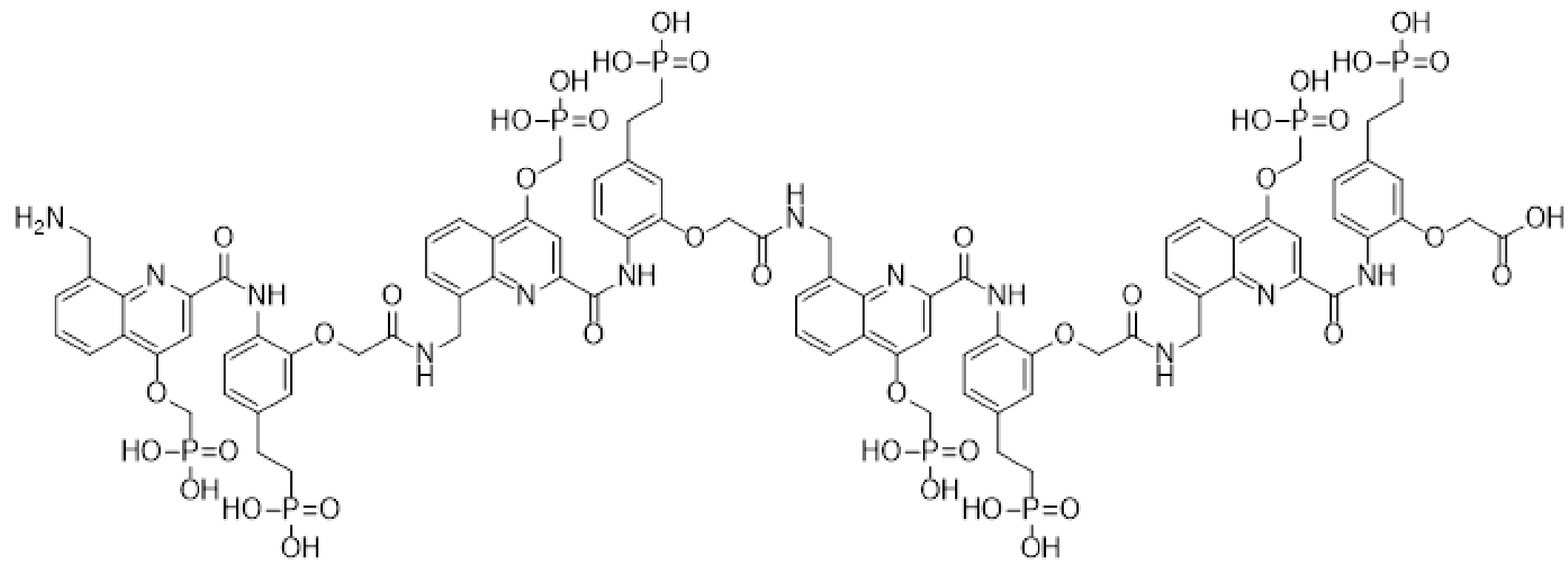

H-MB^{P}MB^{P}MB^{P}MB^{P}-OH (14): Oligomer **14** was synthesized by the automated SPFS,[10] The title compound was obtained as a white solid (13.6 mg, 51%).**^{1}H NMR (500 MHz, H_2O/D_2O 9:1):**δ (ppm) = 10.44 (s, 1H), 10.19 (s, 1H), 10.14 (s, 1H), 8.37 (d, *J* = 9.5 Hz, 1H), 8.26 (t, *J* = 10.0 Hz, 2H), 8.19 (d, *J* = 9.4 Hz, 1H), 7.70 – 7.61 (m, 3H), 7.60 – 7.41 (m, 7H), 7.31 - 7.26 (m, 3H), 7.19 (s, 1H), 7.14 (d, *J* = 9.2 Hz, 2H), 7.07 (t, *J* = 7.5 Hz, 1H), 6.62 - 6.57 (m, 6H), 6.50 - 6.43 (m, 3H), 6.39 (s, 1H), 6.32 – 6.24 (m, 2H), 6.04 - 4.09 (m, 3H), 3.97 (d, *J* = 9.8 Hz, 1H), 3.82 (d, *J* = 9.6 Hz, 3H), 3.55 - 3.51 (m, 5H), 3.05 (d, *J* = 8.6 Hz, 1H), 1.86 (s, 1H), 1.78 (s, 1H), 1.15 - 1.11 (m, 2H). **HRMS** (ESI^{-}) *m/z* calcd. for $C_{88}H_{94}N_{12}O_{41}P_8$: 1110.1697 [M-2H]$^{2-}$; found: 1110.1651.

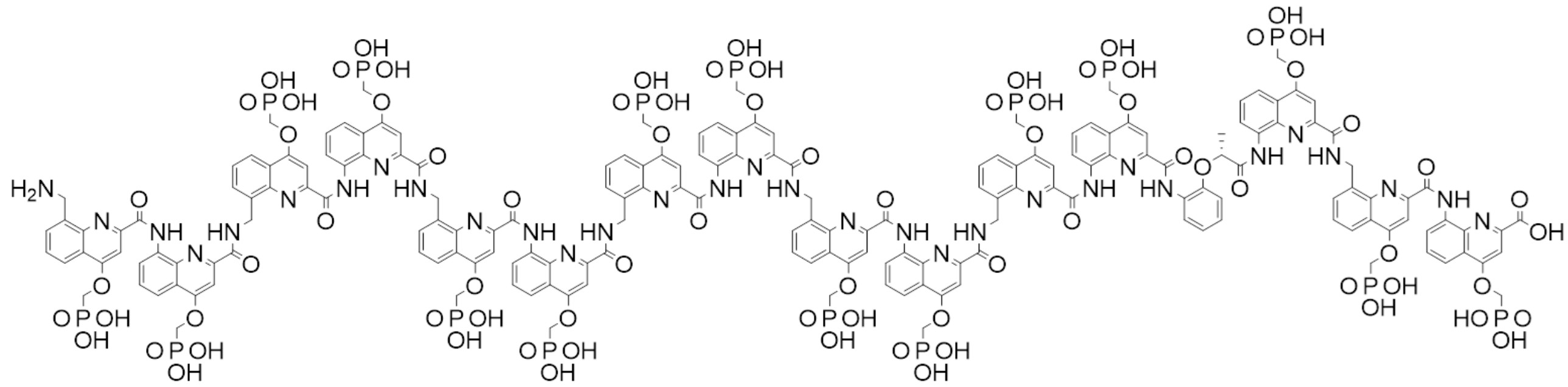

H-MQ^{4}MQ^{4}MQ^{4}MQ^{4}MQ^{4}MQ^{4}B^{R}Q^{4}MQ^{4}-OH(15): Oligomer **15** was previously reported.[12]

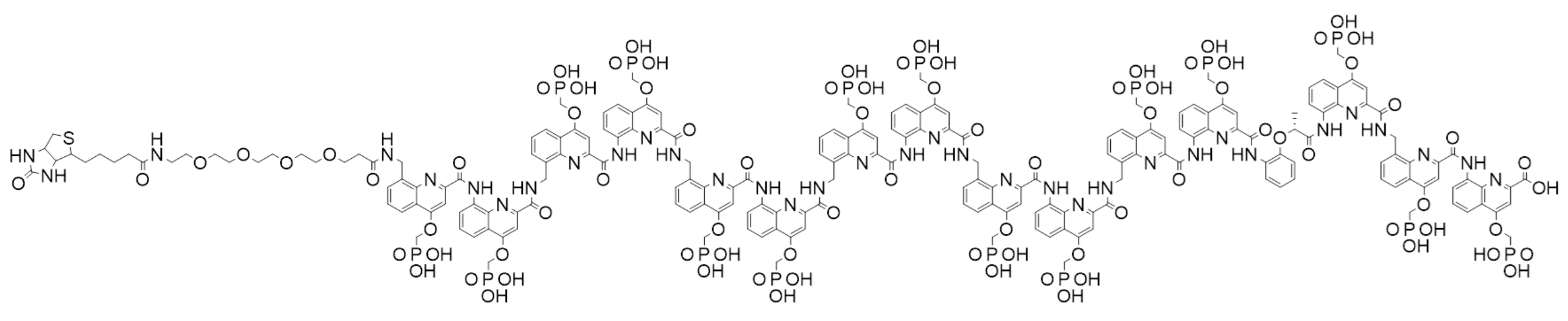

Biotin-PEG$_4$-MQ^{4}MQ^{4}MQ^{4}MQ^{4}MQ^{4}MQ^{4}B^{R}Q^{4}MQ^{4}-OH (15a): Oligomer **15a** was synthesized according to the previously reported protocol[11] and purified by semi-preparative RP-HPLC (TEAA buffer system, linear gradient 0-30% B in A (Kinetex C18 EVO column). From the lyophilized powder, the Et_3NH^+ cations were exchanged with NH_4^+ (Section 3.3) to give the poly-ammonium salt of

compound **15a** as yellow solid (1.0 mg, 50%). **HRMS** (ESI$^-$) *m/z* calcd. for $C_{202}H_{195}N_{34}O_{85}P_{15}S$: 1237.1868 [M-4H]$^{4-}$; found: 1237.2724.

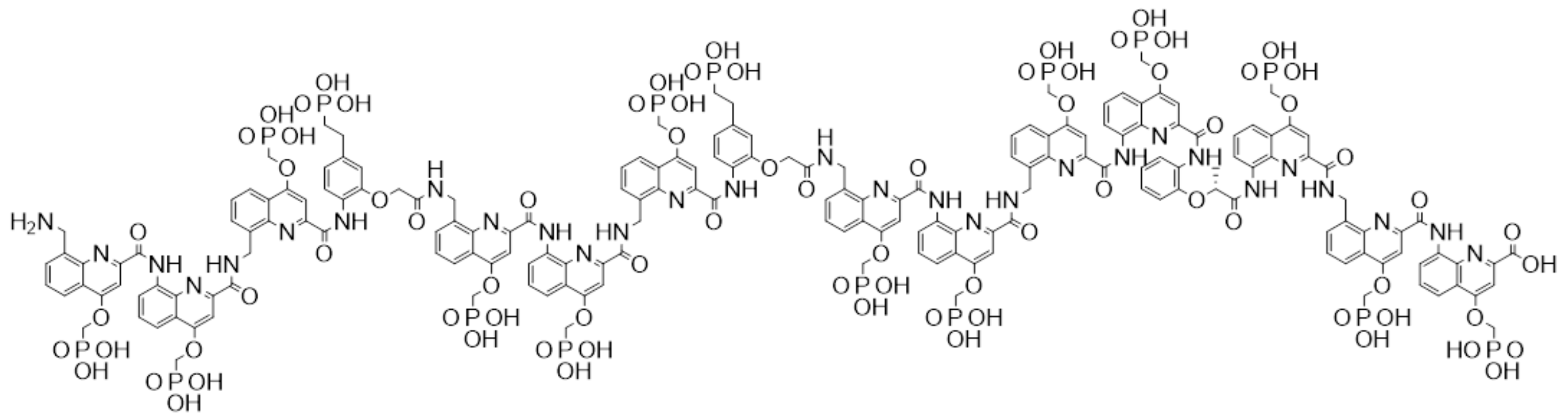

H-MQ^{4}MB^{P}MQ^{4}MB^{P}MQ^{4}MQ^{4}B^{R}Q^{4}MQ^4-OH(16): Oligomer **16** was synthesized by the automated SPFS.[10] The title compound was obtained as a white solid (17.3 mg, 46%).**^{1}H NMR (500 MHz, H_2O/D_2O 9:1):** δ (ppm) = 11.17 (s, 1H), 11.10 (s, 1H), 10.28 (s, 1H), 10.22 -10.19 (m, 2H), 9.69 (s, 1H), 9.56 (d, *J* = 7.7 Hz, 1H), 9.51 (s, 1H), 9.22 (s, 1H), 9.12 (s, 1H), 8.94 (s, 1H), 8.78 (s, 1H), 8.66 (s, 1H), 8.36 (d, *J* = 8.8 Hz, 1H), 8.01 -7.96 (m, 3H), 7.81 -7.75 (m, 4H), 7.71 – 7.54 (m, 6H), 7.50 – 7.27 (m, 8H), 7.26 – 6.84 (m, 17H), 6.84 – 6.61 (m, 10H), 6.53 (t, *J* = 9.0 Hz, 1H), 6.39 (s, 1H), 6.31 (s, 1H), 6.26 (d, *J* = 8.8 Hz, 1H), 6.03 – 5.91 (m, 3H), 5.84 (d, *J* = 9.3 Hz, 1H), 5.78 (s, 1H), 5.69 (d, *J* = 9.1 Hz, 1H), 4.20 – 3.46 (m, 14H), 3.23 (t, *J* = 10.7 Hz, 1H), 3.09 - 2.83 (m, 3H), 2.62 (s, 1H), 2.47 – 2.27 (m, 3H), 2.16 – 1.80 (m, 3H), 1.34 – 1.07 (m, 4H), -0.01 (d, *J* = 6.5 Hz, 3H). **HRMS** (ESI$^-$) *m/z* calcd. for $C_{179}H_{166}N_{29}O_{78}P_{15}$: 1476.8587 [M-3H]$^{3-}$; found: 1476.8543.

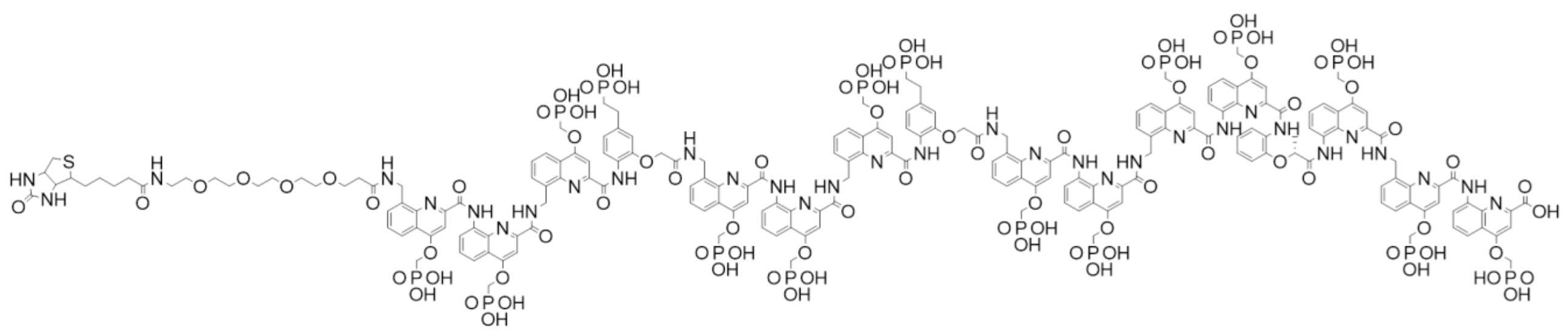

Biotin-PEG$_4$-MQ^{4}MB^{P}MQ^{4}MB^{P}MQ^{4}MQ^{4}B^{R}Q^{4}MQ^4 (16a): Oligomer **16a** was synthesized according to the previously reported protocol[11] and purified by semi-preparative RP-HPLC (TEAA buffer system, linear gradient 0-30% B in A (Kinetex C18 EVO column). From the lyophilized powder, the Et_3NH^+ cations were exchanged with NH_4^+ (Section 3.3) to give the poly-ammonium salt of compound **16a** as yellow solid (1.3 mg, 72%). **HRMS** (ESI$^-$) *m/z* calcd. for $C_{200}H_{201}N_{32}O_{85}P_{15}S$: 1225.6971 [M-4H]$^{4-}$; found: 1225.7696.

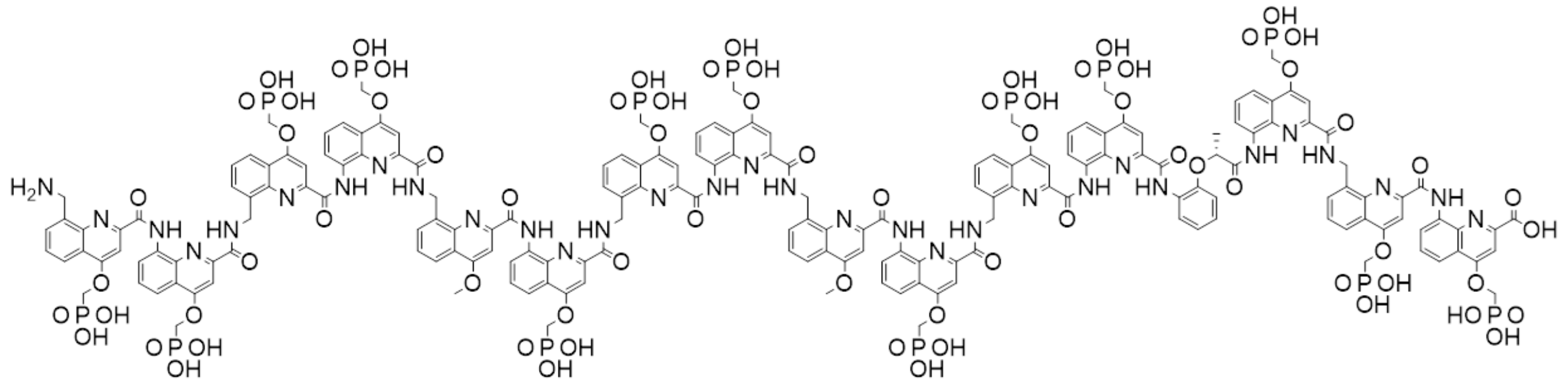

H-MQ^{4}MQ^{4}MQ^{4}MQ^{4}MQ^{4}MQ^{4}B^{R}Q^{4}MQ^4-OH (17): Oligomer **17** was previously reported.

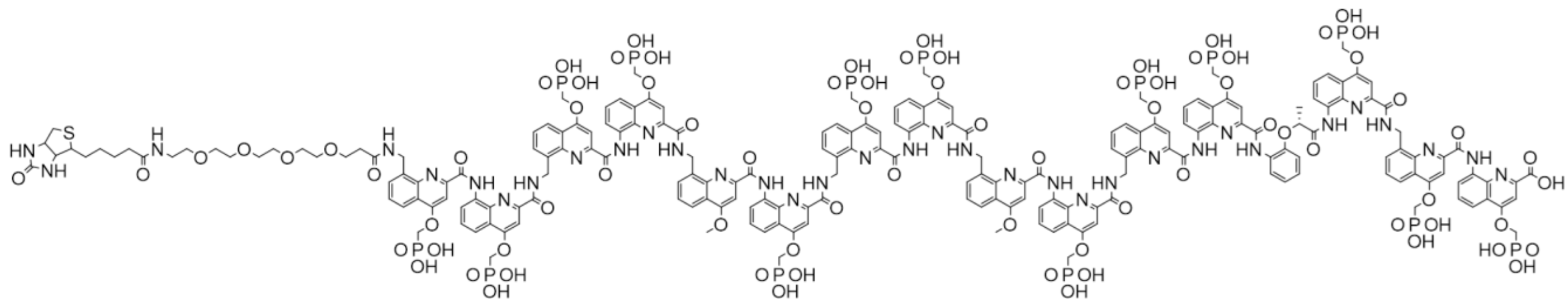

Biotin-PEG$_4$-MQ^{4}MQ^{4}MQ^{4}MQ^{4}MQ^{4}MQ^{4}B^{R}Q^{4}MQ^4-OH (17a): Oligomer **17a** was synthesized according to the previously reported protocol[11] and purified by semi-preparative RP-HPLC (TEAA buffer system, linear gradient 0-30% B in A (Kinetex C18 EVO column). From the lyophilized powder, the Et_3NH^+ cations were exchanged with NH_4^+ (Section 3.3) to give the poly-ammonium salt of compound **17a** as yellow solid (0.8 mg, 37%). **HRMS** (ESI$^-$) *m/z* calcd. for $C_{202}H_{193}N_{34}O_{79}P_{13}S$: 1197.2037 [M-4H]$^{4-}$; found: 1197.2696.

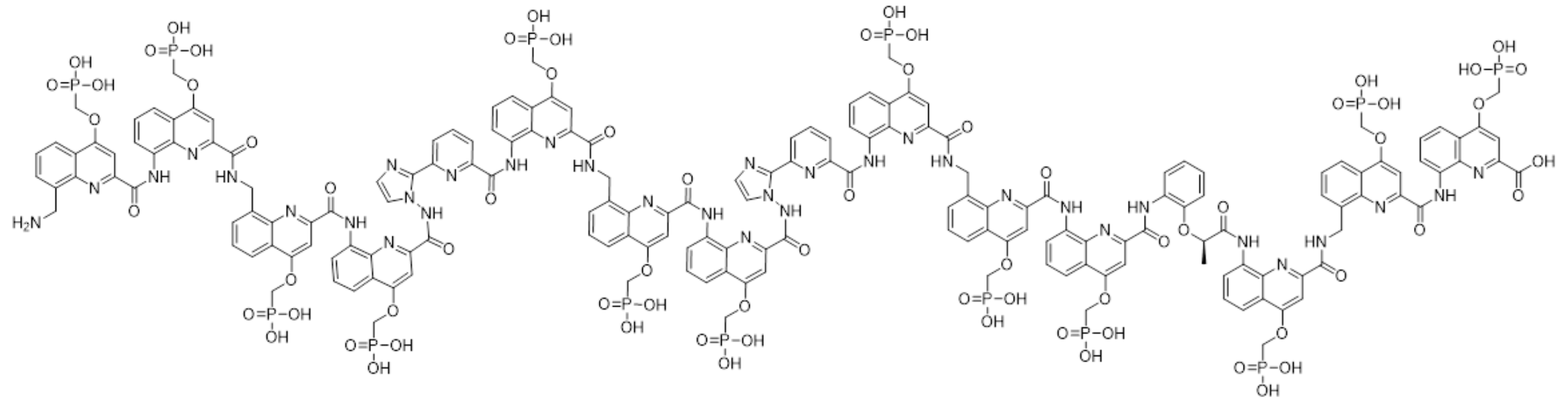

H-MQ^{4}MQ^{4}NQ^{4}MQ^{4}NQ^{4}MQ^{4}B^{R}Q^{4}MQ^4-OH (18): Oligomer **18** was synthesized by the automated SPFS.[10] The title compound was obtained as a white solid (16.2 mg, 37%). **^{1}H NMR (500 MHz, H_2O/D_2O 9:1):** δ (ppm) =12.43 (s, 1H), δ 11.77 (s, 1H), 11.19 (s, 1H), 11.10 (s, 1H), 10.18 (s, 1H), 10.10 (s, 1H), 9.83 (s, 1H), 9.67 (s, 1H), 9.48 (s, 1H), 9.41 (d, *J* = 8.0 Hz, 2H), 9.26 (s, 1H), 9.22 (s, 1H), 8.92 – 8.88 (m, 2H), 8.34 (d, *J* = 8.8 Hz, 1H), 8.07 – 7.97 (m, 3H), 7.88 (d, *J* = 8.7 Hz, 1H), 7.80 (d, *J* = 4.0 Hz, 2H), 7.75 (s, 1H), 7.64 (t, *J* = 6.4 Hz, 1H), 7.56 (d, *J* = 6.6 Hz, 1H), 7.51 – 7.45 (m, 2H), 7.44 – 7.34 (m, 2H), 7.36 – 7.17 (m, 11H), 7.14 (d, *J* = 8.4 Hz, 2H), 7.14 – 7.00 (m, 2H), 7.00 – 6.68 (m, 12H),

6.65 (d, *J* = 8.4 Hz, 2H), 6.63 – 6.38 (m, 11H), 6.34 - 6.25 (d, *J* = 3.6 Hz, 3H), 6.22 – 6.11 (m, 2H), 6.09 (s, 1H), 4.02 – 3.84 (m, 5H), 3.80 - 3.71 (m, 5H), 3.60 – 3.52 (m, 2H), 3.41 – 3.06 (m, 3H), 3.01 – 1.15 (m, 3H), 0.32 (d, *J* = 6.6 Hz, 3H). **HRMS** (ESI^-) *m/z* calcd. for $C_{175}H_{150}N_{35}O_{70}P_{13}$: 1064.8888 $[M-4H]^{4-}$; found: 1064.8857.

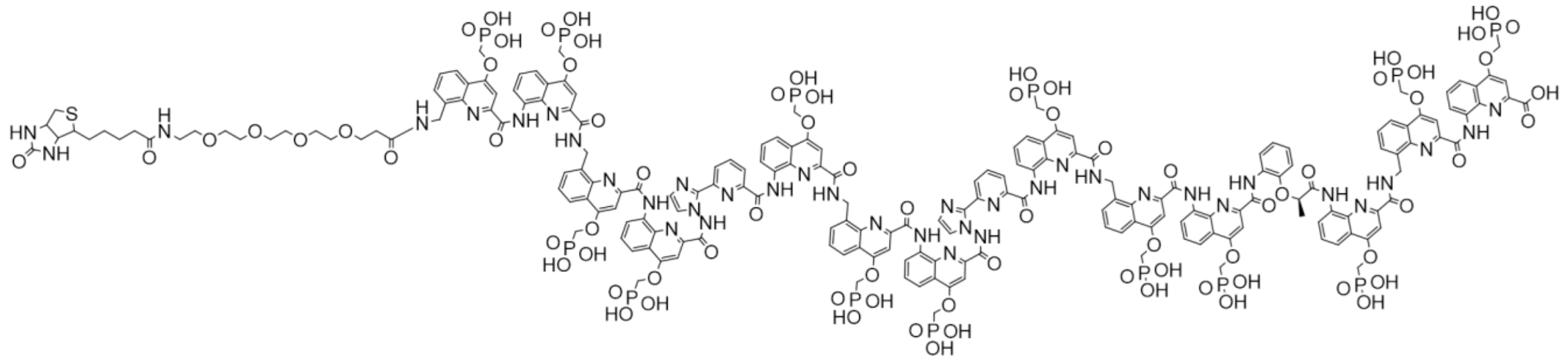

Biotin-PEG$_4$-MQ^{4}MQ^{4}NQ^{4}MQ^{4}NQ^{4}MQ^{4}B^{R}Q^{4}MQ^4-OH (18a): Oligomer **18a** was synthesized according to the previously reported protocol[11] and purified by semi-preparative RP-HPLC (TEAA buffer system, linear gradient 0-30% B in A (Kinetex C18 EVO column). From the lyophilized powder, the Et_3NH^+ cations were exchanged with NH_4^+ (Section 3.3) to give the poly-ammonium salt of compound **18a** as yellow solid (0.5 mg, 39%). **HRMS** (ESI^-) *m/z* calcd. for $C_{196}H_{185}N_{38}O_{77}P_{13}S$: 1183.1937 $[M-4H]^{4-}$; found: 1183.2524.

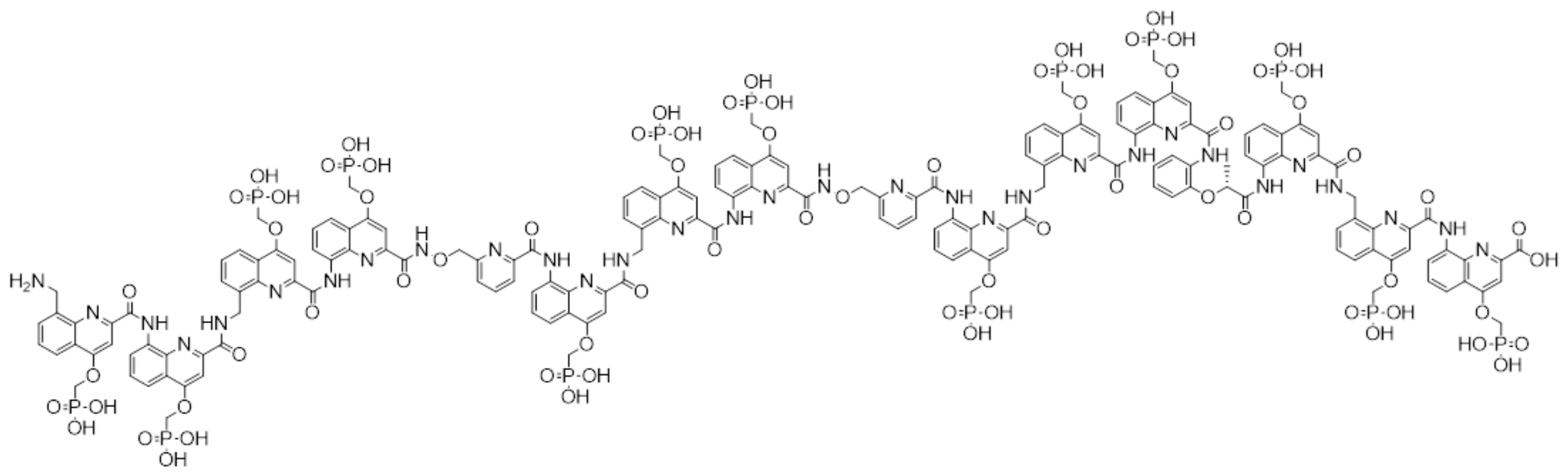

H-MQ^{4}MQ^{4}OQ^{4}MQ^{4}OQ^{4}MQ^{4}B^{R}Q^{4}MQ^4-OH (19): Oligomer **19** was synthesized by the automated SPFS.[10] The title compound was obtained as a white solid (15 mg, 42%). **^{1}H NMR (500 MHz, H_2O/D_2O 9:1):** δ (ppm)= 11.35 (s, 1H), 11.19 (s, 1H), 10.34 (s, 1H), 10.04 (d, *J* = 8.0 Hz, 1H), 9.99 (s, 1H), 9.95 (s, 1H), 9.89 (s, 1H), 9.63 (s, 1H), 9.51 -9.49 (m, 2H), 9.44 (s, 1H), 9.27 (s, 1H), 9.21 (d, *J* = 7.9 Hz, 1H), 9.01 (s, 1H), 8.92 – 8.87 (m, 1H), 8.46 (dd, *J* = 8.9, 2.8 Hz, 2H), 8.40 (d, *J* = 8.9 Hz, 1H), 8.08 -8.03 (m, 2H), 7.87 (d, *J* = 7.9 Hz, 1H), 7.71 – 7.63 (m, 2H), 7.63 – 7.58 (m, 2H), 7.61 – 7.57 (m, 1H), 7.54 (d, *J* = 6.9 Hz, 1H), 7.51 – 7.44 (m, 5H), 7.43 – 7.36 (m, 4H), 7.33 - 7.26 (m, 10H), 7.15 – 6.99 (m, 5H), 6.99 - 6.98 (s, 4H), 6.98 – 6.74 (m, 14H), 6.76 – 6.67 (m, 2H), 6.70 (s, 1H), 6.68 – 6.60 (m,

3H), 6.31- 6.14 (m, 4H), 5.98 (s, 1H), 5.73 (d, *J* = 7.2 Hz, 2H), 4.16 (d, *J* = 10.1 Hz, 2H), 4.14 – 4.05 (m, 3H), 4.05 – 3.83 (m, 5H), 3.80 (d, *J* = 9.3 Hz, 2H), 3.78 – 3.69 (m, 1H), 3.68 (s, 3H), 3.67 – 3.61 (m, 1H), 3.64 – 3.51 (m, 2H), 3.44 - 2.96 (m, 4H), 2.75 – 2.67 (m, 1H), 1.71 - 1.66 (m, 2H), 1.34 – 1.23 (m, 2H), -0.01 (d, *J* = 6.7 Hz, 3H). **HRMS** (ESI^-) *m/z* calcd. for $C_{171}H_{150}N_{31}O_{72}P_{13}$: 1046.8832 $[M-4H]^{4-}$; found: 1046.8864.

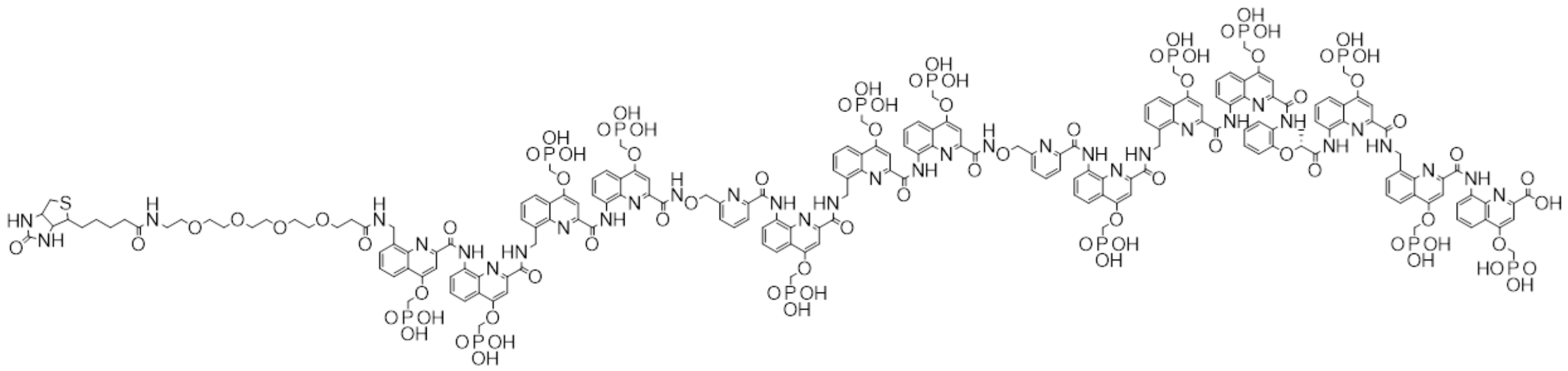

Biotin-PEG$_4$-MQ^{4}MQ^{4}OQ^{4}MQ^{4}OQ^{4}MQ^{4}B^{R}Q^{4}MQ^4-OH (19a): Oligomer **19a** was synthesized according to the previously reported protocol[11] and purified by semi-preparative RP-HPLC (TEAA buffer system, linear gradient 0-30% B in A (Kinetex C18 EVO column). From the lyophilized powder, the Et_3NH^+ cations were exchanged with NH_4^+ (Section 3.3) to give the poly-ammonium salt of compound **19a** as yellow solid (0.6 mg, 32%). **HRMS** (ESI^-) *m/z* calcd. for $C_{192}H_{185}N_{34}O_{79}P_{13}S$: 1165.1880 $[M-4H]^{4-}$; found: 1165.2528.

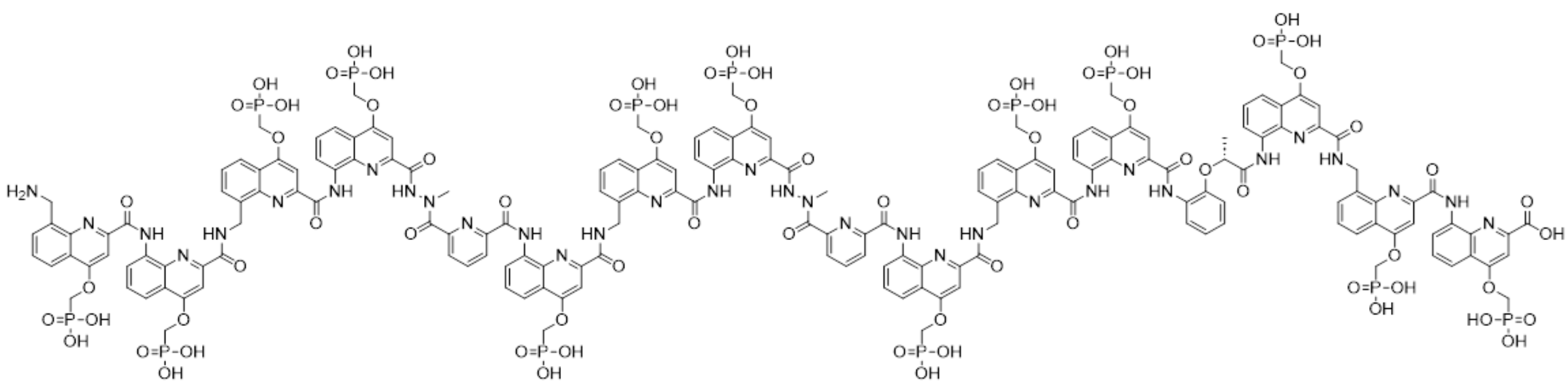

H-MQ^{4}MQ^{4}HQ^{4}MQ^{4}HQ^{4}MQ^{4}B^{R}Q^{4}MQ^4-OH (20): Oligomer **20** was synthesized by the automated SPFS.[10] The title compound was obtained as a white solid (14.8 mg, 35%). **^{1}H NMR (500 MHz, H_2O/D_2O 9:1):** δ (ppm) = 11.31 (s, 1H), 11.01 (s, 1H), 10.55 (s, 1H), 10.30 (s, 1H), 10.12 (s, 1H), 9.92 (s, 1H), 9.64 (d, *J* = 7.8 Hz, 1H), 9.53 (s, 1H), 9.49 (s, 1H), 9.34 (d, *J* = 7.3 Hz, 1H), 9.22 (s, 1H), 9.14 (s, 1H), 9.06 (s, 1H), 8.93 (s, 1H), 8.60 – 8.37 (m, 4H), 8.21 (d, *J* = 8.6 Hz, 1H), 8.09 – 7.94 (m, 3H), 7.92 – 7.58 (m, 15H), 7.57 – 7.31 (m, 12H), 6.87 – 6.63 (m, 8H), 6.62 – 6.51 (m, 2H), 6.44 (d, *J* = 8.7 Hz, 1H), 6.41 (d, *J* = 3.4 Hz, 1H), 6.22 (s, 1H), 6.14 (s, 1H), 5.94 (s, 1H), 5.83 (s, 1H), 3.07 (s, 1H), 2.91 (s, 3H), 2.65 – 2.58 (m, 3H), 0.01 (d, *J* = 6.7 Hz, 3H). **HRMS** (ESI^-) *m/z* calcd. for $C_{173}H_{152}N_{33}O_{72}P_{13}$: 1060.3886 $[M-4H]^{4-}$; found: 1060.3857.

4. NMR spectra

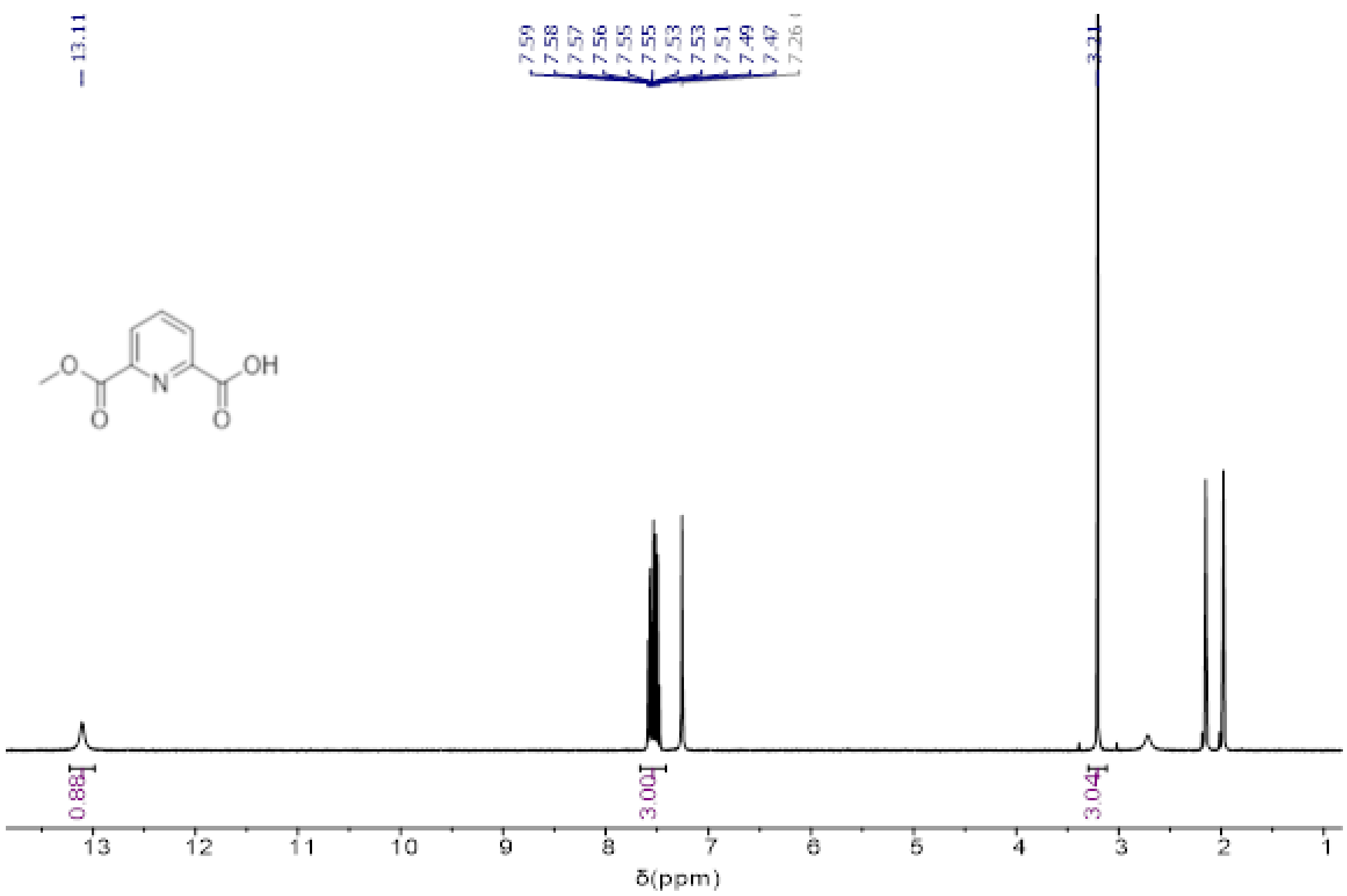

Figure S18. ^{1}H NMR spectrum of compound **1a**. (500 MHz, $CDCl_3$). According to ref. 6 at the start.

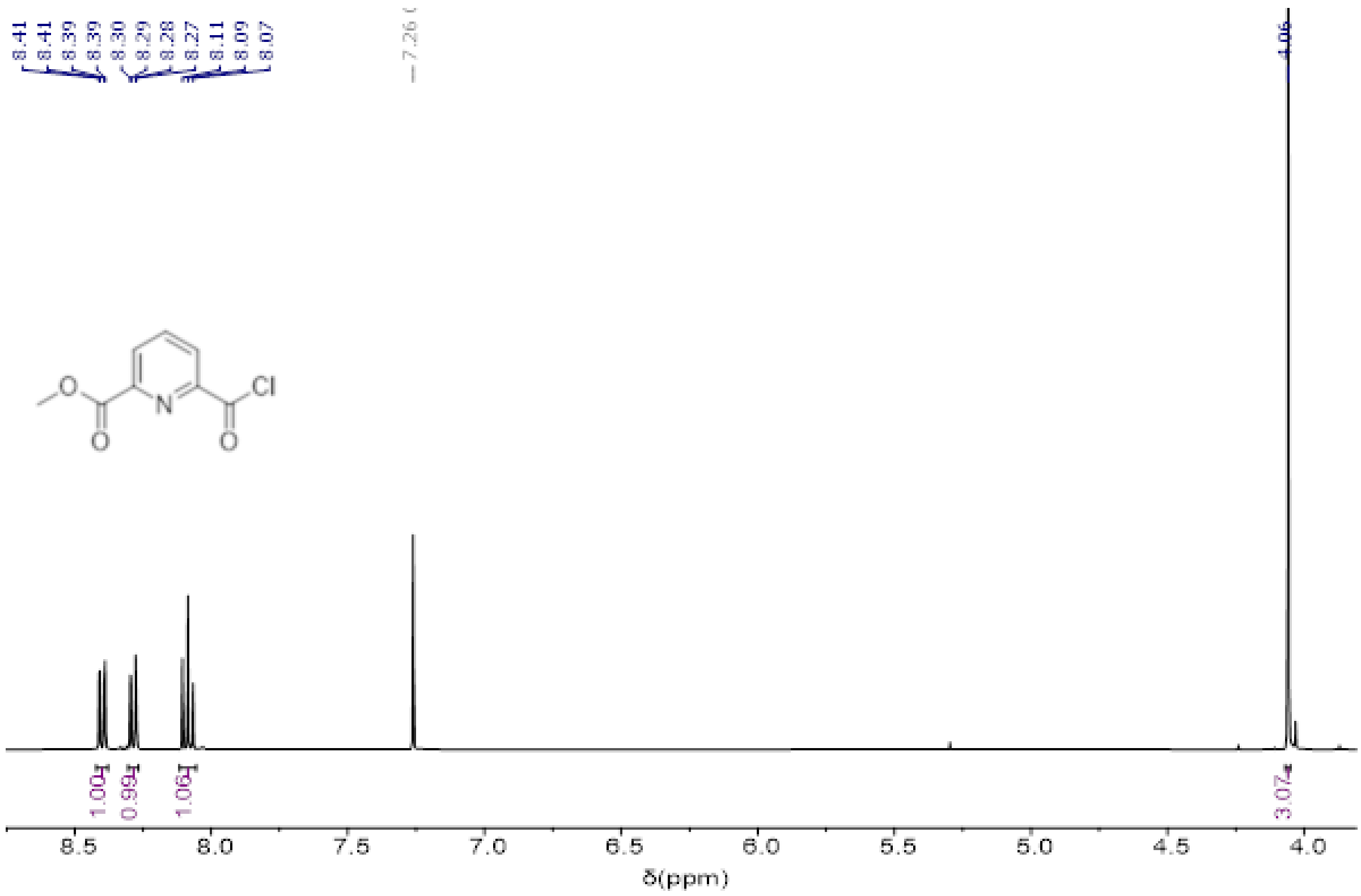

Figure S19. ^{1}H NMR spectrum of compound **1b.** (500 MHz, $CDCl_3$). According to ref. 7 at the start.

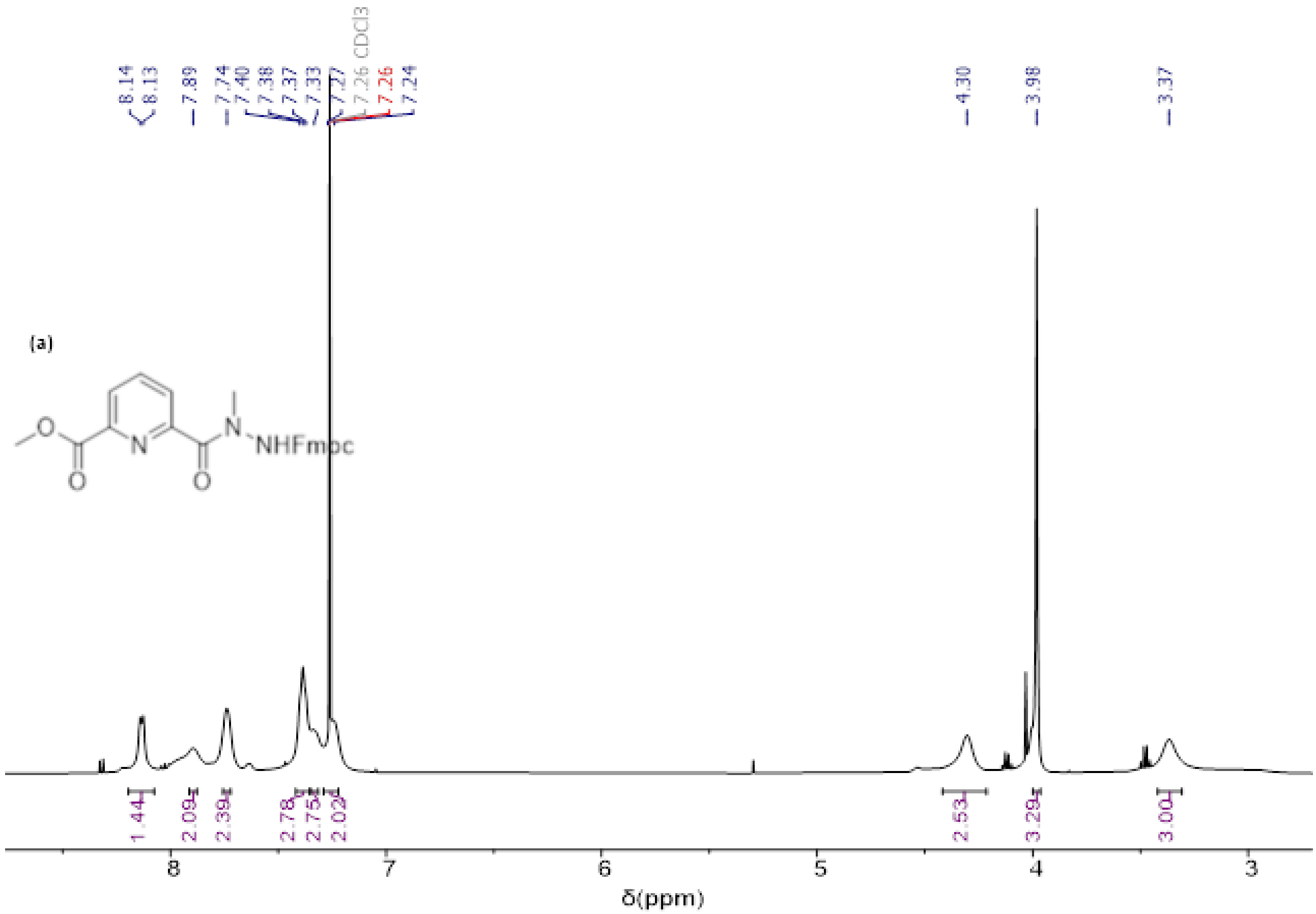

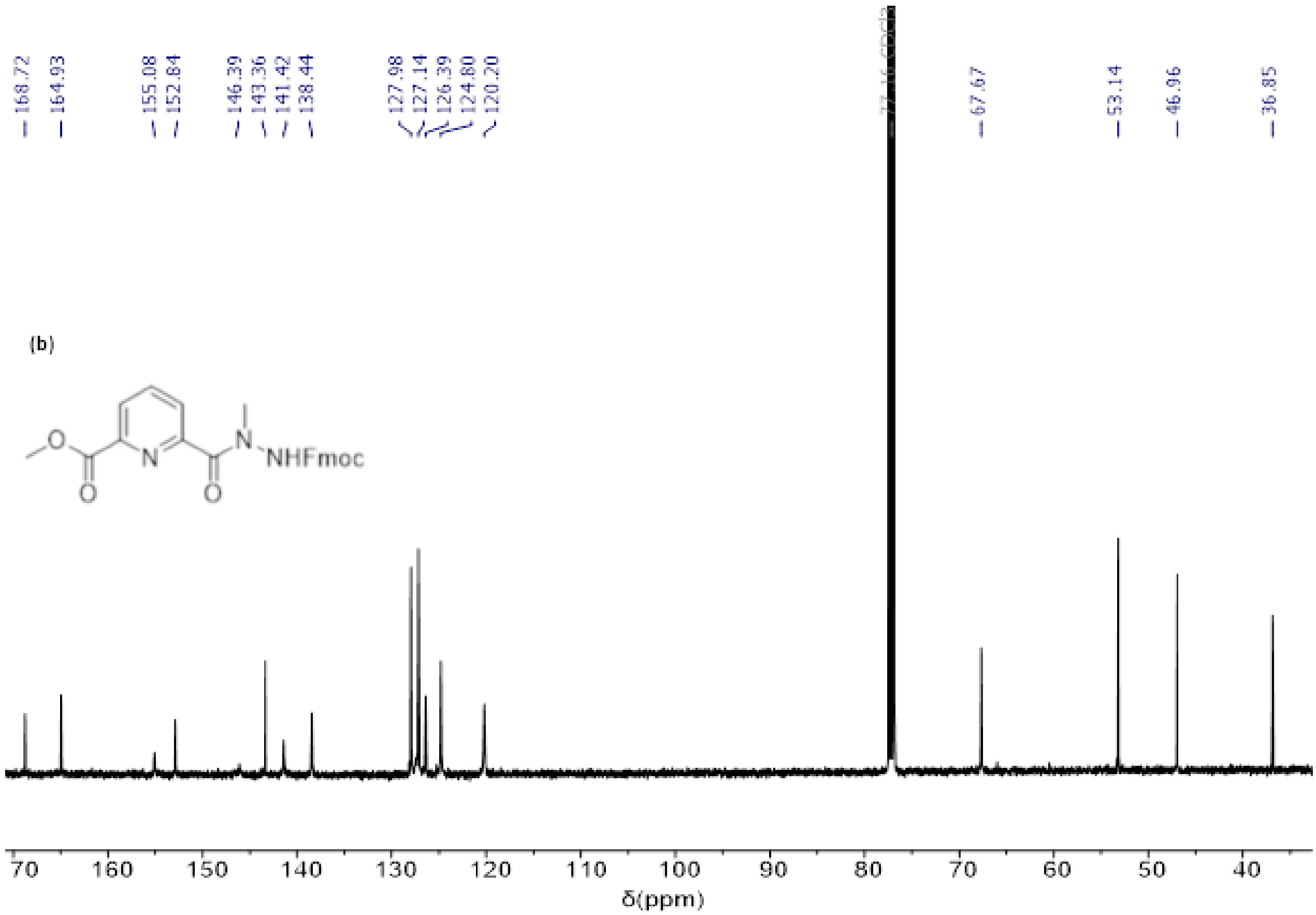

Figure S20. NMR spectra of compound **1c**. (a) ^{1}H NMR (500 MHz, $CDCl_3$). (b) ^{13}C NMR (126 MHz, $CDCl_3$).

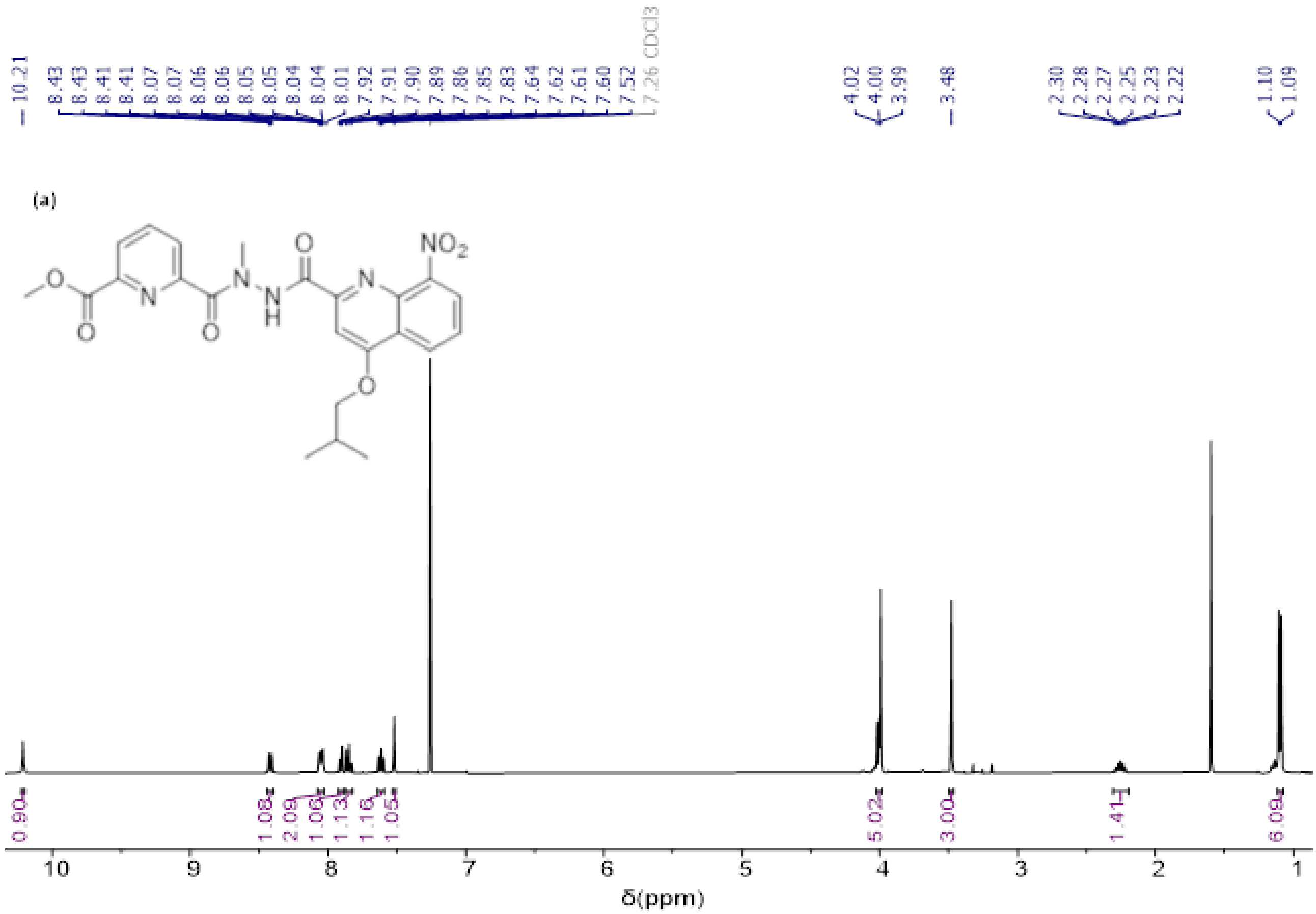

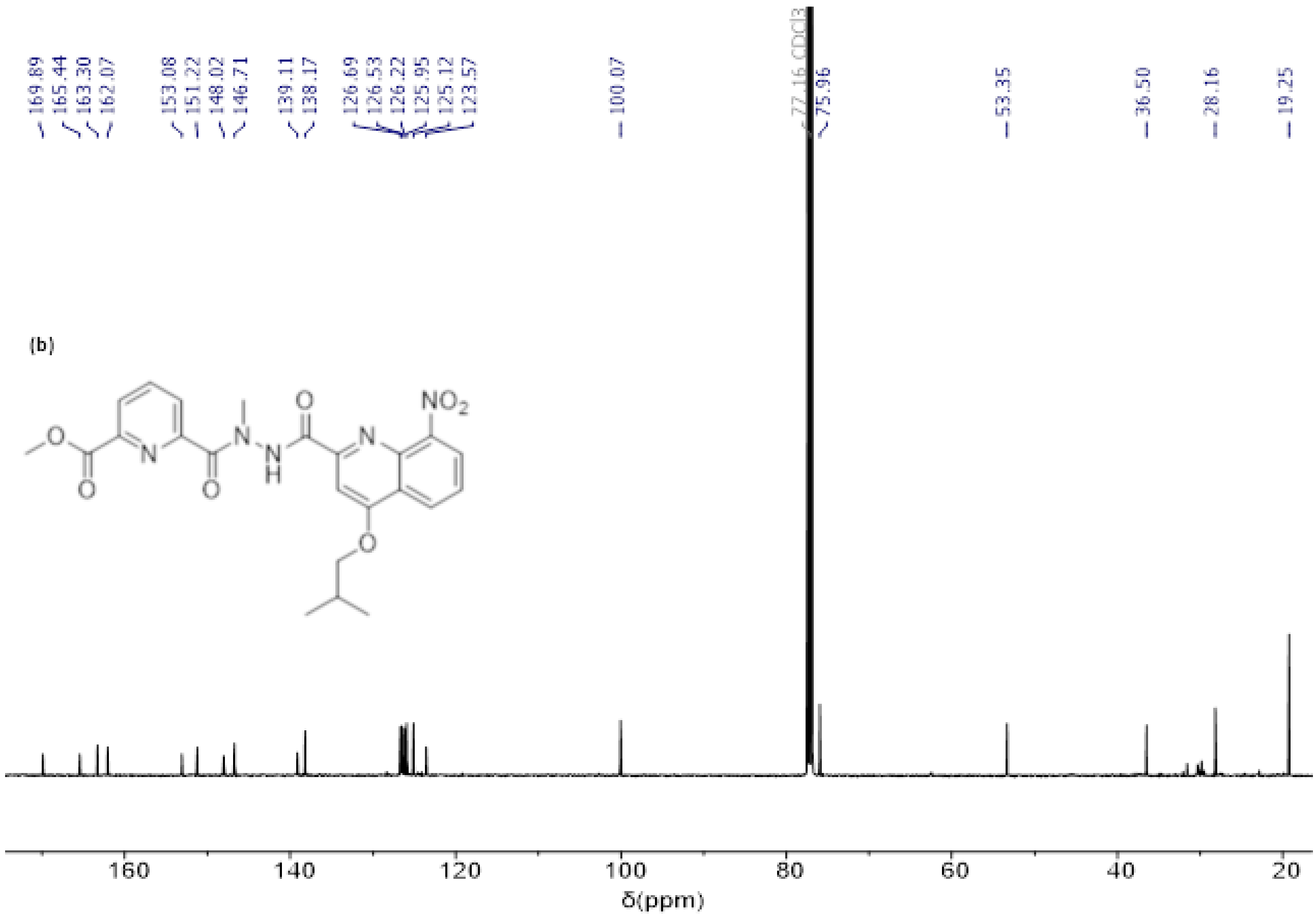

Figure S21. NMR spectra of compound **1d**. (a) ^{1}H NMR (500 MHz, $CDCl_3$). (b) ^{13}C NMR (126 MHz, $CDCl_3$).

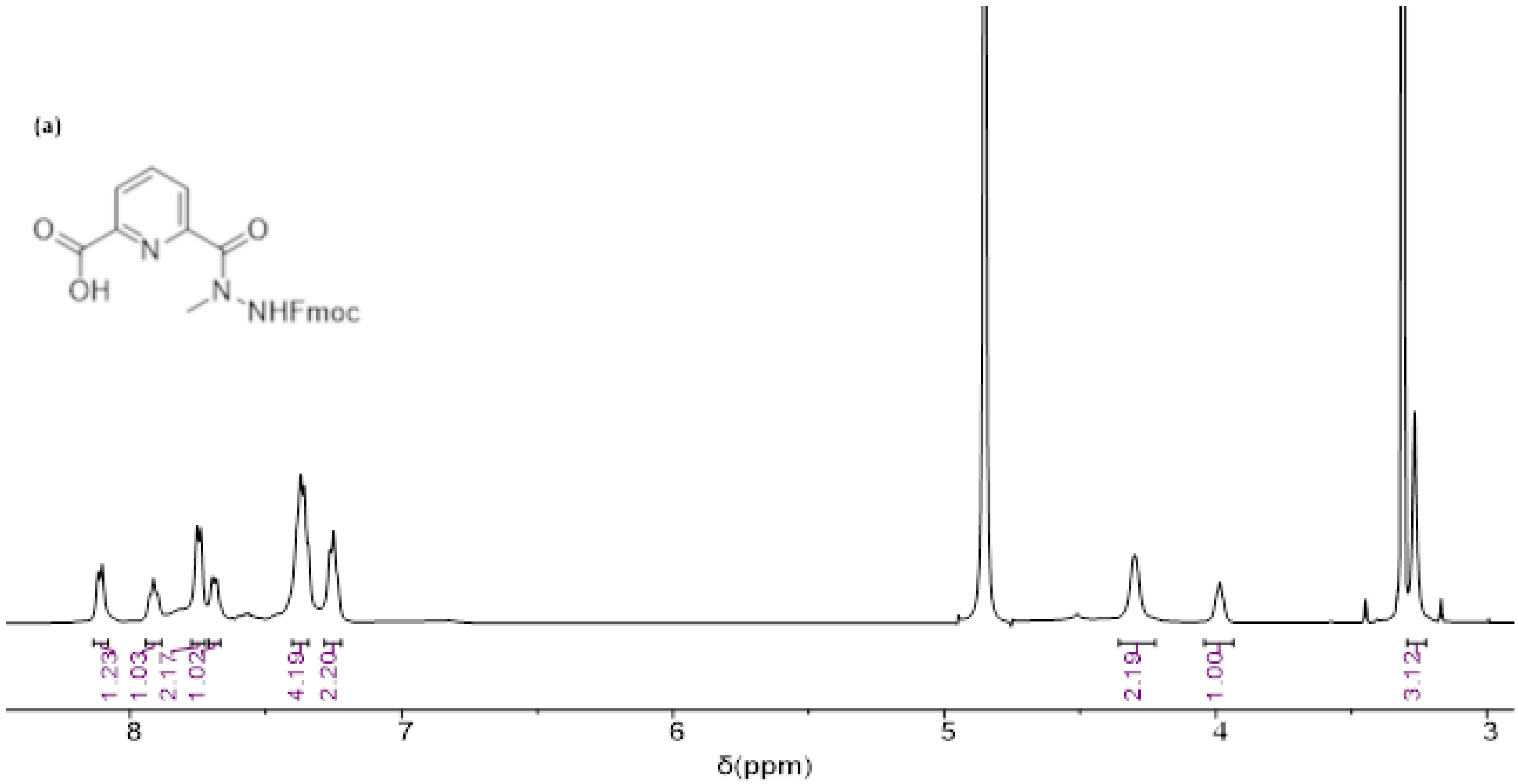

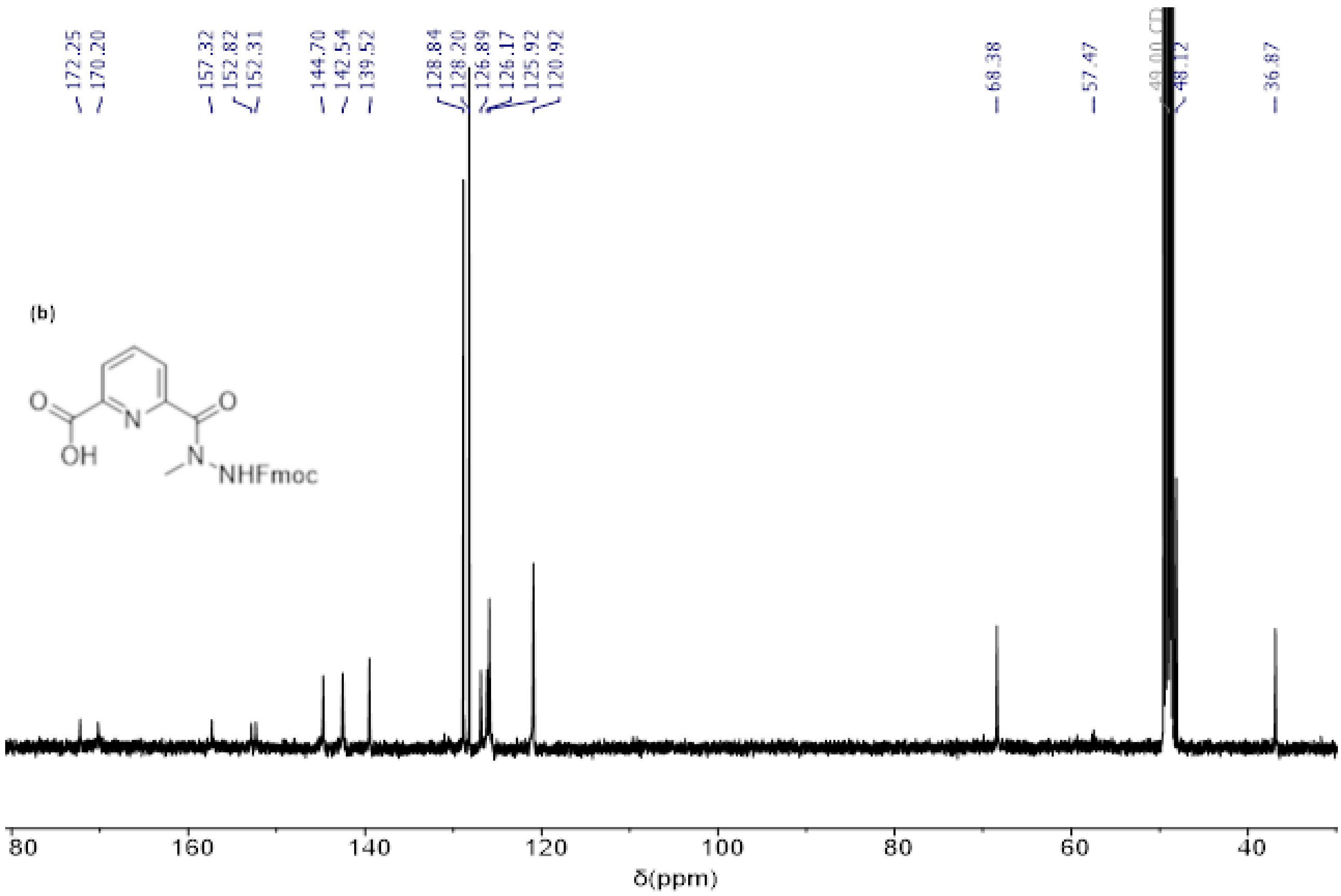

Figure S22. NMR spectra of compound **1**. (a) ^{1}H NMR (500 MHz, CD_3OD). (b) ^{13}C NMR (126 MHz, CD_3OD).

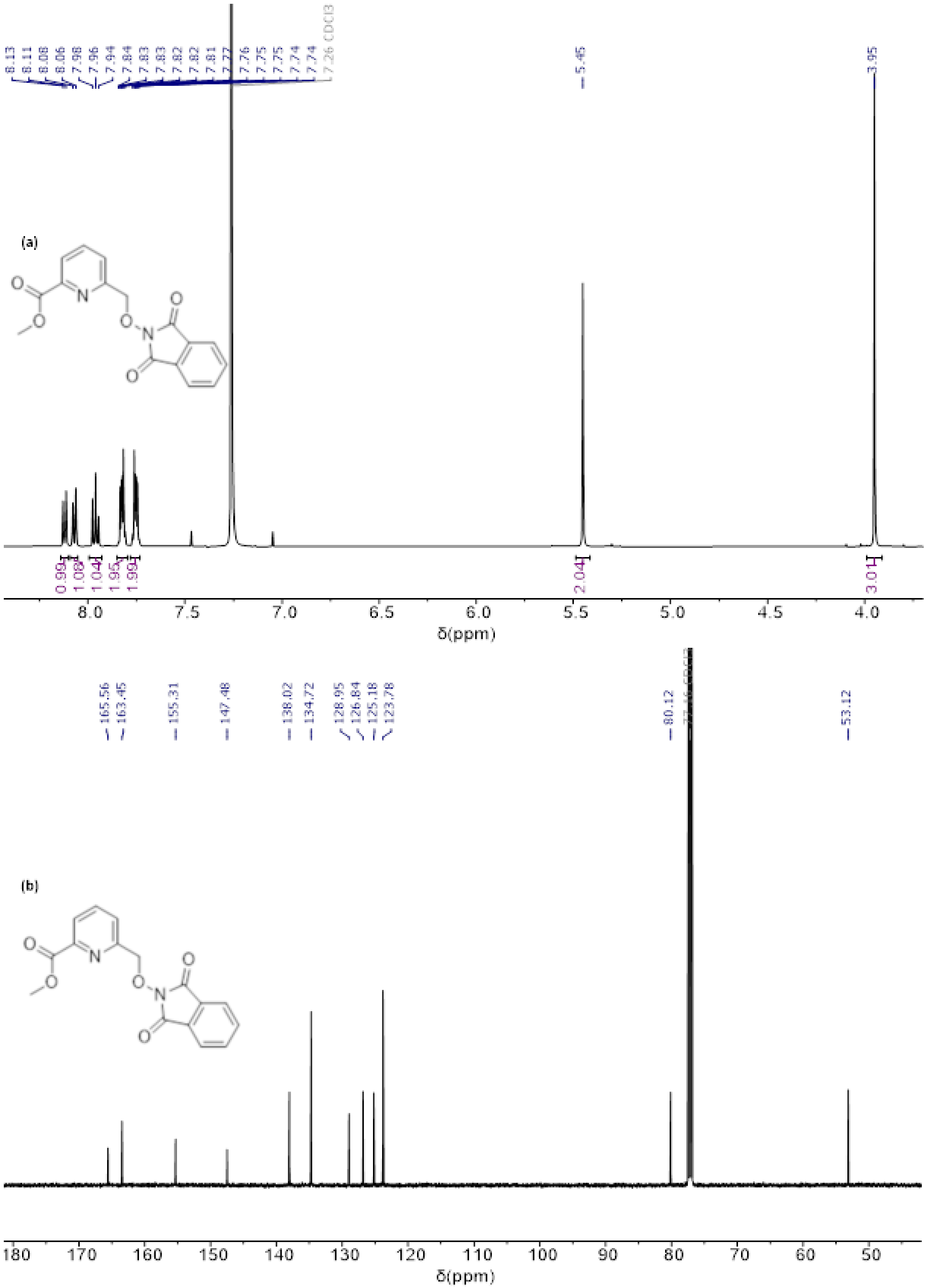

Figure S23. NMR spectra of compound **2a**. (a) ^{1}H NMR (500 MHz, $CDCl_3$). (b) ^{13}C NMR (126 MHz, $CDCl_3$).

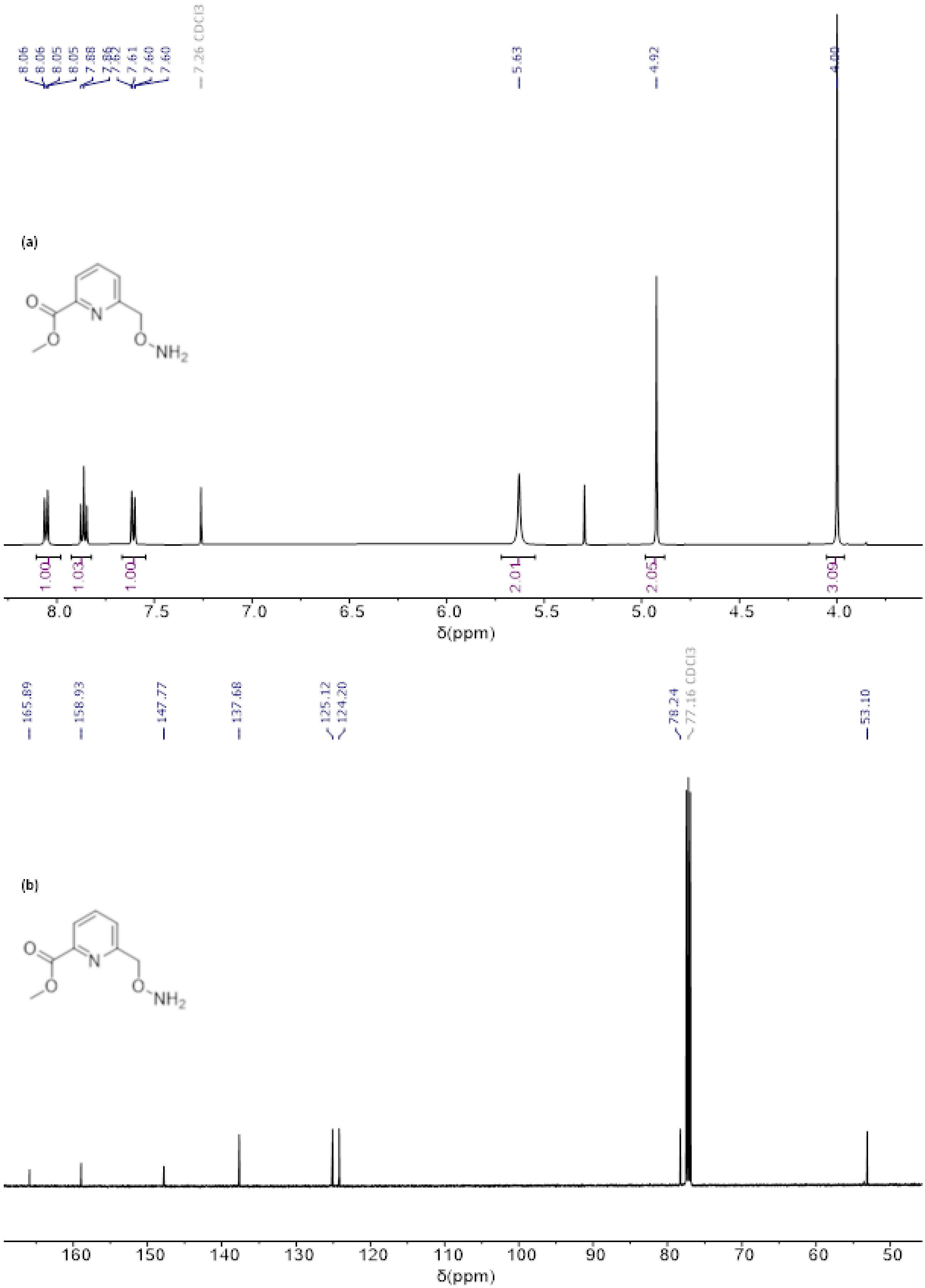

Figure S24. NMR spectra of compound **2b**. (a) ^{1}H NMR (500 MHz, $CDCl_3$). (b) ^{13}C NMR (126 MHz, $CDCl_3$).

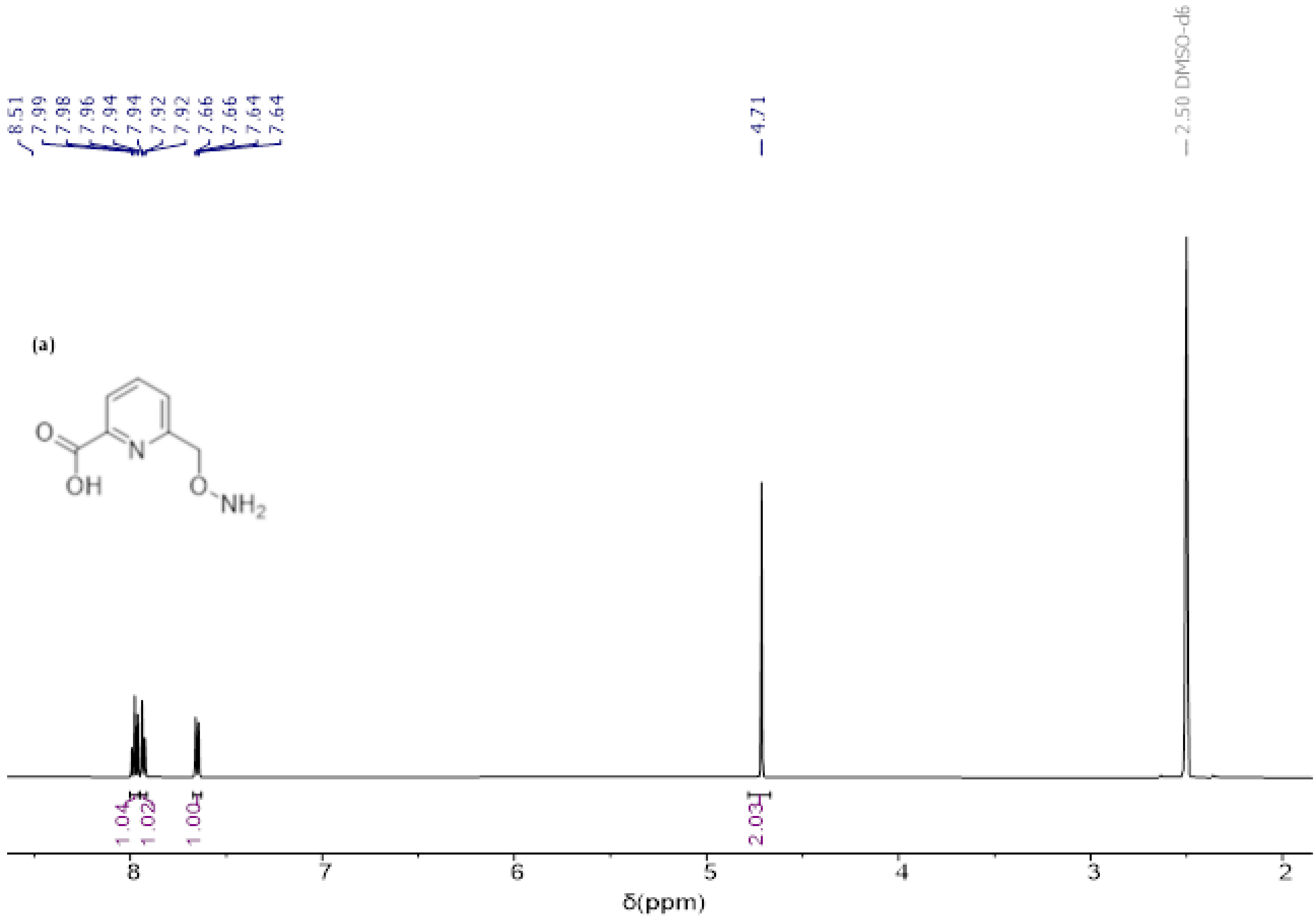

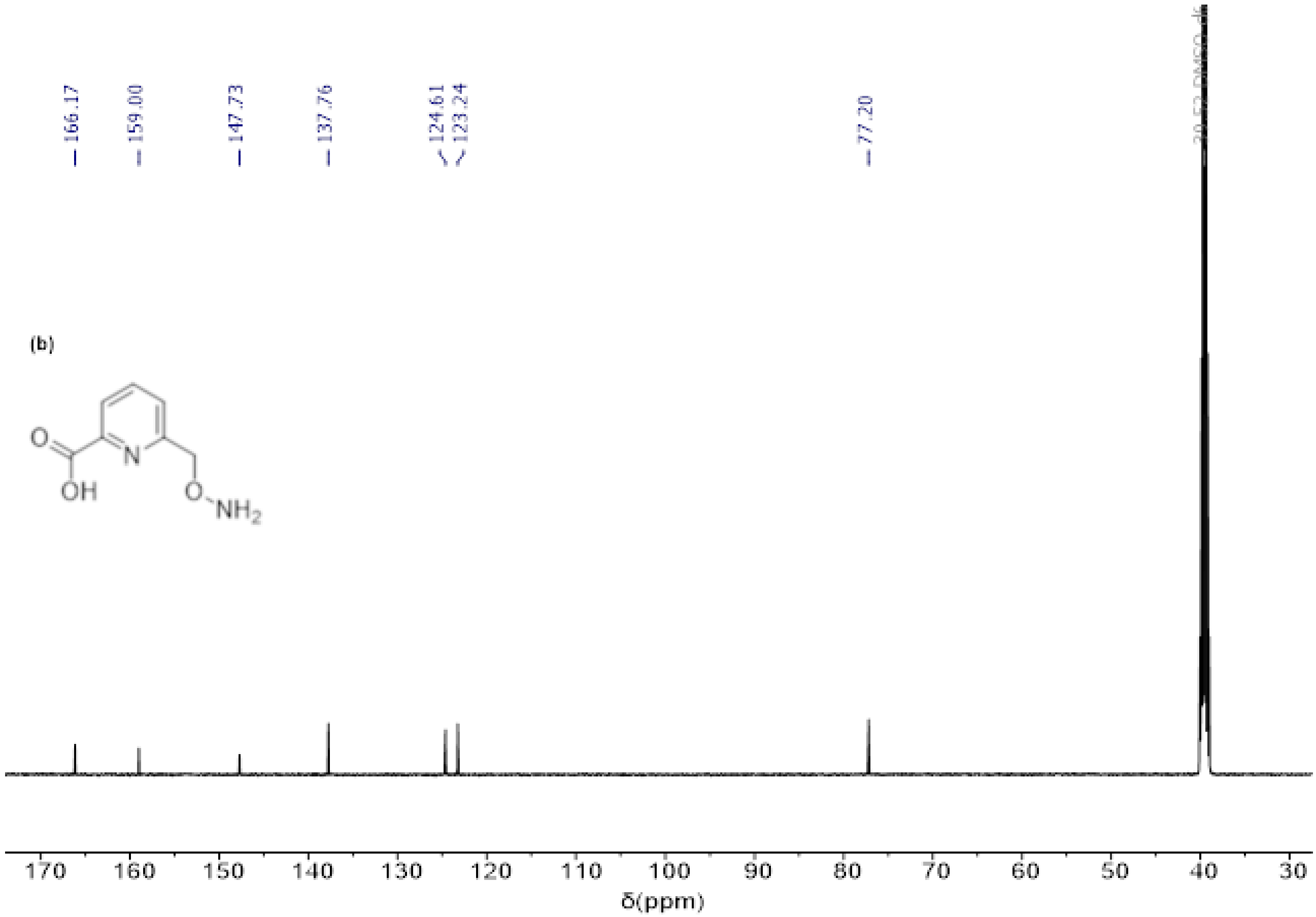

Figure S25. NMR spectra of compound **2c**. (a) ^{1}H NMR (500 MHz, DMSO-d_6). (b) ^{13}C NMR (126 MHz, DMSO-d_6).

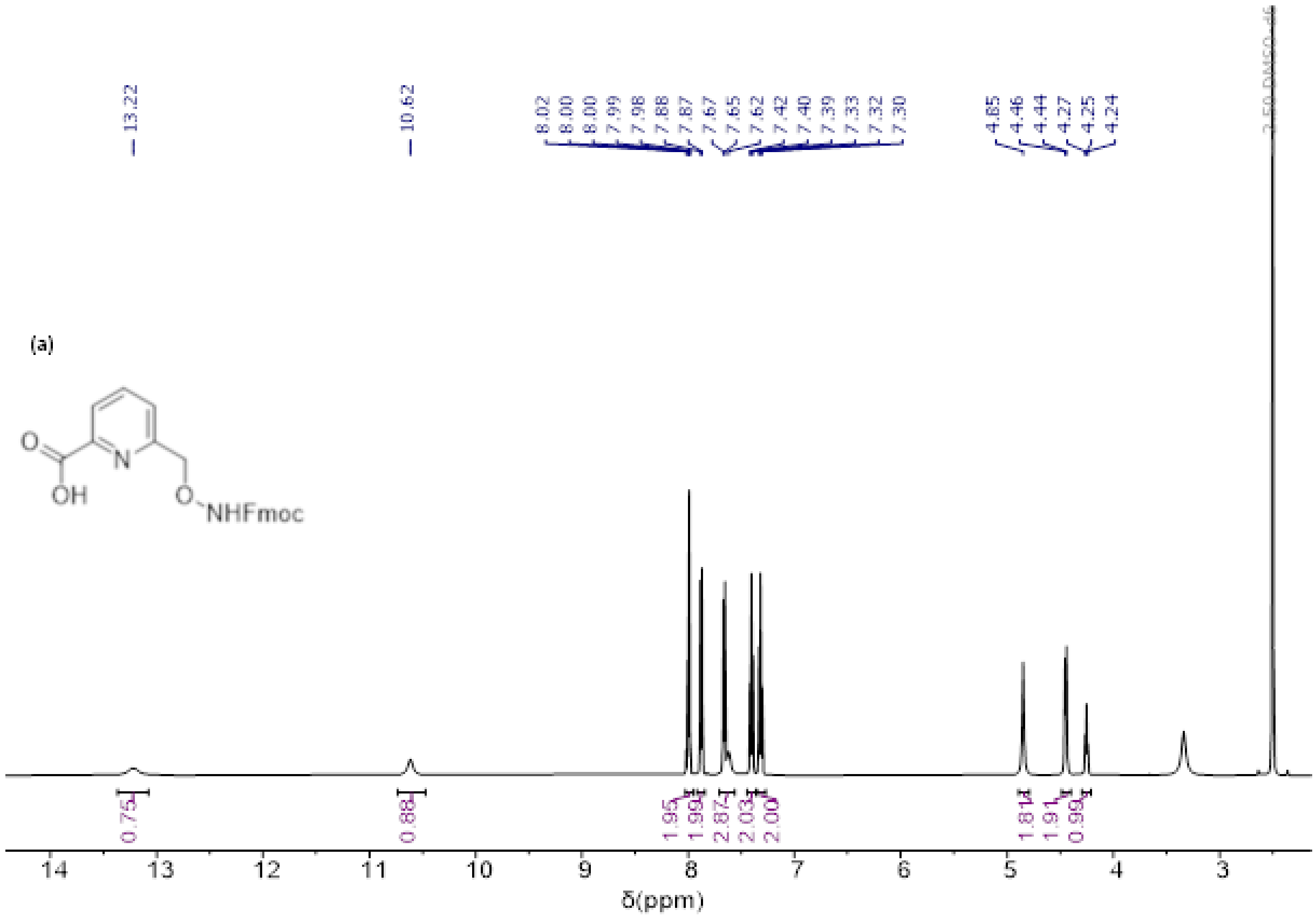

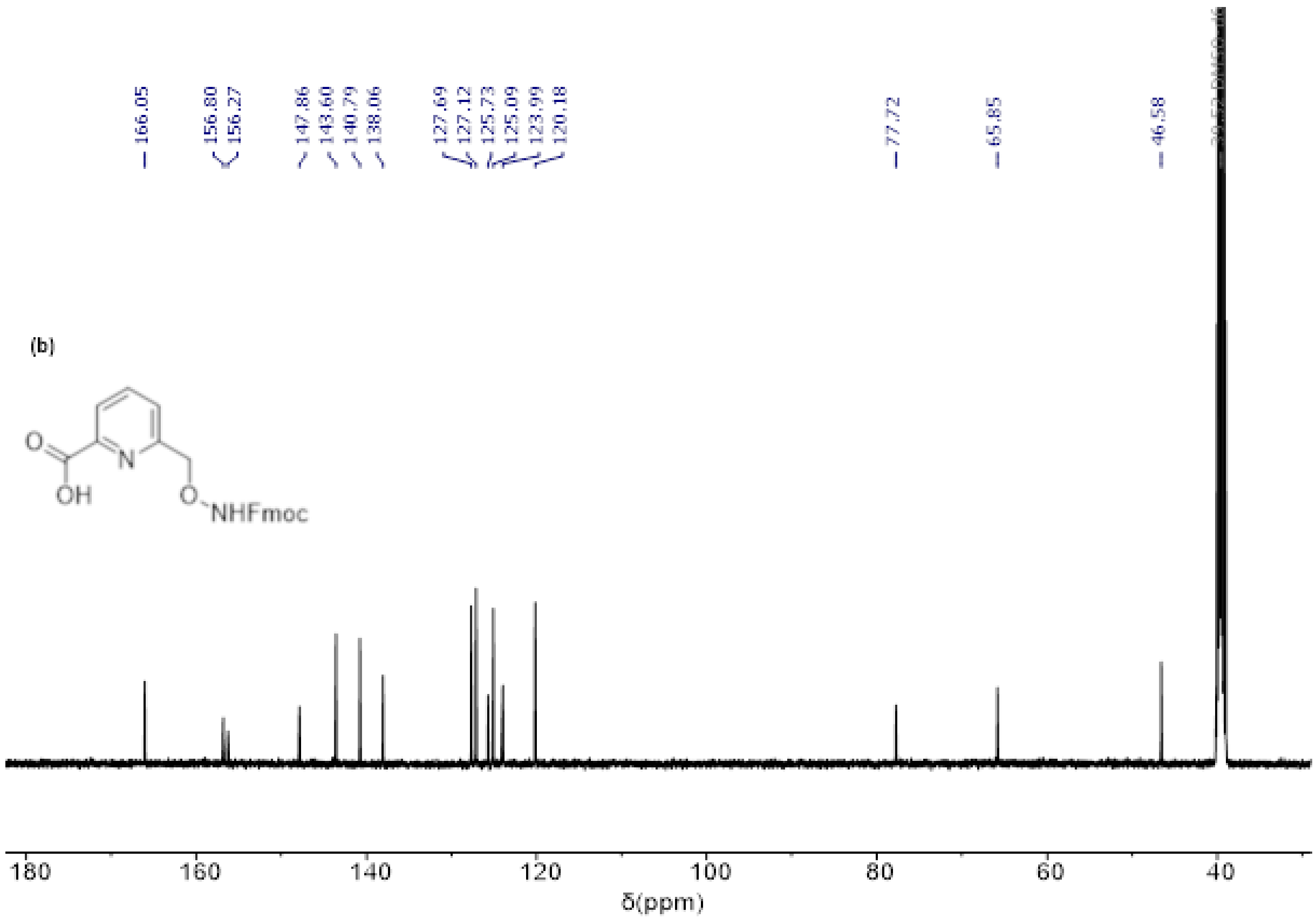

Figure S26. NMR spectra of compound **2**. (a) ^{1}H NMR (500 MHz, DMSO-d_6). (b) ^{13}C NMR (126 MHz, DMSO-d_6).

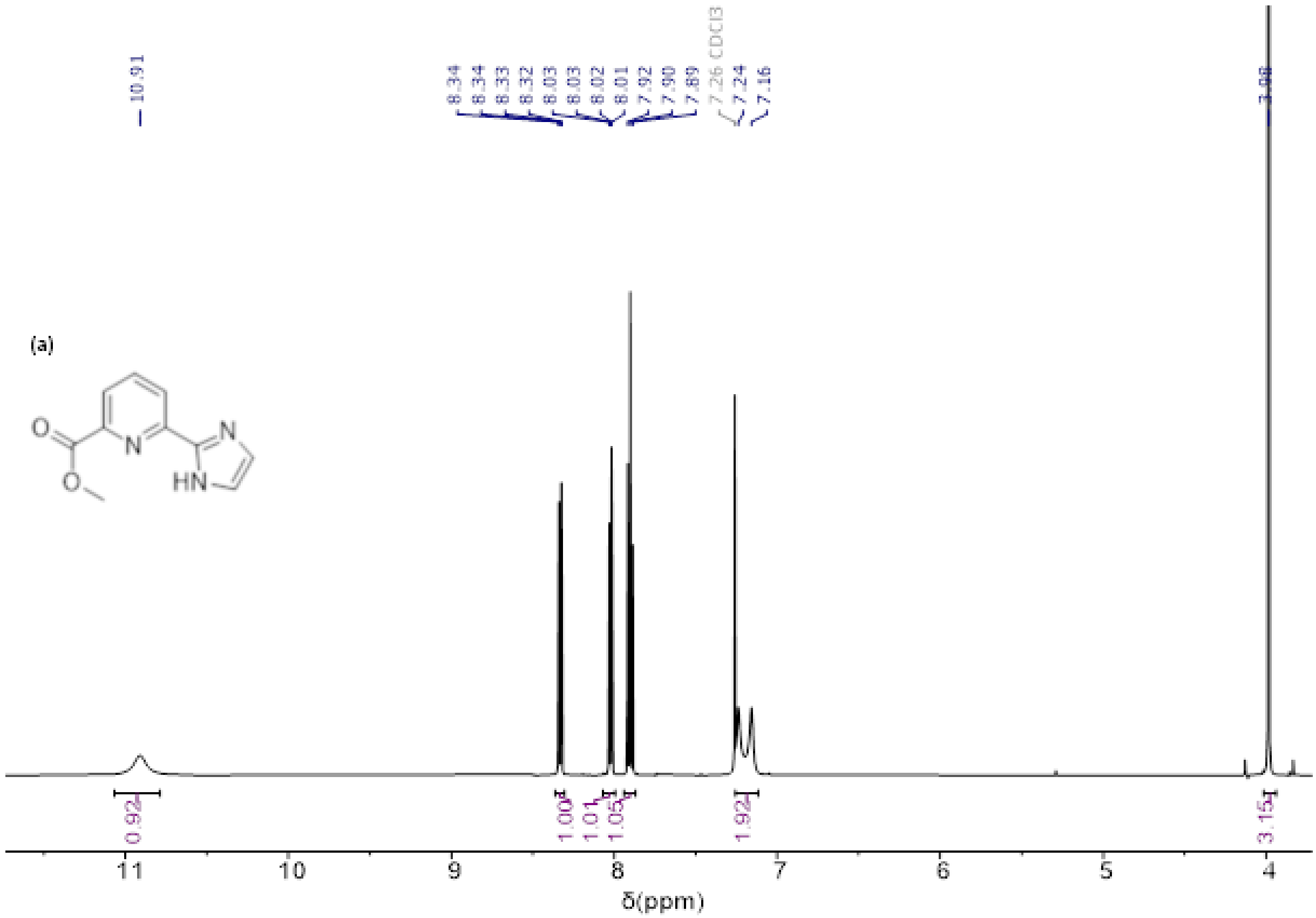

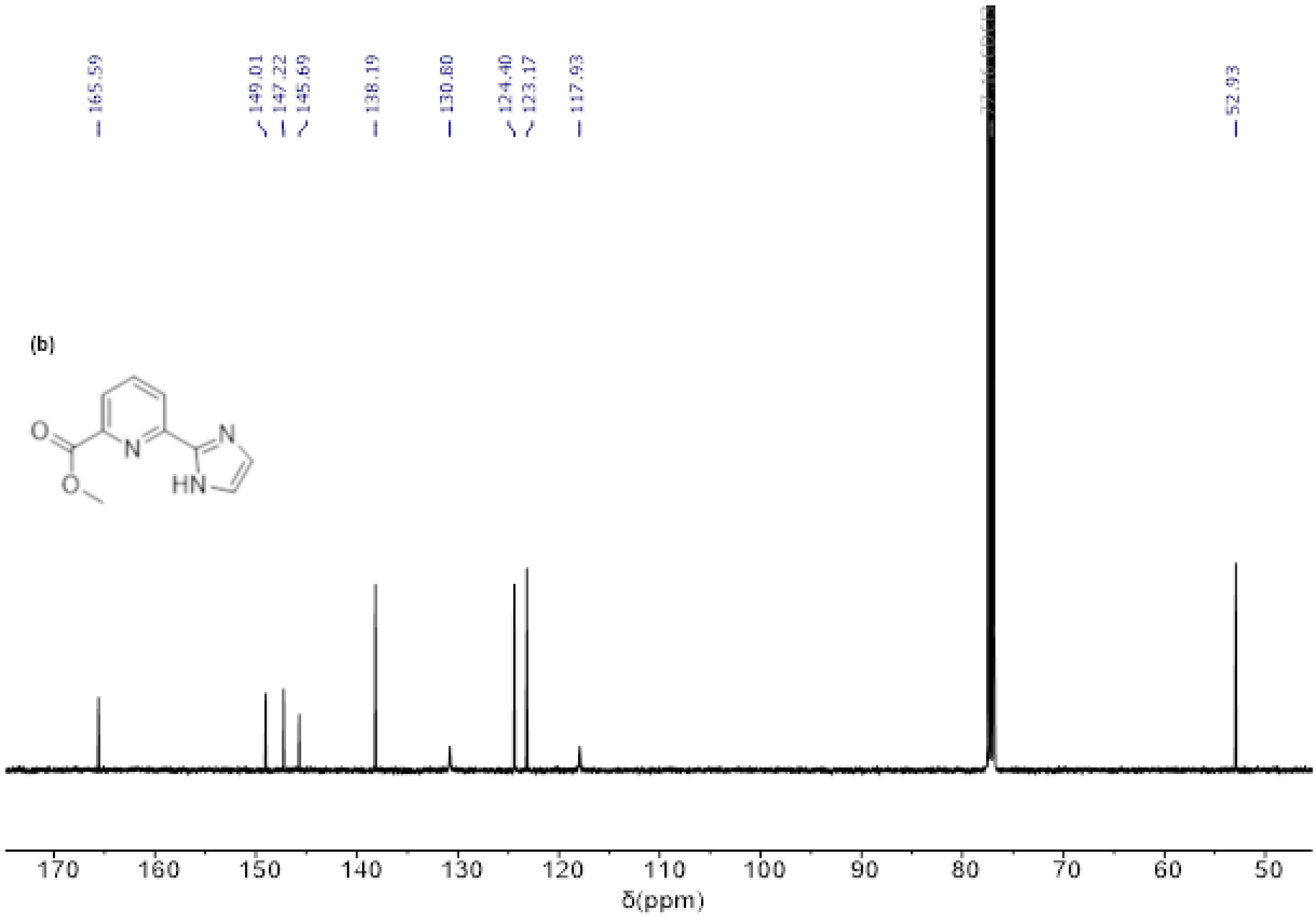

Figure S27. NMR spectra of compound **3a**. (a) ^{1}H NMR (500 MHz, $CDCl_3$). (b) ^{13}C NMR (126 MHz, $CDCl_3$).

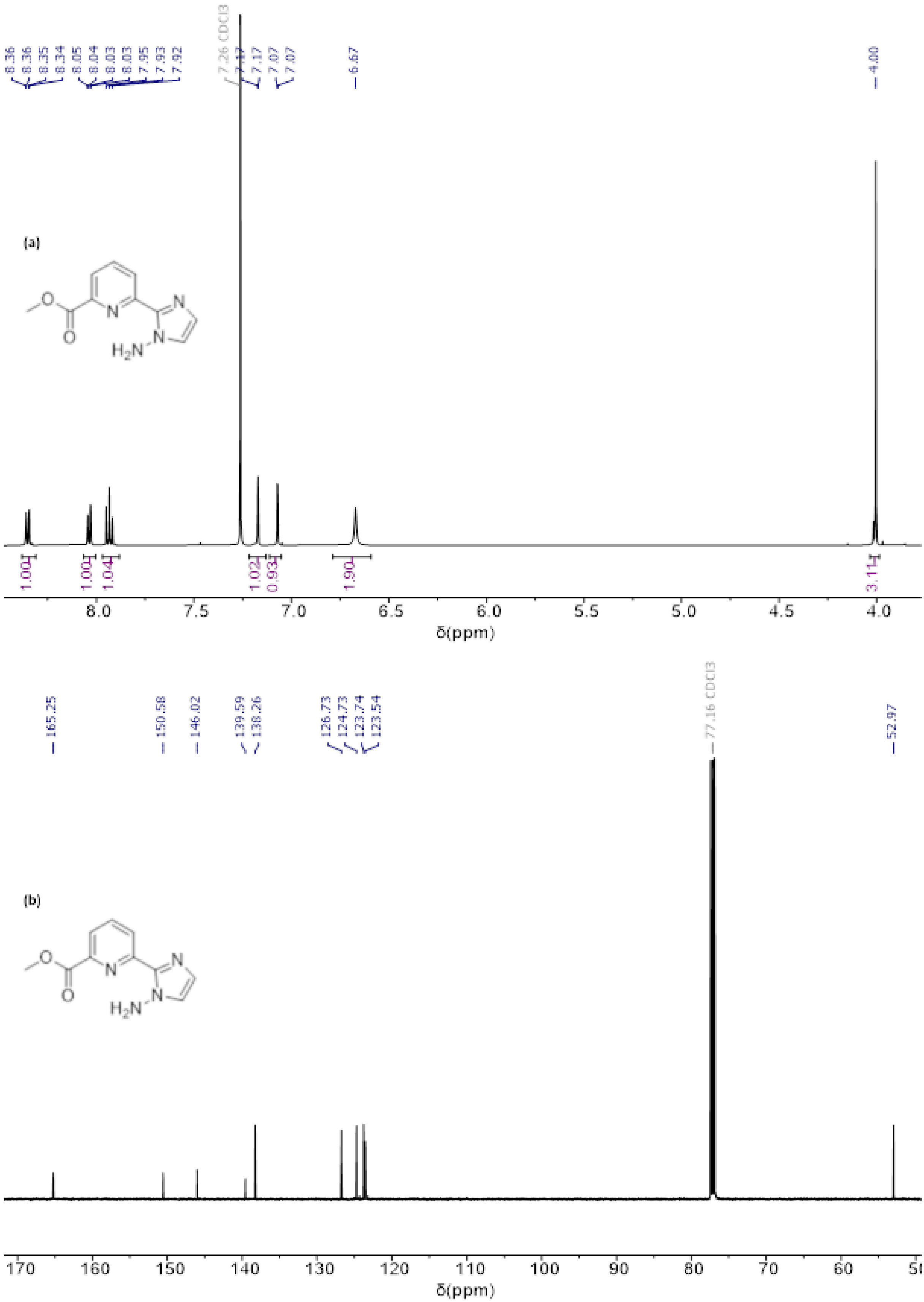

Figure S28. NMR spectra of compound **3b**. (a) ^{1}H NMR (500 MHz, $CDCl_3$). (b) ^{13}C NMR (126 MHz, $CDCl_3$).

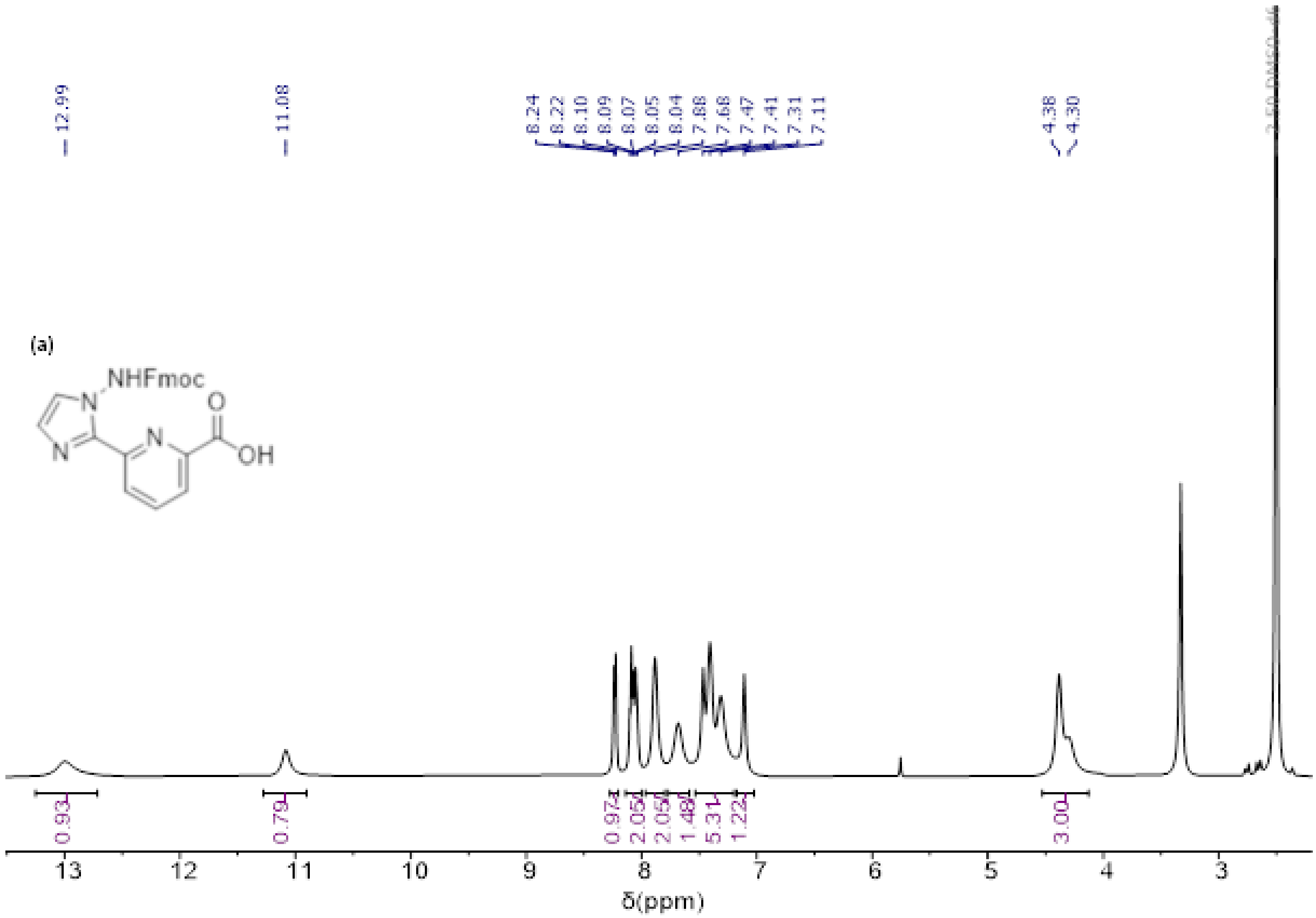

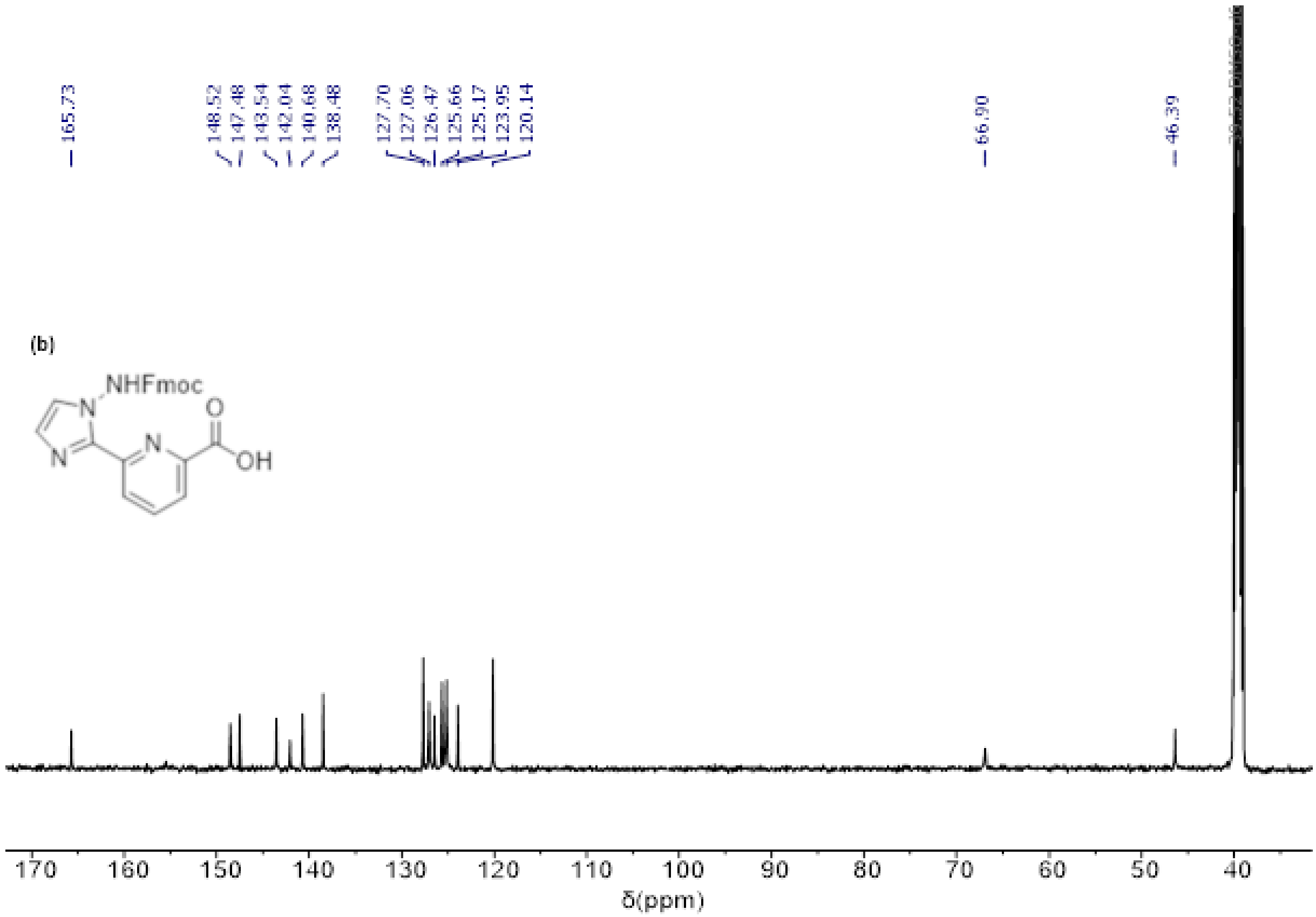

Figure S29. NMR spectra of compound **3.** (a) ^{1}H NMR (500 MHz, DMSO-d_6). (b) ^{13}C NMR (126 MHz, DMSO-d_6).

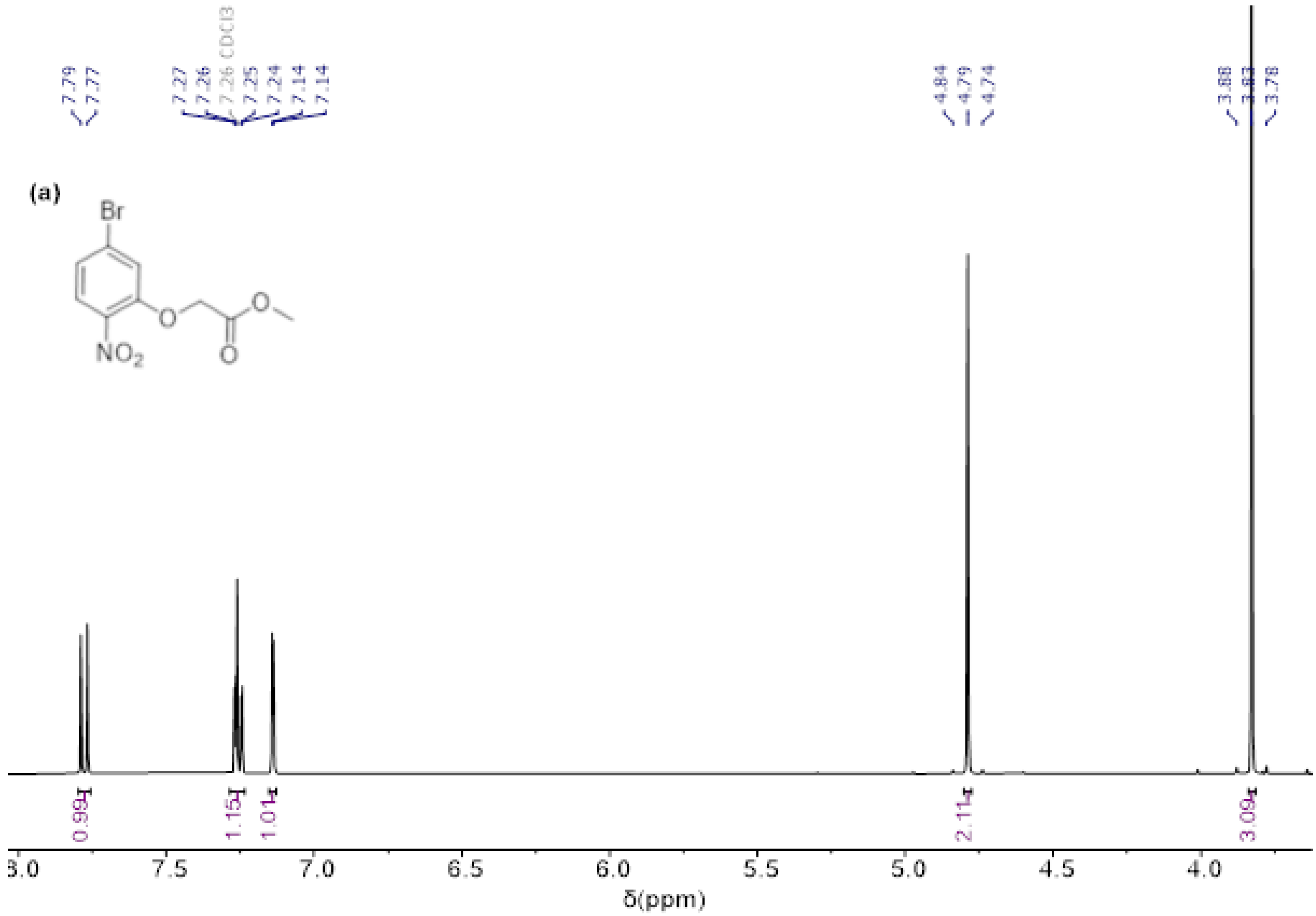

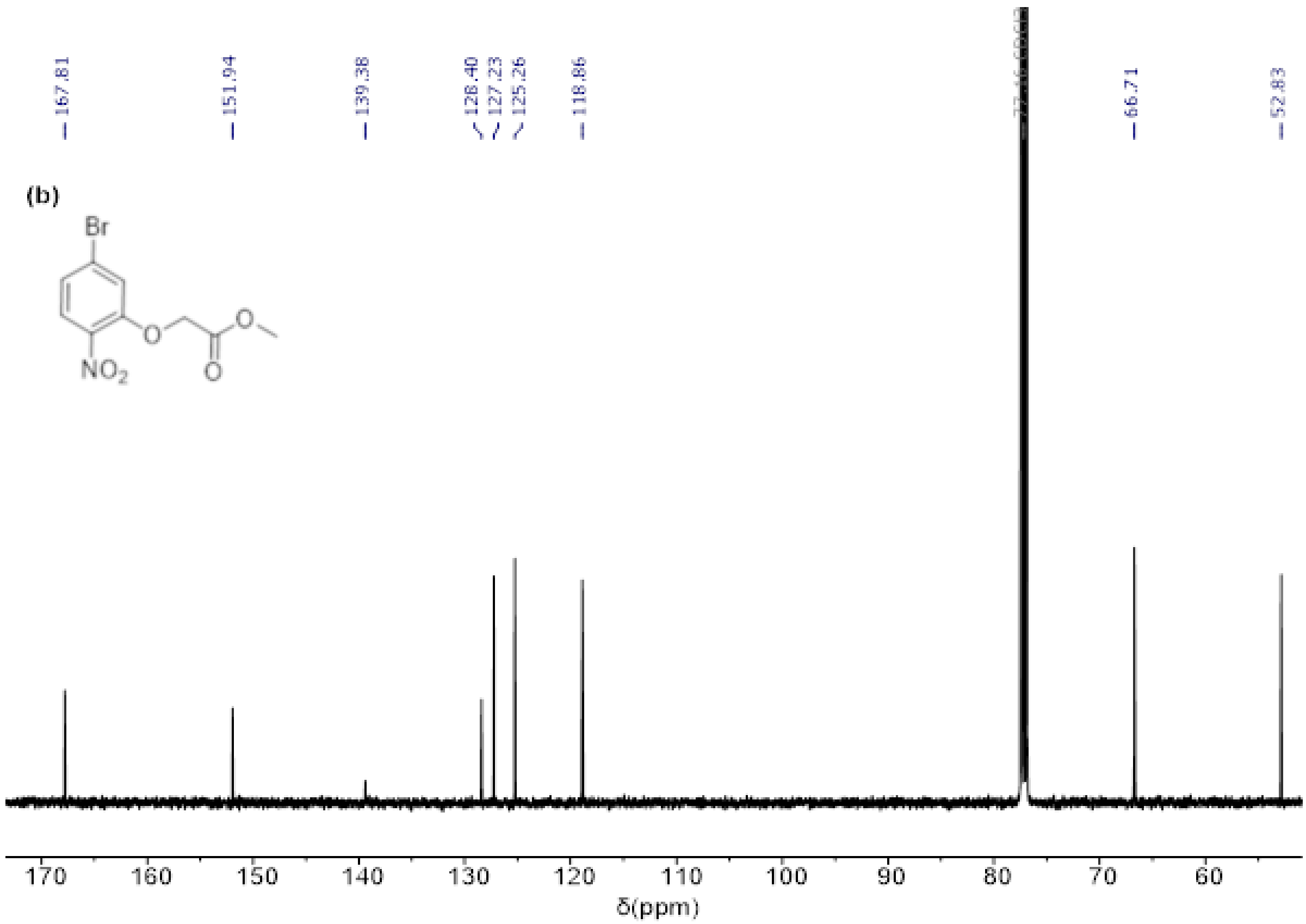

Figure S30. NMR spectra of compound **4a**. (a) ^{1}H NMR (500 MHz, $CDCl_3$). (b) ^{13}C NMR (126 MHz, $CDCl_3$).

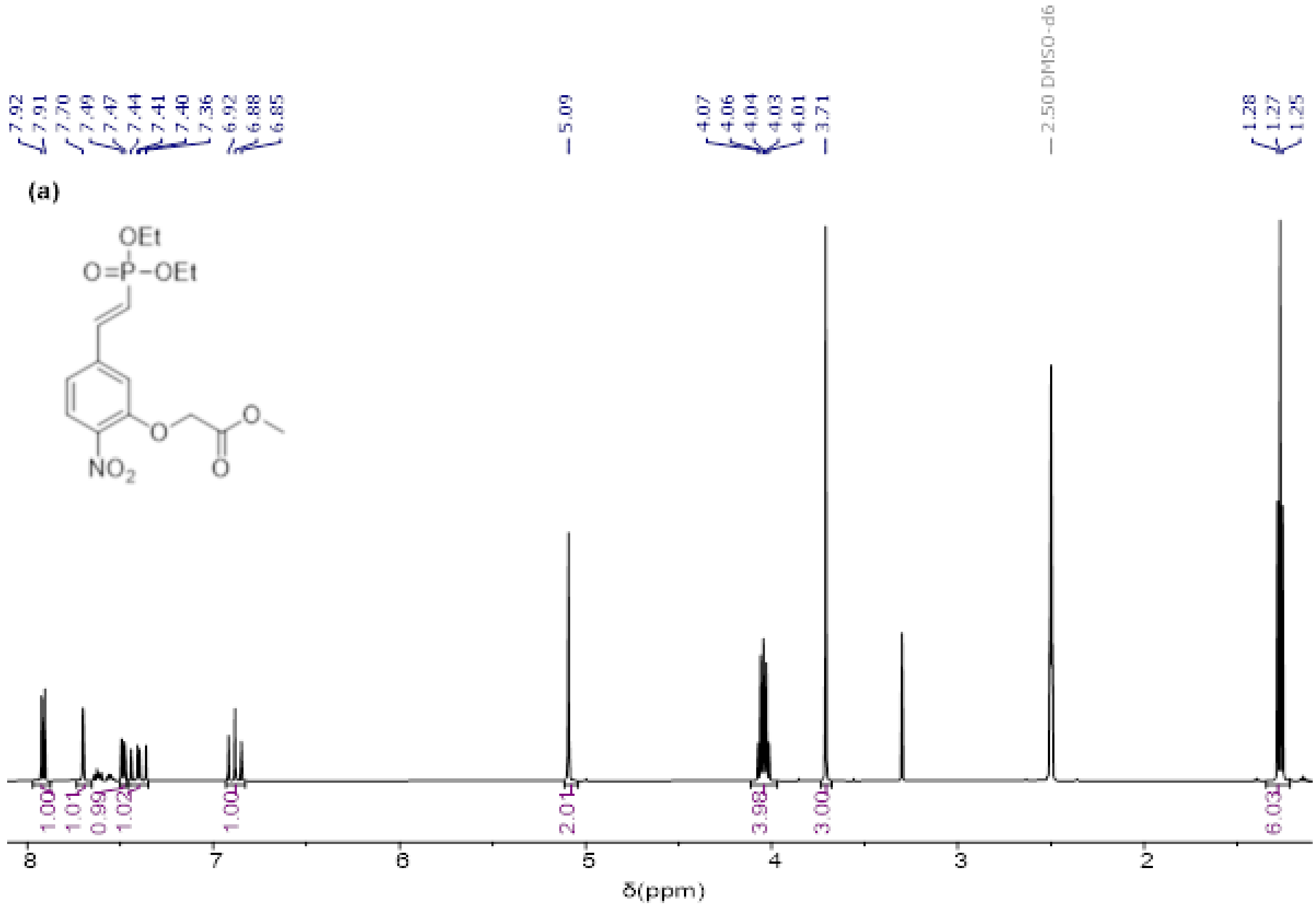

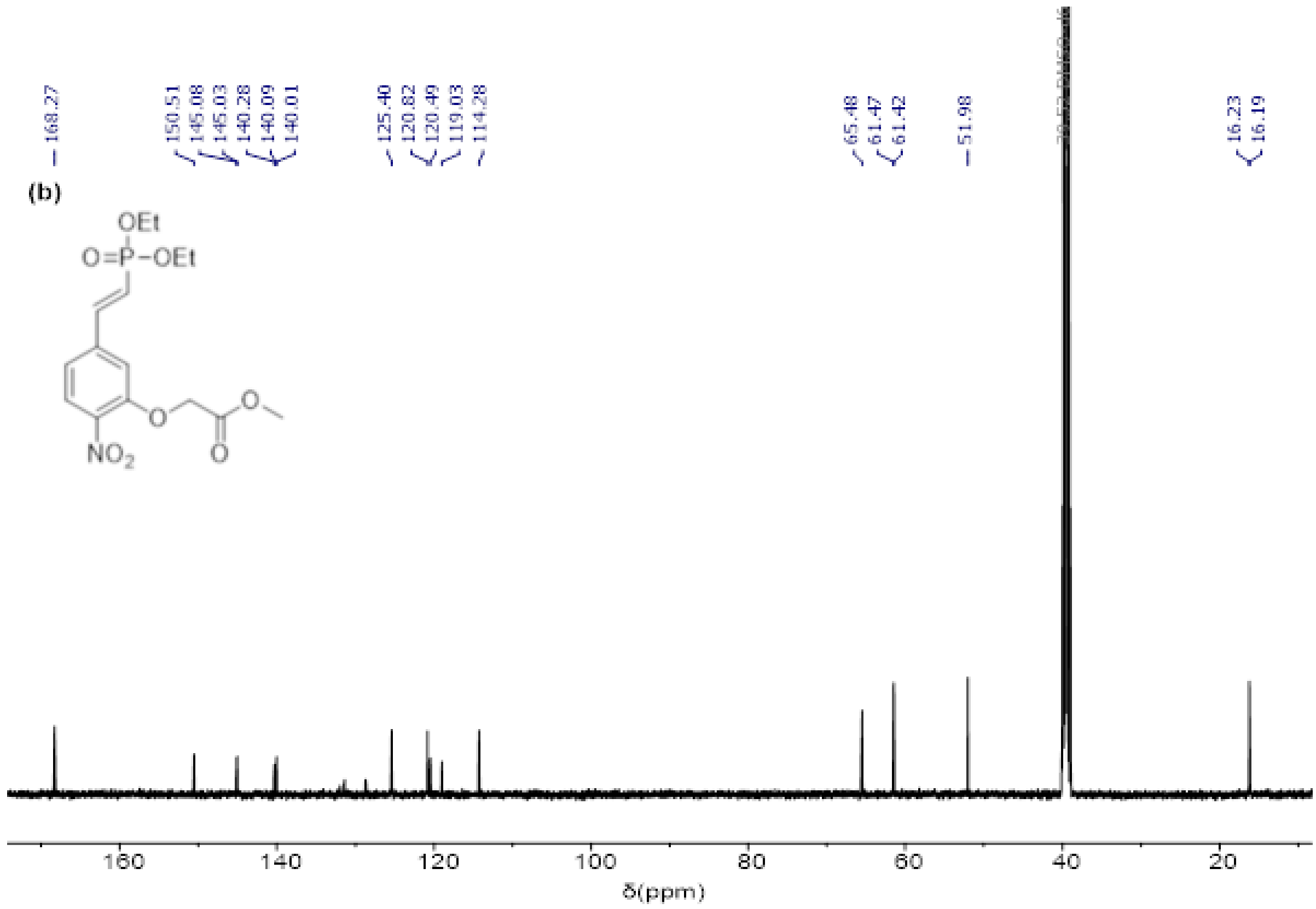

Figure S31. NMR spectra of compound **4b**. (a) ^{1}H NMR (500 MHz, DMSO-d_6). (b) ^{13}C NMR (126 MHz, DMSO-d_6).

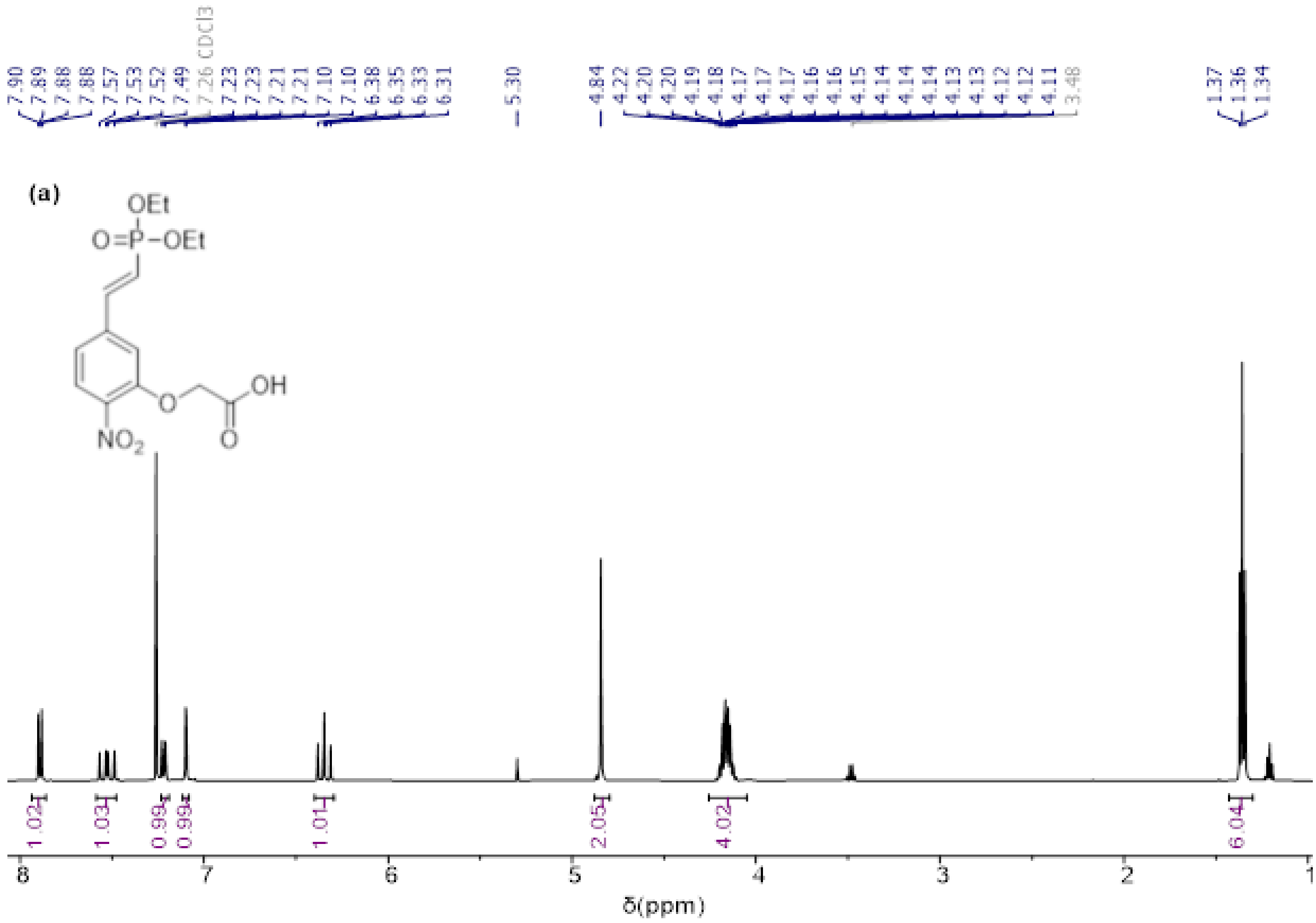

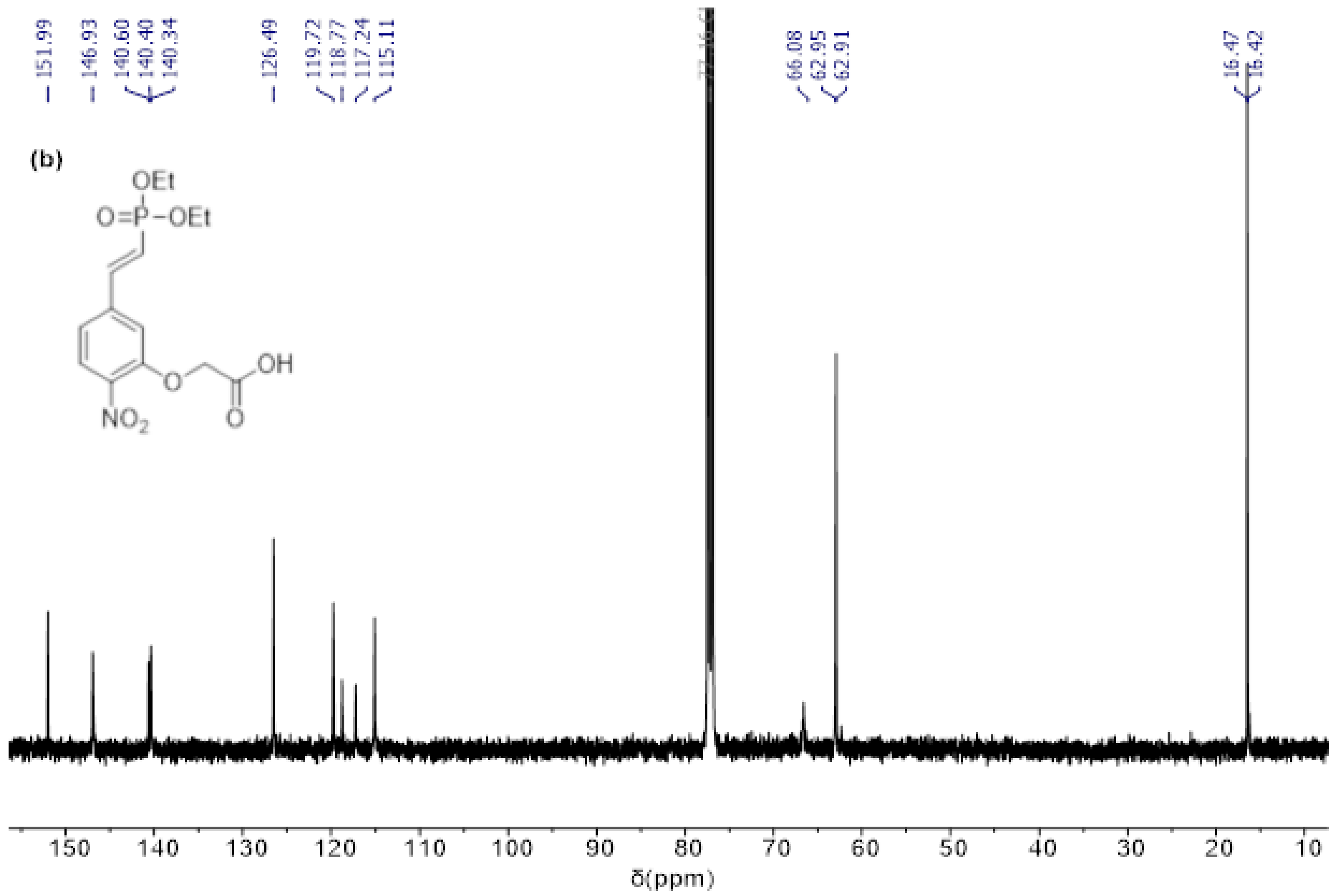

Figure S32. NMR spectrum of Compound **4c**. (a) ^{1}H NMR (500 MHz, $CDCl_3$). (b) ^{13}C NMR (126 MHz, $CDCl_3$).

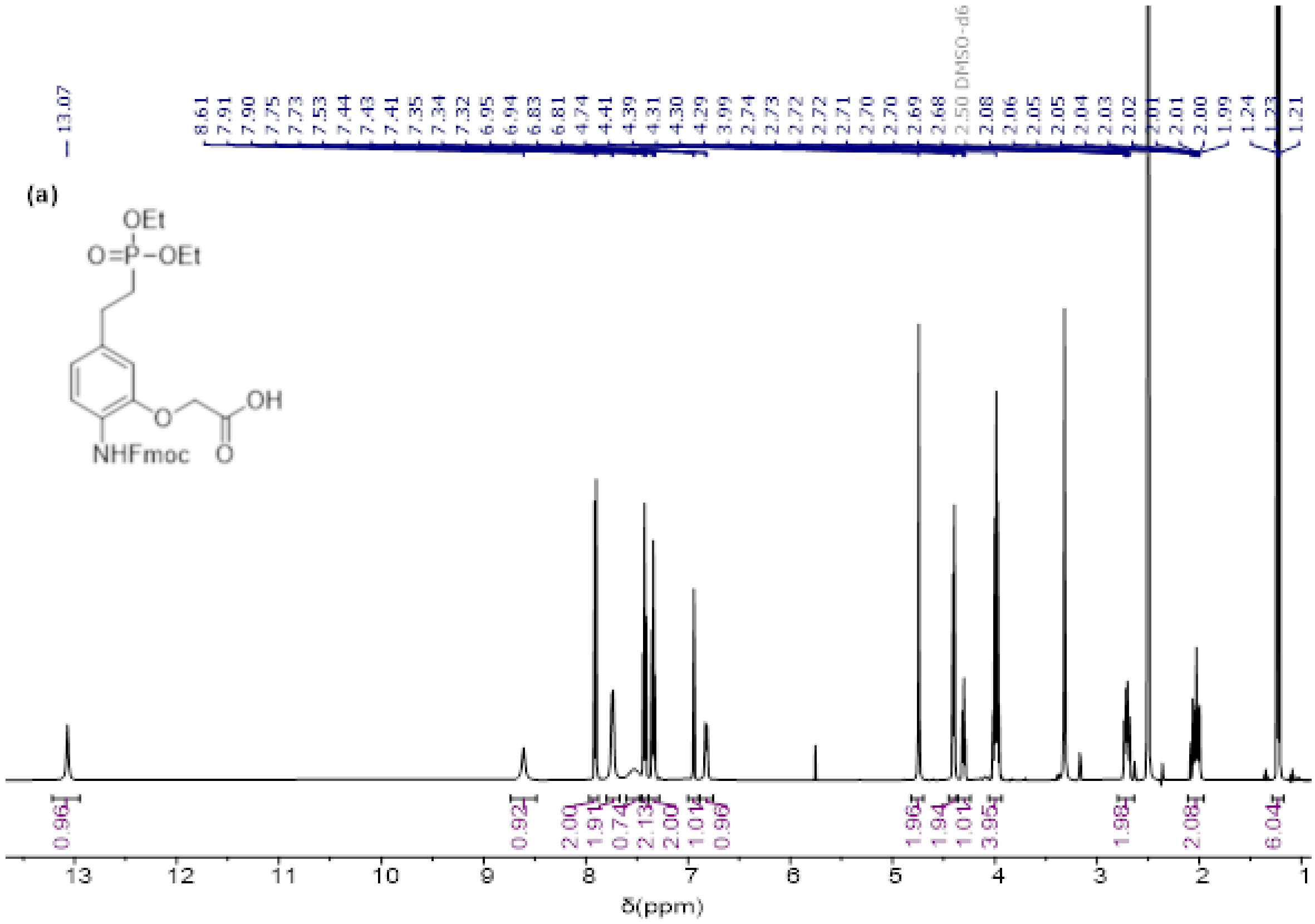

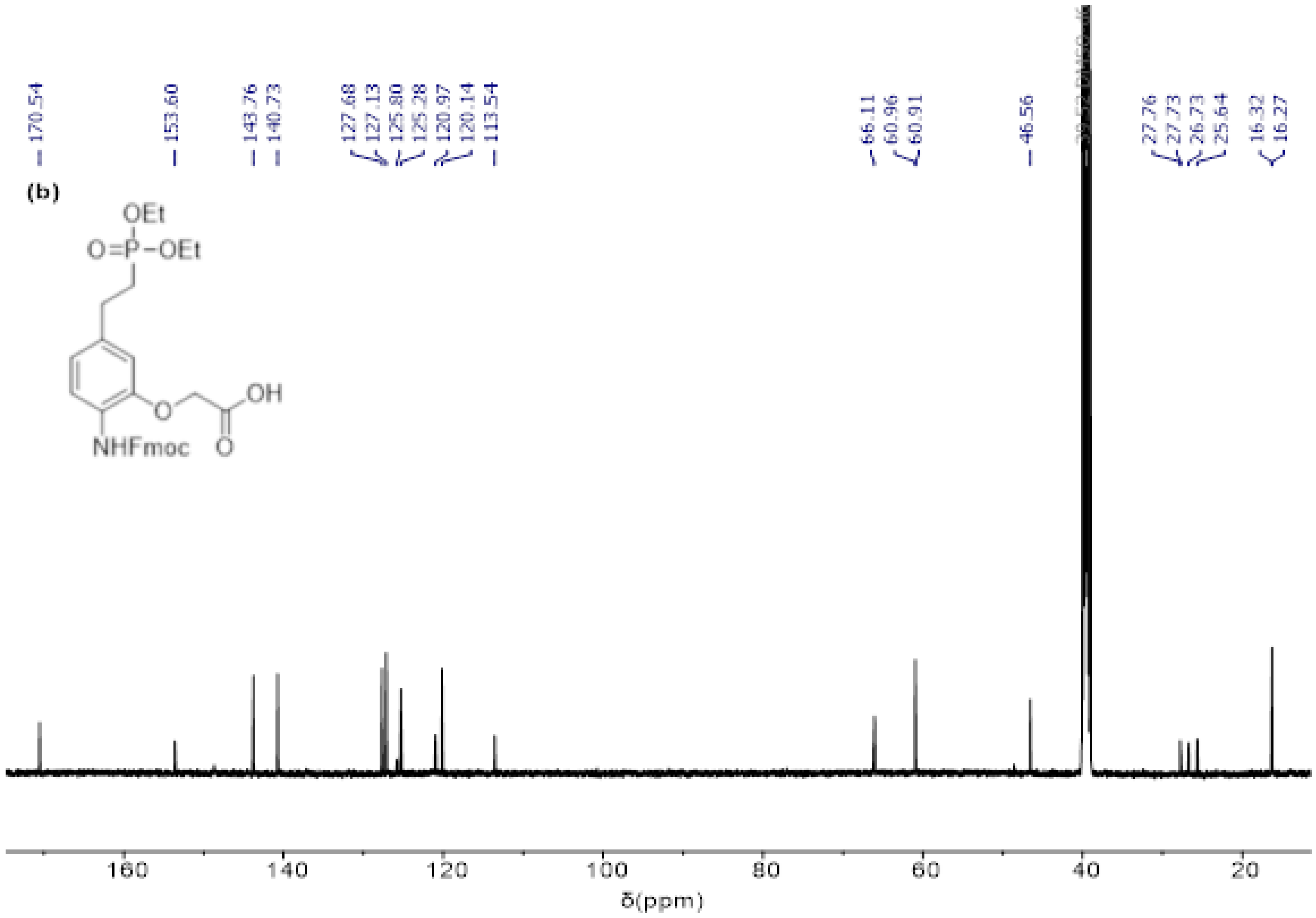

Figure S33. NMR spectra of Compound **4**. (a) ^{1}H NMR (500 MHz, DMSO-d_6). (b) ^{13}C NMR (126 MHz, DMSO-d_6).

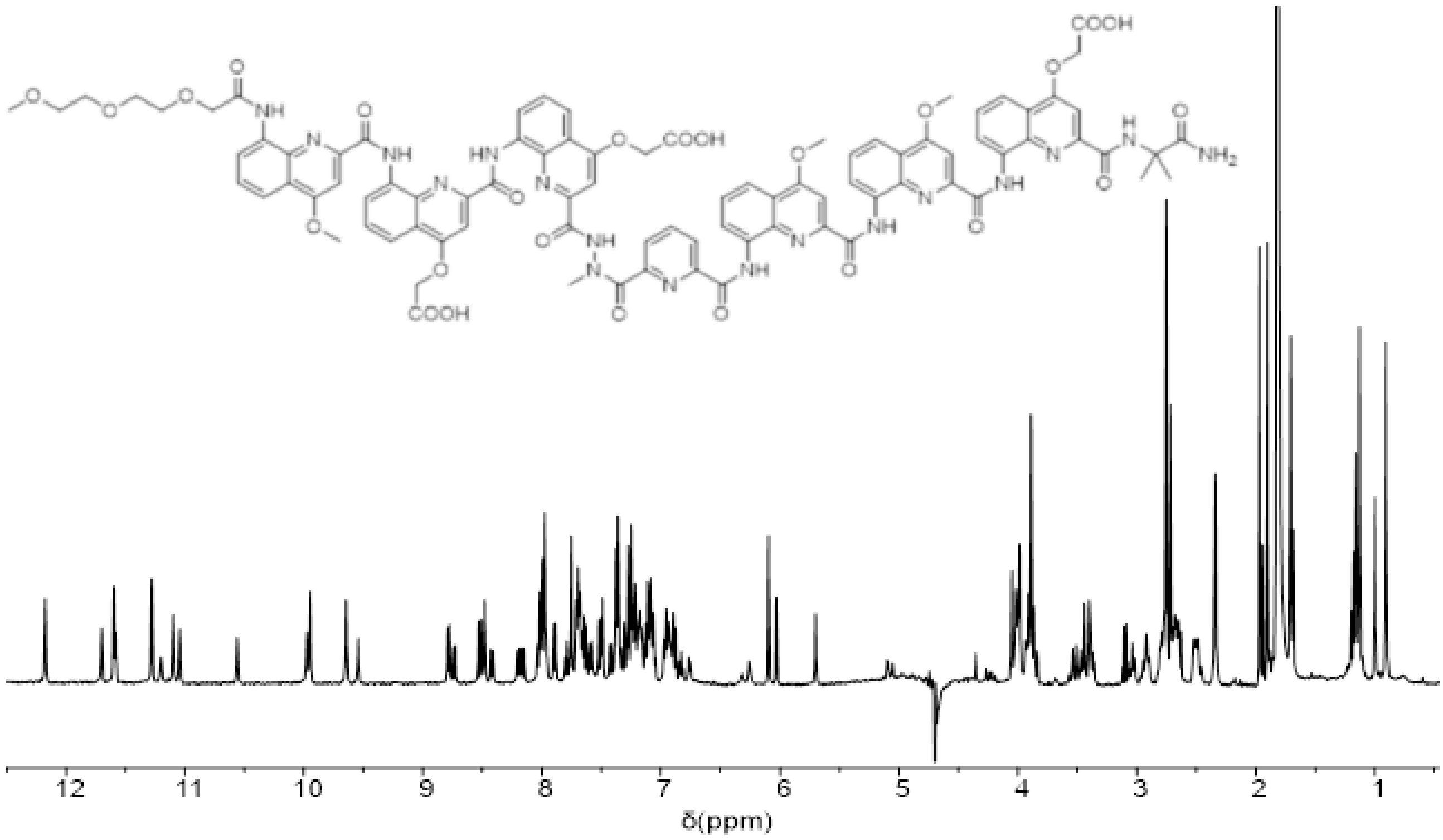

Figure S34. ^{1}H NMR spectrum of oligomer **5**. (500 MHz, 50 mM NH_4HCO_3, pH 8.5, H_2O/D_2O 9:1 v/v, at 25°C, water suppression).

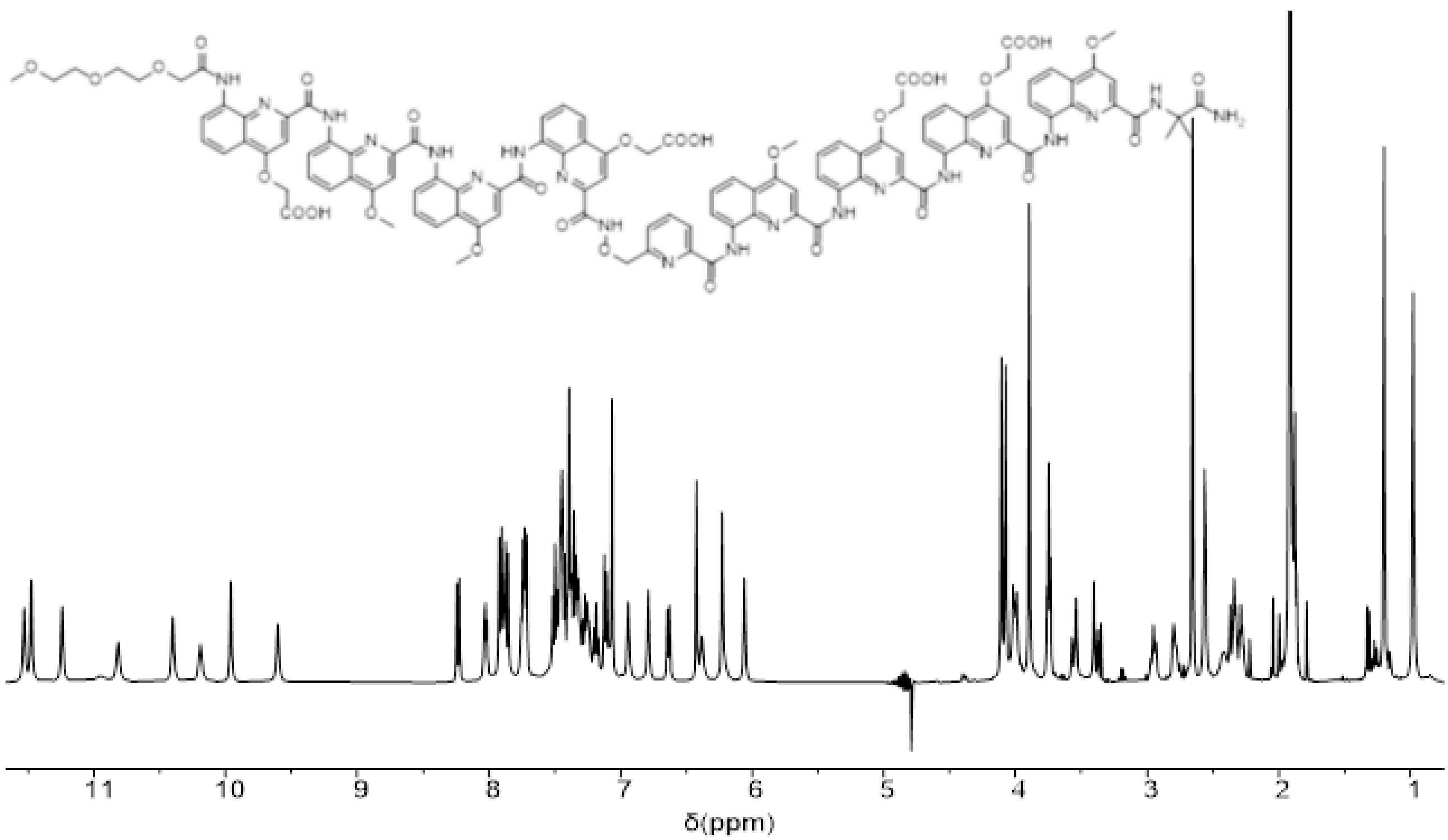

Figure S35. ^{1}H NMR spectra of oligomer **6**. (500 MHz, 50 mM NH_4HCO_3, pH 8.5, H_2O/D_2O 9:1 v/v, at 25°C, water suppression).

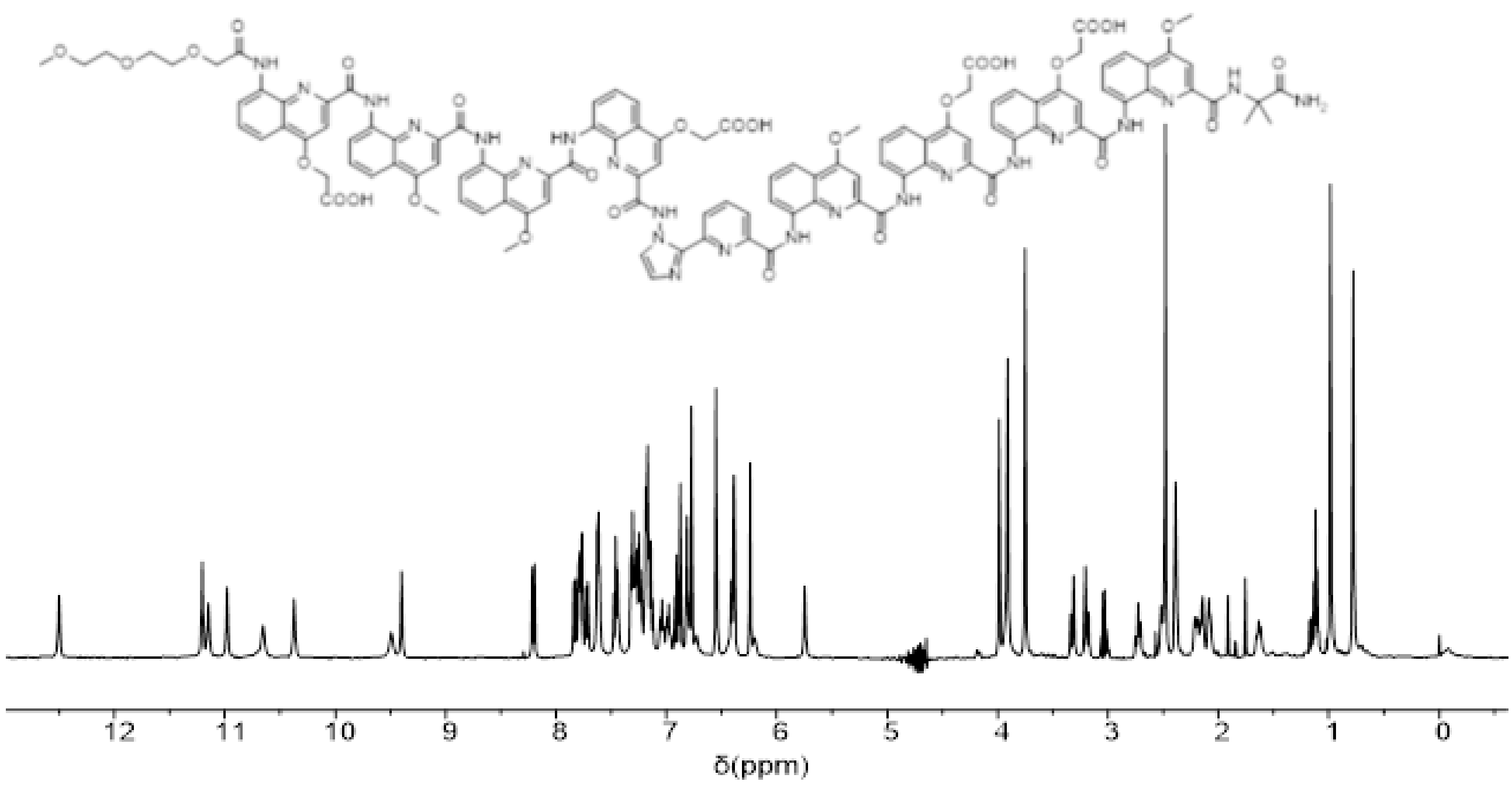

Figure S36. ^{1}H NMR spectra of oligomer **7**. (500 MHz, 50 mM NH_4HCO_3, pH 8.5, H_2O/D_2O 9:1 v/v, at 25°C, water suppression).

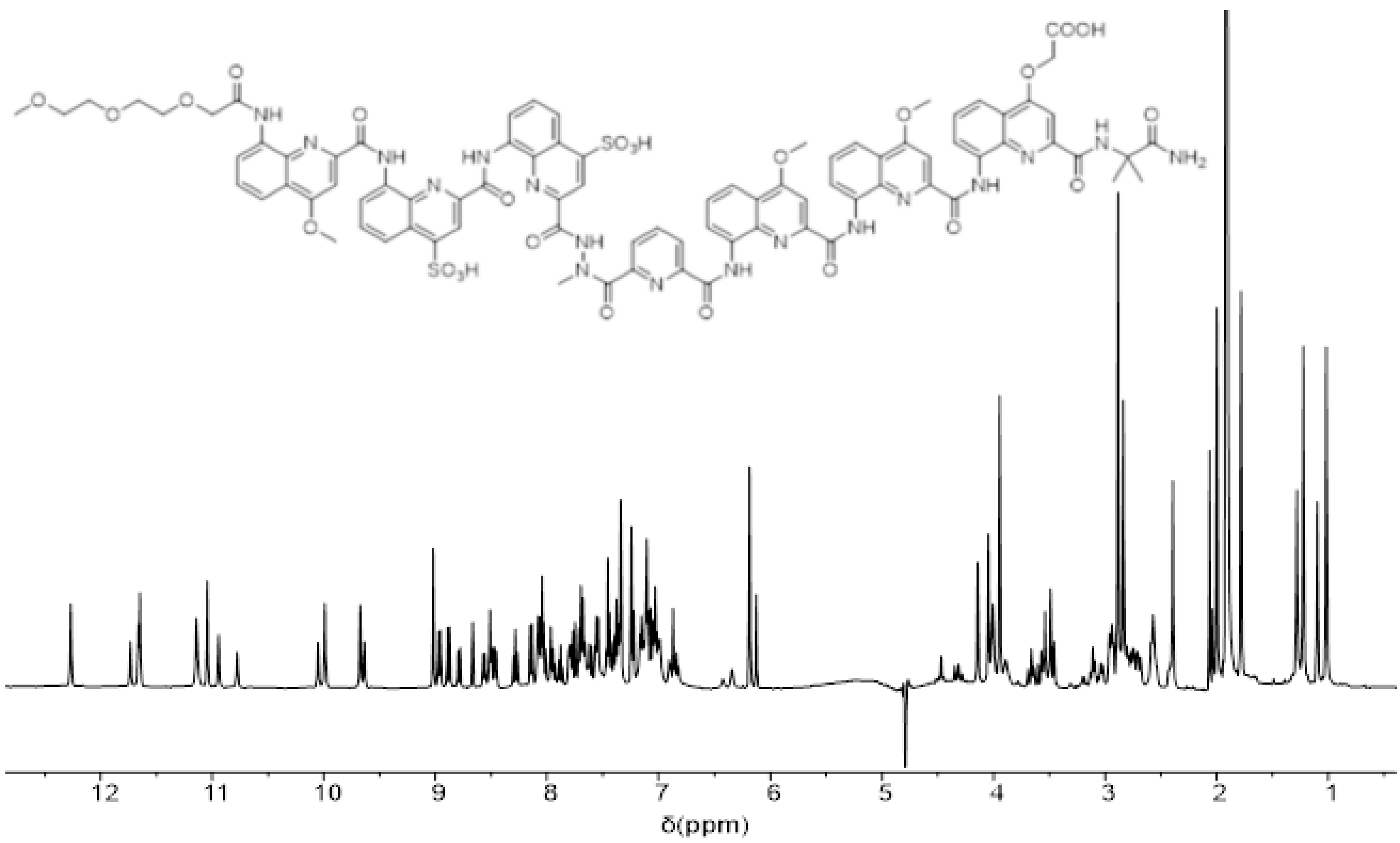

Figure S37. ^{1}H NMR spectrum of **oligomer 8**. (500 MHz, 50 mM NH_4HCO_3, pH 8.5, H_2O/D_2O 9:1 v/v, at 25°C, water suppression).

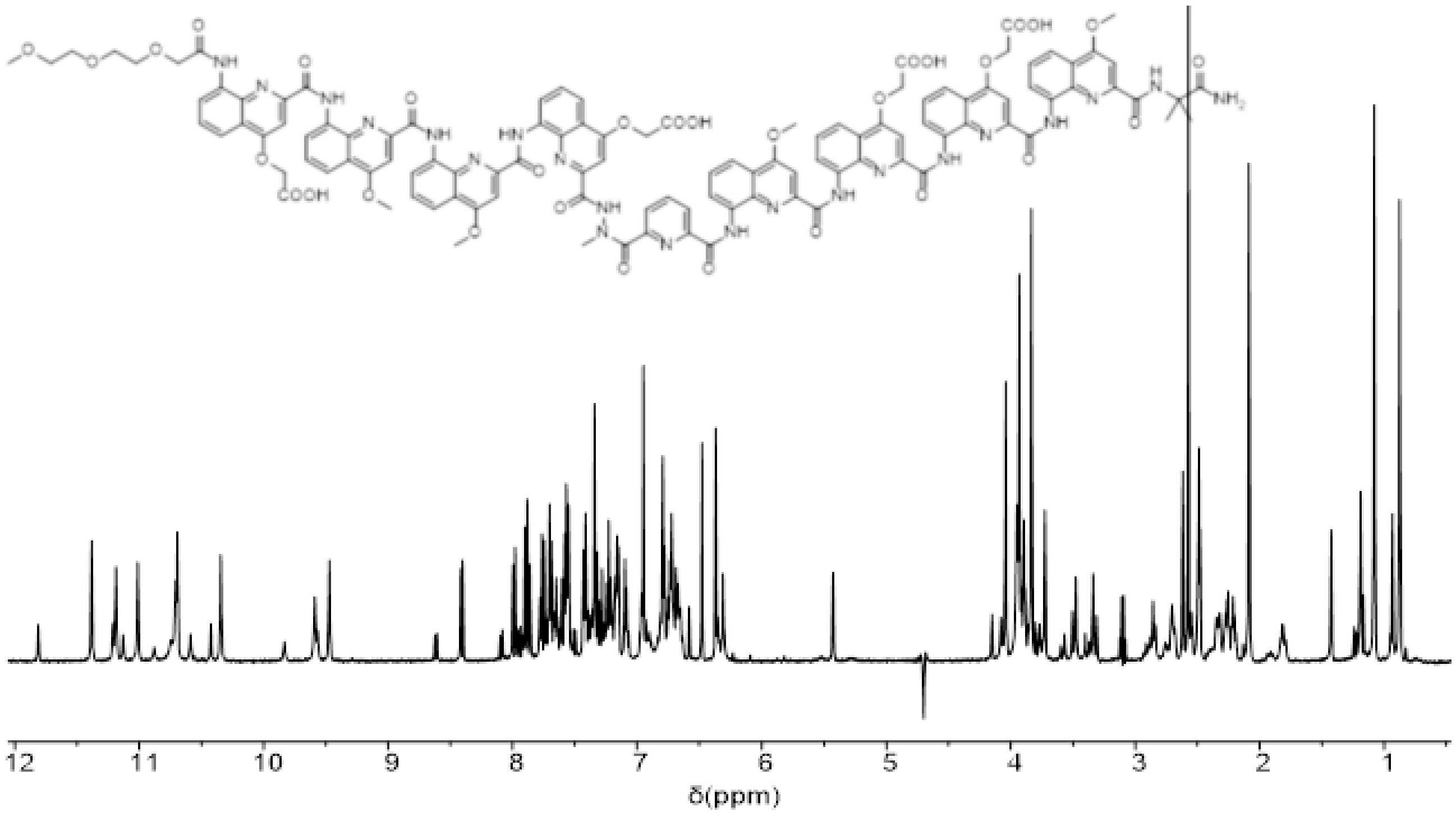

Figure S38. ^{1}H NMR spectra of oligomer **9**. (a) Part of the ^{1}H DOSY NMR. (500 MHz, 50 mM NH_4HCO_3, pH 8.5, H_2O/D_2O 9:1 v/v, at 25°C, water suppression).

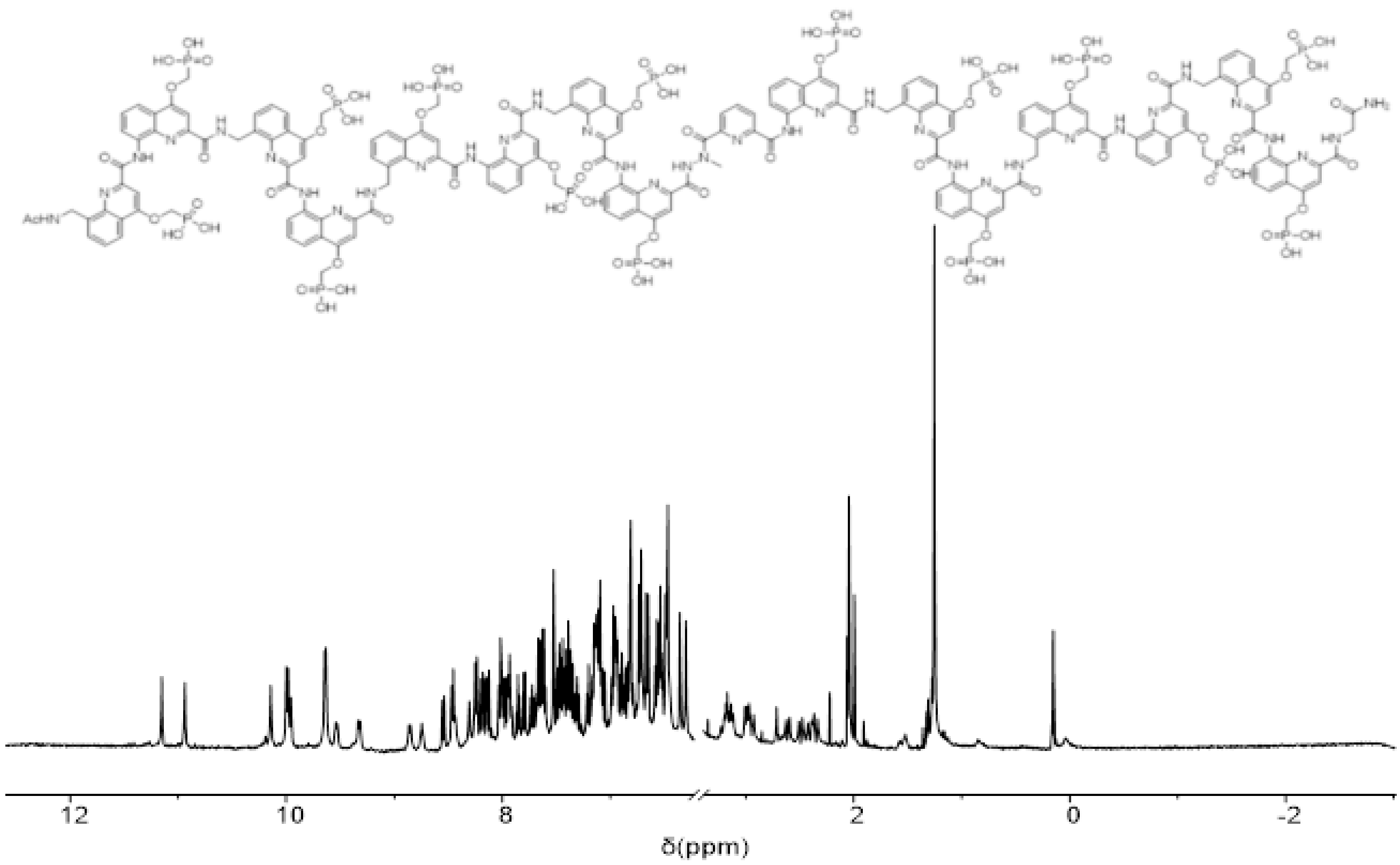

Figure S39. ^{1}H NMR spectra of oligomer **10**. (500 MHz, 50 mM NH_4HCO_3, pH 8.5, H_2O/D_2O 9:1 v/v, at 25°C, water suppression).

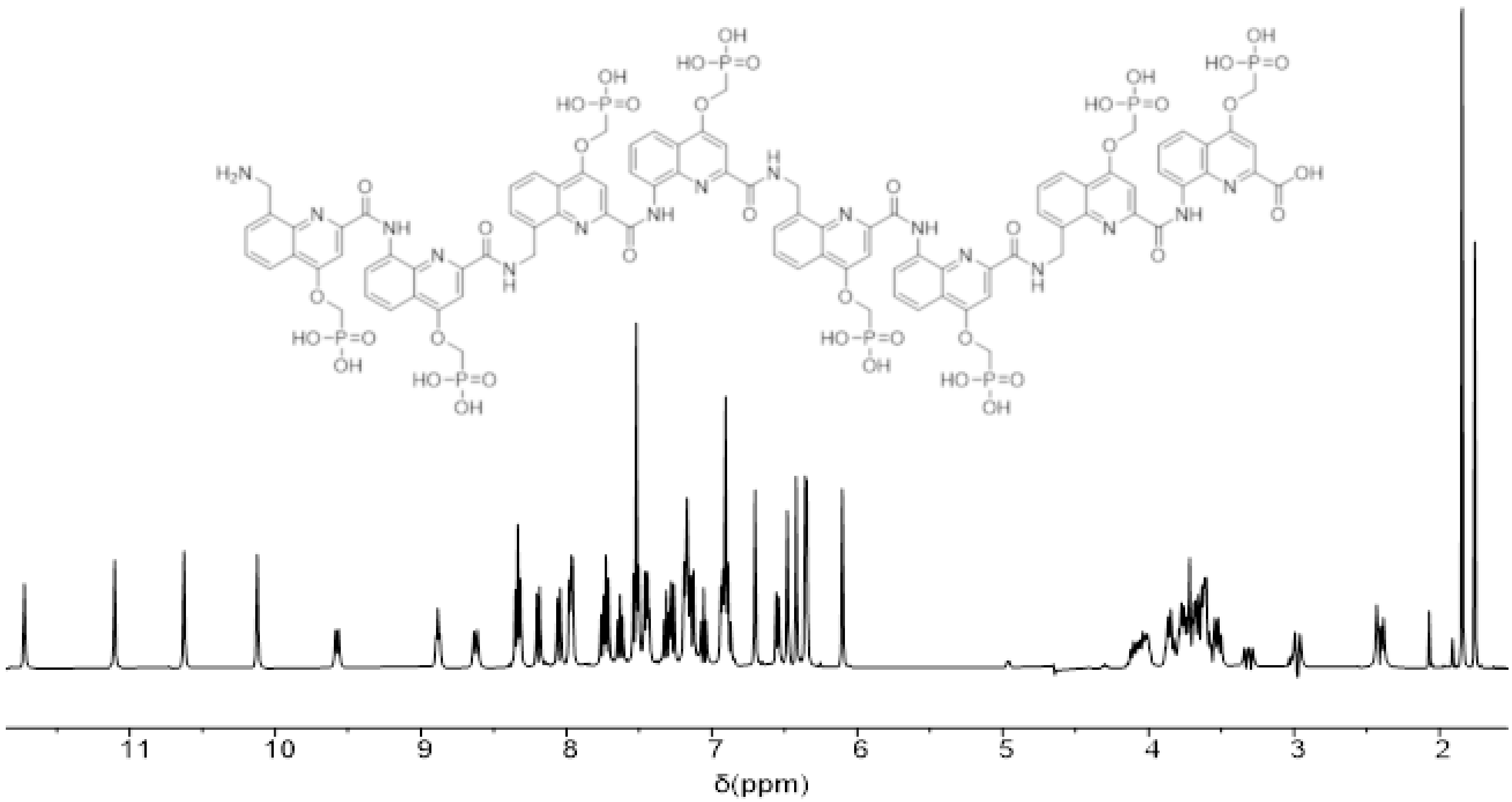

Figure S40. ^{1}H NMR spectra of **oligomer 11**. (500 MHz, 50 mM NH_4HCO_3, pH 8.5, H_2O/D_2O 9:1 v/v, at 25°C, water suppression).

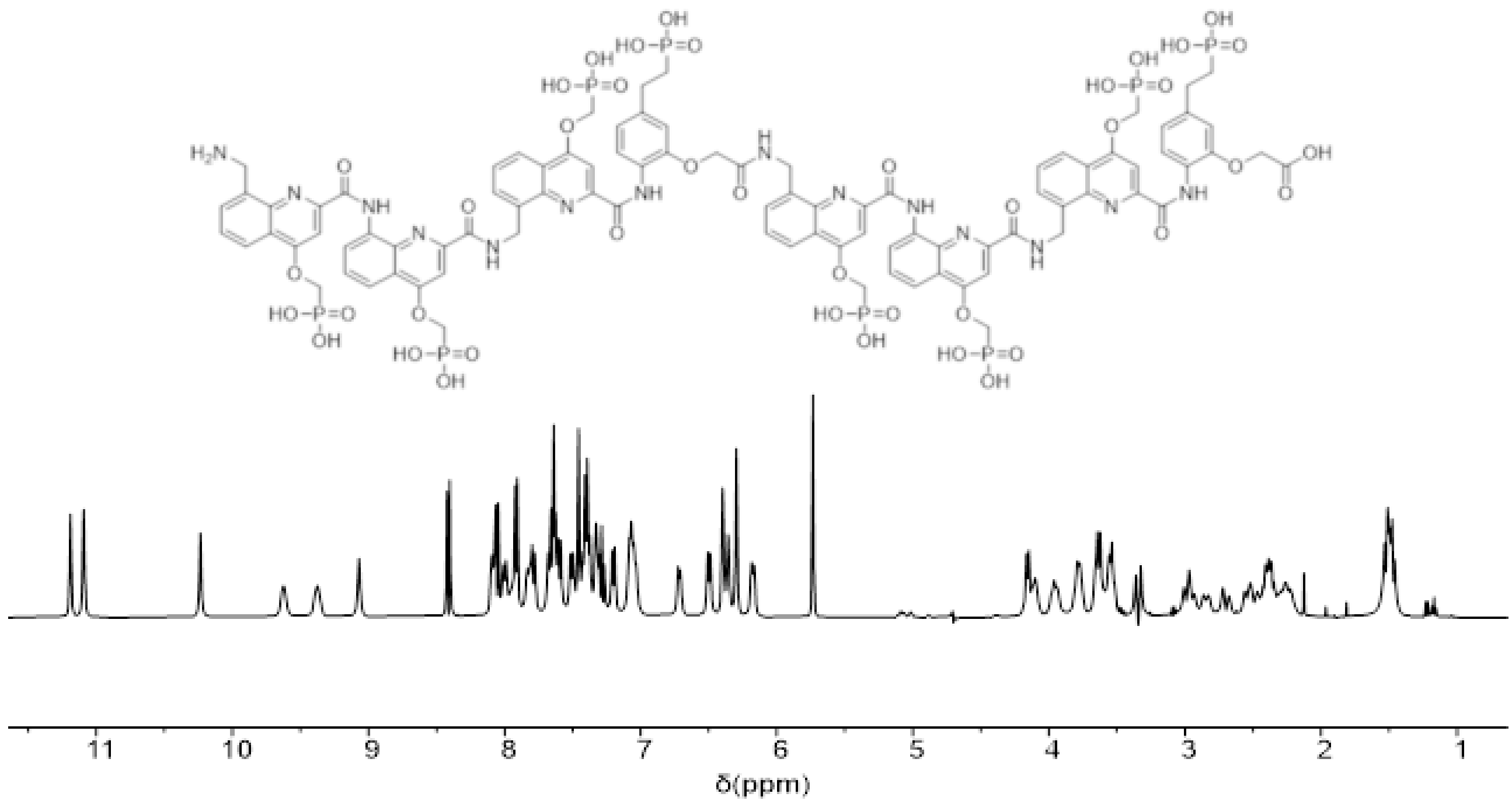

Figure S41. ^{1}H NMR spectra of **oligomer 12**. (500 MHz, 50 mM NH_4HCO_3, pH 8.5, H_2O/D_2O 9:1 v/v, at 25°C, water suppression).

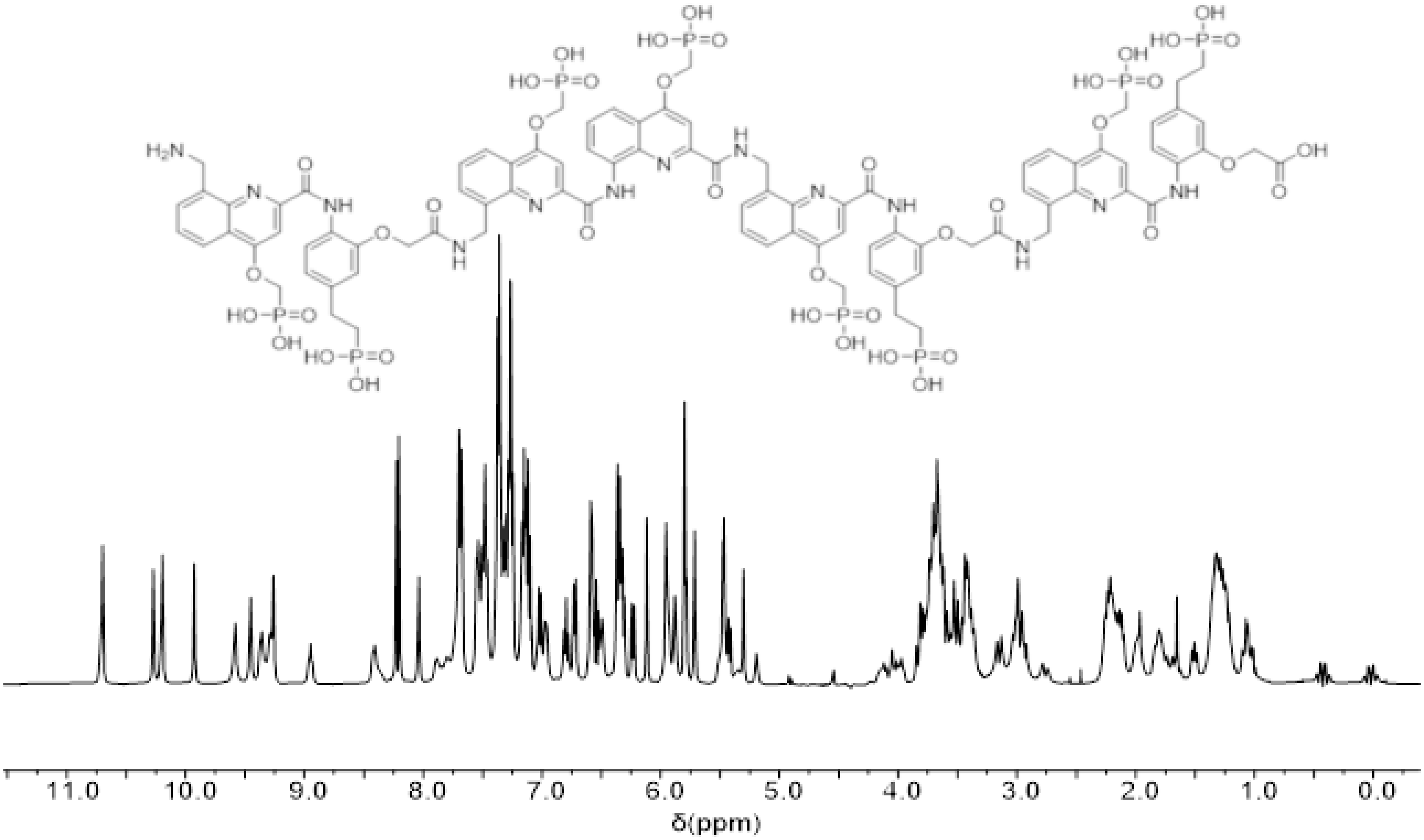

Figure S42. ^{1}H NMR spectra of **oligomer 13**. (500 MHz, 50 mM NH_4HCO_3, pH 8.5, H_2O/D_2O 9:1 v/v, at 25°C, water suppression).

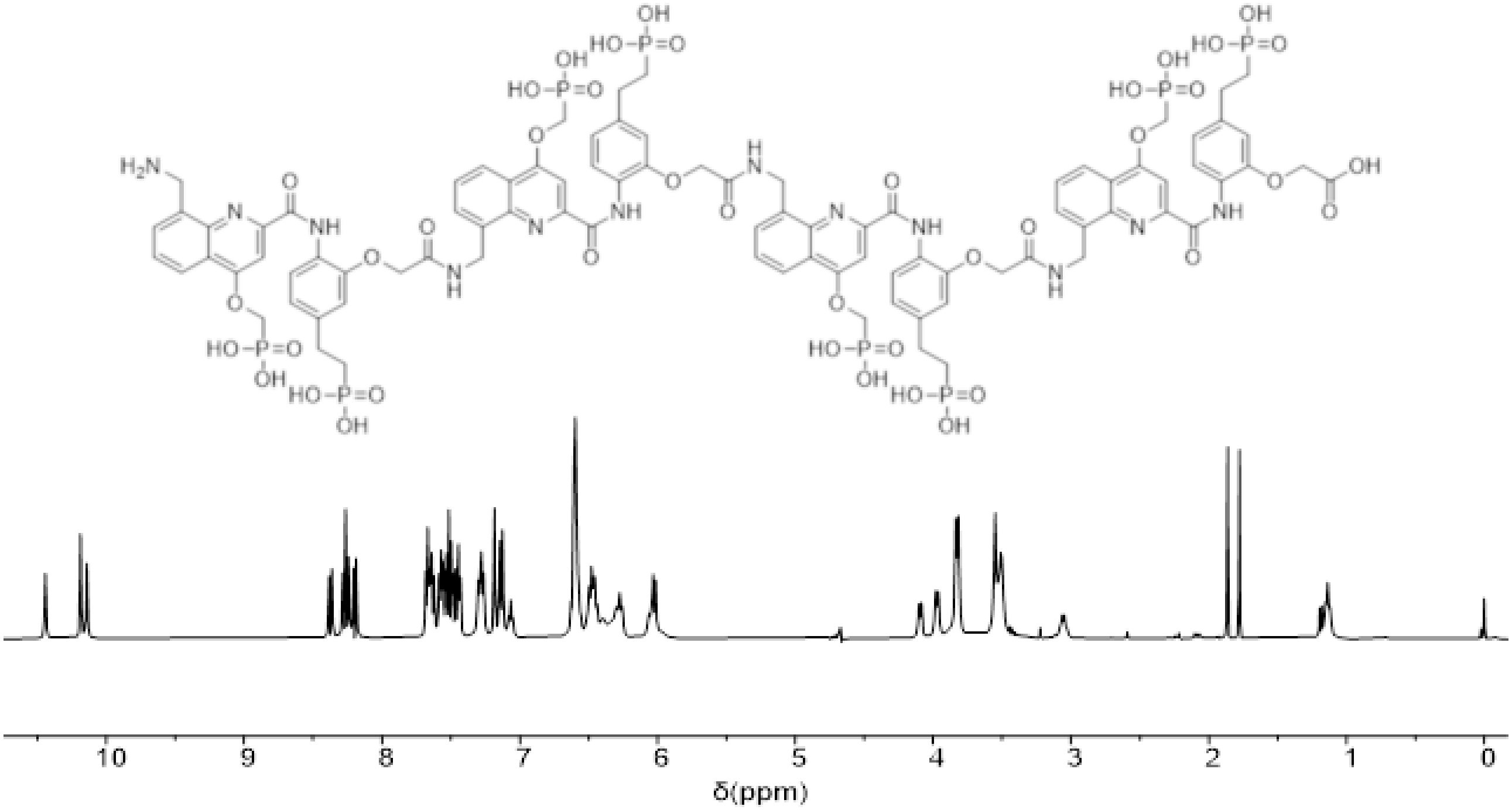

Figure S43. ^{1}H NMR spectrum of **oligomer 14**. (500 MHz, 50 mM NH_4HCO_3, pH 8.5, H_2O/D_2O 9:1 v/v, at 25°C, water suppression).

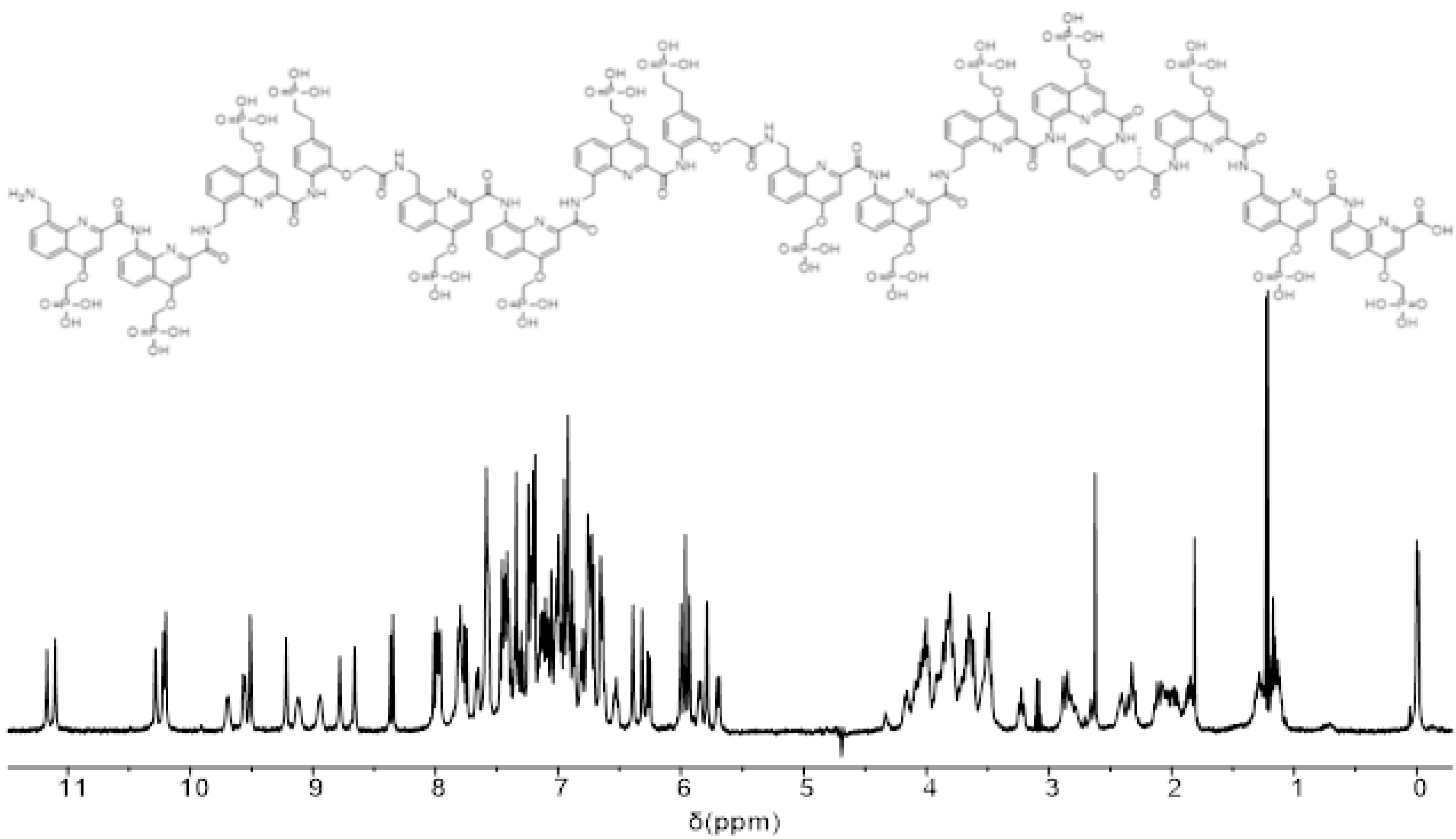

Figure S44. ^{1}H NMR spectrum of oligomer **16**. (500 MHz, 50 mM NH_4HCO_3, pH 8.5, H_2O/D_2O 9:1 v/v, at 25°C, water suppression).

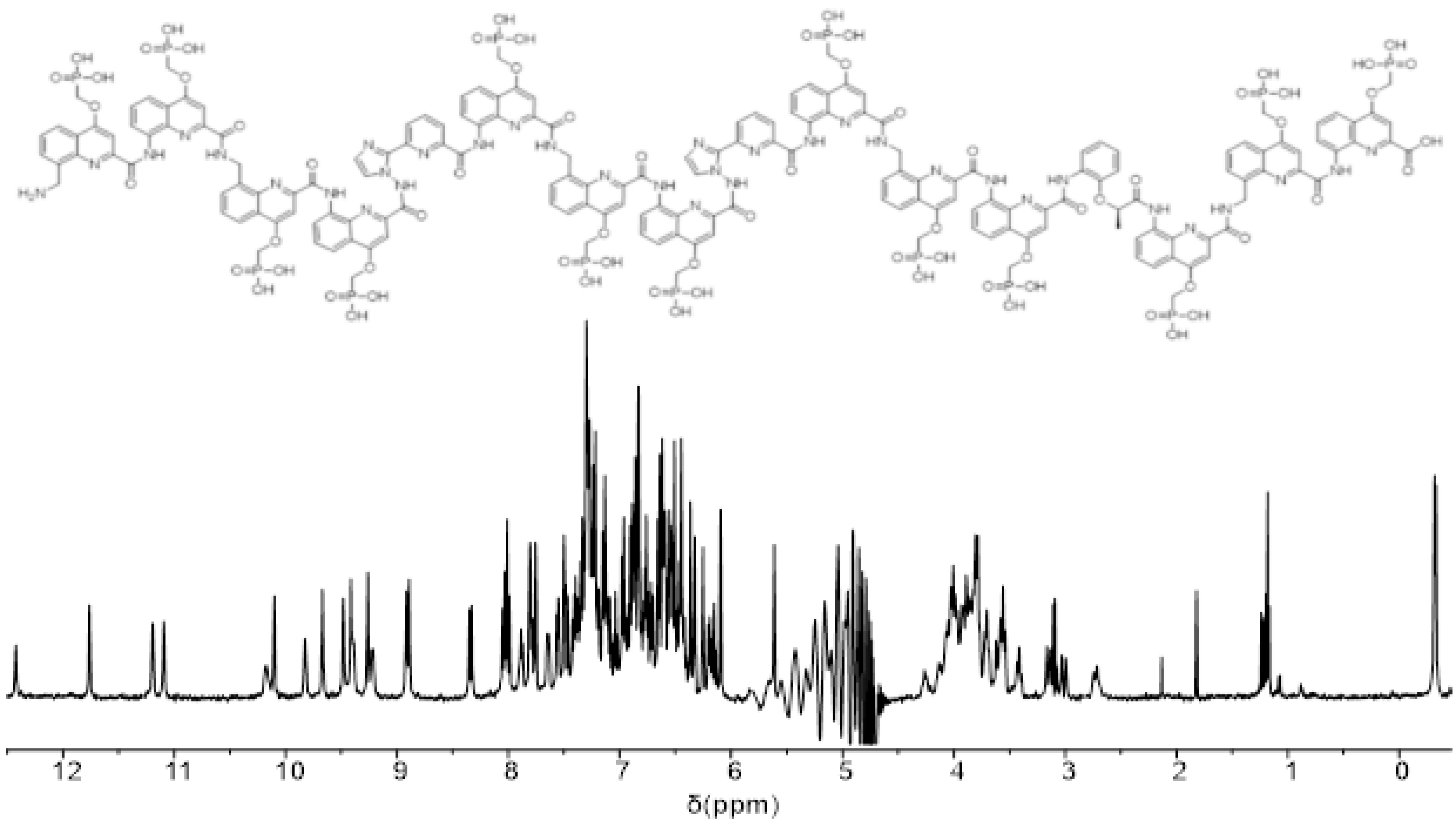

Figure S45. ^{1}H NMR spectrum of oligomer **18**. (500 MHz, 50 mM NH_4HCO_3, pH 8.5, H_2O/D_2O 9:1 v/v, at 25°C, water suppression).

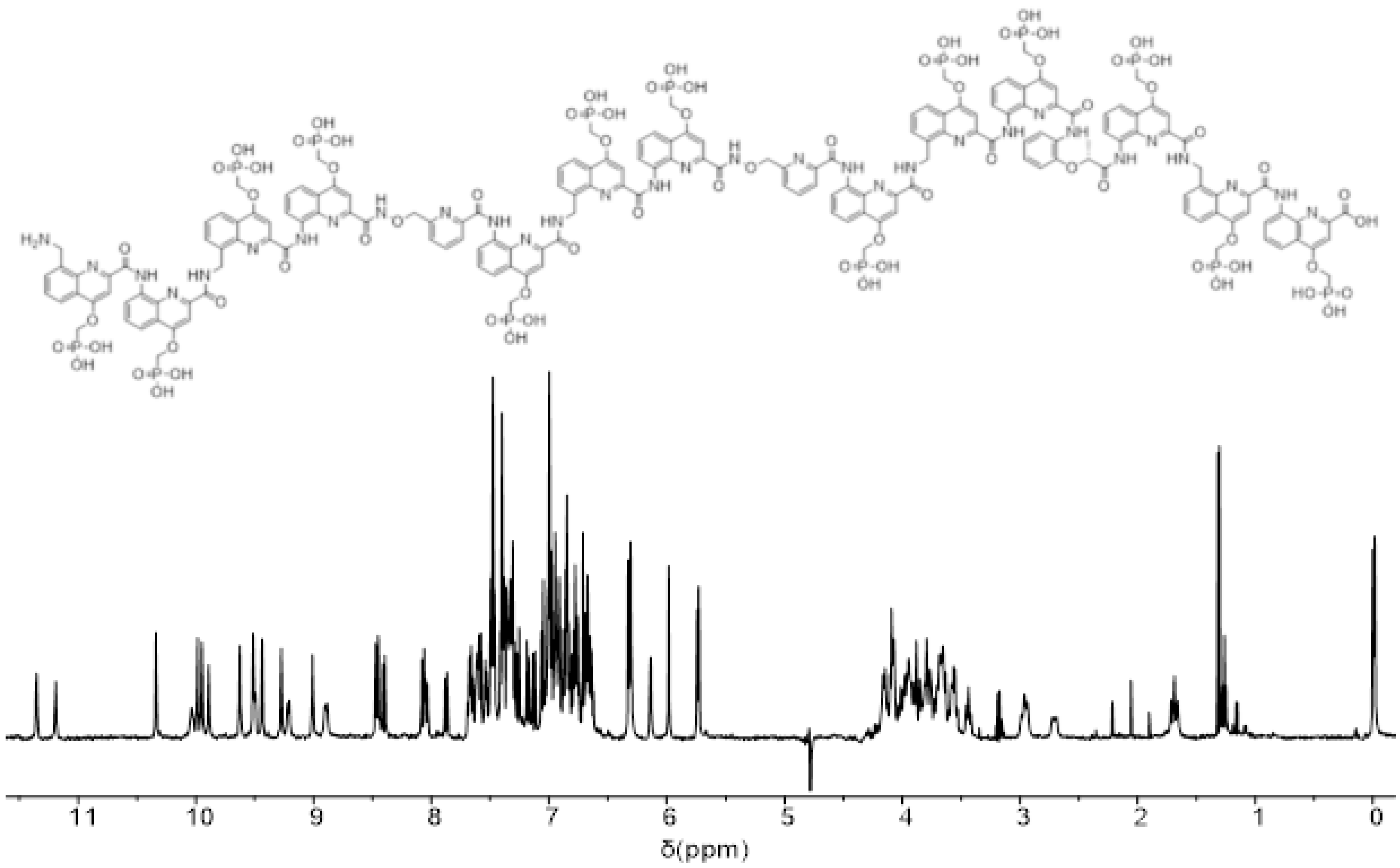

Figure S46. ^{1}H NMR spectrum of oligomer **19**. (500 MHz, 50 mM NH_4HCO_3, pH 8.5, H_2O/D_2O 9:1 v/v, at 25°C, water suppression).

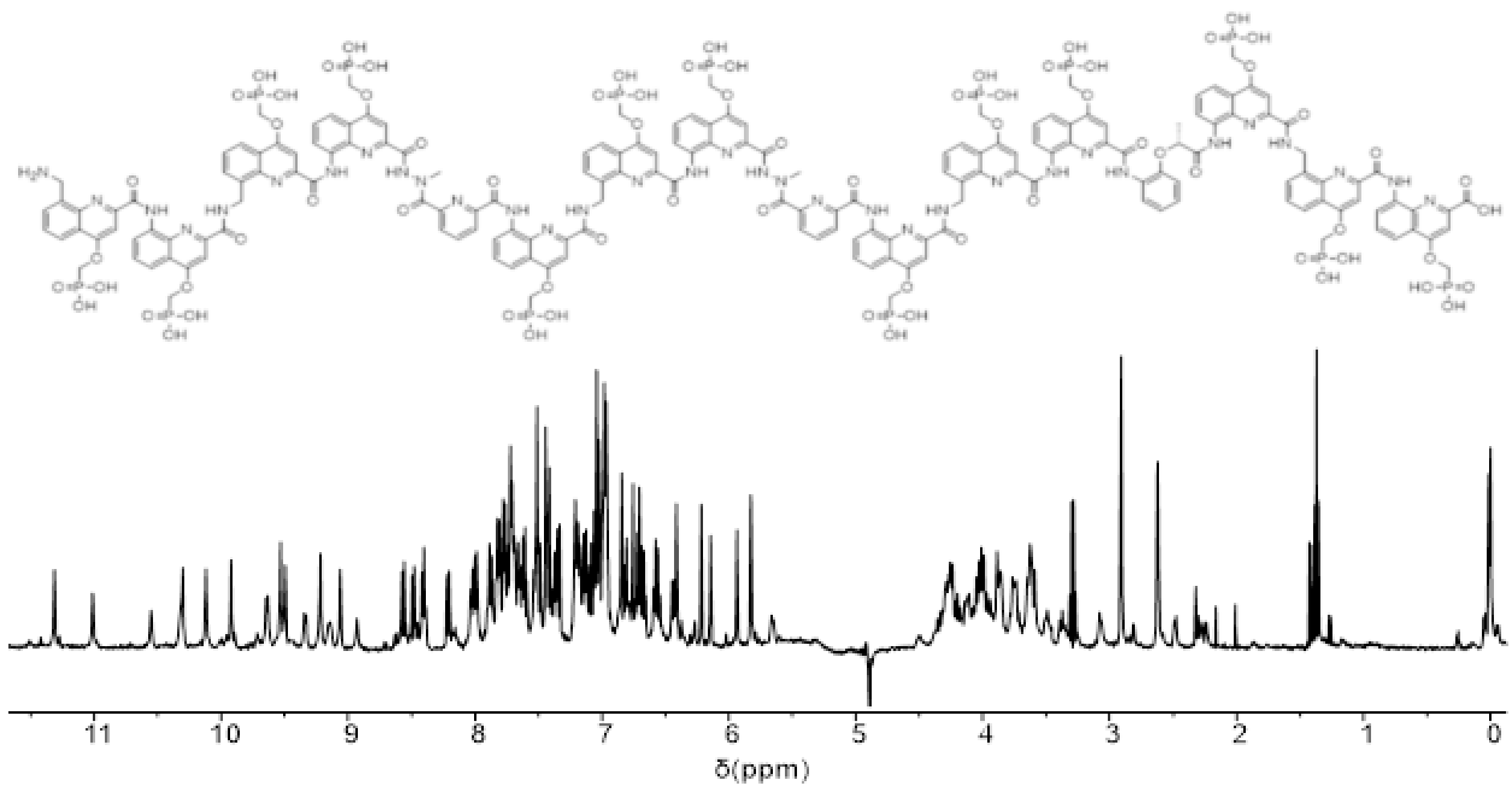

Figure S47. ^{1}H NMR spectrum of oligomer **20**. (500 MHz, 50 mM NH_4HCO_3, pH 8.5, H_2O/D_2O 9:1 v/v, at 25°C, water suppression).

5. HPLC chromatograms

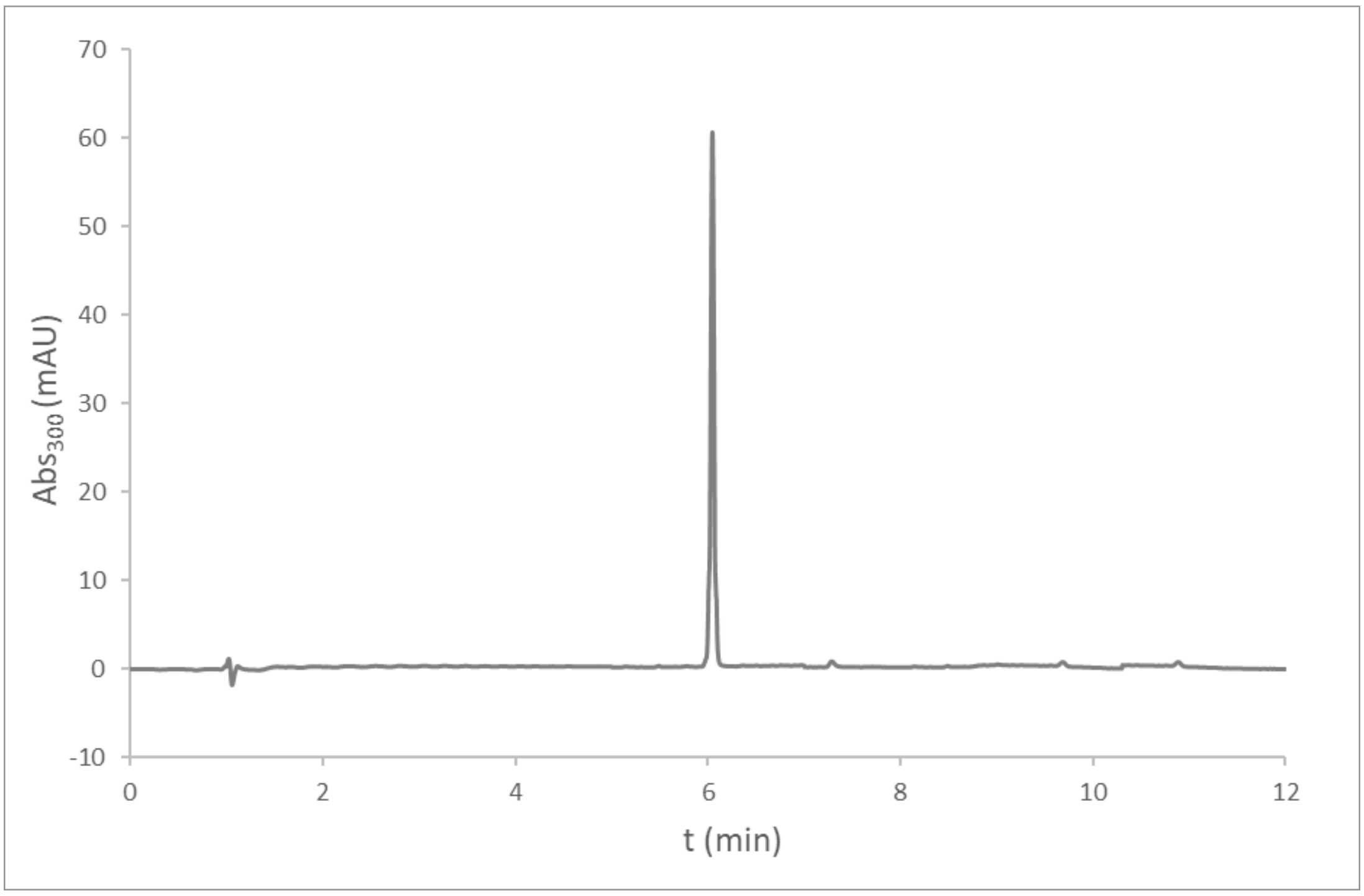

Figure S48. RP-HPLC chromatogram of oligomer **15a** using a linear gradient from 15% B to 100% B in 10 min; A: 12.5 mM TEAA in water, pH 8.5; B: 12.5 mM TEAA in water:acetonitrile (1:2), pH=8.5.

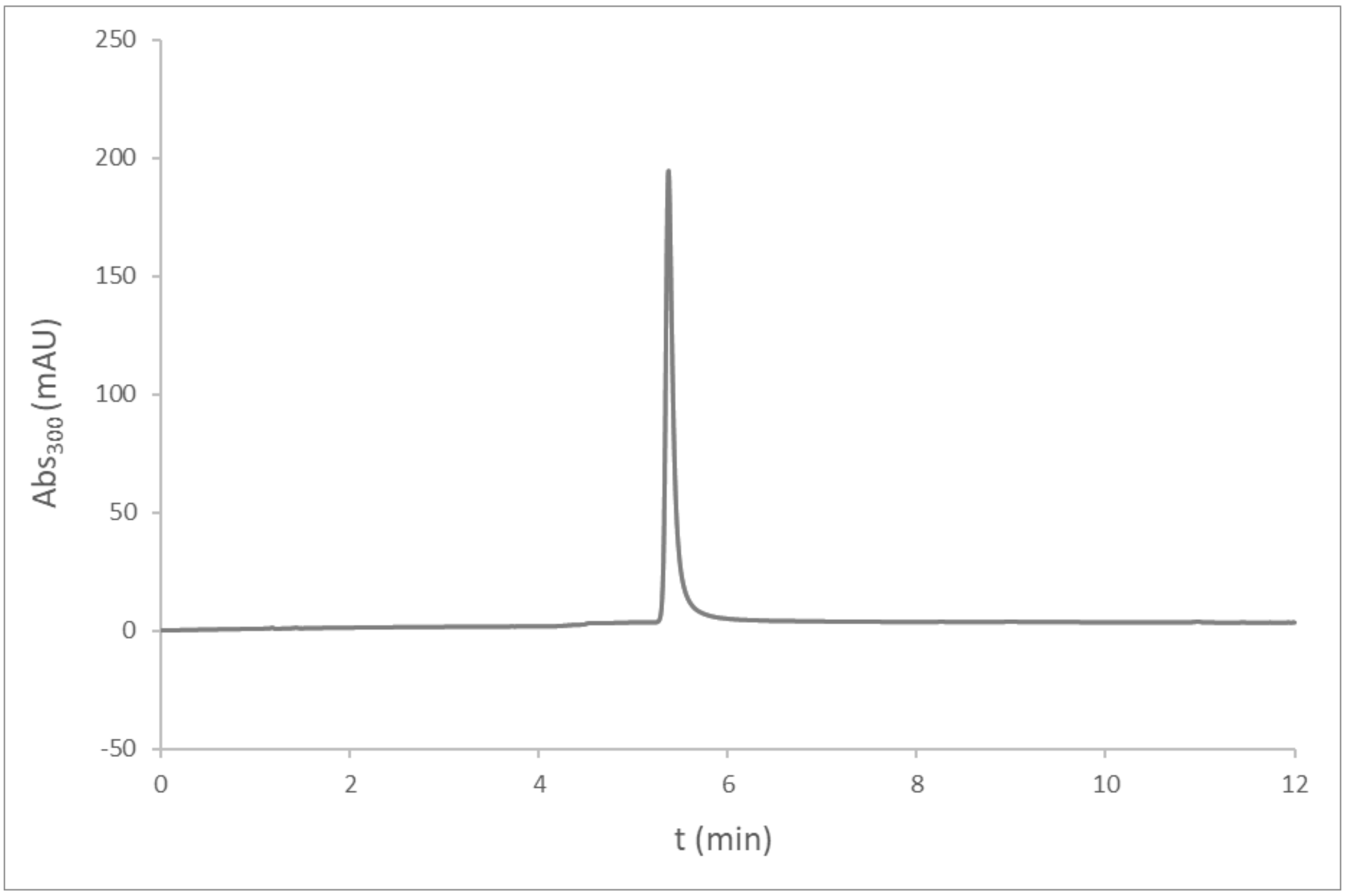

Figure S49. RP-HPLC chromatogram of oligomer **16a** using a linear gradient from 15% B to 100% B in 10 min; A: 12.5 mM TEAA in water, pH 8.5; B: 12.5 mM TEAA in water:acetonitrile (1:2), pH=8.5.

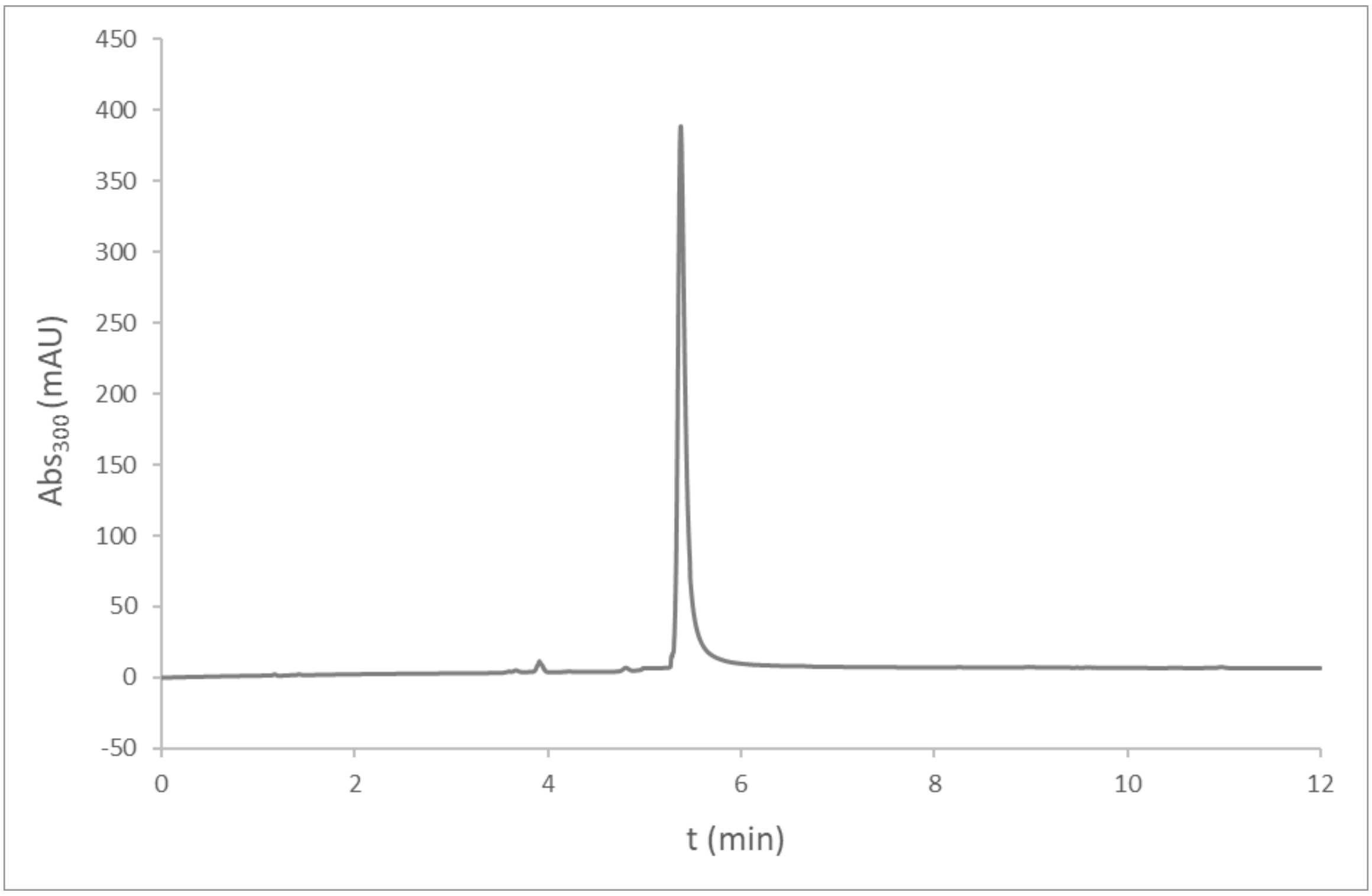

Figure S50. RP-HPLC chromatogram of oligomer **17a** using a linear gradient from 15% B to 100% B in 10 min; A: 12.5 mM TEAA in water, pH 8.5; B: 12.5 mM TEAA in water:acetonitrile (1:2), pH=8.5.

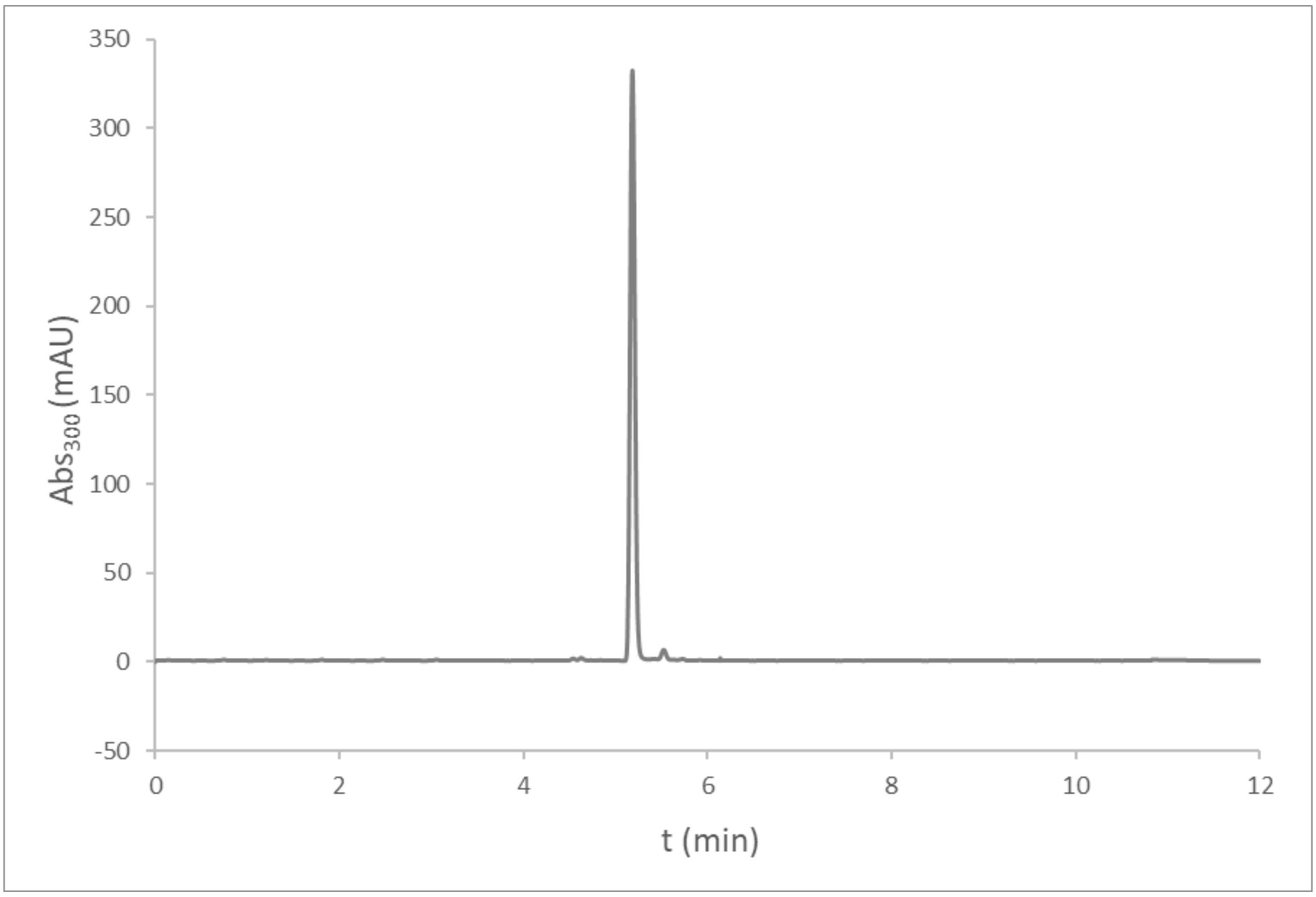

Figure S51. RP-HPLC chromatogram of oligomer **18a** using a linear gradient from 15% B to 100% B in 10 min; A: 12.5 mM TEAA in water, pH 8.5; B: 12.5 mM TEAA in water:acetonitrile (1:2), pH=8.5.

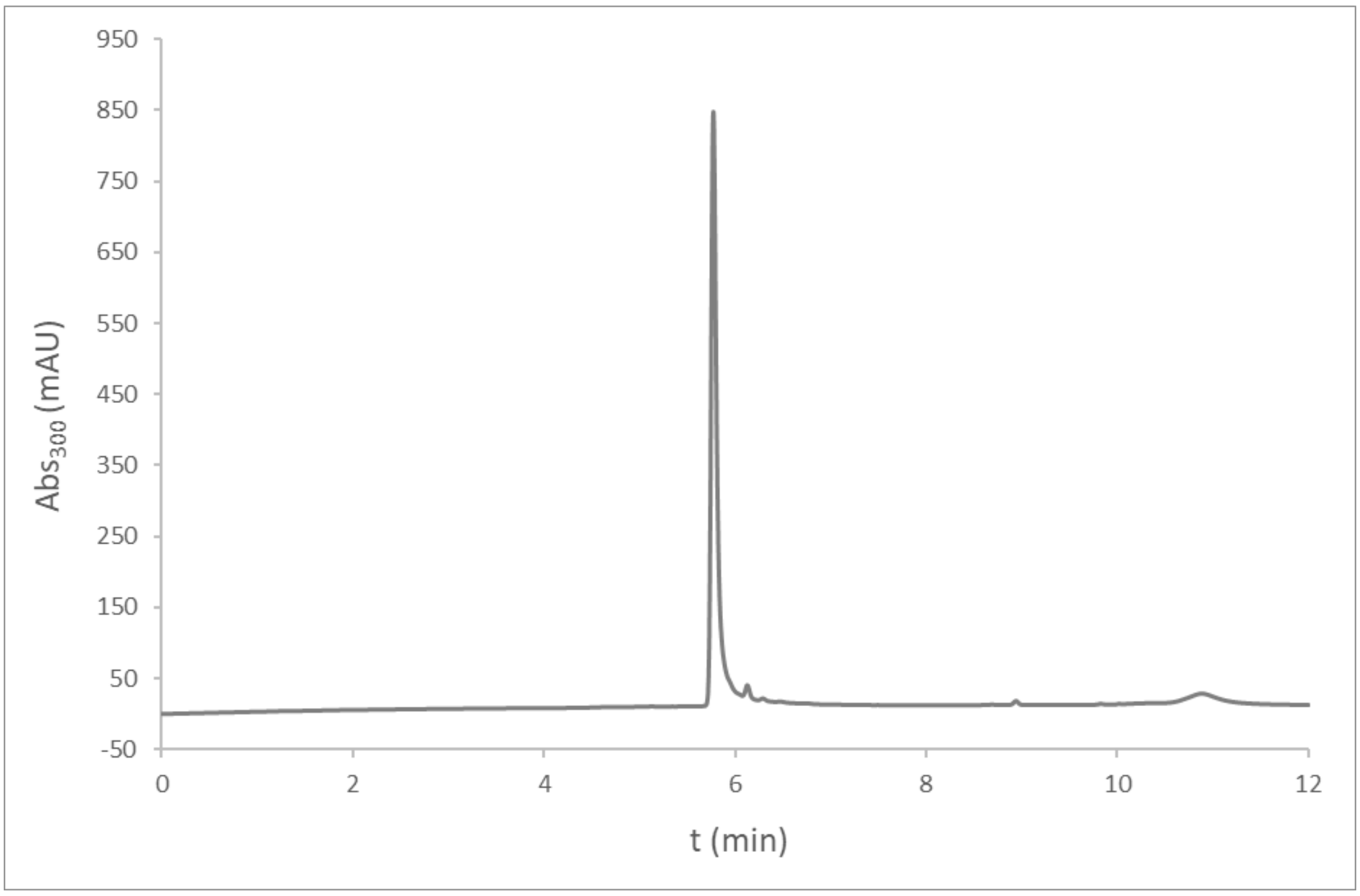

Figure S52. RP-HPLC chromatogram of oligomer **19a** using a linear gradient from 15% B to 100% B in 10 min; A: 12.5 mM TEAA in water, pH 8.5; B: 12.5 mM TEAA in water:acetonitrile (1:2), pH=8.5.

6. Mass spectra

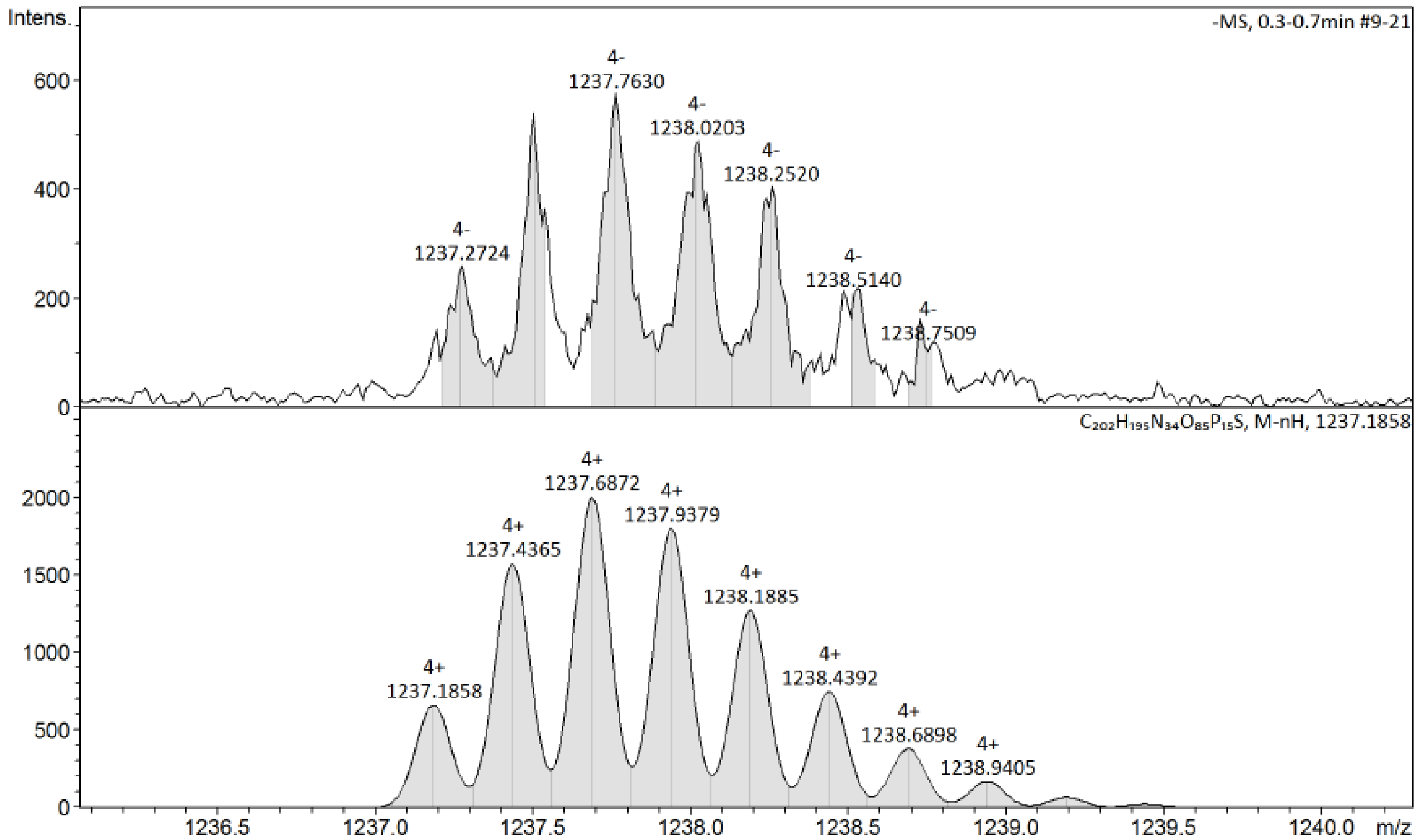

Figure S53. Mass spectrum of oligomer **15a**.

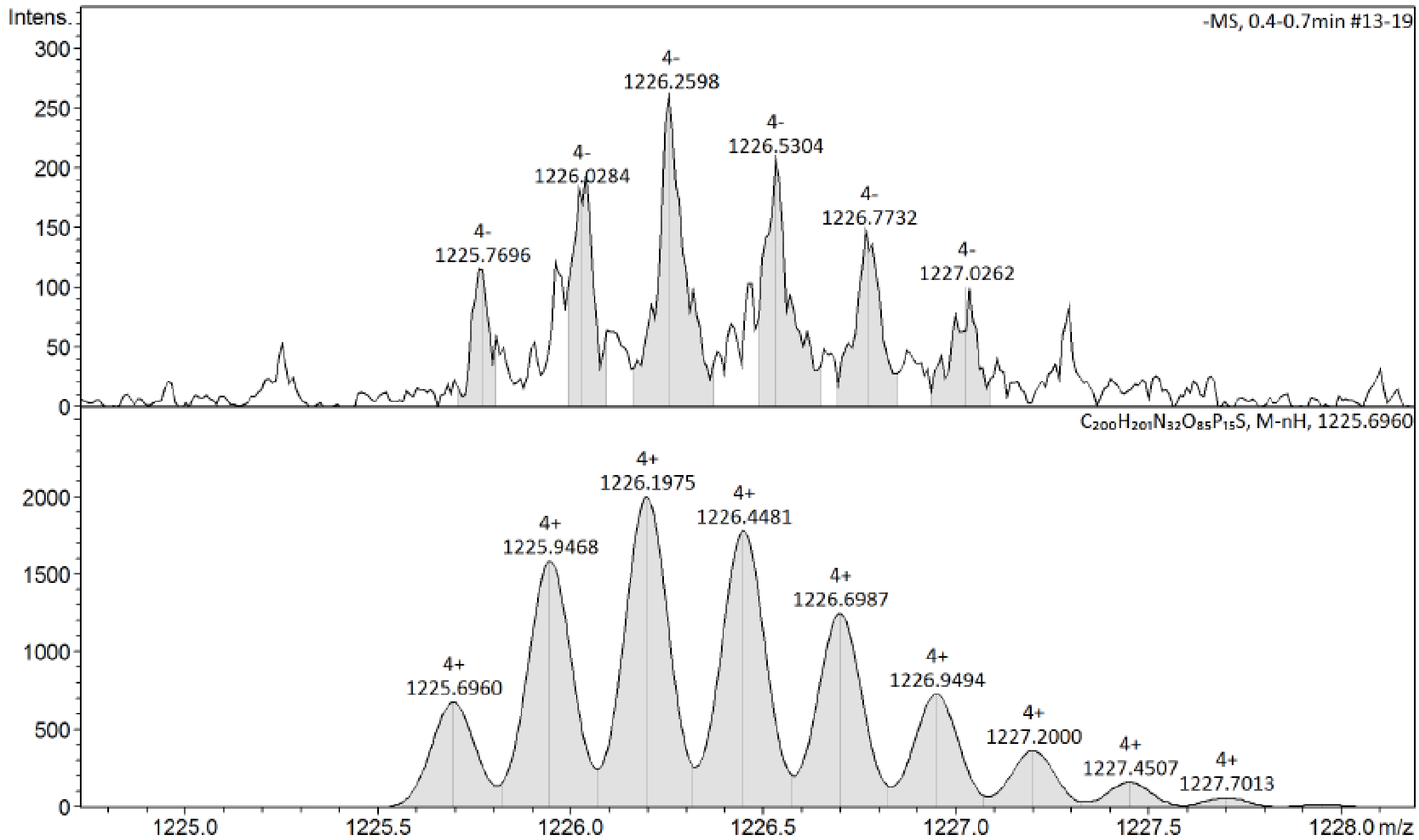

Figure S54. Mass spectrum of oligomer **16a**.

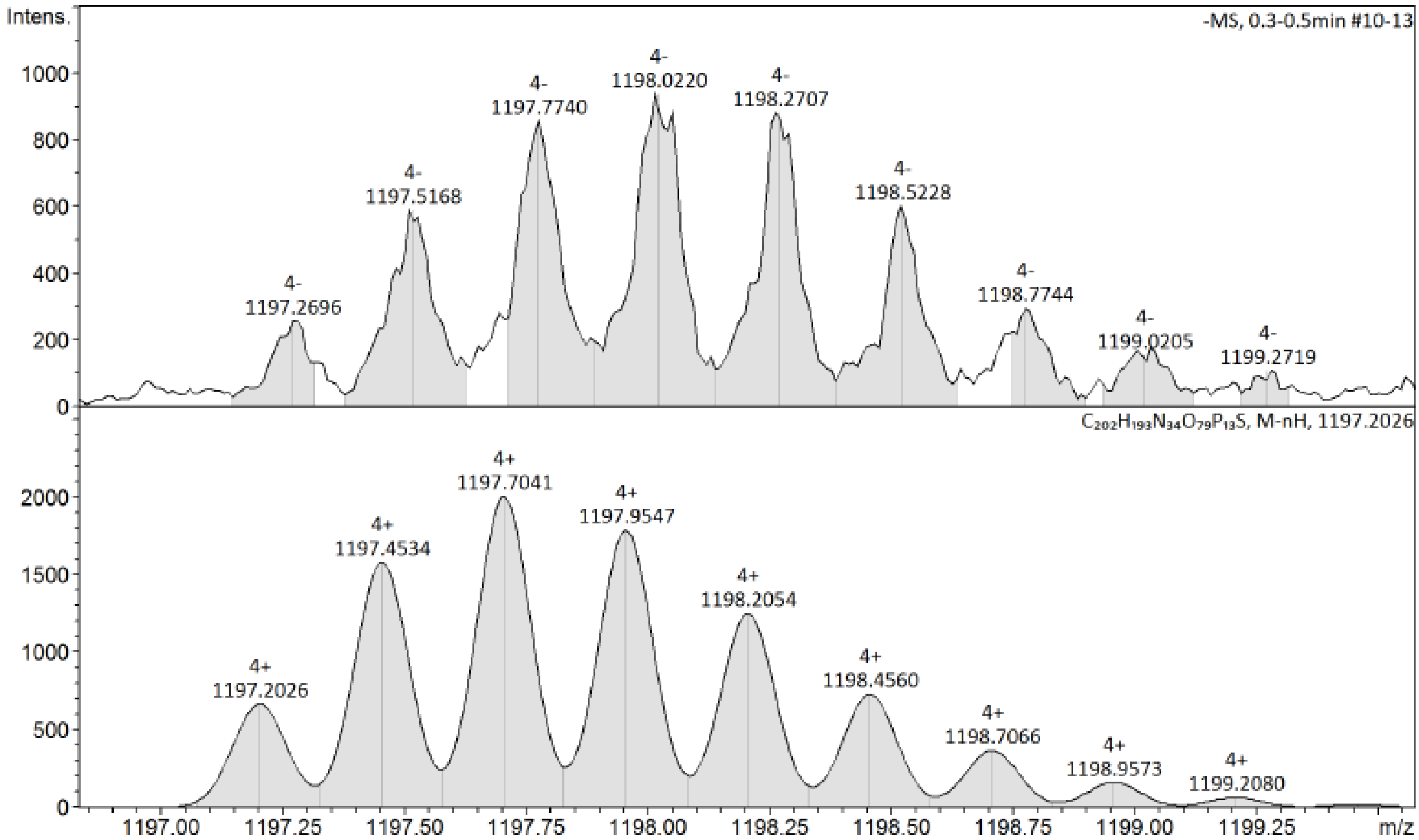

Figure S55. Mass spectrum of oligomer **17a**.

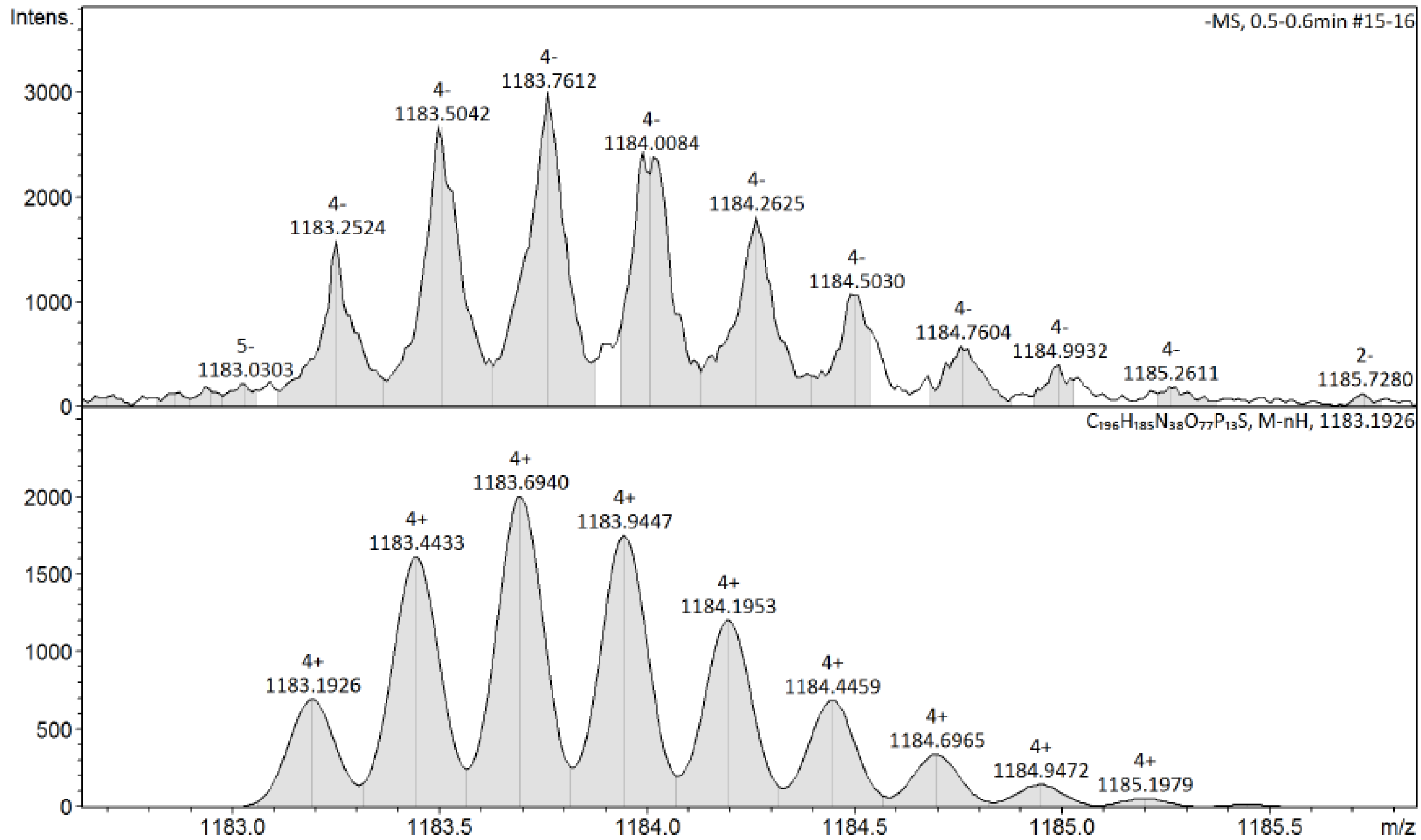

Figure S56. Mass spectrum of oligomer **18a**.

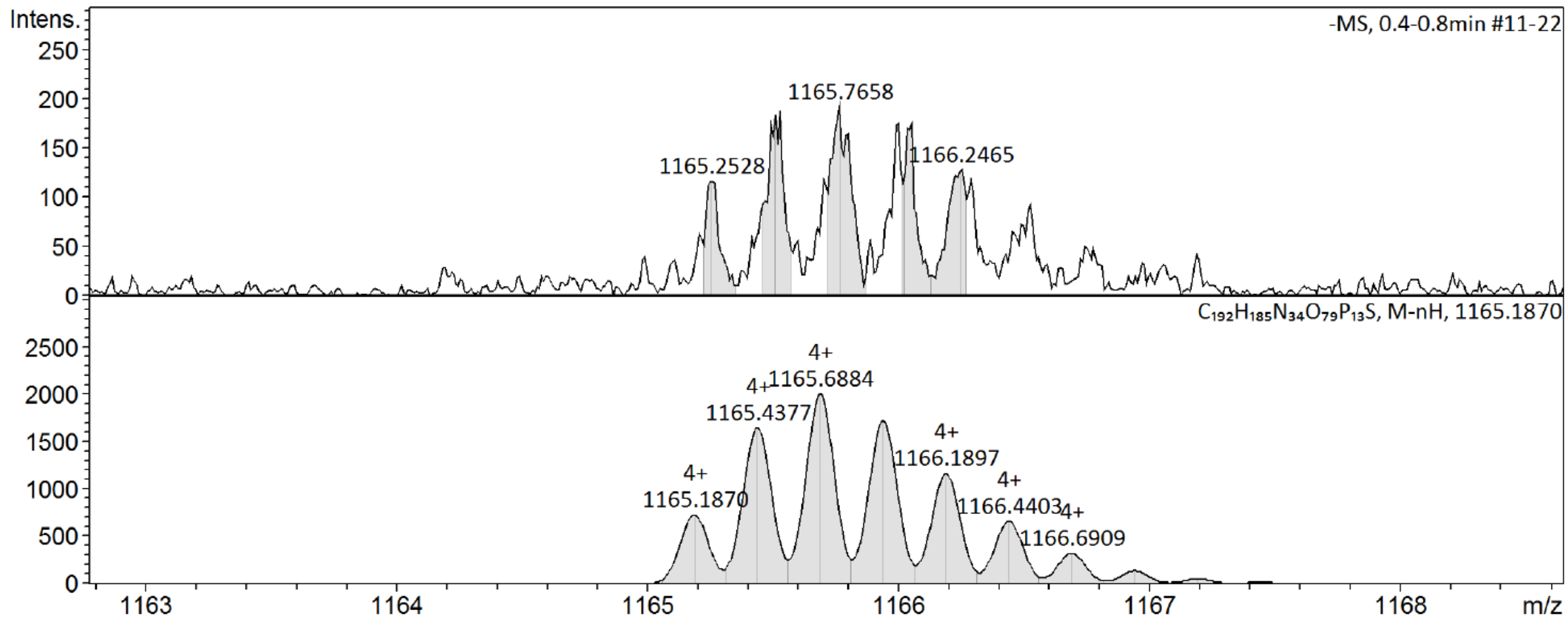

Figure S57. Mass spectrum of oligomer **19a**.

7. X-ray Crystallography

Aqueous solution of oligomer **5** was prepared by dissolving the lyophilized powder in water and 12 mM ammonium acetate to a final concentration of 5mM. Aqueous solution of oligomer **6** was prepared in a similar way. Crystallization trials were carried with commercial sparse matrix screens using standard sitting drop vapor diffusion method at 293 K. X-ray quality crystals of oligomer **5** (Figure S58) were obtained after three months by the addition of 0.8 µl of oligomer **5** and 0.8 µl of 2.9 M 1,6-hexanediol, 0.050 M Tris hydrochloride pH 8.5 and 0.005 M magnesium sulfate in the reservoir. X-ray quality crystals of oligomer **6** (Figure S58) were obtained within 5 days by the addition of 0.8 µl of oligomer **6** and 0.8 µl of 30% w/v PEG 8000, 0.050 M Tris hydrochloride pH 8.5 and 0.2 M magnesium chloride in the reservoir. For low temperature diffraction measurement single crystals were fished using micro loop and plunged into liquid nitrogen. The mother liquor served as cryo-protectants for the crystals.

X-ray diffraction data for oligomer **5** was collected at the ID30B[13] beamline in European Synchrotron Radiation Facility (ESRF), Grenoble. Diffraction data was measured at T = 100 K, λ = 0.9184 Å. The crystal was exposed for 0.02 s and 0.2° oscillation per frame and a rotation pass of 360° was measured using an EIGER2 X 9M detector. Diffraction data was processed using the program XDS[14]. The crystal belonged to the space group Pbnb with four independent helices per asymmetric unit (Z = 32, Z' = 4).

X-ray diffraction data for oligomer **6** was collected at the beamline P13 operated by EMBL Hamburg, at the Petra III storage ring (DESY, Hamburg)[15]. Diffraction data was measured at T = 100 K, λ = 0.8731 Å. The crystal was exposed for 0.008 s and 0.15° oscillation per frame and a rotation pass of 360° was measured using an EIGER 16M detector. Diffraction data was processed using the autoPROC pipeline.[14, 16-18] The crystal belonged to the space group P1 with four independent helices per asymmetric unit (Z = Z' = 4).

Both structures were solved with SHELXD[19] structure solution program using dual space method and refined by full-matrix least-squares method on F2 with SHELXL-2014[20] within Olex2[21]. After each refinement step, visual inspection of the model and the electron-density maps were carried out using Olex2[21] and Coot[22] using 2Fo − Fc and difference Fourier (Fo − Fc) maps.

The initial structure solution of oligomer **5** revealed all main-chain atoms of four helices. Few Q^D side chains and N-terminus tail-atoms were severely disordered and omitted. The N and O atoms at

C-terminus have been placed randomly. All non-H atoms were refined with anisotropic displacement parameters. After several attempts to model the disordered side chains and diethylene glycol tail, the SQUEEZE[23] procedure was used to flatten the electron density map. Very disordered solvent molecules were removed. Calculated total potential solvent accessible void volume and electron count per cell are 32550.9 $Å^3$ and 10144 respectively. Hydrogen atoms for oligomer **5** were placed at idealized positions.

The initial structure solution of oligomer **6** revealed majority of the main chain atoms and few side chains of three helices out of four. After several iterations of least square refinement, the main chain traces were established for three helices out of four. For one helix, only seven monomers out of nine could be traced. Throughout refinement, the terminal PEG tails and some side chain atoms of Q^D and Q^A were not modelled due to the poor quality of data. After several attempts to model the disordered side chains and diethylene glycol tail, the SQUEEZE[18] procedure was used to flatten the electron density map. Calculated total potential solvent accessible void volume and electron count per cell are 10061.8 $Å^3$ and 3048 respectively. Hydrogen atoms for oligomer **6** were placed at idealized positions.

Statistics of data collection and refinement of oligomer **5** and oligomer **6** are described in Table S1. The final cif file of oligomer **5** and oligomer **6** were examined in IUCr's checkCIF algorithm. Due to the large volume fractions of disordered solvent molecules, weak diffraction intensity and poor resolution, a number of A- and B- level alerts remain in the checkCIF file. These alerts are inherent to the data set and refinement procedures. They are listed below and were divided into two groups. The first group demonstrates weak quality of the data and refinement statistics when compared to those expected for small molecule structures from highly diffracting crystals. The second group is concerned to decisions made during refinement and explained below. Atomic coordinates and structure factors of oligomer **5** and oligomer **6** were deposited in the Cambridge Crystallographic Data Centre (CCDC) with accession codes 2286782 and 2478322 respectively. The data is available free of charge upon request (www.ccdc.cam.ac.uk/).

CheckCIF validation of oligomer 5:

Group 1 alerts (these illustrate weak quality of data and refinement statistics if compared to small molecule structures from highly diffracting crystals):

THETM01_ALERT_3_A   The value of sine(theta_max)/wavelength is less than 0.550

Calculated sin(theta_max)/wavelength = 0.4836

PLAT082_ALERT_2_A High R1 Value 0.24

PLAT084_ALERT_3_B High wR2 Value (i.e. > 0.25) 0.61

PLAT410_ALERT_2_A Short Intra H...H Contact

PLAT411_ALERT_2_A Short Intra H...H Contact

PLAT602_ALERT_2_A Solvent Accessible VOID(S) in Structure

PLAT241_ALERT_2_B High 'MainMol' Ueq as Compared to Neighbors

PLAT242_ALERT_2_B Low 'MainMol' Ueq as Compared to Neighbors

PLAT340_ALERT_3_B Low Bond Precision on C-C Bonds .... 0.01624 Ang.

PLAT430_ALERT_2_A Short Inter D...A Contact

PLAT964_ALERT_2_B SHELXL WEIGHT Par. Values in CIF & RES Differ

Group 2 alert (is connected with decision made during refinement and explained below):

PLAT306_ALERT_2_B Isolated Oxygen Atom (H-atoms Missing ?) Check

Dummy O atom was introduced into refinement.

CheckCIF validation of oligomer 6:

Group 1 alerts (these illustrate weak quality of data and refinement statistics if compared to small molecule structures from highly diffracting crystals):

THETM01_ALERT_3_A The value of sine(theta_max)/wavelength is less than 0.550

Calculated sin(theta_max)/wavelength = 0.4348

PLAT029_ALERT_3_A _diffrn_measured_fraction_theta_full value Low . 0.923

PLAT082_ALERT_2_A High R1 Value 0.25

PLAT084_ALERT_3_B High wR2 Value (i.e. > 0.25) 0.57

PLAT412_ALERT_2_A Short Intra H...H Contact

PLAT414_ALERT_2_A Short Intra H...H Contact

PLAT602_ALERT_2_A Solvent Accessible VOID(S) in Structure

PLAT242_ALERT_2_B Low 'MainMol' Ueq as Compared to Neighbors

PLAT340_ALERT_3_B Low Bond Precision on C-C Bonds .... 0.02305 Ang.

PLAT430_ALERT_2_A Short Inter D...A Contact

DIFMN02_ALERT_2_B The minimum difference density is < -0.1*ZMAX*1.00

_refine_diff_density_min given = -1.025 Test value = -0.800

PLAT097_ALERT_2_B Large Reported Max. (Positive) Residual Density 1.37 eA-3

PLAT098_ALERT_2_B Large Reported Min. (Negative) Residual Density -1.02 eA-3

PLAT315_ALERT_2_B Singly Bonded Carbon Detected

PLAT911_ALERT_3_B Missing FCF Refl Between Thmin & STh/L= 0.435

Table S1. Crystallographic data and refinement details for oligomer **5** and oligomer **6**.

Identification code	oligomer **5**	oligomer **6**.
Empirical formula	$C_{865} H_{69.8} Mg_{0.4} N_{17} O_{26.9}$	$C_{91.5} H_{59.3} N_{18.3} O_{18.7}$
Formula weight	1786.46	1714.32
Temperature	100 K	100.15 K
Wavelength	0.8731 Å	0.8731 Å
Crystal system	Orthorhombic	Triclinic
Space group	*P*bnb	*P*1
Unit cell dimensions	*a* = 34.10 (6) Å<br>*b* = 44.70 (5) Å<br>*c* = 58.87 (9) Å	*a* = 22.09 (2) Å<br>*b* = 25.28 (2) Å<br>*c* = 33.72 (3) Å
Volume	89734 (232) Å^3	16857 (26)
Z	32	4
Density (calculated)	1.058 g/cm^3	0.675 g/cm^3
Absorption coefficient	0.135 µ/mm^{-1}	0.080 µ/mm^{-1}
Colour and shape	Yellow, block	Yellow, needle
Crystal size	0.15 x 0.05 x 0.02 mm	0.20 x 0.03 x 0.02 mm
Index ranges	-32 ≤ h ≤ 32<br>-42 ≤ k ≤ 43<br>-56 ≤ l ≤ 56	-19 ≤ h ≤ 19<br>-21 ≤ k ≤ 21<br>-28 ≤ l ≤ 28
Reflections collected	40953	87785
R_{int}	0.0580	0.0323
Data/restraints/parameters	40953/2622/172	42218/520/908
Goodness-of-fit on F^2	2.352	2.838
Final R indexes [$I > 2\sigma(I)$]	R_1 = 0.2980<br>wR_2 = 0.5669	R_1 = 0.2491<br>wR_2 = 0.5207
Final R indexes [all data]	R_1 = 0.2436<br>wR_2 = 0.6144	R_1 = 0.2729<br>wR_2 = 0.5753
Largest diff. peak and hole	1.0/-0.6 e Å^{-3}	1.37/-1.02 e Å^{-3}
CCDC #	2286782	2478322

(a) (b)

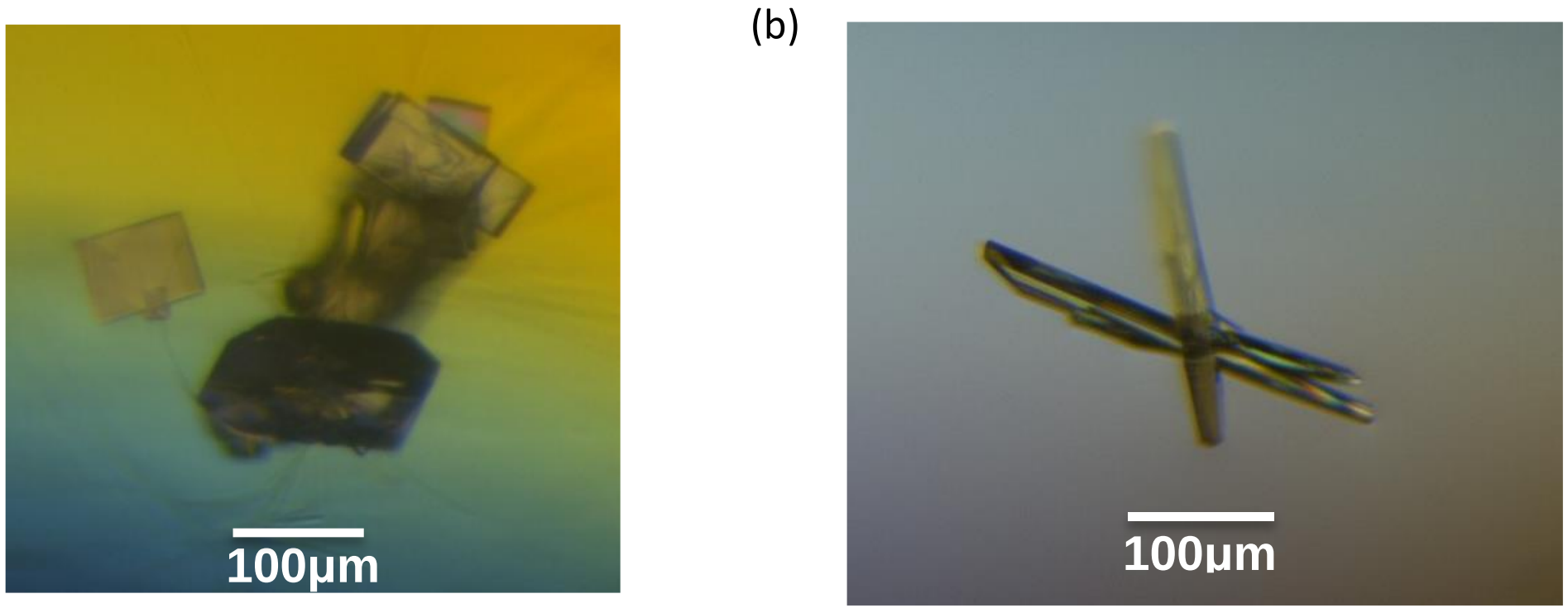

Figure S58. Crystals of oligomer **5** (a) and oligomer **6** (b) observed under crossed polarizing microscope.

The X-ray intensity data of compound **1** and compound **1d** were measured on a Bruker D8 Venture TXS system equipped with a multilayer mirror monochromator and a Mo Kα rotating anode X-ray tube (λ = 0.71073 Å). The frames were integrated with the Bruker SAINT software package.[24] Data were corrected for absorption effects using the Multi-Scan method (SADABS).[25] The structure was solved and refined using the Bruker SHELXTL Software Package [20]. All C-bound hydrogen atoms have been calculated in ideal geometry riding on their parent atoms, the N- and O-bound hydrogen atoms have been refined freely. The structure has been refined as a 2-component perfect inversion twin. The figures have been drawn at the 25% ellipsoid probability level.[26] Atomic coordinates and structure factors of compound **1** and compound **1d** were deposited in the Cambridge Crystallographic Data Centre (CCDC) with accession codes: 2514117, 2514118. The data is available free of charge upon request (www.ccdc.cam.ac.uk/).

Table S2. Crystallographic data and refinement details for compound **1** and compound **1d**.

Identification code	Compound **1**	Compound **1d**
Empirical formula	$C_{23}H_{19}N_3O_5$	$C_{23}H_{23}N_5O_7$
Formula weight	417.41	481.46
crystal size/mm	0.140 × 0.100 × 0.080	0.090 × 0.060 × 0.020
T/K	173.(2)	173.(2)
radiation	MoKα	MoKα
diffractometer	'Bruker D8 Venture TXS'	'Bruker D8 Venture TXS'
crystal system	monoclinic	triclinic
space group	'P 1 c 1'	'P -1'
a/Å	8.905(7)	9.7590(7)
b/Å	10.403(9)	10.1656(6)
c/Å	10.793(9)	12.8980(9)

α/°	90	70.497(2)
β/°	95.66(3)	80.324(2)
γ/°	90	75.834(2)
V/Å^3	995.0(14)	1164.23(14)
Z	2	2
calc. density/g cm^{-3}	1.393	1.373
μ/mm^{-1}	0.100	0.104
absorption correction	Multi-Scan	Multi-Scan
transmission factor range	0.94–0.99	0.95–1.00
refls. measured	18936	12965
R_{int}	0.0485	0.0382
mean σ(I)/I	0.0441	0.0439
θ range	3.020–27.484	3.128–25.345
observed refls.	4118	3153
x, y (weighting scheme)	0.0340, 0.1700	0.0385, 0.5865
hydrogen refinement	mixed	mixed
Flack parameter	0.5	?
refls in refinement	4460	4242
parameters	289	348
restraints	2	0
$R(F_{obs})$	0.0348	0.0472
$R_w(F^2)$	0.0827	0.1107
S	1.042	1.022
shift/error$_{max}$	0.001	0.001
max electron density/e Å^{-3}	0.152	0.218
min electron density/e Å^{-3}	−0.175	−0.213
CCDC #	2514117	2514118

8. HU Protein expression and purification

Overexpression of HU. Full-length HU was cloned into pET21a (a gift from PA Rice) expressed in E. coli BL21 cells. Overnight pre-cultures in Luria broth (LB) supplemented with 100 µg/ml ampicillin were diluted 1000- fold with fresh 4L LB (Luria Broth) media and grown at 37 °C until OD600 reached 0.6. The expression was induced by addition of isopropyl 1-thio-β-D-galactopyranoside to reach a final concentration of 0.8 mM, and the culture was incubated for 3.5 hrs at 37 °C. Next, the cells were harvested at 4000 rpm (J-LITE® JLA9.1000 Rotor, Beckman Coulter) and stored at −20 °C. All further purification procedures were carried out at 4°C.

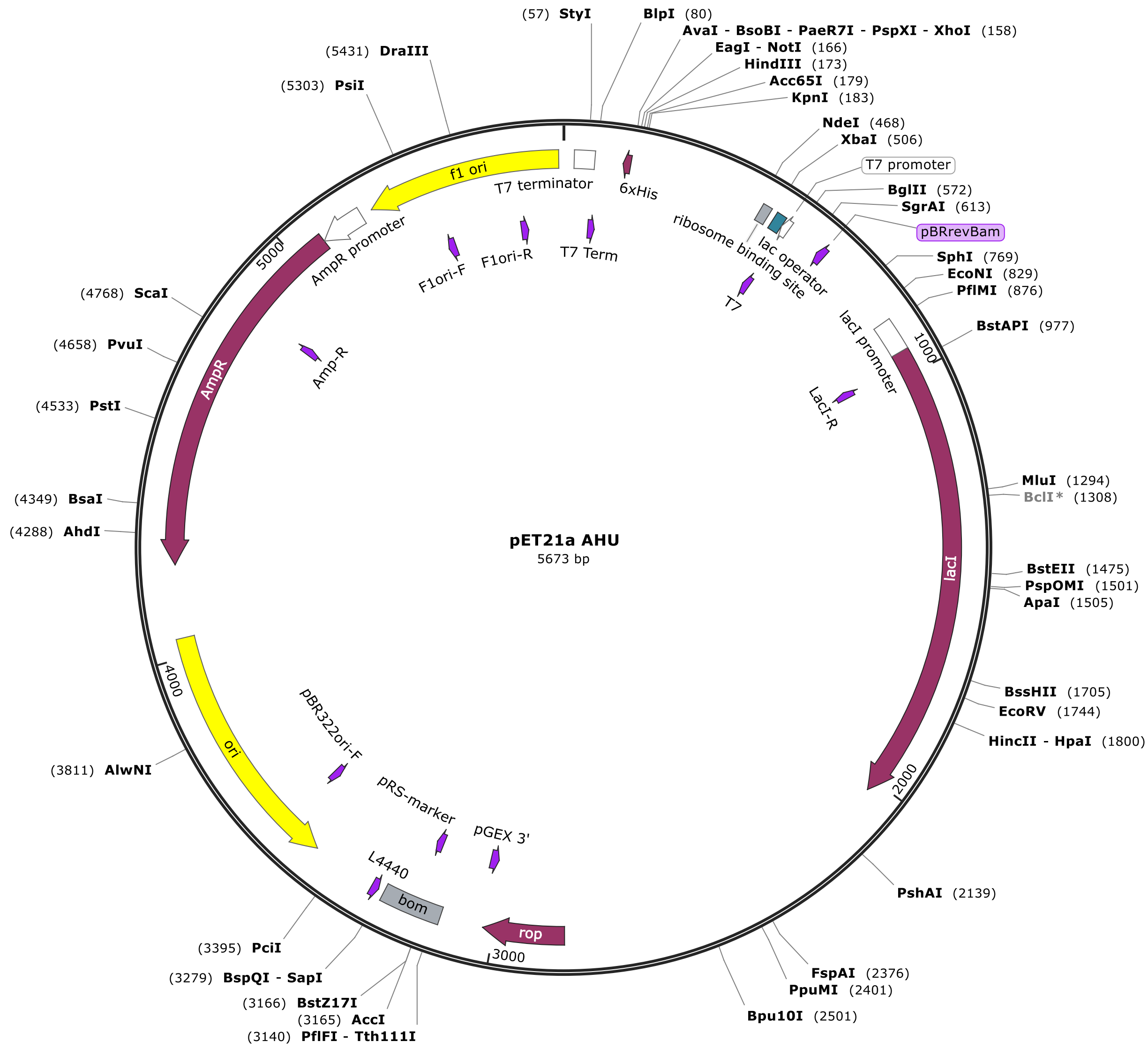

Figure S59. Expression vector of HU.

Purification of HU. The resulting cell pellet was resuspended in 45 ml buffer A (50 mM Tris (pH 8.0), 12.5 mM EDTA, 2.5 mM DTT) + 15 mL buffer B (200 mM Tris (pH 8.0), 1M NaCl) + 1 × protease inhibitor cocktail (HaltTMProtease inhibitor cocktail by thermos scientific). The cells were disrupted through 6 rounds of sonication (3 min/round + 3 min pause in between). The extract was clarified by centrifugation at 17000 rpm (JA-25.5 Rotor, Beckman Coulter) for 45 min. The supernatant was adjusted to 50% saturation in ammonium sulphate. After 60 min stirring, the solution was separated by centrifugation at 17000 rpm (JA-25.5 Rotor, Beckman Coulter) for 45 min. The supernatant was adjusted to 80% saturation in ammonium sulphate.[27] After 60 min stirring, the solution was separated once more by centrifugation at 17000 rpm (JA-25.5 Rotor, Beckman Coulter) for 45 min. A total of 15 mL buffer C (20 mM HEPES (pH 7.5), 150 mM NaCl, 1 mM DTT) was added to the pellet. The material was dialysed against water for 30 min and then against buffer C (20 mM HEPES (pH

7.5), 150 mM NaCl, 1 mM DTT) overnight. The dialysed fraction was applied to a heparin gravity flow column and eluted with a salt gradient at 2 M NaCl. Further purification of HU was performed via size-exclusion chromatography (SEC) HiLoad 26/600 Superdex 200 pg. The fractions were then concentrated and the purity of the protein was confirmed by SDS PAGE gel and LCMS analysis. The protein was then stored in a -80 °C freezer and thawed prior to following BLI measurements.

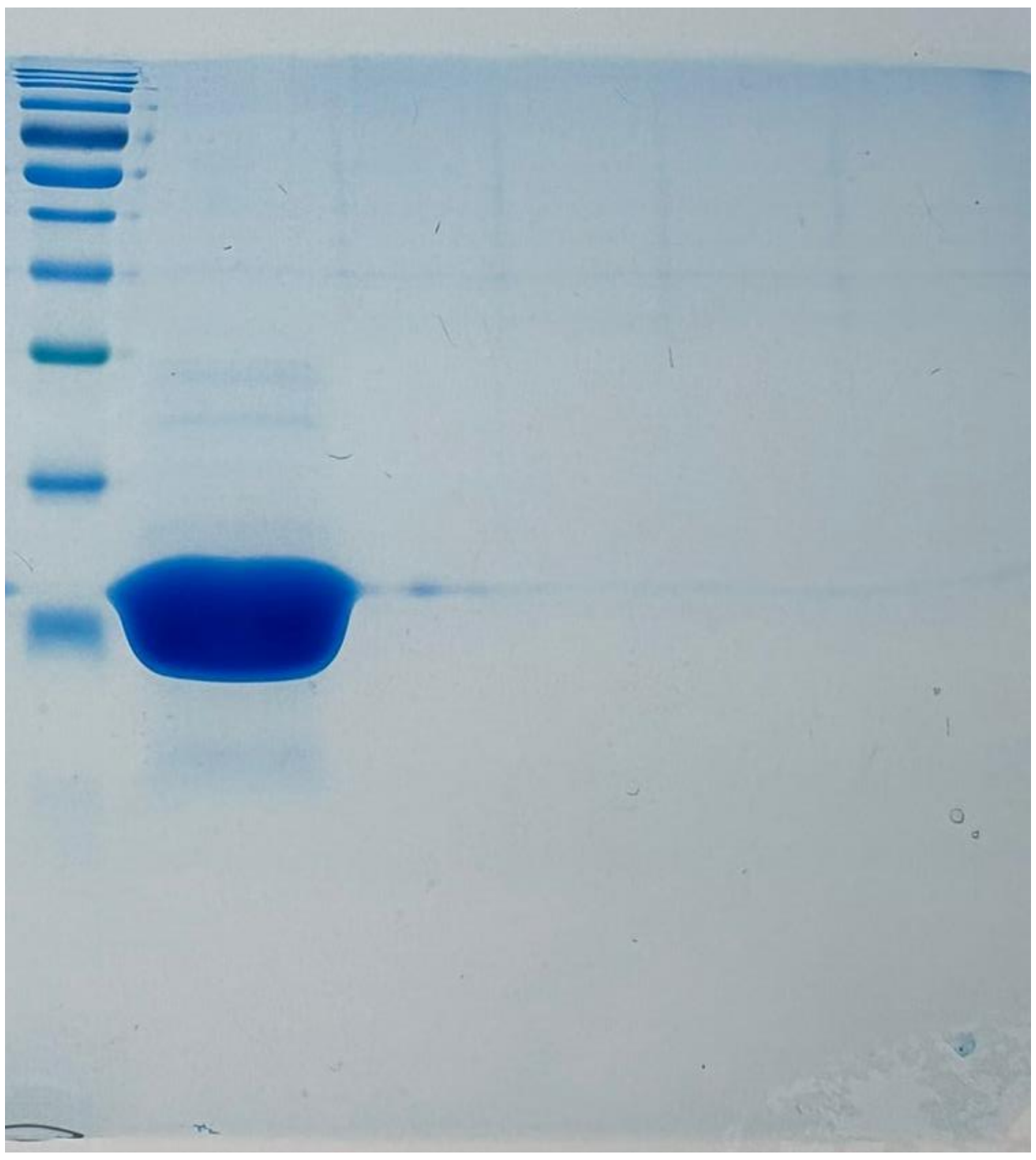

Figure S60. Polyacrylamide gel electrophoresis (PAGE) of purified HU.

9. Biolayer interferometry (BLI)

BLI experiments were performed on an Octet R8 instrument from Sartorius at 25°C. Prior to an assay, streptadvidin (SA) sensors were soaked for at least 10 min in phosphate buffer saline (1 × PBS). The kinetic experiment always starts with a baseline step over 60 s in 1 × PBS buffer, followed by the loading of sequences **15a**, **16a**, **17a**, **18a** and **19a** isolated by HPLC at 2 ug/mL over 120 s in 1 × PBS. After foldamer ligand immobilization, the sensors were washed for 60 s in the same buffer, before to record a second baseline for 120 s, again in 25mM Na_2HPO_4 (pH 7.4), 1mM EDTA, 250 mM NaCl and 0.05% Tween20. Serial column dilutions (× 2) of the HU in 25mM Na_2HPO_4 (pH 7.4), 1mM EDTA, 250 mM NaCl and 0.05% Tween20 were analysed, keeping the last well of the association column free of protein for referencing. Association lasted 120 s, followed by dissociation for another 120 s. The curves were fitted to binding models using the Octet analysis studio 13.0 software and replotted in Excel. The absence of unspecific binding of the proteins to streptavidin was confirmed

by running a kinetic assay in a single well on a SA sensor with no immobilized foldamer at the highest screened protein concentration (Data not Shown).

10.References

1. D. Bindl, E. Heinemann, P. K. Mandal and I. Huc, *Chem. Commun.*, 2021, **57**, 5662-5665.
2. J. Buratto, C. Colombo, M. Stupfel, S. J. Dawson, C. Dolain, B. Langlois d'Estaintot, L. Fischer, T. Granier, M. Laguerre, B. Gallois and I. Huc, *Angew. Chem. Int. Ed.*, 2014, **53**, 883-887.
3. X. Hu, S. J. Dawson, P. K. Mandal, X. de Hatten, B. Baptiste and I. Huc, *Chem. Sci.*, 2017, **8**, 3741-3749.
4. B. Baptiste, C. Douat-Casassus, K. Laxmi-Reddy, F. Godde and I. Huc, *J. Org. Chem.*, 2010, **75**, 7175-7185.
5. L. Schrödinger, 2021, Maestro, New York,NY.
6. V. Corvaglia, D. Carbajo, P. Prabhakaran, K. Ziach, P. K. Mandal, V. D. Santos, C. Legeay, R. Vogel, V. Parissi, P. Pourquier and I. Huc, *Nucleic Acids Res.*, 2019, **47**, 5511-5521.
7. L. Thunus and M. Dejardin-Duchene, *J. Pharm. Belg.*, 1969, **24**, 3-21.
8. R. Young and C. K. Chang, *J. Am. Chem. Soc.*, 1985, **107**, 898-909.
9. WO2008146774, 2008.
10. V. Corvaglia, F. Sanchez, F. S. Menke, C. Douat and I. Huc, *Chem. Eur. J.*, 2023, **29**, e202300898.
11. V. Kleene, V. Corvaglia, E. Chacin, I. Forne, D. B. Konrad, P. Khosravani, C. Douat, C. F. Kurat, I. Huc and A. Imhof, *Nucleic Acids Res*, 2023, **51**, 9629-9642.
12. M. Loos, L. Thurecht, J. Wu, V. Corvaglia, Z. Liu, V. Pophristic, M. Zacharias and I. Huc, *Chem Sci*, 2026, **17**, 3198-3211.
13. A. A. McCarthy, R. Barrett, A. Beteva, H. Caserotto, F. Dobias, F. Felisaz, T. Giraud, M. Guijarro, R. Janocha, A. Khadrouche, M. Lentini, G. A. Leonard, M. Lopez Marrero, S. Malbet-Monaco, S. McSweeney, D. Nurizzo, G. Papp, C. Rossi, J. Sinoir, C. Sorez, J. Surr, O. Svensson, U. Zander, F. Cipriani, P. Theveneau and C. Mueller-Dieckmann, *J. Synchrotron Radiat.*, 2018, **25**, 1249-1260.
14. W. Kabsch, *Acta Crystallogr., Sect. D: Biol. Crystallogr.*, 2010, **66**, 125-132.
15. M. Cianci, G. Bourenkov, G. Pompidor, I. Karpics, J. Kallio, I. Bento, M. Roessle, F. Cipriani, S. Fiedler and T. R. Schneider, *J. Synchrotron Radiat.*, 2017, **24**, 323-332.
16. C. Vonrhein, C. Flensburg, P. Keller, A. Sharff, O. Smart, W. Paciorek, T. Womack and G. Bricogne, *Acta Crystallogr., Sect. D: Biol. Crystallogr.*, 2011, **67**, 293-302.
17. P. R. Evans, *Acta Crystallogr., Sect. D: Biol. Crystallogr.*, 2006, **62**, 72-82.
18. P. R. Evans and G. N. Murshudov, *Acta Crystallogr., Sect. D: Biol. Crystallogr.*, 2013, **69**, 1204-1214.
19. G. M. Sheldrick, *Acta Crystallogr., Sect. A: Found. Crystallogr.*, 2008, **64**, 112-122.
20. G. M. Sheldrick, *Acta Crystallogr., Sect. C: Cryst. Struct. Commun.*, 2015, **71**, 3-8.
21. O. V. Dolomanov, L. J. Bourhis, R. J. Gildea, J. A. K. Howard and H. Puschmann, *J. Appl. Crystallogr.*, 2009, **42**, 339-341.
22. P. Emsley, B. Lohkamp, W. G. Scott and K. Cowtan, *Acta Crystallogr. Sect. D: Struct. Biol.*, 2010, **66**, 486-501.
23. A. L. Spek, *Acta Crystallogr., Sect. D: Biol. Crystallogr.*, 2009, **65**, 148-155.
24. Bruker, 2012, SAINT, Bruker AXS Inc., Madison, Wisconsin, USA.
25. G. M. Sheldrick, SADABS, 1996.University of Göttingen, Germany.
26. L. Farrugia, *J. Appl. Cryst.*, 2012, **45**, 849-854.
27. K. K. Swinger, K. M. Lemberg, Y. Zhang, P. A. Rice. *EMBO J.*, 2003;**22,** 3749-60.
